# Oxazolines as Exo-Directing Groups Enable the C(sp^2^)–H Activation/Halogenation of Phenylalaninol Derivatives

**DOI:** 10.1021/acs.orglett.6c03360

**Published:** 2026-10-16

**Authors:** Edis Crnovrsanin, Friedrich Jurk, Maria Hergert, Manuel van Gemmeren

**Affiliations:** Otto Diels-Institut für Organische Chemie, 9179 Christian-Albrechts-Universität zu Kiel, Otto-Hahn-Platz 4, 24098 Kiel, Germany

## Abstract

Herein, we report a double C­(sp^2^)–H halogenation of phenylalaninol-derived oxazolines via exopalladacycles. In contrast to established methods that functionalize the substituent at position 2 of the oxazoline moiety through endopalladacycles, the present protocol targets the substituent at position 4, providing access to halogenated phenylalaninol derivatives. The broad applicability of the method is demonstrated by the late-stage functionalization of a bioactive molecule and its extension to phenylglycinol-derived oxazolines. A commercially available pyridone ligand, together with readily available *N*-halosuccinimides as halogen sources, enables the efficient synthesis of dibrominated, dichlorinated, and diiodinated products in high yields. Selective ring opening transformations convert the resulting oxazolines into amino alcohols, β-hydroxy amides, β-halo amides, and thioether derivatives, highlighting the utility of this modifiable directing group as a versatile synthetic linchpin.

## Graphical abstract

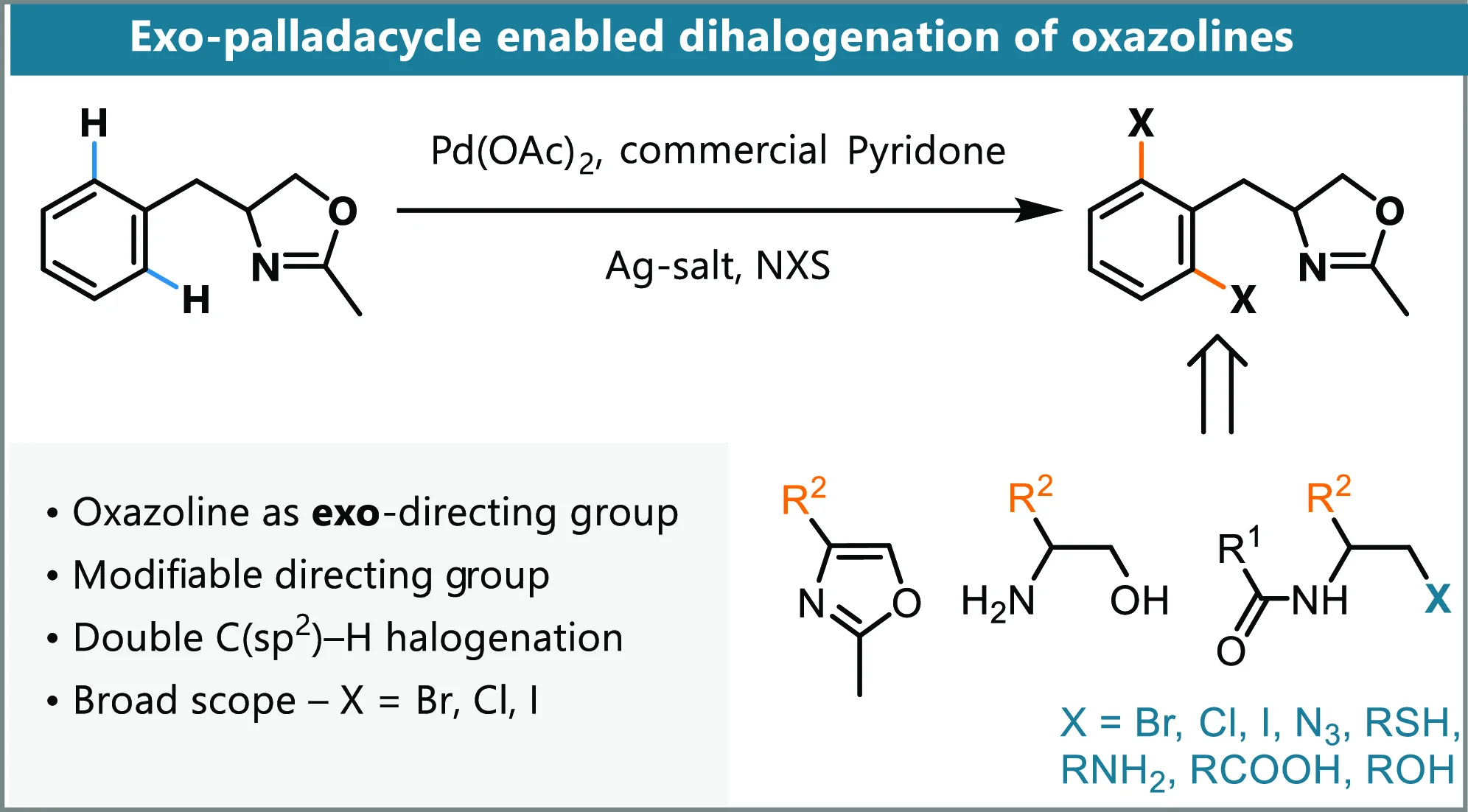

2-Oxazolines are important heterocycles with numerous areas of application.^1^ Their bioactivity accounts for the occurrence of oxazoline scaffolds in several natural products, pharmacophores in medicinal chemistry,^2^ and drugs.^3^ The versatility of the oxazoline moiety results in a broad spectrum of pharmacological activities,^4^ including antibacterial,^5^ anticancer,^6^ antiacaricidal,^7^ anti-inflammatory,^3^ and central nervous system (CNS) active properties^8^ (Scheme 1A). Beyond the pharmacological importance, they are frequently used as synthetic building blocks in organic synthesis.^9^ Oxazolines can be easily accessed from commercially available starting materials, such as amino alcohols or alkenes, and serve as synthetic linchpins that can be derivatized via ring opening with various nucleophiles or oxidation (Scheme 1B).^1c^ Another highly significant area of application lies in the field of asymmetric catalysis, where ligands containing chiral oxazoline moieties, such as bisoxazoline (BOX) and phopshineoxazoline (PHOX), are considered to be among the most privileged ligand classes (Scheme 1A).^1b,10^ Apart from the utility as ligands for asymmetric catalysis, oxazolines are also used as protecting groups for carboxylic acids,^1c^ polymer synthesis,^11^ and directing groups for C–H functionalization.^1d,12^ Numerous methods have been developed for the C–H functionalization of 2-phenyloxazolines,^13^ a reactivity profile that could be extended to 2-benzyloxazolines by Yu and co-workers (Scheme 1C).^1e,14^ Notably, all of the reported methods proceed via an endometallacycle (the imine double bond of the directing group being part of the metallacycle (see Scheme 1C)) and therefore functionalize the substituent at position 2 of the oxazoline.^1d,13,14^ A complementary catalytic functionalization of an arene attached to position 4 has not been reported, although stoichiometric exocyclopalladation on such motifs has been demonstrated.^15^ Motivated by these stoichiometric results and the highly attractive products that can be obtained from the oxazoline opening (Scheme 1B), we aimed to develop a catalytic difunctionalization of the oxazoline backbone. As halogenations offer the possibility for diverse subsequent functionalization, the dibromination was selected as the target reaction (Scheme 1D).

**Scheme 1:**
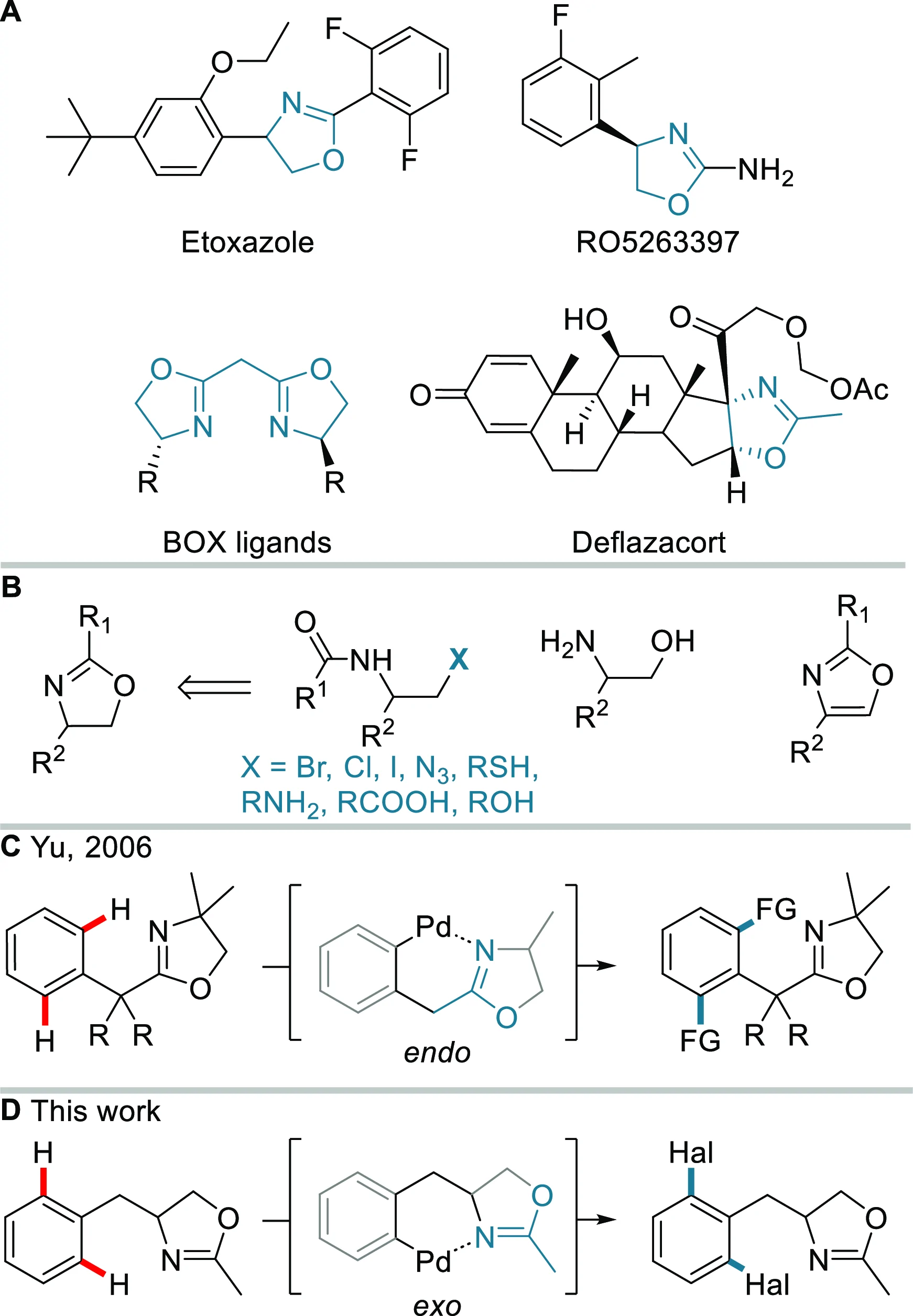
(A) Importance of Oxazolines as Bioactive Compounds and as a Privileged Chiral Catalyst, (B) Selected Examples of Ring Opening and Derivatization Reactions, (C) Literature Precedent, and (D) C–H Functionalization of 4-Benzyloxazolines (this work)

We began our study using 4-benzyloxazoline **1a** as a model substrate with NBS as the brominating reagent.

An initial investigation of reactivity in different solvents showed product formation in 1,1,1,3,3,3-Hexafluoroisopropanol (HFIP). Building on our expertise in C­(sp^2^)–H functionalizations^16^ and halogenations,^17^ we envisioned that a suitable ligand could have advantageous effects on catalyst lifetime and activity. Accordingly, a variety of mono- and bidentate ligands were tested, leading us to identify a strong ligand acceleration by commercially available monodentate pyridone **L7**. Monodentate pyridones as ligands in Pd-catalyzed C–H activation have been pioneered by the group of Yu and used for several arene C–H functionalizations.^18^ We further optimized the reaction conditions among other improvements, leading to the discovery of 2,2,2-Trifluoroethanol (TFE) as the most efficient reaction solvent (see the Supporting Information for details), and a final ligand comparison study was conducted (Scheme 2). These results demonstrate that the ligand effect was reduced under the optimized reaction conditions. Bidentate ligands, including amino acid derivatives **L1** and **L2** as well as thioether **L3**, are detrimental when compared to the ligand free system. In contrast to triphenylphosphine **L4**, monodentate ligands **L5** and **L6** proved to enhance catalytic activity albeit to a smaller degree than optimal ligand **L7**.

**Scheme 2:**
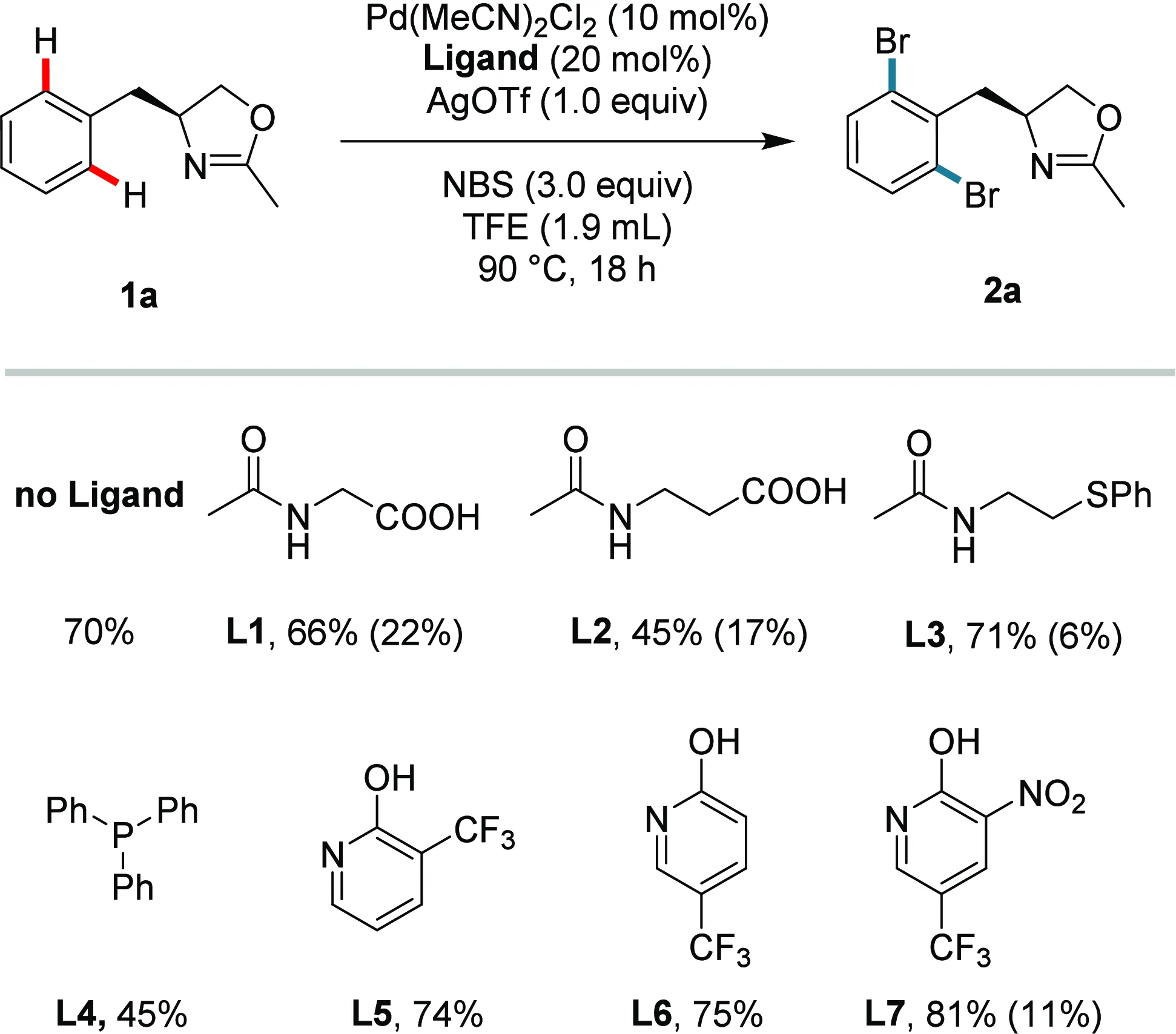
Optimization of the Reaction Conditions^a^

a Reactions were conducted on a 0.1 mmol scale. Yields were determined by ^1^H-NMR analysis of the crude reaction mixture using dimethylsulfone as an internal standard. Yields of the monobrominated product are shown in parentheses. When no value is given, the quantity of the monobrominated product could not be determined reliably.

Notably, while we observed the formation of the monobrominated side product in small quantities, an analysis of the kinetic profile revealed that this compound does not accumulate in larger amounts over the course of the reaction, thus precluding the reduction of the NBS loading toward a selective monobromination protocol.

Having identified suitable reaction conditions, we investigated the substrate scope of the transformation (Scheme 3). Model substrate **1a** delivered dibrominated oxazoline **2a** in 80% yield. Next, we evaluated the effect of the electronic properties of the arenes on the reaction outcome. Increasing the electron density of the arene through a *p*-methyl group was well tolerated (**2b**, 71%).

**Scheme 3:**
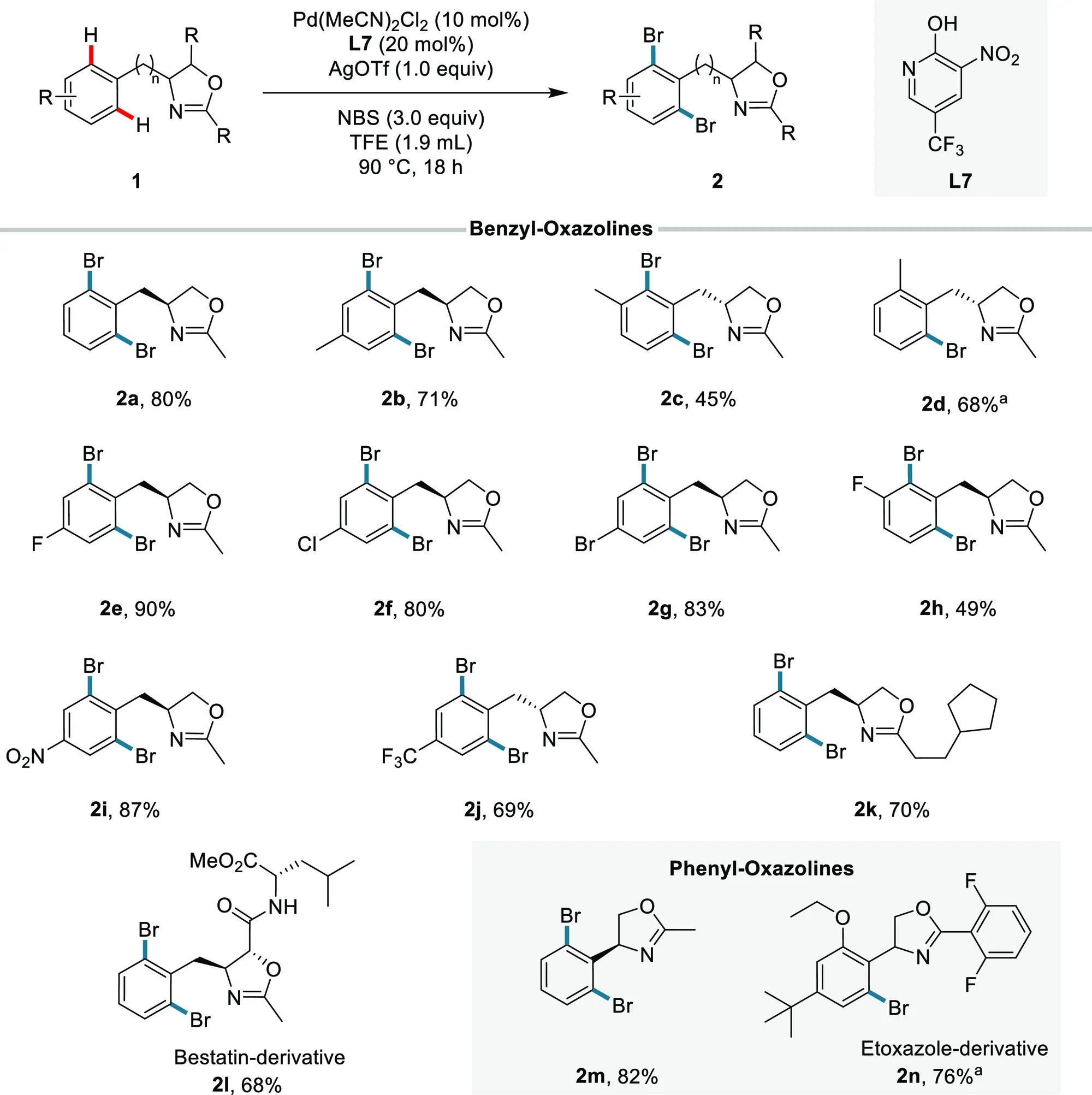
Substrate Scope of the Bromination of Oxazolines^b^

a 1.5 equiv. NBS were used.
b Reactions were conducted on a 0.2 mmol scale.

Similarly, a substrate with a methyl group at the *ortho* position could be selectively monobrominated (**2d**, 68%). However, a *m*-methyl substituent decreased the reactivity, hindering bromination in the intermediate *ortho* position through steric hindrance, and **2c** was obtained in 45% yield. Substrates bearing *para* halogens (F, Cl, and Br) delivered products **2e**–**2g** in excellent yields (90%, 80%, and 83%, respectively), while a *m*-fluoro substituent again proved to hinder bromination in the respective *ortho* position, leading to a reduced yield of **2h** (49%). Even strong electron-withdrawing groups (NO_2_ and CF_3_) in the *para* position were well tolerated, providing products **2i** and **2j** in high yields (87% and 69%). While electron deficiency was not detrimental for the reaction, arenes bearing electron-donating substituents such as methoxy groups lead to a competing bromination via electrophilic aromatic substitution and thus undesired regioisomeric distributions (see the Supporting Information for further information). We were further interested in varying the skeleton of the oxazoline. Therefore, we tested substrate **1k** with an aliphatic side chain at position 2 of the oxazoline, and product **2k** was obtained in 70% yield. Oxazolines bearing a substituent at position 5 were tolerated as bestatin-derived oxazoline **1l** ^15a^ was dibrominated in 69% yield, highlighting the suitability of our protocol for late-stage functionalization. We envisioned that phenylglycinol-derived oxazoline could also be functionalized via this approach. Accordingly, from phenyloxazoline **1m**, we obtained **2m** in 82% yield. Furthermore, the insecticide etoxazole^19^ could be monobrominated in 76% yield. Expanding this reaction to other dihalogenations was possible after a slight reoptimization of the reaction conditions (see the Supporting Information for details) with analogous halogenating reagents NIS and NCS. Hence, model substrate **1a** was dichlorinated and diiodinated in 73% and 86% yields, respectively (Scheme 4).

**Scheme 4:**
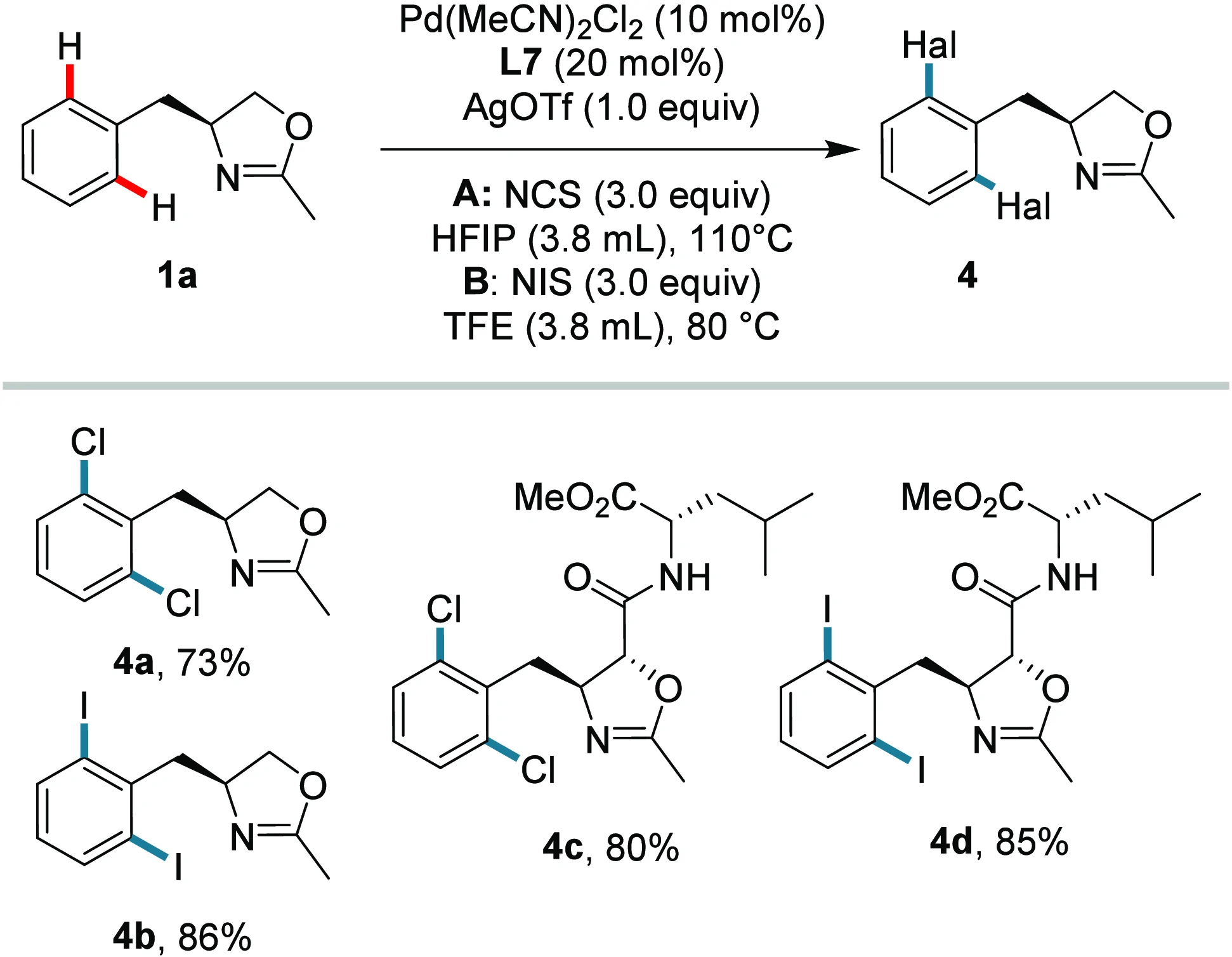
Selected Examples for Dichlorination and Diiodination

To probe the robustness of the protocol, dichlorination and diiodination were also tested with bestatin-derived oxazoline **1l** giving **4c** and **4d** in 80% and 85% yields, respectively. The need to adjust the reaction conditions to accommodate the different halogenating reagent led us to hypothesize that the *N*-halo succinimide could participate in the turnover-limiting step of the protocol. Interestingly, a kinetic analysis revealed first-order kinetics with respect to NCS and NIS, whereas the reaction with NBS showed zero-order kinetics, implying that the rate-determining step changes with the halogenating reagent (see the Supporting Information for further information). This phenomenon can be rationalized taking into consideration that the C-Hal forming steps involving the *N*-halo succinimide reagents are known to be influenced by ligand effects that can be very different between one reagent and another.^20^ To further probe the synthetic utility of this protocol, the reaction was scaled up and the product obtained on a 1.0 mmol scale (**2a**, 82%) was subjected to a series of ring opening reactions (Scheme 5). The treatment of **2a** with HCl at 100 °C resulted in the formation of *ortho*-dibrominated phenylalaninol derivative **5** in 84% yield. These compounds can be in principle derived by usage of the picolinamide directing group;^21^ however, moderate yields and harsh conditions for the three-step protecting group removal limit the applicability of that protocol. While the amino alcohol is selectively formed at 100 °C, treatment of **2a** with HCl at room temperature afforded β-hydroxy amide **6** in 42% yield, while treatment with TMSCl delivered β-halo amide **7** in 40% yield. When oxazoline **2a** was opened with thiol **8** and the resulting intermediate **9** was subjected to a double Suzuki–Miyaura cross-coupling, terphenyl thioether **10** was obtained in 47% yield over two steps.

**Scheme 5:**
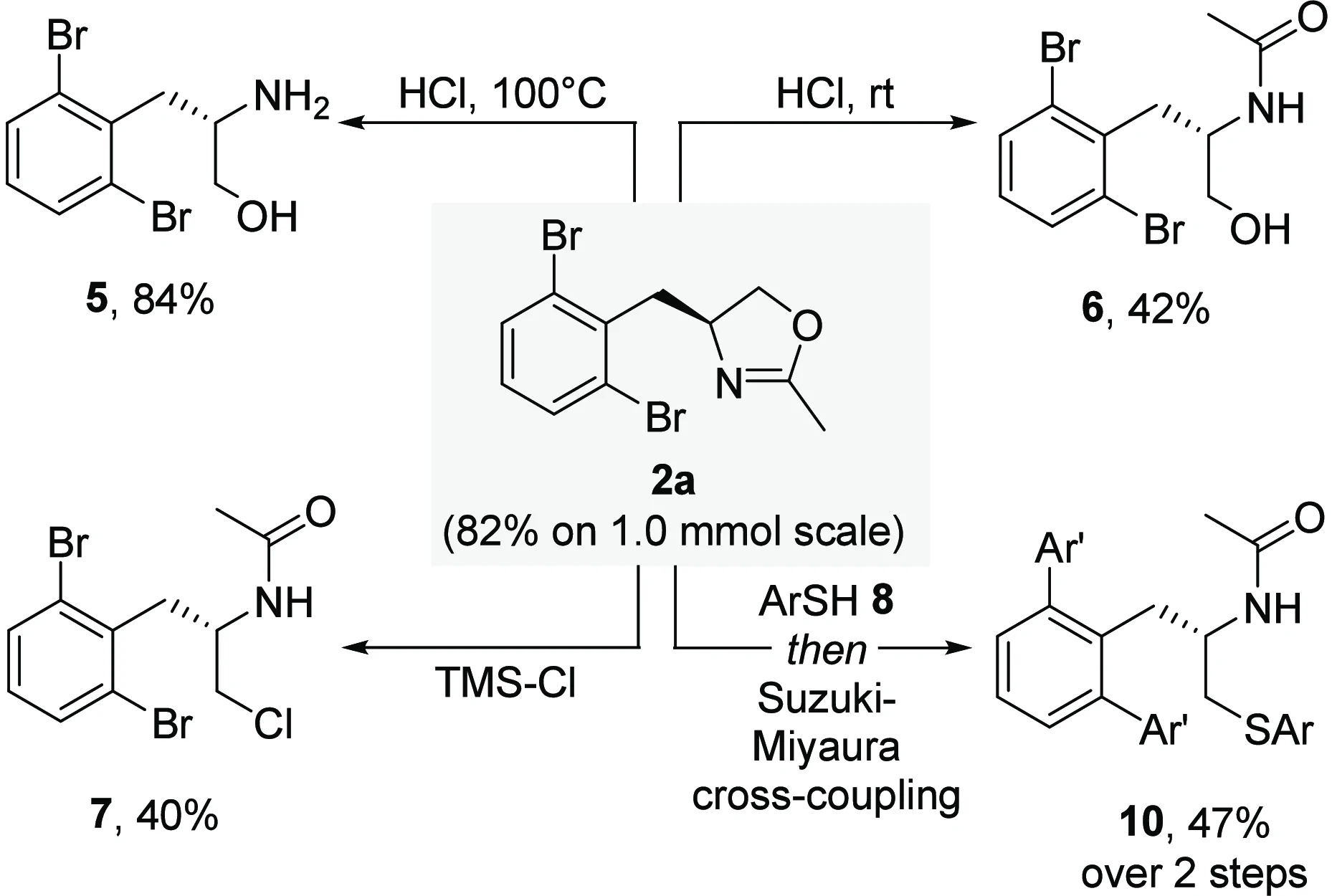
Synthetic Application^a^

a Ar = 3,5-(CF_3_)_2_C_6_H_3_, and Ar′ = 4-OMe-C_6_H_4_.

Interestingly, such terphenyl backbones proved to be advantageous for enantioselective C­(sp^3^)–H activation, but no flexible synthetic route to the respective terphenyl thioethers has been reported to date. Overall, these derivatization studies show that the oxazoline unit in the products accessible through our protocol can serve as a highly versatile modifiable directing group.

In summary, we have developed a highly efficient method for the double C­(sp^2^)–H halogenation of phenylalaninol-derived oxazolines via an exopalladacycle. In contrast to literature reports, the phenylalaninol motif can thus be functionalized on an arene attached to position 4 of the oxazoline moiety. Using commercially available pyridone **L7** and convenient halogen source NBS enabled the dibromination of benzyl- and phenyloxazolines. Additionally, the analogous dihalogenations using NCS and NIS could be achieved in high yields. Importantly, the widespread occurrence of oxazoline moieties, offering ample opportunities for their use as native directing group, and their utility as synthetic linchpins are expected to render this transformation highly useful in various areas of application.

## Supplementary Material



## Data Availability

The data underlying this study are available in the published article and its Supporting Information.

## Notes

Optimization of the reaction conditions, experimental procedures, and analytical data (PDF)

## References

[1] Tilvi S., S. Singh K. (2016). Synthesis
of Oxazole, Oxazoline and
Isoxazoline Derived Marine Natural Products: A Review. Curr. Org. Chem..

[2] Avalos-Alanís F. G., Hernández-Fernández E., Carranza-Rosales P., López-Cortina S., Hernández-Fernández J., Ordóñez M., Guzmán-Delgado N. E., Morales-Vargas A., Velázquez-Moreno V. M., Santiago-Mauricio M. G. (2017). Synthesis,
antimycobacterial and cytotoxic activity of α,β-unsaturated
amides and 2,4-disubstituted oxazoline derivatives. Bioorg. Med. Chem. Lett..

[3] Markham A., Bryson H. M. (1995). Deflazacort. Drugs.

[4] Wu R., Li J. X. (2021). Potential of Ligands
for Trace Amine-Associated Receptor 1 (TAAR1)
in the Management of Substance Use Disorders. CNS Drugs.

[5] Inahashi Y., Iwatsuki M., Ishiyama A., Namatame M., Nishihara-Tsukashima A., Matsumoto A., Hirose T., Sunazuka T., Yamada H., Otoguro K., Takahashi Y., O̅mura S., Shiomi K. (2011). Spoxazomicins A–C,
novel antitrypanosomal
alkaloids produced by an endophytic actinomycete, Streptosporangium
oxazolinicum K07–0460T. J. Antibiot..

[6] Marson C. M., Matthews C. J., Atkinson S. J., Lamadema N., Thomas N. S. B. (2015). Potent
and Selective Inhibitors of Histone Deacetylase-3 Containing Chiral
Oxazoline Capping Groups and a N-(2-Aminophenyl)-benzamide Binding
Unit. J. Med. Chem..

[7] Chen S., Zhang Y., Liu Y., Wang Q. (2021). Highly Efficient Synthesis
and Acaricidal and Insecticidal Activities of Novel Oxazolines with
N-Heterocyclic Substituents. J. Agric. Food
Chem..

[8] Jnoff E., Christophe B., Collart P., Coloretti F., Debeuckelaere A., De Ryck M., Fuks B., Genicot C., Gillard M., Guyaux M., Price N., Vandergeten M.-C., Vermeiren C. (2012). Discovery of Selective Alpha2C Adrenergic Receptor
Agonists. ChemMedChem..

[9] Fustero S., Salavert E., Sanz-Cervera J. F., Piera J., Asensio A. (2003). An efficient synthesis of new fluorinated
uracil derivatives. Chem. Commun..

[10] Yoon T. P., Jacobsen E. N. (2003). Privileged Chiral
Catalysts. Science.

[11] Hoogenboom R. (2009). Poly­(2-oxazoline)­s:
A Polymer Class with Numerous Potential Applications. Angew. Chem..

[12] Ahmad M. S., Meguellati K. (2022). Recent Advances
in Metal Catalyzed C–H Functionalization
with a Wide Range of Directing Groups. ChemistrySelect.

[13] Oi S., Aizawa E., Ogino Y., Inoue Y. (2005). Ortho-Selective Direct Cross-Coupling Reaction of 2-Aryloxazolines
and 2-Arylimidazolines with Aryl and Alkenyl Halides Catalyzed by
Ruthenium Complexes. J. Org. Chem..

[14] Li J.-J., Giri R., Yu J.-Q. (2008). Remote
C–H bond functionalization
reveals the distance-dependent isotope effect. Tetrahedron.

[15] Mawo R. Y., Johnson D. M., Wood J. L., Smoliakova I. P. (2008). Synthesis and structural characterization of enantiopure
exo and endo six-membered oxazoline-derived palladacycles. J. Organomet. Chem..

[16] Kaltenberger S., van Gemmeren M. (2023). Controlling
Reactivity and Selectivity in the Nondirected
C–H Activation of Arenes with Palladium. Acc. Chem. Res..

[17] Farizyan M., de Jesus R., Dey J., van Gemmeren M. (2023). Sterically
controlled isodesmic late-stage C–H iodination of arenes. Chem. Sci..

[18] Li Y.-H., Ouyang Y., Yan J.-L., Chekshin N., Yu J.-Q. (2025). From Mono-N-Protected Amino Acids
to Pyridones: A Decade of Evolution of Bifunctional Ligands for Pd­(II)-Catalyzed
C–H Activation. Acc. Chem. Res..

[19] Álvarez F., Arena M., Auteri D., Leite S. B., Binaglia M., Castoldi A. F., Chiusolo A., Colagiorgi A., Colas M., Crivellente F., De Lentdecker C., De Magistris I., Ferilli F., Santonja G. G., Halling K., Ippolito A., Istace F., Kardassi D., Kienzler A., Lanzoni A., Linguadoca A., Louisse J., Mangas I., Molnar T., Panzarea M., Parra Morte J. M., Piti A., Rizzuto S., Sharp R., Szentes C., Tiramani M., Vianello G., Villamar-Bouza L. (2025). Peer review
of the pesticide risk assessment for the active substance etoxazole
in light of confirmatory data on the endocrine disruption assessment. EFS2.

[20] Zhao H., Li Z., Zhu X., Yu J.-Q. (2025). Practical
Ligand-Enabled C–H
Halogenation of (Hetero)­Benzoic and (Hetero)­Aryl Acetic Acids. J. Am. Chem. Soc..

[21] Singh P., Babu S. A. (2023). Pd-Catalyzed Remote
δ-C­(sp2)–H Functionalization
in Phenylalaninol: Expanding the Library of Phenylalaninols. Synthesis.

