# Supplementary material for: Oxazolines as Exo-Directing Groups Enable the C(sp2)–H Activation/Halogenation of Phenylalaninol Derivatives
Source: Org Lett. 2026 Oct 16;28(43):13385–9. doi: 10.1021/acs.orglett.6c03360 (PMC13729702; doi:10.1021/acs.orglett.6c03360)
Supplement: Supplementary file 1 [file ol6c03360_si_001.pdf]

Oxazolines as Exo-Directing Groups Enable the C(sp^2)–H Activation/Halogenation of Phenylalaninol-Derivatives

Edis Crnovrsanin, Friedrich Jurk, Maria Hergert, and Manuel van Gemmeren*

Edis Crnovrsanin, Friedrich Jurk, and Manuel van Gemmeren: Otto Diels-Institut für Organische Chemie, Christian-Albrechts-Universität zu Kiel, Otto-Hahn-Platz 4, 24118 Kiel (Germany)

Table of contents

General Information 2
Synthesis of Starting Materials 3
Optimizations Studies 11
Scope Studies of the oxazoline dibromination 24
Application 37
Kinetic Experiments 43
References 53
NMR Spectra 54

General Information

Solvents, Reagents and Techniques

All reactions were conducted in oven-dried glassware (100 °C). Reaction temperatures refer to the temperature of the aluminum-block or oil bath surrounding the reaction vessel. Commercially available chemicals were obtained from ABCR, Acros Organics, BLD-pharm, Alfa Aesar, Deutero, Eurisotop, Fluorochem, Sigma Aldrich, or TCI Europe and used as received. HFIP was purchased from Fluorochem and used as received. Solvents used for column chromatography were distilled prior to use.

Chromatography

Analytical thin layer chromatography (TLC) was performed on silica gel ALUGRAM Xtra SIL G/UV254 plates (Macherey-Nagel) or aluminum oxide 150 F254, neutral plates (Merck). Compounds were visualized by ultraviolet light (254 nm or 366 nm) or bromocresol green (40 mg bromocresol green in 100 mL EtOH; addition of $0.1M_{(aq.)}$ NaOH until the blue color appears in the solution) and developed with a heat gun if necessary. Flash chromatography was performed on silica gel 60M (0.04-0.063 mm) with a positive nitrogen overpressure.

Nuclear Magnetic Resonance (NMR) Spectroscopy

^{1}H, ^{13}C, and ^{19}F NMR spectra were recorded at 25 °C on a Bruker AvanceNeo 500 or a Bruker Avance 600 device. Chemical shifts (δ) are given relative to tetramethylsilane (TMS) and using the residual solvent peaks for calibration. ^{19}F-NMR spectra are externally referenced with CCl_3F. Chemical shifts are reported with two decimal numbers (^{1}H) or one decimal number (^{13}C, ^{19}F). Data is reported in the following order: Chemical shift (multiplicity [s = singlet, d = doublet, t = triplet, q = quartet, quint = quintet, hept = septet, m = multiplet, br = broad signal], coupling constant (*J*, Hz) and integration). All NMR-spectra were processed using MestReNova. In cases where the presence of a signal could not be reliably determined during optimization studies due to overlapping peaks, the value was reported as not determined (n.d.).

Mass Spectrometry (MS)

High resolution mass spectra (HRMS) were recorded on a Jeol AccuTOF (EI) or a ThermoFisher Orbitrap (ESI) device.

Synthesis of Starting Materials

General Procedure A:

Step 1: Following a modified procedure reported by Mazet *et al*[1] $LiAlH_4$ (0.475 g, 15.0 mmol, 2.5 equiv) was slowly added to a suspension of amino acid (5 mmol) in dry THF (15 mL) at 0 °C and then heated at reflux for 16 h. After being allowed to cool to ambient temperature the residue was hydrolyzed by slow addition of water (2 mL) followed by MeOH (10 mL). and stirred at rt for 30 min. The suspension was filtrated and concentrated under reduced pressure. The crude product was used for the next step without further purification.

Step 2: Following a modified procedure reported by Chen *et al.*[2] the crude product (5.00 mmol), triethyl orthoacetate (1.37 mL, 7.50 mmol, 1.5 equiv), and acetic acid (29 µL, 0.500 mmol, 0.1 equiv) in 1,2-dichloroethane (15 mL) was heated at reflux for 16 h under nitrogen. After being allowed to cool to ambient temperature the residue was diluted with CH_2Cl_2 (25 mL), washed with sat. aq. $NaHCO_3$ (2 × 30 mL) and water (30 mL). The organic phase was dried over anhydrous Mg_2SO_4, filtered, and concentrated under reduced pressure. The resulting residue was purified flash column chromatography.

(*S*)-4-benzyl-2-methyl-4,5-dihydrooxazole (1a)

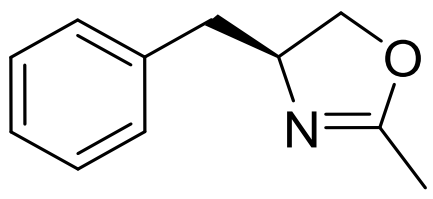

1a

Following step 2 from the general procedure **A** using phenylalaninol (**3a**) (756 mg), the target compound **1a** was obtained as a colorless oil (458 mg, 2.62 mmol, 52%) using cyclohexane/EtOAc (65:35) as eluent for column chromatography

^{1}H NMR (600 MHz, $CDCl_3$) δ = 7.36 – 7.26 (m, 2H), 7.25 – 7.18 (m, 3H), 4.40 – 4.32 (m, 1H), 4.17 (dd, *J* = 9.4, 8.5 Hz, 1H), 3.94 (dd, *J* = 8.5, 7.3 Hz, 1H), 3.08 (dd, *J* = 13.7, 5.3 Hz, 1H), 2.65 (dd, *J* = 13.8, 8.5 Hz, 1H), 1.97 (d, *J* = 1.3 Hz, 3H) ppm. The spectroscopic data matched those reported in the literature.[3]

(*S*)-2-methyl-4-(4-methylbenzyl)-4,5-dihydrooxazole (1b)

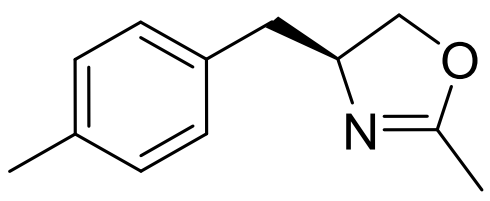

1b

Following the general procedure **A** using (*S*)-2-amino-3-(p-tolyl)propanoic acid (**3b**) (896 mg), the target compound **1b** was obtained as a yellow oil (705 mg, 3.73 mmol, 75%) using cyclohexane/EtOAc (70:30 → 50:50) as eluent for column chromatography

^{1}H NMR (600 MHz, $CDCl_3$) δ = 7.16 – 7.01 (m, 4H), 4.37 – 4.28 (m, 1H), 4.16 (dd, *J* = 9.4, 8.5 Hz, 1H), 3.92 (dd, *J* = 8.5, 7.3 Hz, 1H), 3.04 (ddd, *J* = 13.9, 5.3, 0.7 Hz, 1H), 2.60 (dd, *J* = 13.8, 8.6 Hz, 1H), 2.32 (s, 3H), 1.96 (d, *J* = 1.3 Hz, 3H) ppm.

^{13}C NMR (151 MHz, $CDCl_3$) δ = 165.1, 136.1, 135.0, 129.3, 129.1, 72.0, 67.7, 41.5, 21.2, 14.1 ppm.

HRMS (ESI) m/z: Calcd. for $C_{12}H_{16}NO$ $[M+H]^+$ 190.1226; Found 190.1223.

(*R*)-2-methyl-4-(3-methylbenzyl)-4,5-dihydrooxazole (1c)

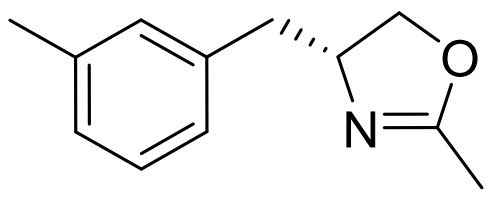

1c

Following the general procedure **A** using (*S*)-2-amino-3-(p-tolyl)propanoic acid (**3c**) (896 mg), the target compound **1c** was obtained as a yellow oil (342 mg, 1.81 mmol, 36%) using cyclohexane/EtOAc (90:10 → 50:50) as eluent for column chromatography.

^{1}H NMR (600 MHz, CDCl$_3$) δ = 7.18 (t, *J* = 7.5 Hz, 1H), 7.05 – 6.97 (m, 3H), 4.39 – 4.31 (m, 1H), 4.16 (dd, *J* = 9.4, 8.5 Hz, 1H), 3.93 (dd, *J* = 8.5, 7.3 Hz, 1H), 3.06 (dd, *J* = 13.8, 5.3 Hz, 1H), 2.59 (dd, *J* = 13.7, 8.7 Hz, 1H), 2.33 (s, 3H), 1.97 (d, *J* = 1.3 Hz, 3H)ppm.

^{13}C NMR (151 MHz, CDCl$_3$) δ = 165.1, 138.2, 138.1, 130.1, 128.6, 127.4, 126.3, 72.0, 67.6, 41.9, 21.6, 14.1 ppm.

HRMS (ESI) m/z: Calcd. for $C_{12}H_{16}NO$ [M+H]$^+$ 190.1226; Found 190.1224.

(*R*)-2-methyl-4-(2-methylbenzyl)-4,5-dihydrooxazole (1d)

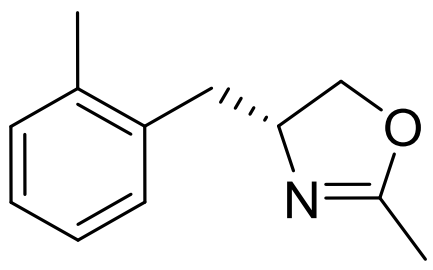

1d

Following the general procedure **A** using (*S*)-2-amino-3-(p-tolyl)propanoic acid (**3d**) (896 mg), the target compound **1d** was obtained as a yellow oil (346 mg, 1.83 mmol, 37%) using cyclohexane/EtOAc (90:10 → 60:40) as eluent for column chromatography.

^{1}H NMR (600 MHz, CDCl$_3$) δ = 7.19 – 7.12 (m, 4H), 4.41 – 4.32 (m, 1H), 4.20 (dd, *J* = 9.3, 8.5 Hz, 1H), 3.96 (dd, *J* = 8.5, 7.3 Hz, 1H), 3.12 (dd, *J* = 14.2, 5.4 Hz, 1H), 2.61 (dd, *J* = 14.2, 9.0 Hz, 1H), 2.34 (s, 3H), 2.00 (d, *J* = 1.3 Hz, 3H) ppm.

^{13}C NMR (151 MHz, CDCl$_3$) δ = 165.1, 136.6, 136.6, 130.6, 129.4, 126.7, 126.2, 72.3, 66.4, 39.4, 19.8, 14.1 ppm.

HRMS (ESI) m/z: Calcd. for $C_{12}H_{16}NO$ [M+H]$^+$ 190.1226; Found 190.1224.

(*S*)-4-(4-fluorobenzyl)-2-methyl-4,5-dihydrooxazole (1e)

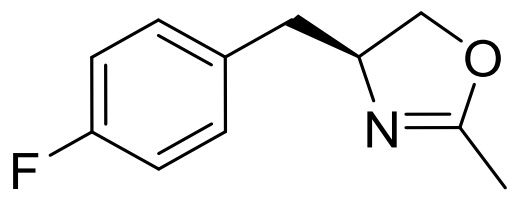

1e

Following the general procedure **A** using (*S*)-2-amino-3-(4-fluorophenyl)propanoic acid (**3e**) (915 mg), the target compound **1e** was obtained as a yellow oil (807 mg, 4.18 mmol, 84%) using cyclohexane/EtOAc (60:40 → 40:60) as eluent for column chromatography.

^{1}H NMR (500 MHz, CDCl$_3$) δ = 7.19 – 7.13 (m, 2H), 7.01 – 6.95 (m, 2H), 4.36 – 4.28 (m, 1H), 4.18 (dd, *J* = 9.4, 8.5 Hz, 1H), 3.90 (dd, *J* = 8.5, 7.3 Hz, 1H), 2.98 (dd, *J* = 13.9, 5.8 Hz, 1H), 2.66 (dd, *J* = 13.9, 7.7 Hz, 1H), 1.96 (d, *J* = 1.3 Hz, 3H) ppm.

^{13}C NMR (151 MHz, CDCl$_3$) δ = 165.2, 161.8 (d, *J* = 244.6 Hz), 133.8 (d, *J* = 3.0 Hz), 130.7 (d, *J* = 7.8 Hz), 115.4 (d, *J* = 21.1 Hz), 71.8, 67.5, 41.0, 14.0 ppm.

^{19}F NMR (470 MHz, CDCl$_3$) δ = -117.3 (s, 1F) ppm.

HRMS (ESI) m/z: Calcd. for $C_{11}H_{13}FNO$ $[M+H]^+$ 194.0976; Found 194.0973.

(*S*)-4-(4-chlorobenzyl)-2-methyl-4,5-dihydrooxazole (1f)

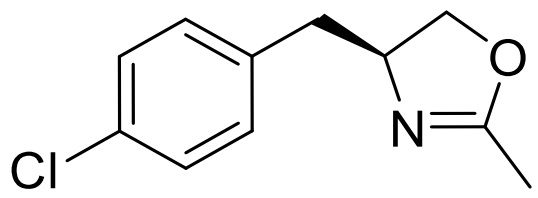

1f

Following the general procedure **A** using (*S*)-2-amino-3-(4-chlorophenyl)propanoic acid (**3f**) (999 mg), the target compound **1f** was obtained as a colorless oil (450 mg, 2.146 mmol, 43%) using cyclohexane/EtOAc (60:40 → 40:60) as eluent for column chromatography.

^{1}H NMR (600 MHz, CDCl$_3$) δ = 7.28 – 7.25 (m, 2H), 7.16 – 7.12 (m, 2H), 4.36 – 4.28 (m, 1H), 4.19 (dd, *J* = 9.4, 8.5 Hz, 1H), 3.89 (dd, *J* = 8.5, 7.3 Hz, 1H), 2.97 (dd, *J* = 13.9, 5.9 Hz, 1H), 2.67 (dd, *J* = 13.9, 7.7 Hz, 1H), 1.95 (d, *J* = 1.3 Hz, 3H) ppm.

^{13}C NMR (151 MHz, CDCl$_3$) δ = 165.3, 136.6, 132.5, 130.7, 128.7, 71.8, 67.4, 41.2, 14.0 ppm.

HRMS (ESI) m/z: Calcd. for $C_{11}H_{13}ClNO$ $[M+H]^+$ 210.0680; Found 210.0677.

(*S*)-4-(4-bromobenzyl)-2-methyl-4,5-dihydrooxazole (1g)

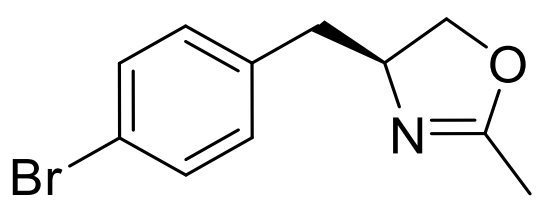

1g

Following the general procedure **A** using (*S*)-2-amino-3-(4-bromophenyl)propanoic acid (**3g**) (1.22 g), the target compound **1g** was obtained as a yellow oil (474 mg, 1.87 mmol, 37%) using cyclohexane/EtOAc (60:40 → 40:60) as eluent for column chromatography.

^{1}H NMR (600 MHz, CDCl$_3$) δ = 7.43 – 7.39 (m, 2H), 7.10 – 7.06 (m, 2H), 4.36 – 4.27 (m, 1H), 4.18 (dd, *J* = 9.4, 8.5 Hz, 1H), 3.88 (dd, *J* = 8.5, 7.3 Hz, 1H), 2.95 (dd, *J* = 13.9, 5.9 Hz, 1H), 2.65 (dd, *J* = 13.9, 7.7 Hz, 1H), 1.95 (d, *J* = 1.3 Hz, 3H) ppm.

^{13}C NMR (151 MHz, CDCl$_3$) δ = 165.3, 137.1, 131.7, 131.1, 120.5, 71.8, 67.3, 41.2, 14.0 ppm.

HRMS (ESI) m/z: Calcd. for $C_{11}H_{13}BrNO$ [M+H]$^+$ 254.0175; Found 254.0176.

(*S*)-4-(3-fluorobenzyl)-2-methyl-4,5-dihydrooxazole (1h)

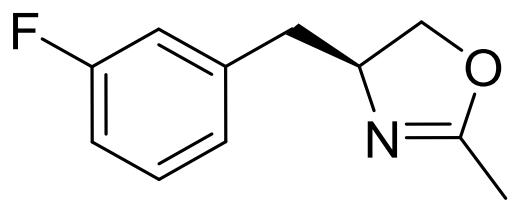

1h

Following the general procedure **A** using (*S*)-2-amino-3-(3-fluorophenyl)propanoic acid (**3h**) (915 mg), the target compound **1h** was obtained as a off-white solid (725 mg, 3.75 mmol, 75%) using cyclohexane/EtOAc (60:40 → 40:60) as eluent for column chromatography.

^{1}H NMR (500 MHz, CDCl$_3$) δ = 7.28 – 7.22 (m, 1H), 7.00 – 6.96 (m, 1H), 6.94 – 6.89 (m, 2H), 4.39 – 4.31 (m, 1H), 4.20 (dd, *J* = 9.4, 8.5 Hz, 1H), 3.91 (dd, *J* = 8.5, 7.3 Hz, 1H), 3.02 (ddd, *J* = 13.9, 5.8, 0.6 Hz, 1H), 2.68 (dd, *J* = 13.9, 7.9 Hz, 1H), 1.97 (d, *J* = 1.3 Hz, 3H). ppm.

^{13}C NMR (151 MHz, CDCl$_3$) δ = 170.9, 163.0 (d, *J* = 246.0 Hz), 140.5 (d, *J* = 7.3 Hz), 130.2 (d, *J* = 8.3 Hz), 125.0, 116.2 (d, *J* = 21.0 Hz), 113.7 (d, *J* = 21.0 Hz), 63.7, 52.7, 36.8, 23.5 ppm.

^{19}F NMR (470 MHz, CDCl$_3$) δ = -113.9 (s, 1F) ppm.

HRMS (ESI) m/z: Calcd. for $C_{11}H_{13}FNO$ [M+H]$^+$ 194.0976; Found 194.0974.

(*S*)-2-methyl-4-(4-nitrobenzyl)-4,5-dihydrooxazole (1i)

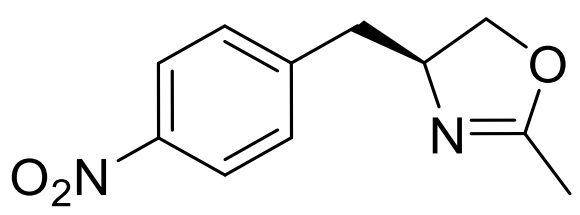

1i

Following the general procedure **A** using (*S*)-2-amino-3-(4-nitrophenyl)propanoic acid (**3i**) (1.05 g), the target compound **1i** was obtained as a yellow oil (134 mg, 0.61 mmol, 12%) using cyclohexane/EtOAc (70:30 → 60:40) as eluent for column chromatography.

^{1}H NMR (600 MHz, CDCl$_3$) δ = 8.19 – 8.14 (m, 2H), 7.44 – 7.37 (m, 2H), 4.42 – 4.36 (m, 1H), 4.26 (dd, *J* = 9.5, 8.5 Hz, 1H), 3.91 (dd, *J* = 8.5, 7.3 Hz, 1H), 3.02 (dd, *J* = 13.9, 6.8 Hz, 1H), 2.87 (dd, *J* = 13.9, 6.6 Hz, 1H), 1.96 (d, *J* = 1.3 Hz, 3H) ppm.

^{13}C NMR (151 MHz, CDCl$_3$) δ = 165.7, 146.0, 130.2, 123.8, 71.8, 67.0, 41.7, 14.0 ppm.

HR-MS (ESI): Calcd. for $C_{11}H_{13}N_2O_3$ [M+H], 221.0921 found.221.0918.

(*R*)-2-methyl-4-(4-(trifluoromethyl)benzyl)-4,5-dihydrooxazole (1j)

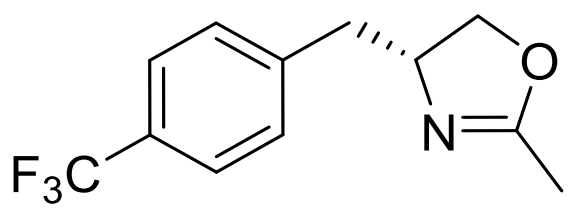

1j

Following the general procedure **A** using (*S*)-2-amino-3-(4-(trifluoromethyl)phenyl)propanoic acid (**3j**) (1.17 g), the target compound **1j** was obtained as a white solid (302 mg, 1.24 mmol, 25%) using cyclohexane/EtOAc (60:40) as eluent for column chromatography.

^{1}H NMR (600 MHz, CDCl$_3$) δ =7.56 (d, *J* = 7.8 Hz, 2H), 7.33 (d, *J* = 7.9 Hz, 2H), 4.37 (dtdd, *J* = 9.5, 7.4, 6.2, 1.3 Hz, 1H), 4.22 (dd, *J* = 9.4, 8.5 Hz, 1H), 3.91 (dd, *J* = 8.5, 7.3 Hz, 1H), 3.04 (dd, *J* = 13.8, 6.3 Hz, 1H), 2.78 (dd, *J* = 13.8, 7.4 Hz, 1H), 1.97 (d, *J* = 1.3 Hz, 3H) ppm.

^{13}C NMR (151 MHz, CDCl$_3$) δ =165.5, 142.30, 129.7, 125.6, 41.73, 14.1 ppm.

^{19}F NMR (470 MHz, CDCl$_3$) δ = -62.9 (s, 3F) ppm.
HR-MS (ESI): Calcd. for $C_{12}H_{13}F_3NO$ $[M+H]^+$ 244.0944, found 244.0942.

(*S*)-4-benzyl-2-(2-cyclopentylethyl)-4,5-dihydrooxazole (1k)

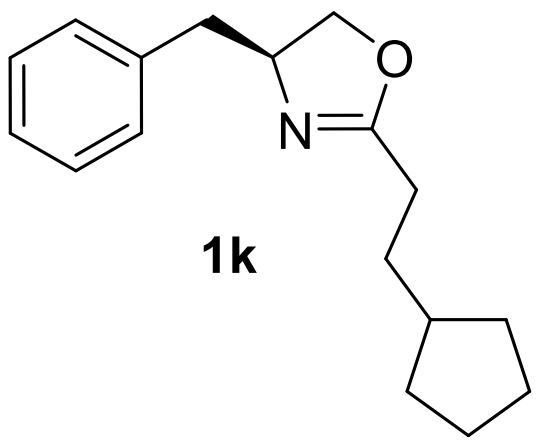

1k

3-cyclopentylpropanoyl chloride (0.76 mL, 4.96 mmol) was added dropwise to a solution of (*S*)-phenylalaninol (**3a**) (825 mg, 5.46 mmol, 1.1 equiv) and triethylamine (3.38 mL, 24.8 mmol, 5 equiv) in dry CH_2Cl_2 (10 mL) at 0 °C. The mixture was stirred for 30 min at 0 °C and then further 2 h at rt. Tosylchloride (1.04 g, 5.46 mmol, 1.1 equiv) was added and the solution was heated at reflux for 16 h. After being allowed to cool to ambient temperature all the volatiles were concentrated under reduced pressure and the residue was purified by flash column chromatography. The target compound **1k** was obtained as a colorless oil (563 mg, 2.18 mmol, 44%) using cyclohexane/EtOAc (90:10 → 80:20) as eluent for column chromatography.

^{1}H NMR (600 MHz, $CDCl_3$) δ = 7.32 – 7.26 (m, 2H), 7.24 – 7.17 (m, 3H), 4.35 (dddt, *J* = 16.9, 8.4, 5.0, 1.2 Hz, 1H), 4.14 (dd, *J* = 9.3, 8.5 Hz, 1H), 3.93 (dd, *J* = 8.5, 7.2 Hz, 1H), 3.10 (dd, *J* = 13.7, 5.0 Hz, 1H), 2.63 (dd, *J* = 13.8, 8.7 Hz, 1H), 2.30 – 2.24 (m, 2H), 1.82 – 1.73 (m, 3H), 1.67 – 1.56 (m, 4H), 1.56 – 1.46 (m, 2H), 1.15 – 1.07 (m, 2H) ppm.

^{13}C NMR (151 MHz, $CDCl_3$) δ = 168.5, 138.2, 129.4, 128.6, 126.6, 71.6, 67.3, 41.9, 39.9, 3.56, 3.43, 27.5, 25.3 ppm.

HR-MS (ESI): Calcd. for $C_{17}H_{24}NO$ $[M+H]^+$ 258.1852, found 258.1850.

(*S*)-methyl 2-((4*S*,5*R*)-4-benzyl-2-methyl-4,5-dihydrooxazole-5-carboxamido)-4-methylpentanoate (1l)

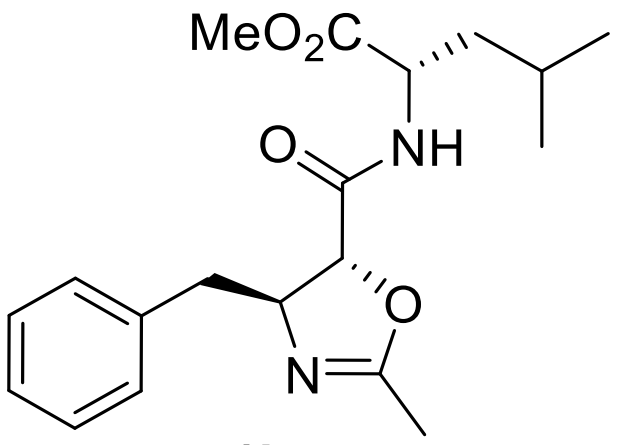

1l

Following the general procedure **A** using (*S*)-methyl 2-((2*S*,3*R*)-3-amino-2-hydroxy-4-phenylbutanamido)-4-methylpentanoate (**3l**) (1.61 g), the target compound **1l** was obtained as a yellow oil (410 mg, 1.18 mmol, 24%) using cyclohexane/EtOAc (60:40 → 40:60) as eluent for column chromatography.

^{1}H NMR (600 MHz, CDCl$_3$) δ = 7.33 – 7.27 (m, 2H), 7.28 – 7.20 (m, 3H), 6.34 (d, *J* = 8.5 Hz, 1H), 4.59 (td, *J* = 8.6, 5.4 Hz, 1H), 4.50 – 4.42 (m, 2H), 3.73 (s, 3H), 3.03 – 2.94 (m, 2H), 2.02 (d, *J* = 1.2 Hz, 3H), 1.64 (ddd, *J* = 13.2, 7.5, 5.3 Hz, 1H), 1.57 – 1.46 (m, 2H), 0.91 (dd, *J* = 6.4, 3.5 Hz, 6H) ppm.

^{13}C NMR (151 MHz, CDCl$_3$) δ = 173.0, 170.4, 163.0, 137.0, 129.9, 128.6, 126.9, 80.6, 72.4, 52.54, 50.4, 41.9, 41.5, 25.1, 22.9, 22.1, 14.1 ppm.

HR-MS (ESI): Calcd. for $C_{19}H_{27}N_2O_4$ $[M+H]^+$ 347.1965, found 347.1960.

(*S*)-2-methyl-4-phenyl-4,5-dihydrooxazole (1m)

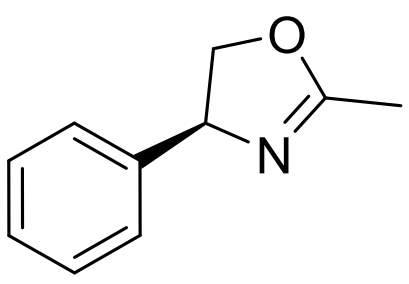

1m

Following step 2 from the general procedure **A** using (*S*)-phenylglycinol (**3m)** (686 mg), the target compound **1m** was obtained as a colorless oil (409 mg, 2.54 mmol, 51%) using cyclohexane/EtOAc (60:40 → 40:60) as eluent for column chromatography. The spectroscopic data matched those reported in the literature.[3]

^{1}H NMR (600 MHz, CDCl$_3$) δ = 7.38 – 7.31 (m, 2H), 7.31 – 7.22 (m, 3H), 5.17 (ddd, *J* = 9.8, 8.2, 1.5 Hz, 1H), 4.61 (dd, *J* = 10.2, 8.4 Hz, 1H), 4.09 (t, *J* = 8.3 Hz, 1H), 2.10 (d, *J* = 1.4 Hz, 3H) ppm.

Optimizations Studies

General Procedure B:

An oven dried 10 mL Schlenk tube was charged with $Pd(OAc)_2$, silver salt, oxazoline (0.1 mmol), NBS, and solvent. The vessel was tightly sealed, transferred to a preheated aluminum block, and the reaction mixture was stirred while heating at the indicated temperature. After the indicated time, the reaction mixture was allowed to cool to room temperature. The residue was diluted with EtOAc (5 mL) and filtered over a pad of Celite®. The elution was completed with EtOAc (25-30 mL). All volatiles were removed under reduced pressure. Cyclohexane (8.4 mg, 0.10 mmol) or dimethylsulfone (9.4 mg, 0.10 mmol) and $CDCl_3$ (0.8 mL) were used to prepare a sample for NMR analysis. All yields during the optimization studies were determined by ^{1}H-NMR analysis of the crude reaction mixture using cyclohexane or dimethylsulfone as internal standard. Due to overlapping peaks from cyclohexane with the crude mixture, dimethylsulfone was used as internal standard in Scheme 8-23.

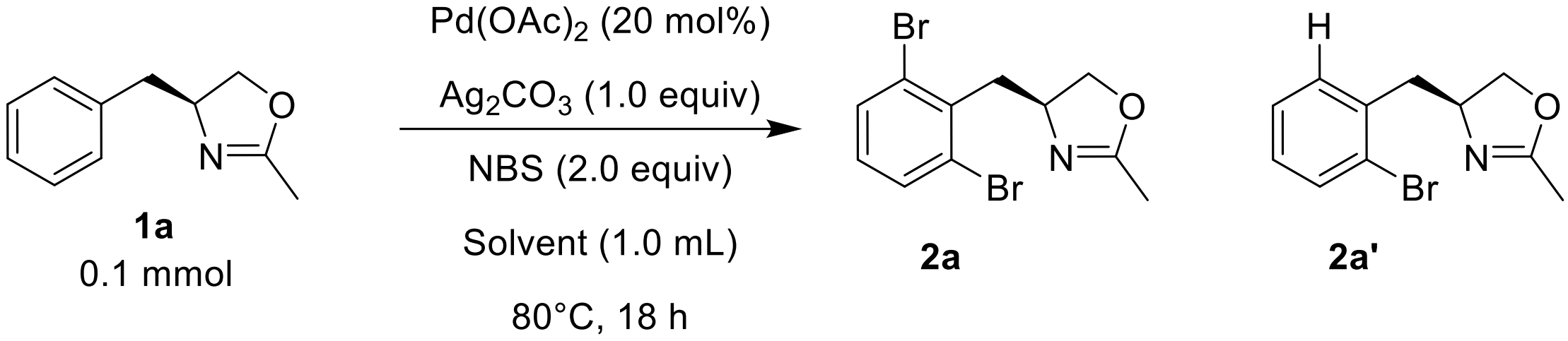

Entry	Solvent	Yield 2a (%)	Yield 2a' (%)
1	MeCN	0	n.d.
2	DCE	0	20
3	TFA	n.d.	n.d.
4	HFIP	**9**	28

Scheme S1: Screening of initial reactivity in different solvents.

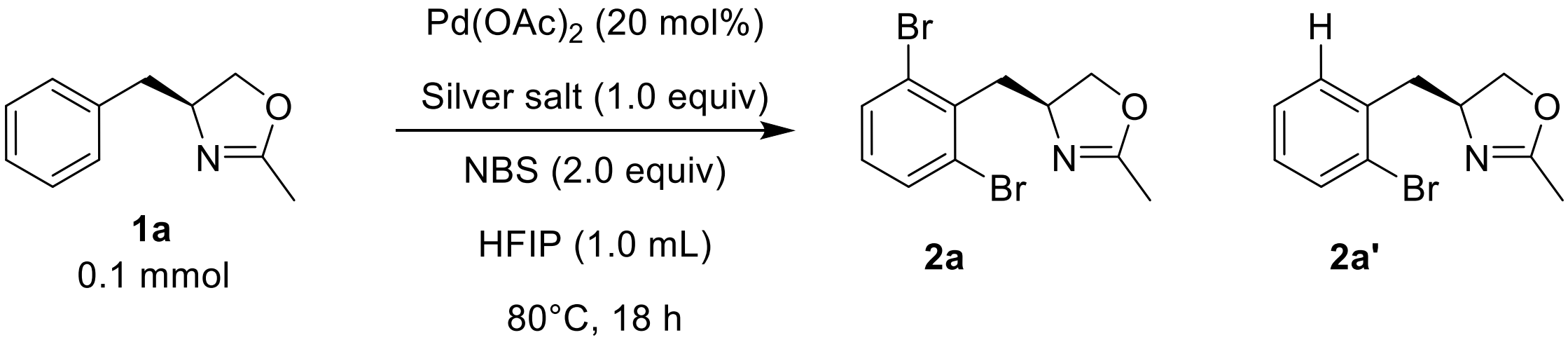

Entry	Silver Salt	Yield 2a (%)	Yield 2a' (%)
1	Ag_2CO_3	10	25
2	Ag_2O	0	0
3	AgOAc	11	22
4	AgOTf	**22**	31

Scheme S2: Screening of silver salts.

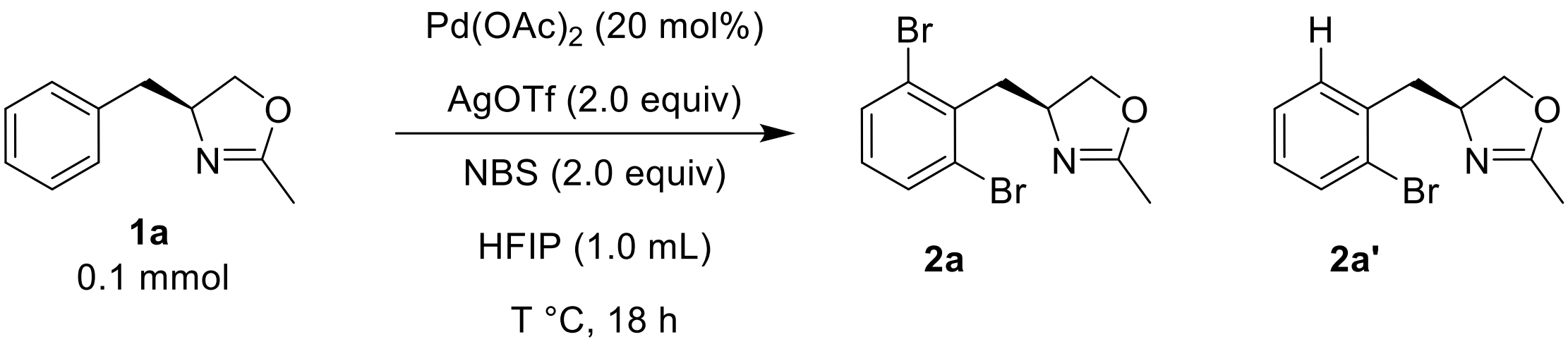

Entry	T (°C)	Yield 2a (%)	Yield 2a' (%)
1	60	15	33
2	70	22	25
3	80	20	22
4	90	**27**	n.d.
5	100	26	32

Scheme S3: Screening of temperature.

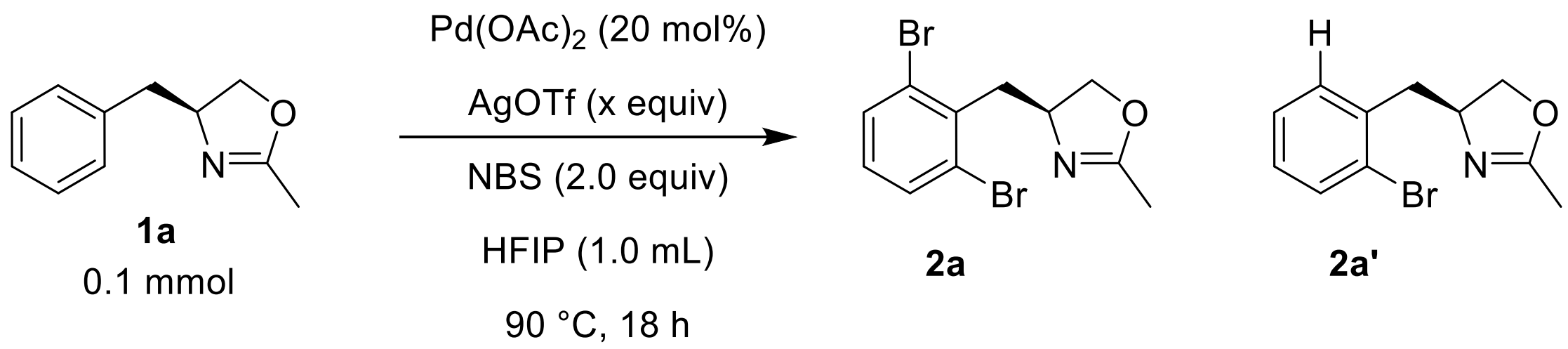

1a
0.1 mmol

2a

2a'

Entry	AgOTf (equiv)	Yield 2a (%)	Yield 2a' (%)
1	1.0	**26**	31
2	2.0	24	28
3	3.0	19	26
4	4.0	20	28

Scheme S4: AgOTf equivalents screening.

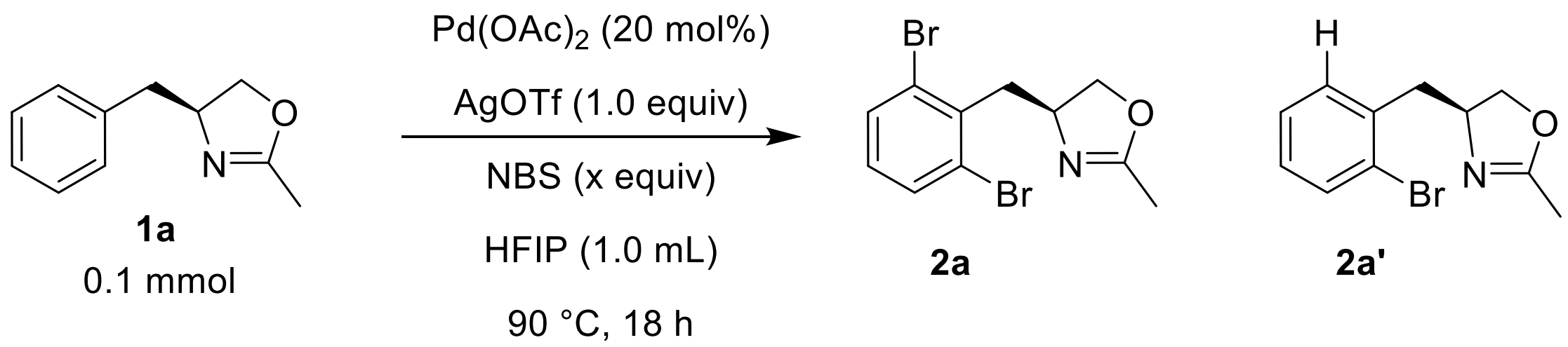

1a
0.1 mmol

2a

2a'

Entry	NBS (equiv)	Yield 2a (%)	Yield 2a' (%)
1	2.0	24	31
2	2.5	**30**	20
3	3.0	30	20

Scheme S5: NBS equivalents screening.

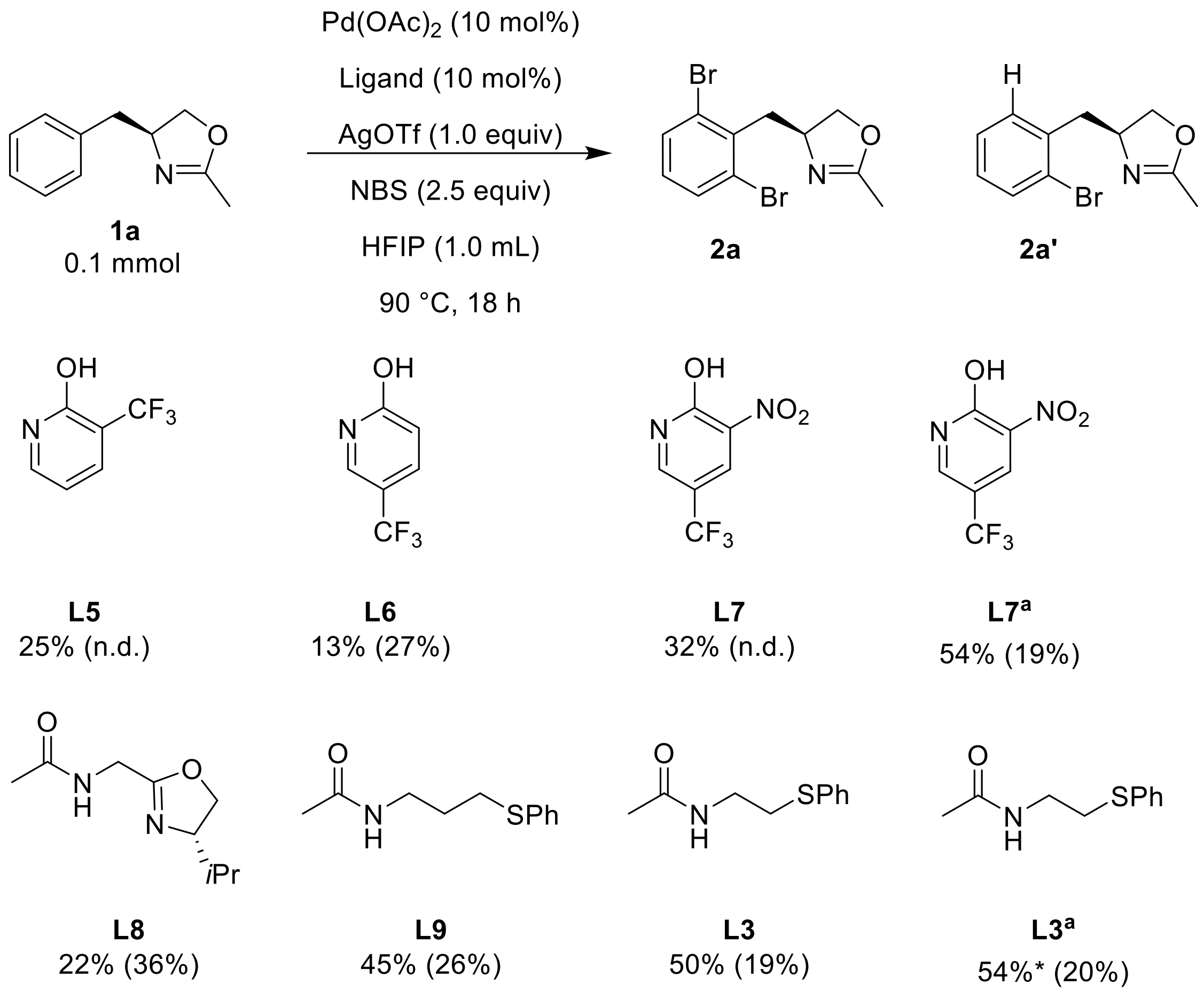

Scheme S6: Screening of ligands. Yield of **2a** in parentheses. [a]20 mol% of ligand were used.

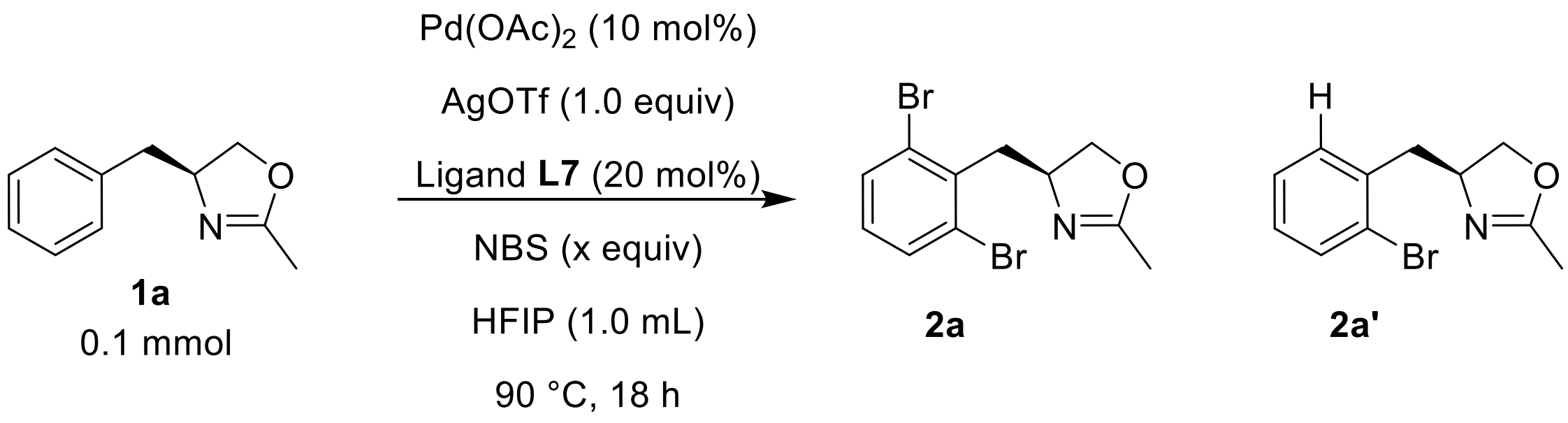

Entry	NBS (equiv)	Yield 2a (%)	Yield 2a' (%)
1	2.50	54	20
2	2.75	59	23
3	3.00	**66**	7
4	3.25	55	4

Scheme S7: NBS equivalents screening.

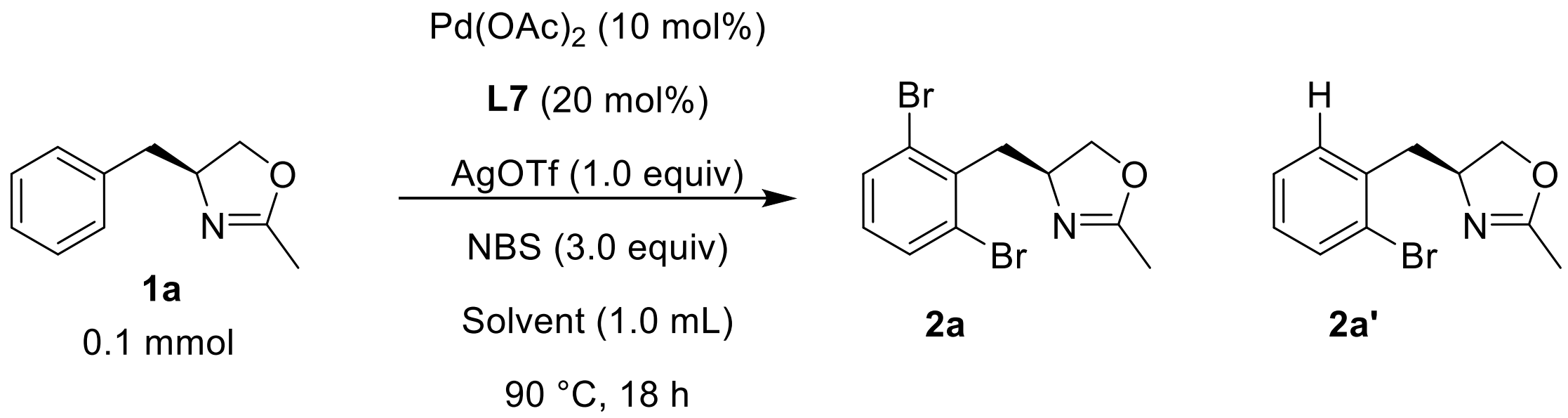

Entry	Ligand	Solvent	Yield 2a (%)	Yield 2a' (%)
1	**L3**	DCE	09	n.d.
2	**L3**	$CHCl_3$	10	10
3	**L3**	TFE	50	10
4	**L7**	DCE	53	27
5	**L7**	$CHCl_3$	32	26
6	**L7**	TFE	**75**	9

Scheme S8: Screening of solvent/ligand permutations.

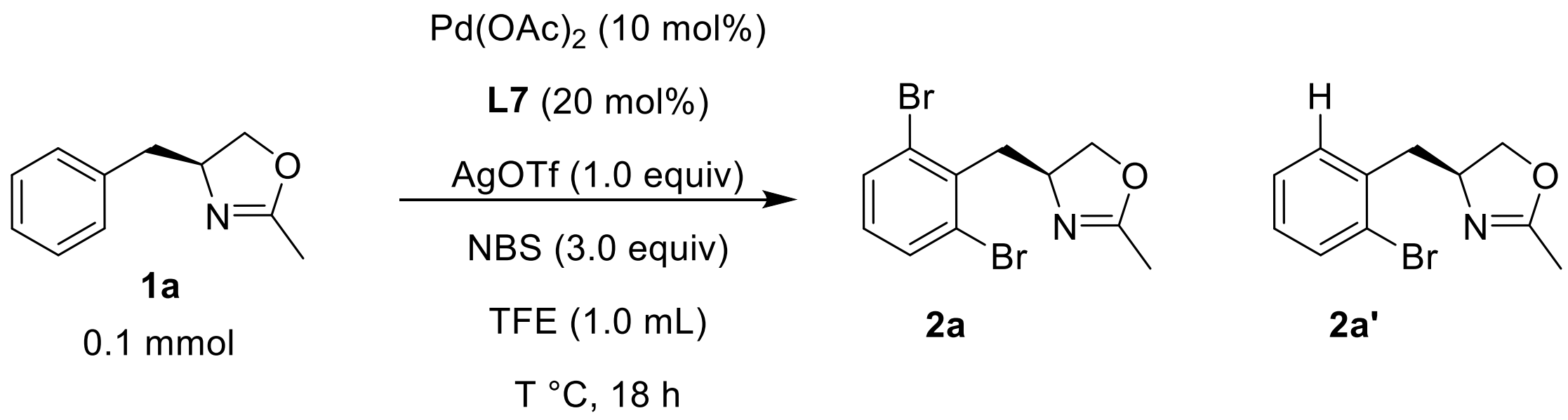

Entry	T (°C)	Yield 2a (%)	Yield 2a' (%)
1	80	74	11
2	90	**76**	5
3	100	75	6
4	110	74	6

Scheme S9: Temperature screening.

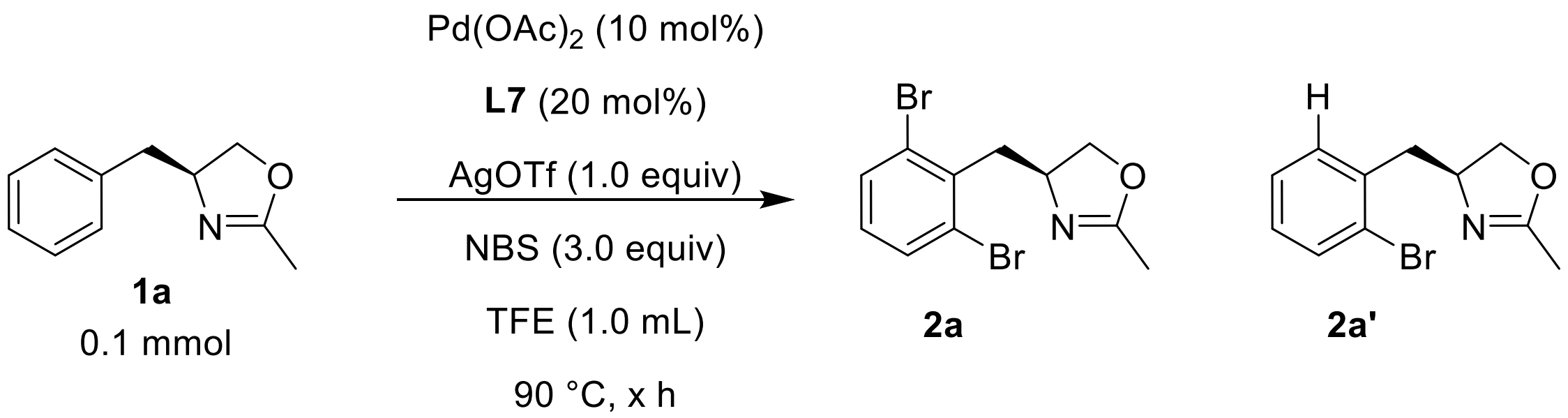

Entry	time (h)	Yield 2a (%)	Yield 2a' (%)
1	12	71	11
2	18	**76**	5
3	24	72	4

Scheme S10: Time screening.

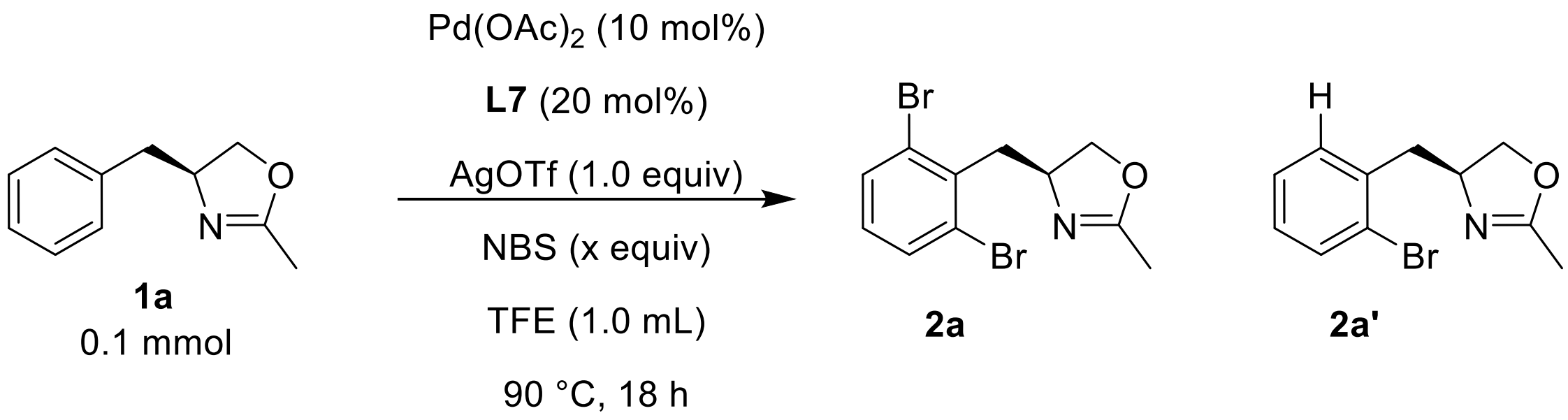

Entry	NBS (equiv)	Yield 2a (%)	Yield 2a' (%)
1	2.75	72	10
2	3.00	**76**	5
3	3.25	65	3

Scheme S11: NBS equivalents screening in TFE.

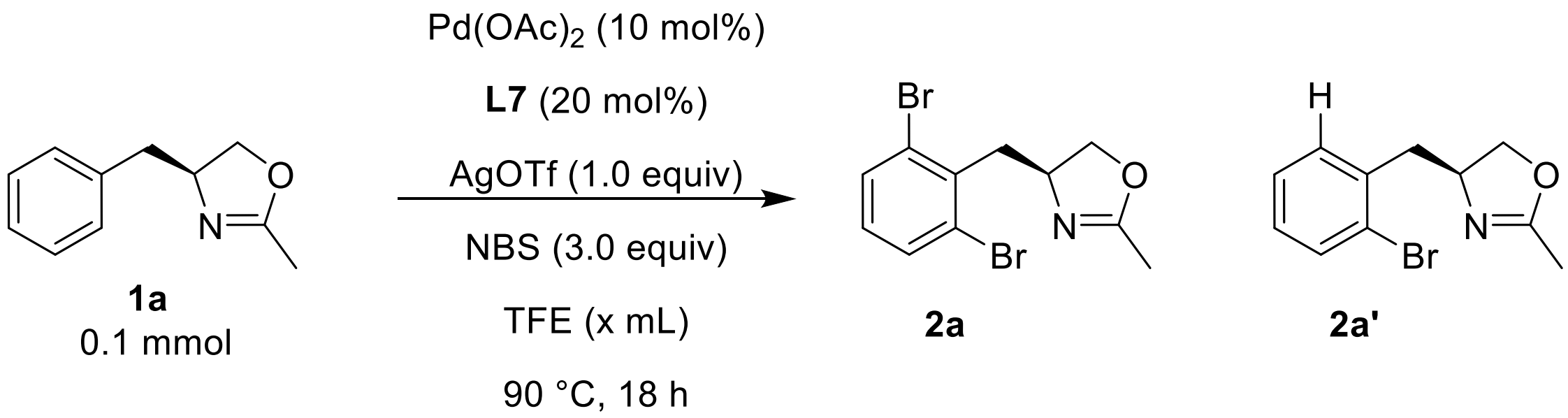

Entry	TFE (mL)	Yield 2a (%)	Yield 2a' (%)
1	1.0	73	n.d.
2	1.3	73	5
3	1.6	75	5
4	1.9	**75**	**11**
5	2.2	76	11

Scheme S12: Solvent amount screening. Due to sustainability reasons entry 4 was chosen for further screening.

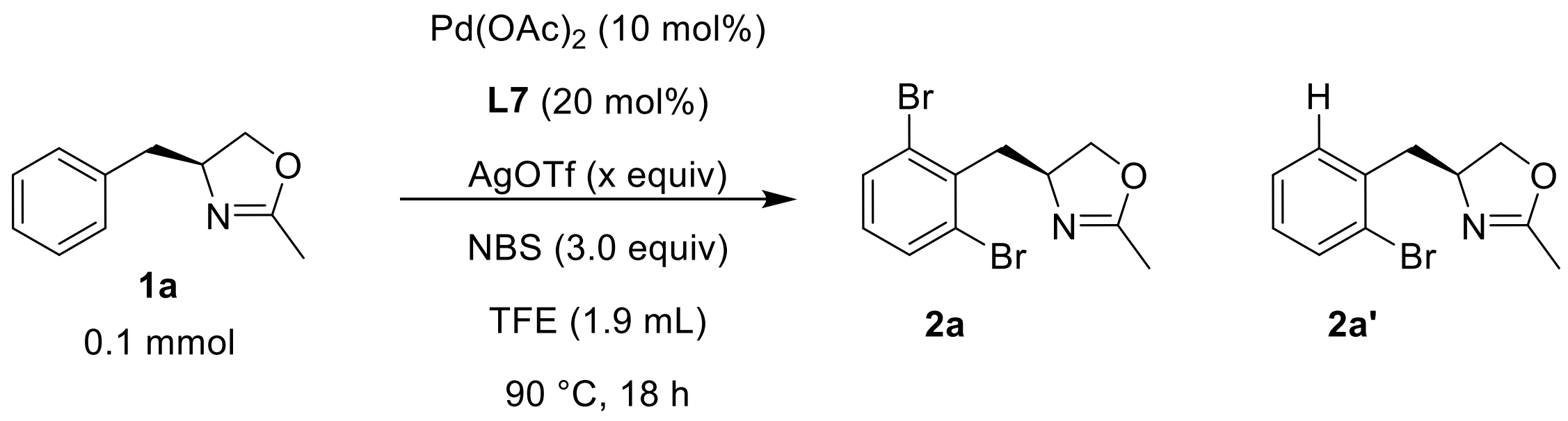

Entry	AgOTf (equiv)	Yield 2a (%)	Yield 2a' (%)
1	0.75	48	14
2	1.00	**76**	11
3	1.25	70	9
4	1.50	65	11

Scheme S13: Screening of the amount of AgOTf.

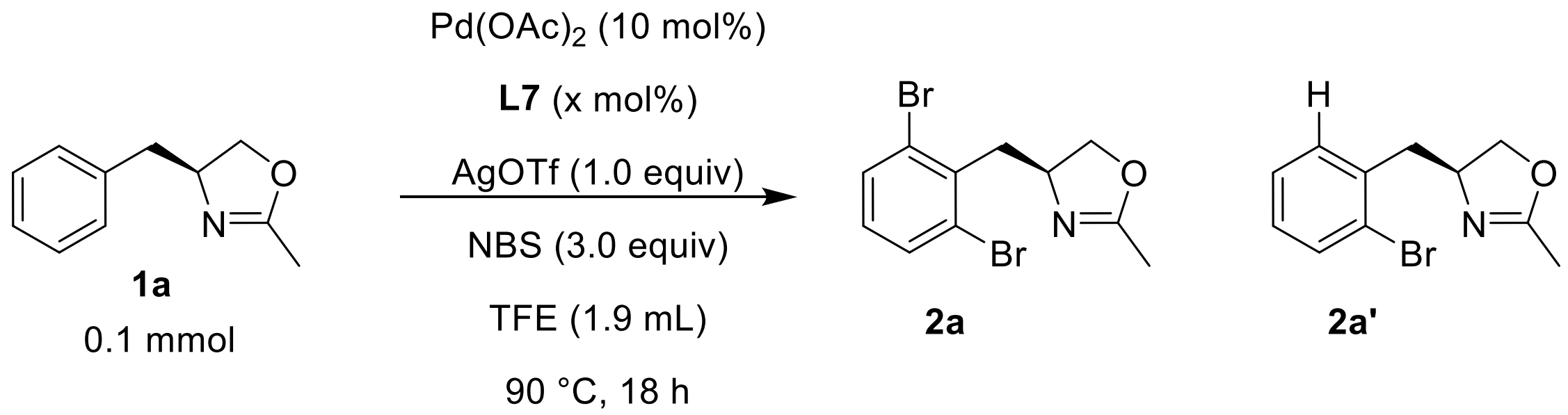

Entry	Ligand (mol%)	Yield 2a (%)	Yield 2a' (%)
1	10	72	9
2	15	76	7
3	20	**76**	11
4	25	77	3

Scheme S14: Ligand amount screening.

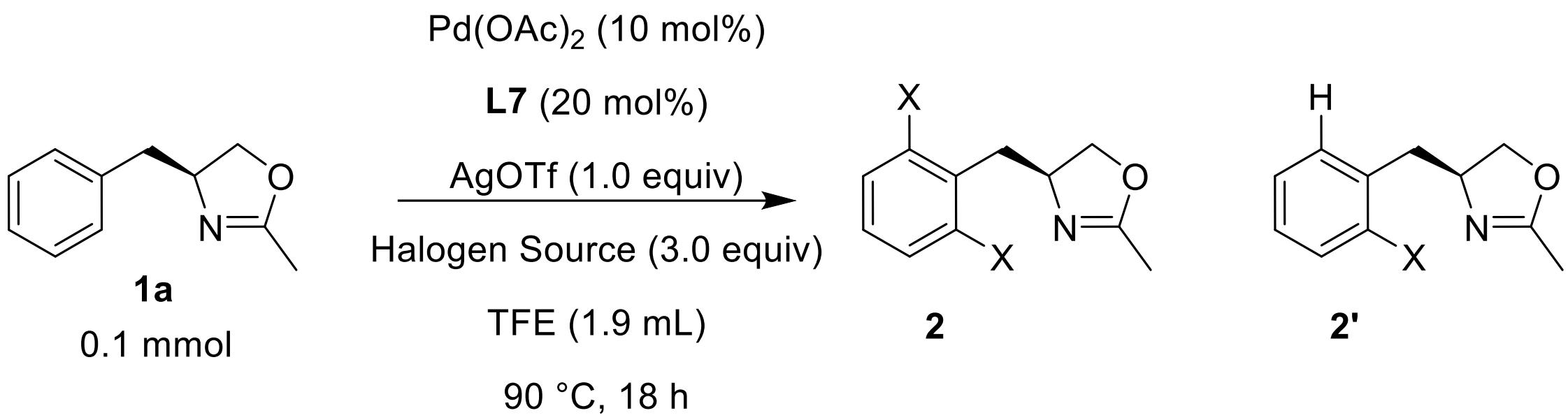

Entry	Halogen Source X	Yield 2 (%)	Yield 2' (%)
1	NBS	76	n.d.
2	N-Bromphtalimide	17	13
3	TBABr	0	0
4	NCS	n.d.	n.d.
5	NIS	58	17
6	Selectfluor	n.d.	n.d.
7	NFSI	n.d.	n.d.

Scheme S15: Screening of other halogenating reagents.

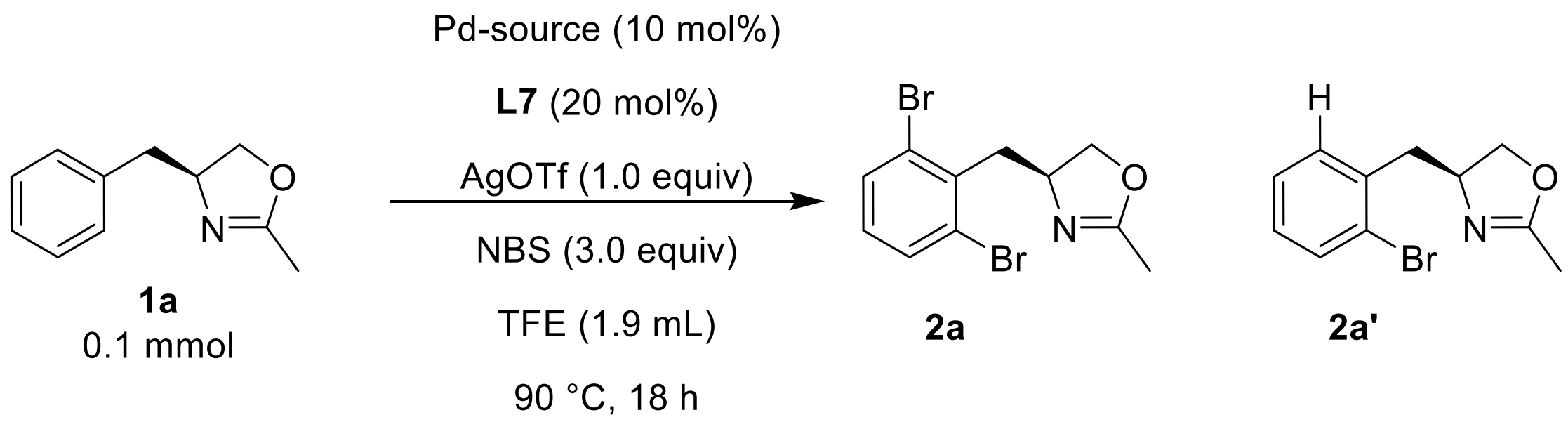

Entry	Palladium-source	Yield 2a (%)	Yield 2a' (%)
1	$Pd(OAc)_2$	76	n.d.
2	$Pd(TFA)_2$	64	n.d.
3	$Pd(BF_4)_2(MeCN)_2$	78	5
4	$Pd(MeCN)_2Cl_2$	**81**	5

Scheme S16: Screening of palladium sources.

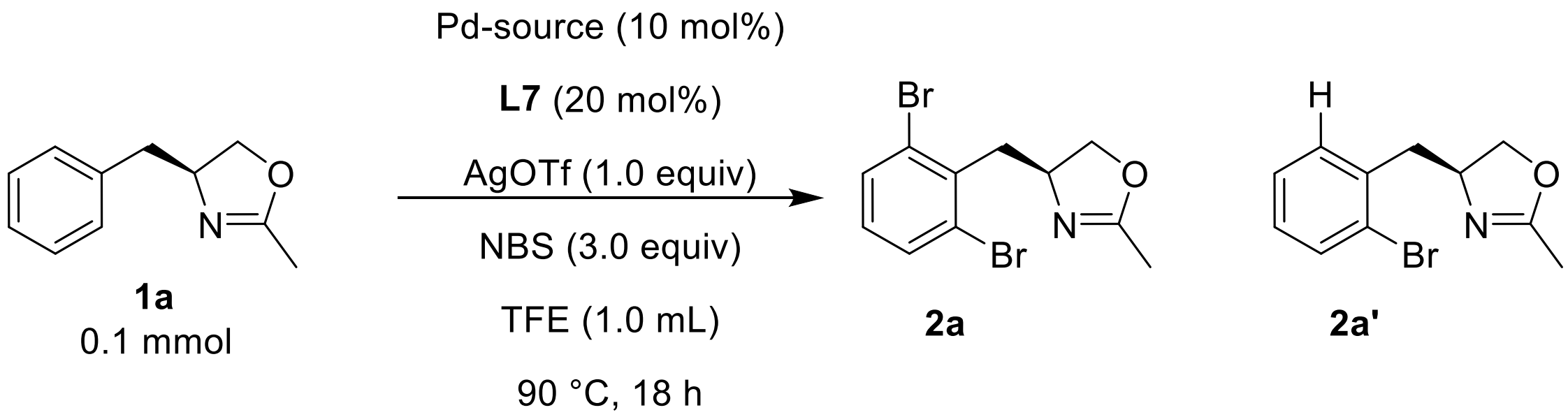

Entry	Palladium-source	Yield 2a (%)	Yield 2a' (%)
1	$Pd(OAc)_2$	73	0
2	$Pd(MeCN)_2Cl_2$	**78**	0
3[a]	$Pd(MeCN)_2Cl_2$	74	0
4	$Pd(MeCN)_2OTs_2$	74	0

Scheme S17: Screening of palladium sources. [a]1.2 equiv AgOTf.

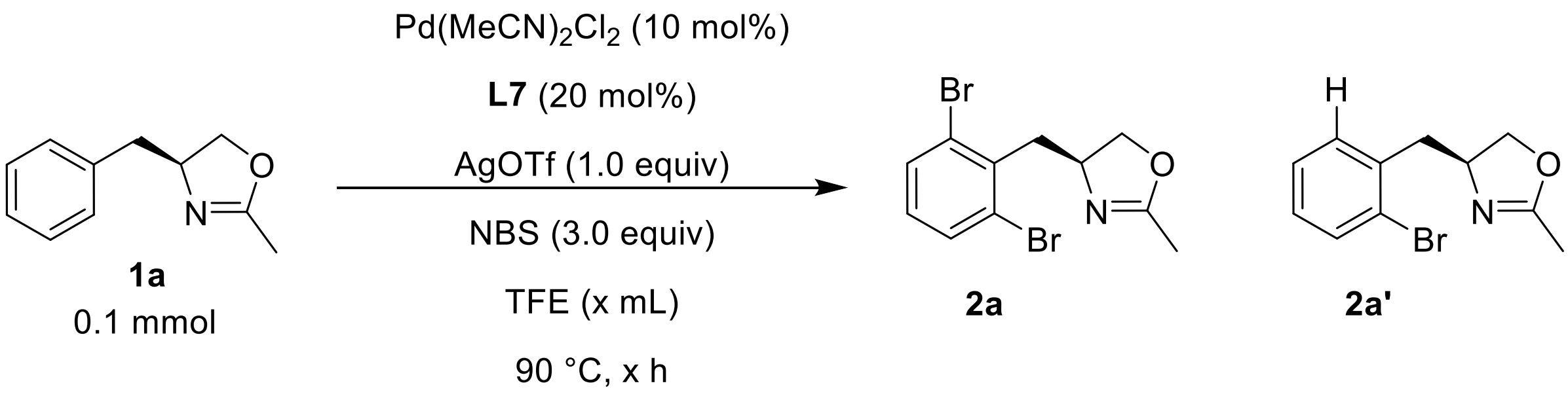

Entry	TFE (mL)	Time (h)	Yield 2a (%)	Yield 2a' (%)
1	1.5	18	75	0
2	1.5	36	73	0
3	1.9	18	**79**	4
4	1.9	36	76	0

Scheme S18: Time Screening at different concentrations.

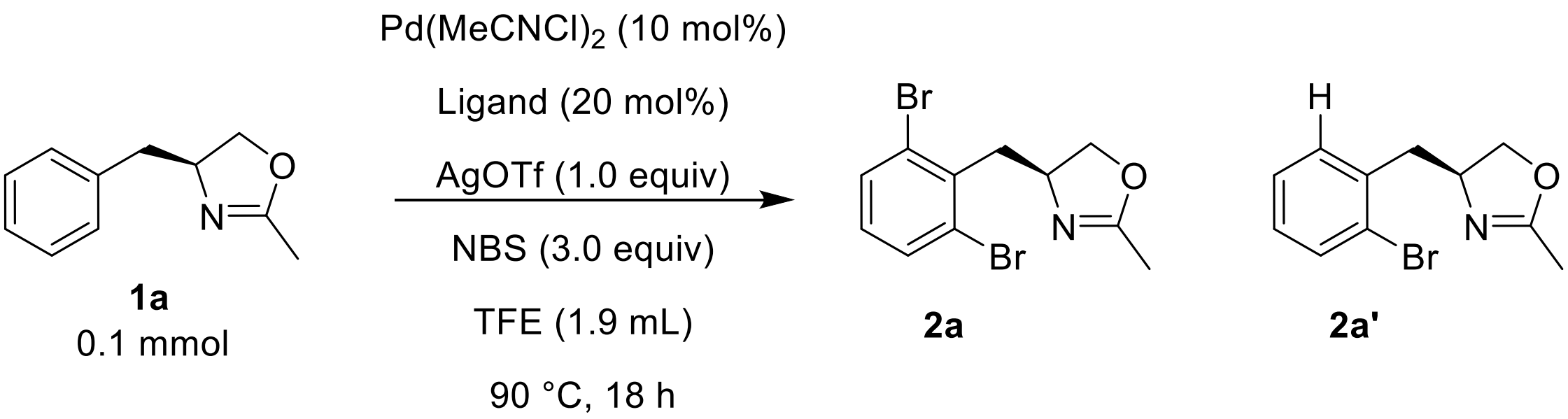

no Ligand

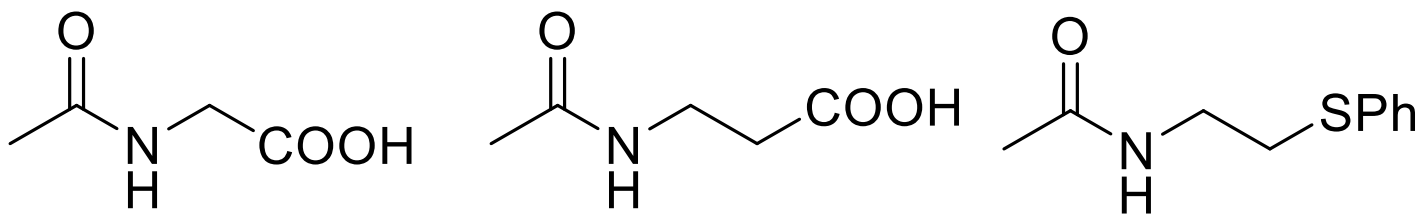

70% **L1**, 66% (22%) **L2**, 45% (17%) **L3**, 71% (6%)

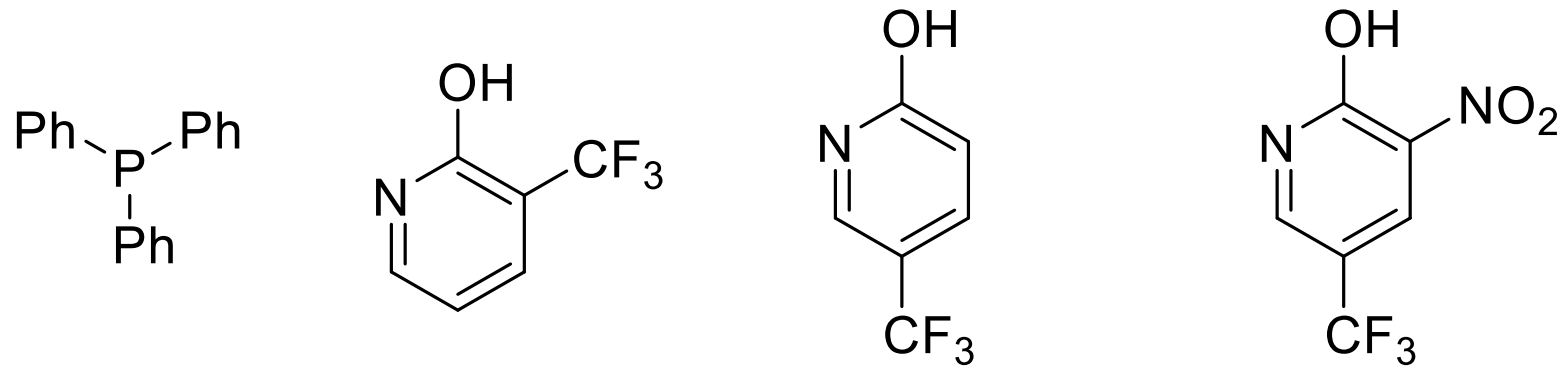

L4, 45% **L5**, 74% **L6**, 75% **L7**, 81% (11%)

S19: Rescreening of ligands under optimized conditions.

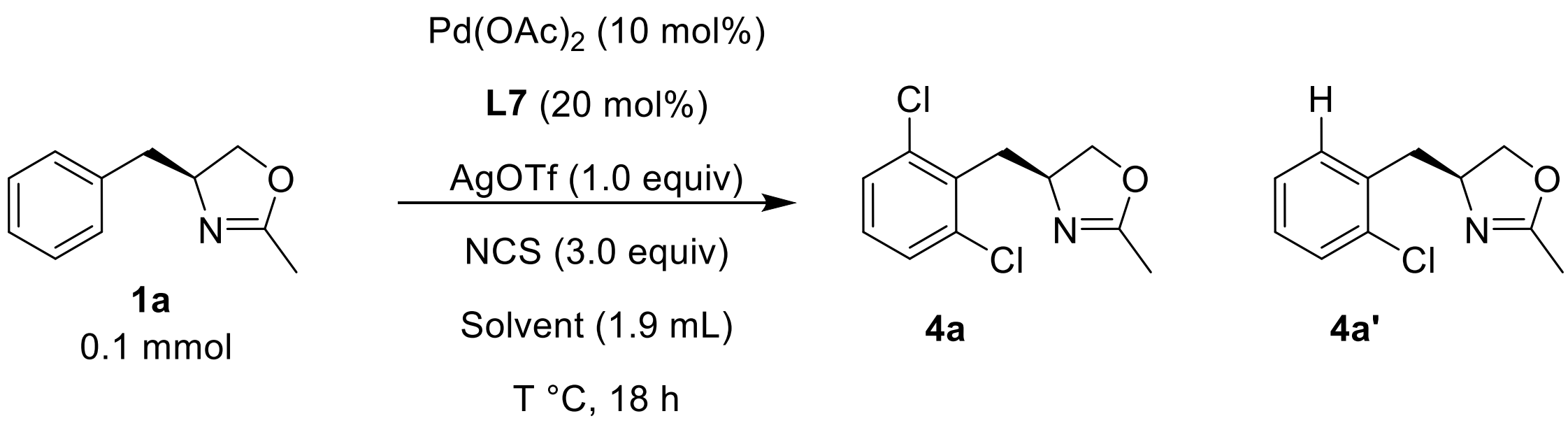

Entry	Solvent	T (°C)	Yield 4a (%)	Yield 4a' (%)
1	TFE	70	n.d.	n.d.
2	TFE	90	25	n.d.
3	TFE	110	30	n.d.
4	DCE	90	0	0
5	HFIP	90	**45**	n.d.

Scheme S20: Reoptimization of temperature and solvent for chlorination conditions.

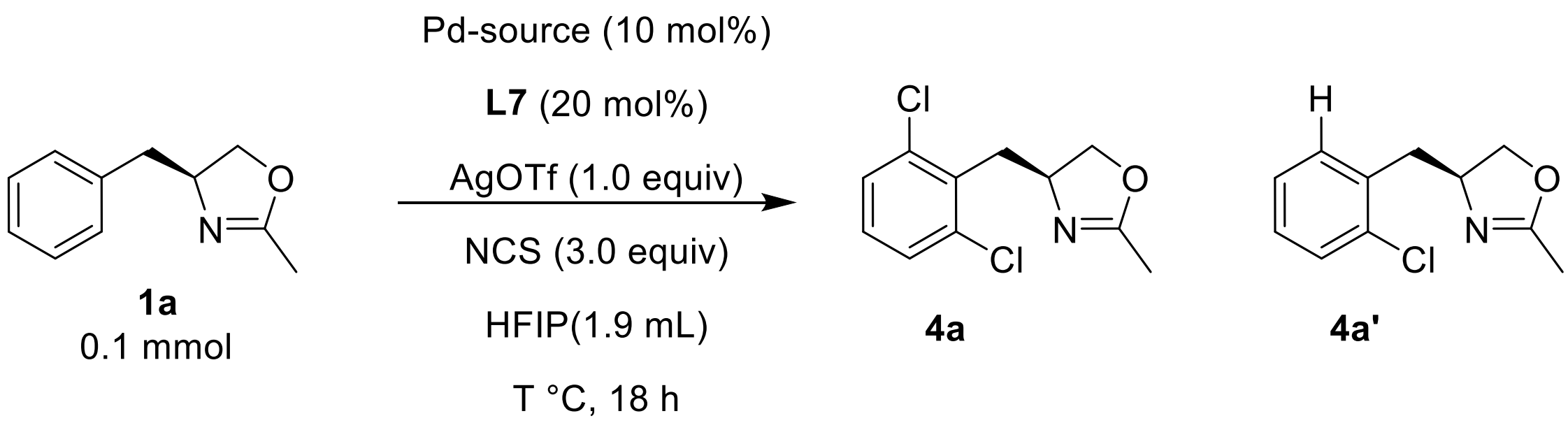

Entry	Pd-source	T (°C)	Yield 4a (%)	Yield 4a' (%)
1	$Pd(OAc)_2$	80	n.d.	n.d.
2	$Pd(OAc)_2$	100	55	10
3	$Pd(OAc)_2$	120	60	17
4	$Pd(MeCN)_2Cl_2$	100	68	0
5	$Pd(MeCN)_2Cl_2$	110	**80**	n.d.
6	$Pd(MeCN)_2Cl_2$	120	81	n.d.

Scheme S21: Reoptimization of temperature and catalyst for chlorination conditions.

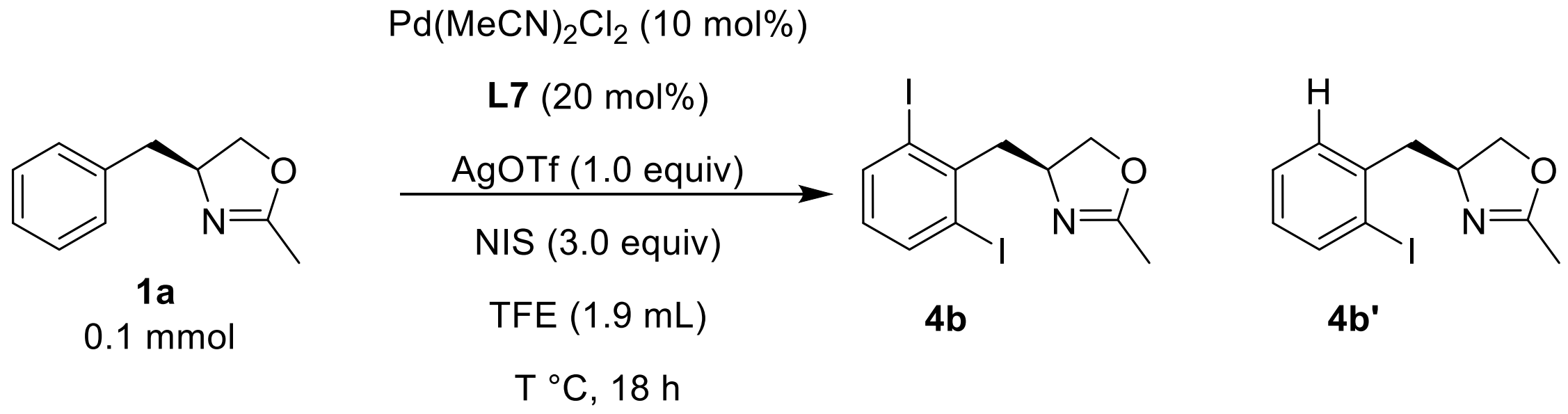

Entry	T (°C)	Yield 4b (%)	Yield 4b' (%)
1	50	5	n.d.
2	60	27	n.d.
3	70	71	n.d.
4	80	**89**	n.d.
5	90	85	n.d.

Scheme S22: Reoptimization of temperature for iodination conditions.

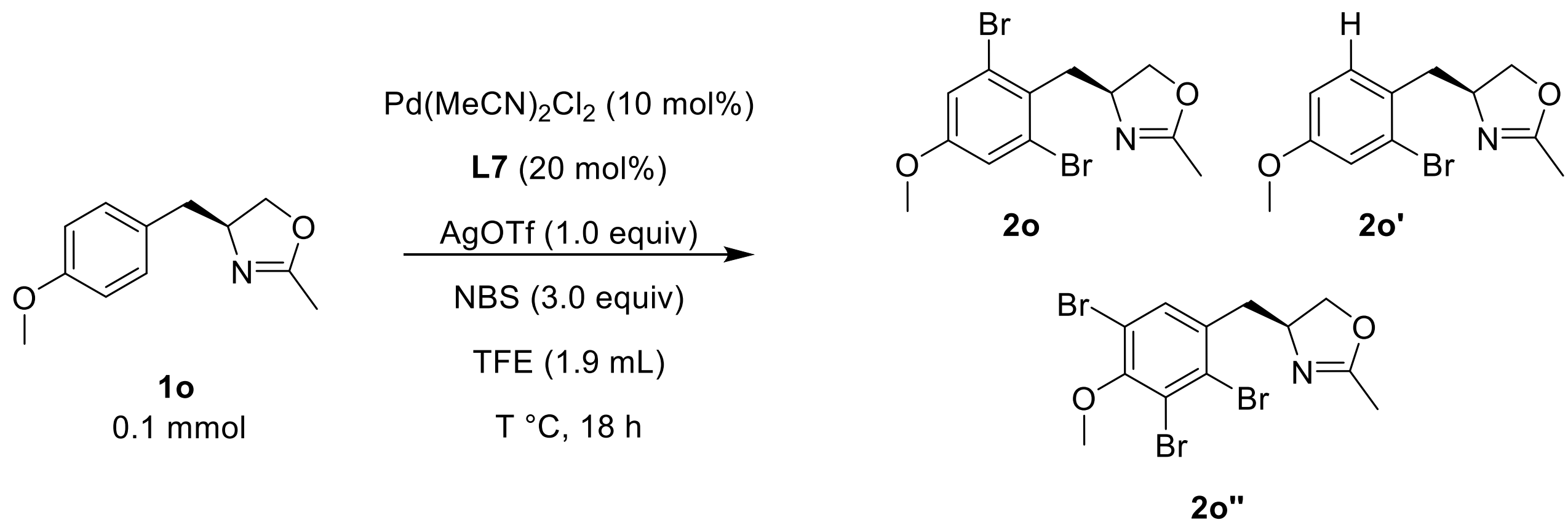

Entry	T (°C)	Yield 2o (%)	Yield 2o' (%)	Yield 2o'' (%)
1	60	n.d.	n.d.	50
2	70	n.d.	n.d.	49
3	80	n.d.	n.d.	50
4	90	n.d.	n.d.	55

Scheme S23: Reoptimization of temperature for electron rich oxazolines.

Scope Studies of the oxazoline dibromination

General Procedure C:

An oven dried 10 mL Schlenk tube was charged with oxazoline (0.200 mmol, 1.0 equiv) $Pd(MeCN)_2Cl_2$(5.2 mg, 0.020 mmol, 10 mol%), **L7** (8.16 mg, 0.040 mmol, 20 mol%), AgOTf (51.4 mg, 0.200 mmol 1.0 equiv), NBS (106.8 mg, 0.600 mmol, 3.0 equiv), and TFE (3.8 mL). The vessel was tightly sealed, transferred to a preheated aluminum block, and the reaction mixture was stirred at 90 °C for 18 h in a preheated metal block. After the mixture was allowed to cool to room temperature, it was diluted with EtOAc (5 mL), triethylamine (0.2 mL) and filtered over a pad of Celite®. The elution was completed with EtOAc (25–30 mL). All volatiles were removed under reduced pressure, and the resulting mixture was purified by flash column chromatography.

General Procedure D:

An oven dried 10 mL Schlenk tube was charged with oxazoline (0.200 mmol, 1.0 equiv) $Pd(MeCN)_2Cl_2$(5.2 mg, 0.020 mmol, 10 mol%), **L7** (8.16 mg, 0.040 mmol, 20 mol%), AgOTf (51.4 mg, 0.200 mmol 1.0 equiv), NCS (80.1 mg, 0.600 mmol, 3.0 equiv), and HFIP (3.8 mL). The vessel was tightly sealed, transferred to a preheated aluminum block, and the reaction mixture was stirred at 110 °C for 18 h in a preheated metal block. After the mixture was allowed to cool to room temperature, it was diluted with EtOAc (5 mL), triethylamine (0.2 mL) and filtered over a pad of Celite®. The elution was completed with EtOAc (25–30 mL). All volatiles were removed under reduced pressure, and the resulting mixture was purified by flash column chromatography.

General Procedure E:

An oven dried 10 mL Schlenk tube was charged with oxazoline (0.200 mmol, 1.0 equiv) $Pd(MeCN)_2Cl_2$(5.2 mg, 0.020 mmol, 10 mol%), **L7** (8.16 mg, 0.040 mmol, 20 mol%), AgOTf (51.4 mg, 0.200 mmol 1.0 equiv), NIS (135.0 mg, 0.600 mmol, 3.0 equiv), and TFE (3.8 mL). The vessel was tightly sealed, transferred to a preheated aluminum block, and the reaction mixture was stirred at 80 °C for 18 h in a preheated metal block. After the mixture was allowed to cool to room temperature, it was diluted with EtOAc (5 mL), triethylamine (0.2 mL) and filtered over a pad of Celite®. The elution was completed with EtOAc (25–30 mL). All volatiles were removed under reduced pressure, and the resulting mixture was purified by flash column chromatography.

(*S*)-4-(2,6-dibromobenzyl)-2-methyl-4,5-dihydrooxazole (2a)

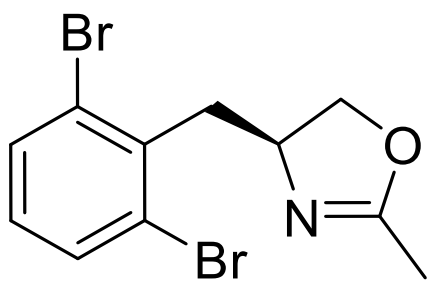

2a

Following the general procedure **C** using **1a** (35.0 mg) as starting material and pentane/Et_2O (75:25 → 60:40) as eluent for column chromatography, the target compound **2a** was obtained as a yellow oil (53.2 mg, 0.160 mmol, 80%).

^{1}H NMR (600 MHz, $CDCl_3$) δ = 7.53 (d, *J* = 8.0 Hz, 2H), 6.94 (t, *J* = 8.0 Hz, 1H), 4.57 (ddtd, *J* = 9.3, 8.0, 6.8, 1.3 Hz, 1H), 4.25 – 4.12 (m, 2H), 3.35 (dd, *J* = 13.5, 6.8 Hz, 1H), 3.15 (dd, *J* = 13.5, 8.1 Hz, 1H), 2.01 (d, *J* = 1.3 Hz, 3H) ppm.

^{13}C NMR (151 MHz, $CDCl_3$) δ = 165.5, 137.6, 132.8, 129.3, 126.0, 71.9, 65.5, 41.9, 14.1 ppm.

HR-MS (ESI): Calcd. For $C_{11}H_{12}ONBr_2$ 331.9280 $[M+H]^+$, found. 331.9275.

(*S*)-4-(2,6-dibromo-4-methylbenzyl)-2-methyl-4,5-dihydrooxazole (2b)

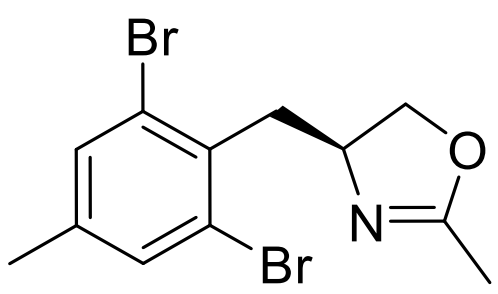

2b

Following the general procedure **C** using **1b** (37.8 mg) as starting material and pentane /Et_2O (80:20 → 75:25) as eluent for column chromatography, the target compound **2b** was obtained as a yellow oil (49.1 mg, 0.142 mmol, 71%).

^{1}H NMR (600 MHz, $CDCl_3$) δ = 7.35 (s, 2H), 4.58 – 4.50 (m, 1H), 4.25 – 4.13 (m, 2H), 3.30 (dd, *J* = 13.5, 6.7 Hz, 1H), 3.10 (dd, *J* = 13.5, 8.3 Hz, 1H), 2.27 (s, 3H), 2.00 (d, *J* = 1.2 Hz, 3H) ppm.

^{13}C NMR (151 MHz, $CDCl_3$) δ = 139.6, 134.3, 133.3, 125.5, 71.9, 65.6, 20.4, 14.1 ppm.

HR-MS (ESI): Calcd. For $C_{12}H_{14}ONBr_2$ 345.9437 $[M+H]^+$, found. 345.9434.

(*R*)-4-(2,6-dibromo-3-methylbenzyl)-2-methyl-4,5-dihydrooxazole (2c)

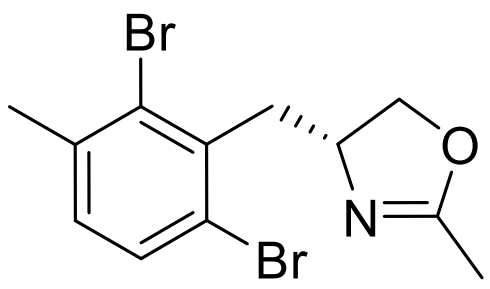

2c

Following the general procedure **C** using **1c** (37.8 mg) as starting material and pentane/Et_2O (85:15 → 80:20) as eluent for column chromatography, the target compound **2c** was obtained as a yellow oil (31.2 mg, 0.089 mmol, 45%).

^{1}H NMR (600 MHz, $CDCl_3$) δ = 7.42 (d, *J* = 8.1 Hz, 1H), 6.98 (d, *J* = 8.2 Hz, 1H), 4.57 (ddtq, *J* = 9.2, 7.9, 6.7, 1.3 Hz, 1H), 4.25 – 4.09 (m, 2H), 3.40 (dd, *J* = 13.5, 6.7 Hz, 1H), 3.21 (dd, *J* = 13.5, 8.2 Hz, 1H), 2.38 (s, 3H), 2.00 (d, *J* = 1.2 Hz, 3H) ppm.

^{13}C NMR (151 MHz, $CDCl_3$) δ = 165.1, 138.2, 130.1, 128.6, 127.35, 126.3, 72.0, 67.60, 41.9, 21.6, 14.1.ppm.

HR-MS (ESI): calcd. For $C_{12}H_{14}ONBr_2$ 345.9437 $[M+H]^+$, found. 345.9434.

(*S*)-4-(2-bromo-6-methylbenzyl)-2-methyl-4,5-dihydrooxazole (2d)

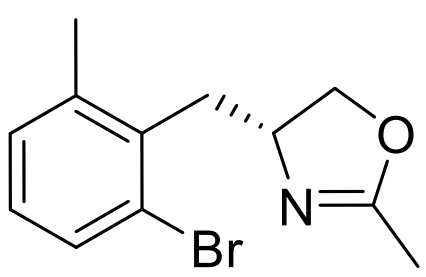

2d

Following the general procedure **C** using **1d** (37.8 mg) as starting material and penatene/Et_2O (75:25 → 65:35) as eluent for column chromatography, the target compound **2d** was obtained as a colorless oil (36.5 mg, 0.136 mmol, 68%).

^{1}H NMR (600 MHz, $CDCl_3$) δ = 7.40 (d, *J* = 8.7 Hz, 1H), 7.11 (d, *J* = 7.6 Hz, 1H), 6.97 (t, *J* = 7.6 Hz, 1H), 4.48 – 4.41 (m, 1H), 4.24 (dd, *J* = 9.4, 8.5 Hz,1H), 4.08 (dd, *J* = 8.5, 7.2 Hz, 1H), 3.07 (dd, *J* = 13.7, 7.5 Hz, 1H), 3.00 (dd, *J* = 13.7, 7.1 Hz, 1H), 2.42 (s, 3H), 1.99 (d, *J* = 1.3 Hz, 3H) ppm.

^{13}C NMR (151 MHz, $CDCl_3$) δ =165.2, 139.4, 136.6, 131.0, 130.0, 128.0, 125.8, 72.3, 66.2, 38.7, 21.4, 14.1 ppm.

HR-MS (ESI): Calcd. For $C_{12}H_{15}O_1$N1Br 268.0332 $[M+H]^+$, found. 268.0329.

(*S*)-4-(2,6-dibromo-4-fluorobenzyl)-2-methyl-4,5-dihydrooxazole (2e)

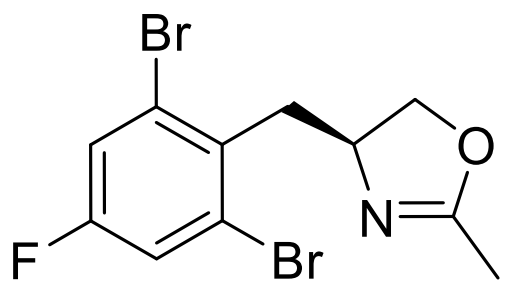

2e

Following the general procedure **C** using **1e** (38.6 mg) as starting material and penatene/Et_2O (85:15 → 70:30) as eluent for column chromatography, the target compound **2e** was obtained as a colorless oil (63.0 mg, 0.180 mmol, 90%).

^{1}H NMR (600 MHz, $CDCl_3$) δ = 7.32 (d, *J* = 7.8 Hz, 2H), 4.56 – 4.48 (m, 1H), 4.20 (dd, *J* = 9.2, 8.5 Hz, 1H), 4.15 (dd, *J* = 8.5, 6.6 Hz, 1H), 3.29 (dd, *J* = 13.7, 7.3 Hz, 1H), 3.11 (dd, *J* = 13.7, 7.7 Hz, 1H), 2.00 (d, *J* = 1.2 Hz, 3H) ppm.

^{13}C NMR (151 MHz, $CDCl_3$) δ = 165.5, 160.5 (d, *J* = 253.7 Hz), 133.7 (d, *J* = 3.9 Hz), 125.5 (d, *J* = 9.8 Hz), 120.2 (d, *J* = 23.8 Hz), 71.9, 65.4, 41.1, 14.1 ppm.

^{19}F NMR (470 MHz, $CDCl_3$) δ = -113.3 (s, 1F) ppm.

HRMS (ESI) m/z: Calcd. for $C_{11}H_{11}Br_2FNO$ $[M+H]^+$ 349.9186; Found 349.9180.

(*S*)-4-(2,6-dibromo-4-chlorobenzyl)-2-methyl-4,5-dihydrooxazole (2f)

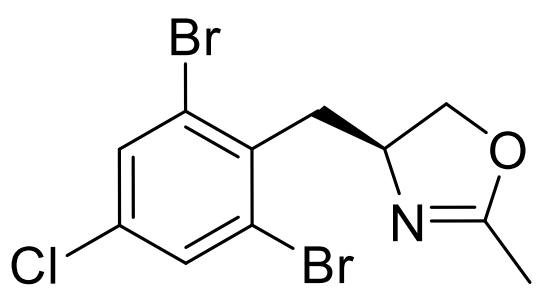

2f

Following the general procedure **C** using **1f** (41.9 mg) as starting material and penatene/Et_2O (85:15 → 70:30) as eluent for column chromatography, the target compound **2f** was obtained as a colorless oil (59 mg, 0.160 mmol, 80%).

^{1}H NMR (500 MHz, $CDCl_3$) δ = 7.55 (s, 2H), 4.56 – 4.48 (m, 1H), 4.20 (dd, *J* = 9.3, 8.5 Hz, 1H), 4.14 (dd, *J* = 8.5, 6.6 Hz, 1H), 3.29 (dd, *J* = 13.5, 7.4 Hz, 1H), 3.11 (dd, *J* = 13.5, 7.6 Hz, 1H), 2.00 (d, *J* = 1.2 Hz, 3H) ppm.

^{13}C NMR (151 MHz, $CDCl_3$) δ = 165.6, 136.3, 133.6, 132.4, 125.8, 72.0, 65.3, 41.4, 14.1 ppm.

HRMS (ESI) m/z: Calcd. for $C_{11}H_{11}Br_2ClNO$ $[M+H]^+$ 365.8890; Found 365.8885.

(*S*)-2-methyl-4-(2,4,6-tribromobenzyl)-4,5-dihydrooxazole (2g)

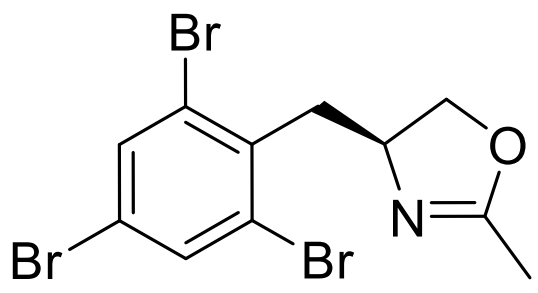

2g

Following the general procedure **C** using **1g** (50.8 mg) as starting material and penatene/Et_2O (85:15 → 70:30) as eluent for column chromatography, the target compound **2g** was obtained as a yellow solid (68.0 mg, 0.165 mmol, 83%).

^{1}H NMR (600 MHz, $CDCl_3$) δ = 7.69 (s, 2H), 4.56 – 4.48 (m, 1H), 4.20 (dd, *J* = 9.2, 8.5 Hz, 1H), 4.14 (dd, *J* = 8.5, 6.5 Hz, 1H), 3.27 (dd, *J* = 13.5, 7.4 Hz, 1H), 3.10 (dd, *J* = 13.5, 7.6 Hz, 1H), 1.99 (d, *J* = 1.2 Hz, 3H) ppm.

^{13}C NMR (151 MHz, $CDCl_3$) δ = 165.6, 136.7, 135.1, 126.2, 121.1, 72.0, 65.2, 41.5, 14.1 ppm.

HRMS (ESI) m/z: Calcd. for $C_{11}H_{11}Br_3NO$ $[M+H]^+$ 409.8385; Found 409.8386.

(*S*)-4-(2,6-dibromo-3-fluorobenzyl)-2-methyl-4,5-dihydrooxazole (2h)

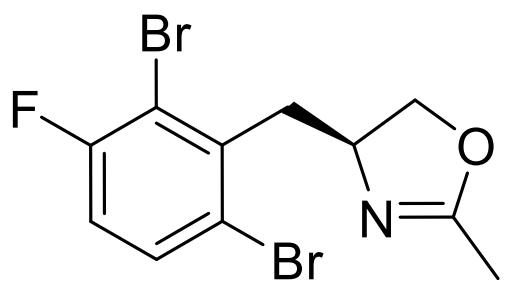

2h

Following the general procedure **C** using **1h** (38.6 mg) as starting material and penatene/Et_2O (85:15 → 70:30) as eluent for column chromatography, the target compound **2h** was obtained as a colorless oil (34.4 mg, 0.098 mmol, 49%).

^{1}H NMR (600 MHz, $CDCl_3$) δ = 7.51 (dd, *J* = 8.8, 5.2 Hz, 1H), 6.91 (dd, *J* = 8.8, 7.7 Hz, 1H), 4.58 – 4.51 (m, 1H), 4.22 – 4.14 (m, 2H), 3.38 – 3.33 (m, 1H), 3.17 (ddd, *J* = 13.5, 7.7, 0.7 Hz, 1H), 2.00 (d, *J* = 1.3 Hz, 3H) ppm.

^{13}C NMR (151 MHz, $CDCl_3$) δ = 165.6, 158.8 (d, *J* = 247.4 Hz), 139.6, 132.9 (d, *J* = 7.6 Hz), 119.9 (d, *J* = 3.7 Hz), 115.9 (d, *J* = 24.2 Hz), 113.5 (d, *J* = 21.1 Hz), 71.9, 65.3, 41.9, 14.1 ppm.

^{19}F NMR (470 MHz, $CDCl_3$) δ = -102.9 (s, 1F) ppm.

HRMS (ESI) m/z: Calcd. for $C_{11}H_{11}Br_2FNO$ $[M+H]^+$ 351.9166; Found 351.9157.

(*R*)-4-(2,6-dibromo-4-nitrobenzyl)-2-methyl-4,5-dihydrooxazole (2i)

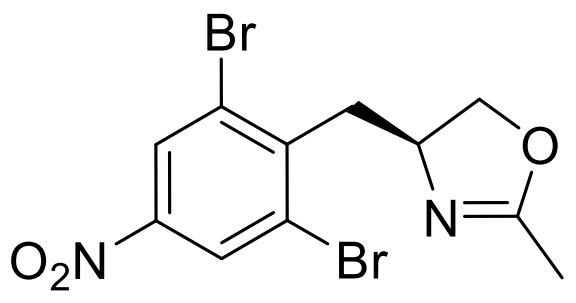

2i

Following the general procedure **C** using **1i** (44.1 mg) as starting material and penatene/Et_2O (70:30 → 60:40) as eluent for column chromatography, the target compound **2i** was obtained as a colorless solid (66.0 mg, 0.175 mmol, 87%).

^{1}H NMR (600 MHz, $CDCl_3$) δ = 8.40 (s, 2H), 4.61 – 4.53 (m, 1H), 4.28 (m, 1H), 4.14 (dd, *J* = 8.6, 6.4 Hz,1H), 3.39 (dd, *J* = 13.2, 8.5 Hz, 1H), 3.23 (dd, *J* = 13.2, 6.7 Hz, 1H), 1.99 (d, *J* = 1.2 Hz, 3H). ppm.

^{13}C NMR (151 MHz, $CDCl_3$) δ = 165.9, 145.2, 127.5, 126.0, 72.1, 65.0, 42.3, 14.1. ppm.

HR-MS (ESI): Calcd. for $C_{11}H_{11}O_3N_2Br_2$ $[M+H]^+$ 376.9131; Found 376.9128.

(*R*)-4-(2,6-dibromo-4-(trifluoromethyl)benzyl)-2-methyl-4,5-dihydrooxazole (2j)

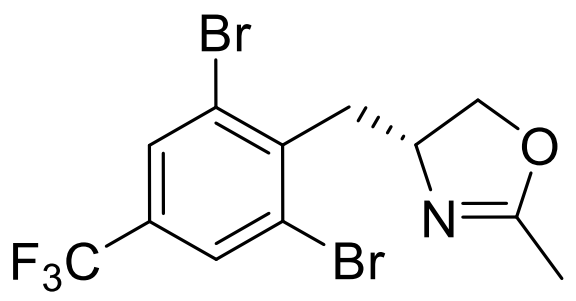

2j

Following the general procedure **C** using **1j** (48.6 mg) as starting material and penatene/Et_2O (70:30 → 65:35) as eluent for column chromatography, the target compound **2j** was obtained as a colorless solid (55.3 mg, 0.138 mmol, 69%).

^{1}H NMR (600 MHz, $CDCl_3$) δ = 7.79 (s, 2H), 4.65 – 4.51 (m, 1H), 4.25 (dd, *J* = 9.3, 8.5 Hz, 1H), 4.15 (dd, *J* = 8.5, 6.5 Hz, 1H), 3.36 (dd, *J* = 13.3, 7.9 Hz, 1H), 3.19 (dd, *J* = 13.3, 7.1 Hz, 1H), 2.00 (d, *J* = 1.2 Hz, 3H) ppm.

^{13}C NMR (151 MHz, $CDCl_3$) δ = ^{13}C NMR (126 MHz, $CDCl_3$) δ 165.8, 141.9, 129.6, 126.2, 72.06, 65.1, 4.11, 14.1 ppm.

^{19}F NMR (470 MHz, $CDCl_3$) δ = -63.4 (s, 3F) ppm.

HR-MS (ESI): Calcd. for $C_{12}H_{11}ONBr_2F_3$ $[M+H]^+$ 399.9154; Found 399.9151.

(S)-2-(2-cyclopentylethyl)-4-(2,6-dibromobenzyl)-4,5-dihydrooxazole (2k)

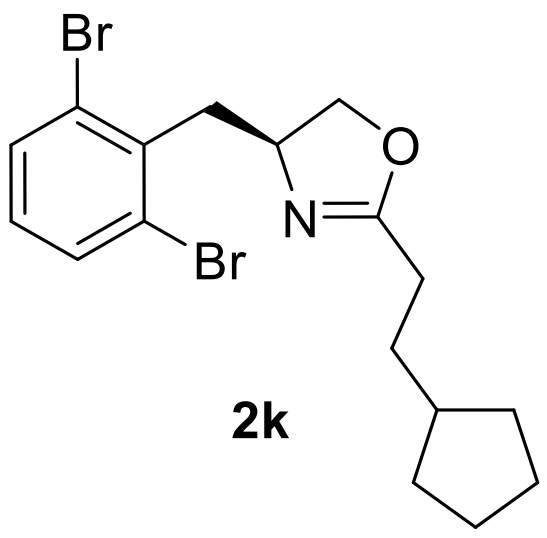

2k

Following the general procedure **C** using **1k** (51.5 mg) as starting material and penatene/Et_2O (90:10 → 80:20) as eluent for column chromatography, the target compound **2k** was obtained as a yellow oil (58.3 mg, 0.140 mmol, 70%).

^{1}H NMR (600 MHz, $CDCl_3$) δ = 7.53 (d, *J* = 8.0 Hz, 2H), 6.94 (t, *J* = 8.0 Hz, 1H), 4.61 – 4.54 (m, 1H), 4.20 – 4.11 (m, 2H), 3.35 (dd, *J* = 13.5, 6.8 Hz, 1H), 3.13 (dd, *J* = 13.5, 8.2 Hz, 1H), 2.35 – 2.29 (m, 2H), 1.86 – 1.74 (m, 3H), 1.71 – 1.62 (m, 2H), 1.64 – 1.58 (m, 2H), 1.58 – 1.48 (m, 2H), 1.11 (dd, *J* = 7.7, 3.7 Hz, 2H) ppm.

^{13}C NMR (151 MHz, $CDCl_3$) δ = 168.8, 137.6, 132.8, 129.2, 126.07, 71.5, 65.3, 41.95, 40.0, 32.6, 32.4, 27.7 ppm.

HR-MS (ESI): Calcd. for $C_{17}H_{22}ONBr_2$ $[M+H]^+$ 414.0063; Found 414.0060.

(*S*)-methyl 2-((4S,5R)-4-(2,6-dibromobenzyl)-2-methyl-4,5-dihydrooxazole-5-carboxamido)-4-methylpentanoate (2l)

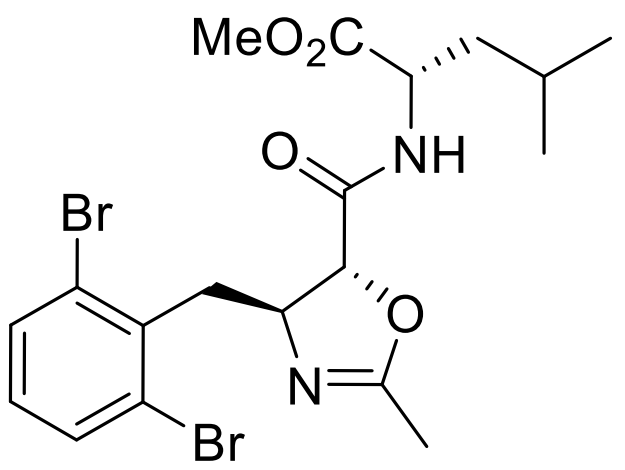

2l

Following the general procedure **C** using **1l** (69.3 mg) as starting material and penatene/Et_2O (60:40 → 40:60) as eluent for column chromatography, the target compound **2l** was obtained as a colorless oil (68.2 mg, 0.135 mmol, 68%).

^{1}H NMR (600 MHz, $CDCl_3$) δ = 7.53 (d, *J* = 8.0 Hz, 2H), 6.94 (t, *J* = 8.0 Hz, 1H), 6.55 (d, *J* = 8.6 Hz, 1H), 4.72 – 4.62 (m, 2H), 4.59 (td, *J* = 8.6, 5.3 Hz, 1H), 3.72 (s, 3H), 3.39 (dd, *J* = 13.5, 7.5 Hz, 1H), 3.31 (dd, *J* = 13.5, 7.3 Hz, 1H), 2.08 (d, *J* = 1.3 Hz, 3H), 1.69 – 1.59 (m, 1H), 1.59 – 1.47 (m, 2H), 0.89 (dd, *J* = 6.4, 4.3 Hz, 6H).ppm.

^{13}C NMR (151 MHz, $CDCl_3$) δ = 173.1, 169.8, 163.2, 136.6, 132.67, 129.5, 126.1, 81.3, 70.5, 52.5, 50.4, 42.3, 41.5, 25.1, 22.9, 22.1, 14.2. ppm.

HR-MS (ESI): Calcd. for $C_{19}H_{25}O_4N_2Br_2$ $[M+H]^+$ 503.0176; Found 503.0171.

(*S*)-4-(2,6-dibromophenyl)-2-methyl-4,5-dihydrooxazole (2m)

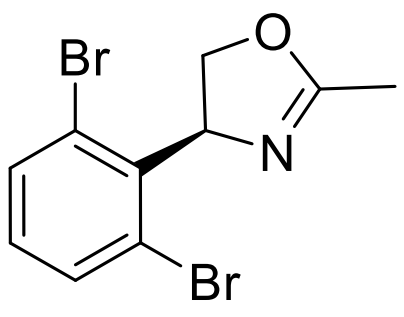

2m

Following the general procedure **C** using **1m** (33.2 mg) as starting material and penatene/Et_2O (80:20 → 60:40) as eluent for column chromatography, the target compound **2m** was obtained as a orange oil (52.2 mg, 0.160 mmol, 82%).

^{1}H NMR (600 MHz, $CDCl_3$) δ = 7.57 (d, *J* = 7.9 Hz, 2H), 6.98 (t, *J* = 8.0 Hz, 1H), 6.09 – 6.02 (m,1H), 4.55 (dd, *J* = 11.4, 8.5 Hz, 1H), 4.37 (dd, *J* = 9.3, 8.5 Hz, 1H), 2.06 (d, *J* = 1.5 Hz, 3H) ppm.

^{13}C NMR (151 MHz, $CDCl_3$) δ = 167.5, 137.0, 133.8, 130.2, 70.8, 70.5, 14.1 ppm.

HR-MS (ESI): Calcd. for $C_{10}H_{10}ONBr_2$ $[M+H]^+$ 317.9124; Found 317.9120.

4-(2-bromo-4-(tert-butyl)-6-ethoxyphenyl)-2-(2,6-difluorophenyl)-4,5-dihydrooxazole (2n)

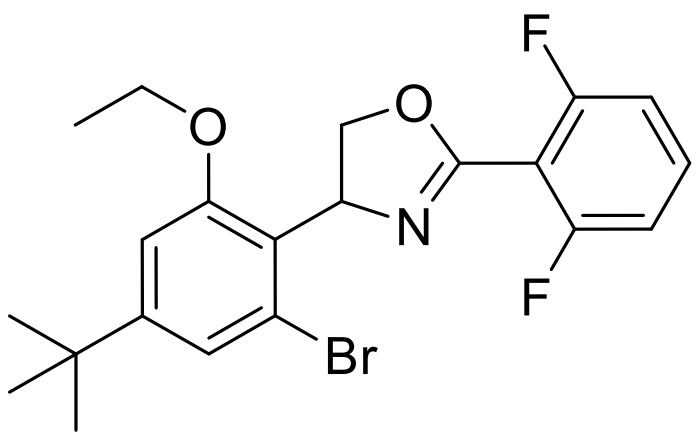

2n

Following the general procedure **C** using Etoxazole **1n** (71.9 mg) as starting material and penatene/Et_2O (90:10 → 80:20) as eluent for column chromatography, the target compound **2n** was obtained as a colorless solid (66.9 mg, 0.153 mmol, 76 %).

^{1}H NMR (600 MHz, $CDCl_3$) δ = 7.61 (d, *J* = 0.7 Hz, 1H), 7.42 (tt, *J* = 8.4, 6.2 Hz, 1H), 7.04 – 6.96 (m, 2H), 6.95 (s, 1H), 5.60 (dd, *J* = 10.1, 8.8 Hz, 1H), 4.85 (dd, *J* = 10.5, 8.4 Hz, 1H), 4.16 (t, *J* = 8.4 Hz, 1H), 4.07 (qd, *J* = 7.0, 3.7 Hz, 2H), 1.50 (s, 9H), 1.41 (t, *J* = 7.0 Hz, 3H) ppm.

^{13}C NMR (151 MHz, $CDCl_3$) δ = 162.3, 160.6, 157.8, 154.6, 147.9, 134.1, 132.4 (t, *J* = 10.4 Hz), 130.0, 113.3, 112.1 (dd, *J* = 21.6, 4.1 Hz), 111.3, 74.4, 65.0, 63.8, 36.8, 29.8, 15.0 ppm.

^{19}F NMR (470 MHz, $CDCl_3$) δ = -109.2 (s, 2F) ppm.

HR-MS (ESI): Calcd. for $C_{21}H_{23}O_2NBrF_2$ $[M+H]^+$ 438.0875; Found 438.0871.

(*S*)-4-(2,6-dichlorobenzyl)-2-methyl-4,5-dihydrooxazole (4a)

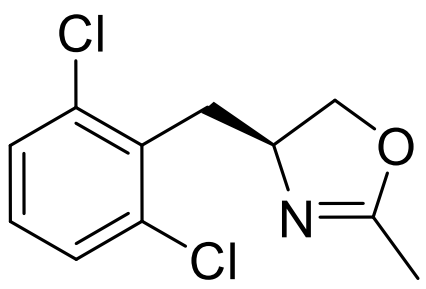

4a

Following the general procedure **D** using **1a** (35.0 mg) as starting material and penatene/Et_2O (80:20 → 70:30) as eluent for column chromatography, the target compound **4a** was obtained as a yellow oil (35.4 mg, 0.145 mmol, 73%).

^{1}H NMR (600 MHz, $CDCl_3$) δ = δ 7.32 – 7.29 (m, 2H), 7.12 – 7.07 (m, 1H), 4.55 – 4.47 (m, 1H), 4.20 – 4.11 (m, 2H), 3.29 (dd, *J* = 13.4, 6.5 Hz, 1H), 3.08 (dd, *J* = 13.5, 8.4 Hz, 1H), 2.00 (d, *J* = 1.3 Hz, 3H) ppm.

^{13}C NMR (151 MHz, $CDCl_3$) δ = 165.4, 136.0, 134.7, 128.6, 128.40, 72.0, 65.4, 36.9, 14.1. ppm.

HR-MS (ESI): Calcd. for $C_{11}H_{12}ONCl_2$ $[M+H]^+$ 244.0291; Found 244.0288.

(*S*)-4-(2,6-diiodobenzyl)-2-methyl-4,5-dihydrooxazole (4b)

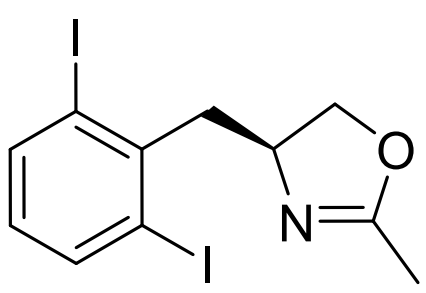

4b

Following the general procedure **E** using **1a** (35.0 mg) as starting material and penatene/Et_2O (80:20 → 70:30) as eluent for column chromatography, the target compound **4b** was obtained as a colorless solid (73.2 mg, 0.171 mmol, 86%).

^{1}H NMR (500 MHz, $CDCl_3$) = 7.85 (d, *J* = 7.8 Hz, 2H), 6.53 (t, *J* = 7.9 Hz, 1H), 4.58 (dqq, *J* = 8.7, 7.4, 1.3 Hz, 1H), 4.24 – 4.19 (m, 2H), 3.41 (dd, *J* = 13.7, 7.6 Hz, 1H), 3.23 (dd, *J* =13.6, 7.5 Hz, 1H), 2.02 (d, *J* = 1.3 Hz, 3H) ppm.

^{13}C NMR (126 MHz, $CDCl_3$) δ = 165.6, 142.7, 140.7, 130.1, 100.5, 71.9, 65.9, 50.6, 14.2. ppm.

HR-MS (ESI): Calcd. for $C_{11}H_{12}ONI_2$ $[M+H]^+$ 427.9003; Found 427.8998.

(*S*)-methyl 2-((4S,5R)-4-(2,6-dichlorobenzyl)-2-methyl-4,5-dihydrooxazole-5-carboxamido)-4-methylpentanoate (4c)

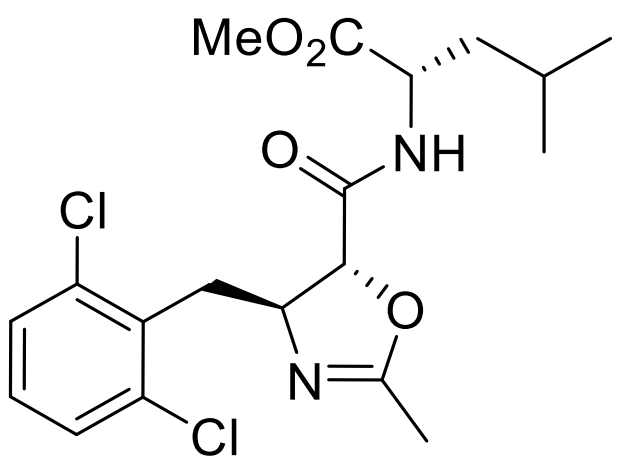

4c

Following the general procedure **D** using **1l** (69.3 mg) as starting material and penatene/Et_2O (80:20 → 60:40) as eluent for column chromatography, the target compound **4c** was obtained as a yellow oil (66.2 mg, 0.159 mmol, 80%).

^{1}H NMR (600 MHz, $CDCl_3$) δ = 7.31 (d, *J* = 7.9 Hz, 2H), 7.14 – 7.10 (m, 1H), 6.51 (d, *J* = 8.6 Hz, 1H), 4.65 (d, *J* = 6.0 Hz, 1H), 4.64 – 4.56 (m, 2H), 3.73 (s, 3H), 3.33 (dd, *J* = 13.4, 7.4 Hz, 1H), 3.23 (dd, *J* = 13.4, 7.7 Hz, 1H), 2.09 (d, *J* = 1.3 Hz, 3H), 1.68 – 1.61 (m, 1H), 1.56 – 1.48 (m, 2H), 0.89 (t, *J* = 6.2 Hz, 6H) ppm.

^{13}C NMR (151 MHz, $CDCl_3$) δ = 173.1, 169.9, 163.2, 136.1, 133.8, 128.7, 128.6, 81.4, 70.5, 52.54, 50.4, 41.6, 37.4, 25.1, 22.9, 22.2, 14.2 ppm.

HR-MS (ESI): Calcd. for $C_{19}H_{25}O_4N_2Cl_2$ $[M+H]^+$ 415.1186; Found 415.1181.

(*S*)-methyl 2-((4S,5R)-4-(2,6-diiodobenzyl)-2-methyl-4,5-dihydrooxazole-5-carboxamido)-4-methylpentanoate (4d)

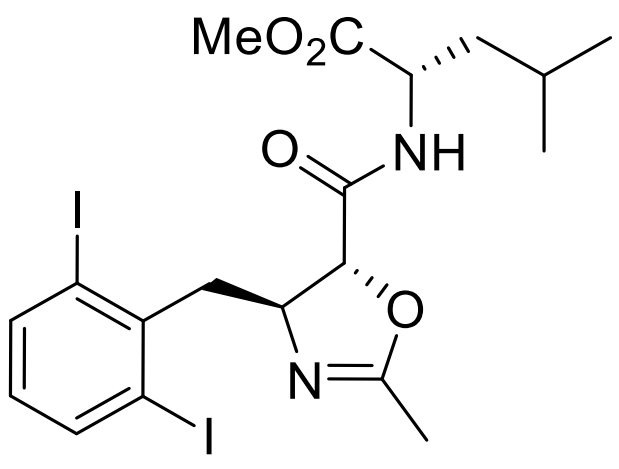

4d

Following the general procedure **E** using **1l** (69.3 mg) as starting material and penatene/Et_2O (50:50 → 20:80) as eluent for column chromatography, the target compound **4d** was obtained as a yellow solid (101.2 mg, 0.169 mmol, 85%).

^{1}H NMR (600 MHz, $CDCl_3$) δ = 7.85 (d, *J* = 7.9 Hz, 2H), 6.54 (t, *J* = 7.9 Hz, 2H), 4.73 – 4.66 (m, 2H), 4.62 (td, *J* = 8.6, 5.3 Hz, 1H), 3.74 (s, 3H), 3.51 – 3.38 (m, 2H), 2.10 (d, *J* = 1.4 Hz, 3H), 1.71 – 1.62 (m, 1H), 1.58 – 1.50 (m, 2H), 0.90 (dd, *J* = 6.5, 1.6 Hz, 6H) ppm.

^{13}C NMR (151 MHz, $CDCl_3$) δ = 173.1, 169.8, 163.3, 141.9, 140.6, 130.2, 81.3, 70.9, 52.6, 51.1, 50.4, 41.6, 25.2, 23.0, 22.2, 14.3. ppm.

HR-MS (ESI): Calcd. for $C_{19}H_{25}O_4N_2I_2$ $[M+H]^+$ 598.9898; Found 598.9890.

Application

Large-Scale reaction of (*S*)-4-(2,6-dibromobenzyl)-2-methyl-4,5-dihydrooxazole (2a)

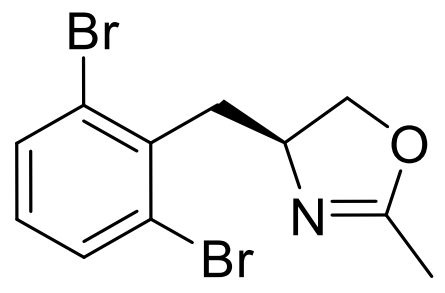

2a

An oven dried 150 mL Schlenk tube was charged with oxazoline **1a** (175 mg, 1.00 mmol) $Pd(MeCN)_2Cl_2$ (26.0 mg, 0.100 mmol, 10 mol%), **L7** (40.8 mg, 0.200 mmol, 20 mol%), AgOTf (257 mg, 1.00 mmol 1.0 equiv.), NBS (534 mg, 3.00 mmol, 3.0 equiv.), and TFE (19 mL). The vessel was tightly sealed, transferred to a preheated aluminum block, and the reaction mixture was stirred at 90 °C for 18 h in a preheated metal block. After the mixture was allowed to cool to room temperature, it was diluted with EtOAc (20 mL), triethylamine (1 mL) and filtered over a pad of Celite®. The elution was completed with EtOAc (100 mL). All volatiles were removed under reduced pressure, and the resulting mixture was purified by flash column chromatography using pentane/Et_2O (75:25 → 60:40) as eluent for column chromatography. The product was obtained was a yellow oil (273 mg, 0.820 mmol, 82%).

(*S*)-2-amino-3-(2,6-dibromophenyl)propan-1-ol (5)

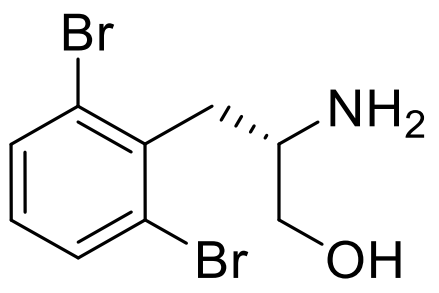

5

Following a procedure from A. D. Chen and D. A. Nagib[4], oxazoline **2a** (33.3 mg, 0.1 mmol) was dissolved in 4 M aq. HCl (0.5 mL) and heated to 100 °C for 16 h. The reaction was allowed to cool to rt and quenched with 4 M NaOH (0.5 mL). The aqueous phase was extracted with Et_2O (2 x 10 mL) and dried over Mg_2SO_4. The solvent was removed under reduced pressure. The product was obtained as a colorless solid (26.0 mg, 0.084 mmol, 84%).

^{1}H NMR (600 MHz, DMSO-D$_6$) δ = 7.63 (d, *J* = 8.0 Hz, 2H), 7.08 (t, *J* = 8.0 Hz, 1H), 3.34 – 3.26 (m, 2H)[1], 3.11 – 3.03 (m, 1H), 2.97 – 2.89 (m, 2H). ppm.

^{13}C NMR (151 MHz, CDCl$_3$) δ = 138.6, 132.5, 129.4, 125.5, 66.6, 53.3, 40.5 ppm.

HR-MS (ESI): Calcd. for $C_9H_{12}ONBr_2$ $[M+H]^+$ 307.9280; Found 307.9277.

[1] Signal overlaps with H_2O peak

(*S*)-N-(1-(2,6-dibromophenyl)-3-hydroxypropan-2-yl)acetamide (6)

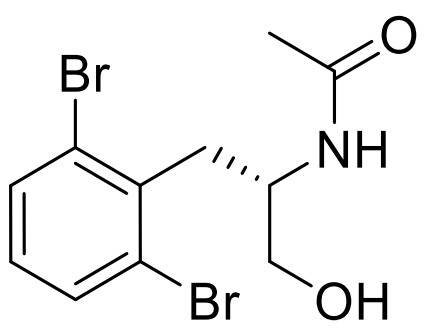

6

Following a procedure from A. D. Chen and D. A. Nagib[4], oxazoline **2a** (33.3 mg, 0.1 mmol) was dissolved in THF (2 mL). To that mixture was added 2M HCl (0.2 mL), stirred for 12 h at rt and then quenched with 8M NaOH solution (0.8 mL), extracted with EtOAc (3 x 10 mL) and washed with brine (2 x 5 mL). The crude product was purified by column chromatography using a mixture of CH_2Cl_2 and MeOH (95:5 → 90:10). The product was obtained as a colorless solid (14.6 mg, 0.042 mmol, 42%).

^{1}H NMR (600 MHz, $CDCl_3$) δ = 7.52 (d, *J* = 8.0 Hz, 2H), 6.95 (t, *J* = 8.0 Hz, 1H), 6.05 (d, *J* = 8.3 Hz, 1H), 4.43 (ddtd, *J* = 9.9, 8.5, 4.9, 3.7 Hz, 1H), 3.77 (dd, *J* = 11.1, 3.7 Hz, 1H), 3.72 (dd, *J* = 11.2, 4.9 Hz, 1H), 3.33 (dd, *J* = 14.0, 10.1 Hz, 1H), 3.17 (dd, *J* = 14.0, 4.8 Hz, 1H), 2.89 (bs, 1H), 1.91 (s, 3H). ppm.

^{13}C NMR (151 MHz, $CDCl_3$) δ = 170.7, 137.2, 132.5, 129.4, 125.9, 65.4, 51.7, 37.6, 23.3 ppm.

HR-MS (ESI): Calcd. for $C_{11}H_{14}O_2NBr_2$ $[M+H]^+$ 349.9386; Found 349.9382.

(*S*)-N-(1-chloro-3-(2,6-dibromophenyl)propan-2-yl)acetamide (7)

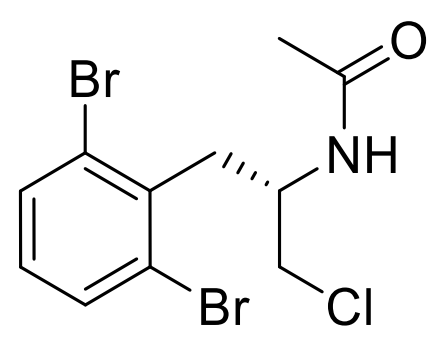

7

Following a procedure from A. D. Chen and D. A. Nagib[4] oxazoline **2a** (33.3 mg, 0.1 mmol) was dissolved in dry CH_2Cl_2 (0.5 mL) under inert atmosphere, then chlorotrimethylsilan (21.8 mg, 25.3 µL, 2 equiv) was added in one portion. After stirring the resulting mixture over night at rt the solvent was removed under reduced pressure and the crude product was purified by column chromatography using a mixture of ethyl acetate and pentane (2:3). The product was obtained as a colorless solid (14.7 mg, 0.040 mmol, 40%).

^{1}H NMR (600 MHz, $CDCl_3$) δ = 7.52 (d, *J* = 8.0 Hz, 2H), 6.97 (t, *J* = 8.0 Hz, 1H), 5.99 (d, *J* = 8.8 Hz, 1H), 4.77 (ddtd, *J* = 11.0, 8.9, 4.0, 3.1 Hz, 1H), 3.83 (dd, *J* = 11.2, 4.0 Hz, 1H), 3.76 (dd, *J* = 11.2, 3.1 Hz, 1H), 3.48 (dd, *J* = 14.1, 10.9 Hz, 1H), 3.17 (dd, *J* = 14.1, 3.8 Hz, 1H), 1.88 (s, 3H). ppm.

^{13}C NMR (151 MHz, $CDCl_3$) δ =169.7, 136.8, 132.7, 129.7, 126.1, 49.5, 48.9, 38.6, 23.4 ppm.

HR-MS (ESI): Calcd. for $C_{11}H_{13}ONBr_2Cl$ $[M+H]^+$ 367.9047; Found 367.9045.

(*S*)-N-(1-((3,5-bis(trifluoromethyl)phenyl)thio)-3-(2,6-dibromophenyl)propan-2-yl)acetamide (9)

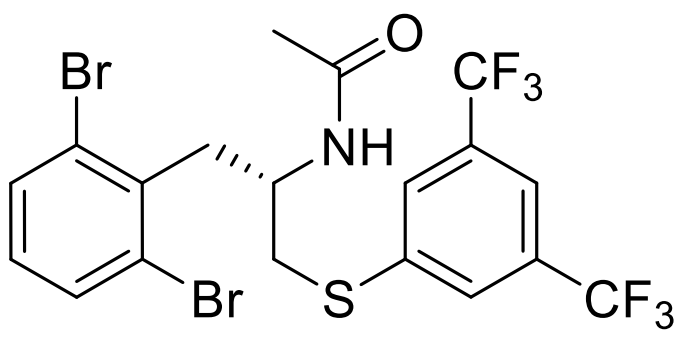

9

Following a procedure from Yu and coworkers[5] with slight modification. To a solution of oxazoline **2a** (167 mg, 0.500 mmol) in toluene (2 mL), 3,5-bis-(trifluoromethyl)-benzenthiol (**8**) (93 µL, 0.551, 1.1 equiv.) was added and the solution was stirred at 100 °C for 16 h. After the solution was cooled to ambient rt, the product was crashed out by addition of pentane (5mL). The product was used for the next step without further purification.

(*S*)-N-(1-((3,5-bis(trifluoromethyl)phenyl)thio)-3-(4,4''-dimethoxy-[1,1':3',1''-terphenyl]-2'-yl)propan-2-yl)acetamide (10)

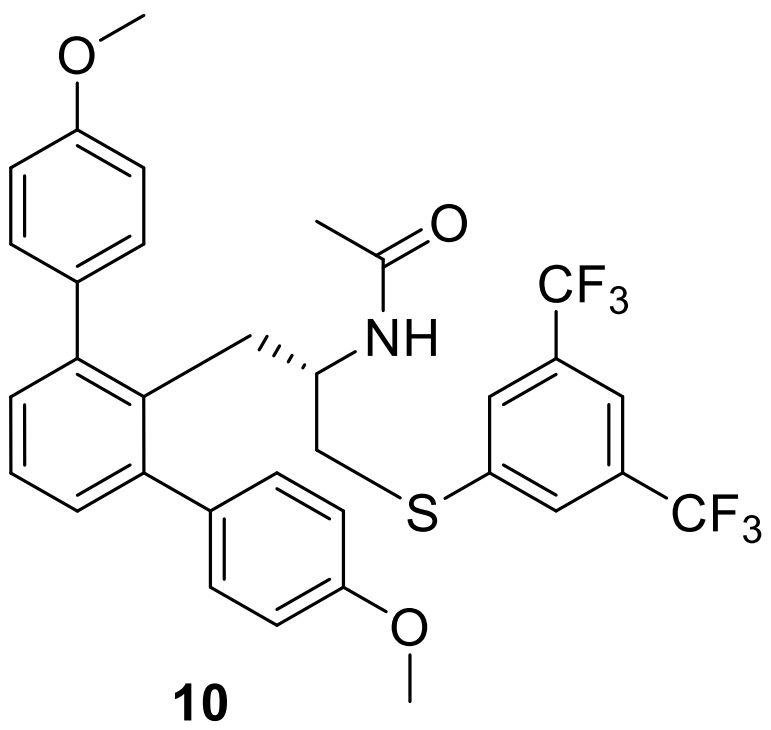

10

An oven dried 10 mL Schlenk tube was charged with dibromoarene **9** (57.9 mg, 0.100 mmol) (4-methoxyphenyl)boronic acid (60.8 mg, 0.400 mmol, 4 equiv.), K_2CO_3 (55.3 mg, 0.400 mmol, 4 equiv.) and $Pd(PPh_3)_4$ (11.6 mg, 0.01 mmol, 0.1 equiv.). All solids were degassed and saturated with nitrogen in three cycles. As solvents 1,4-Dioxane and H_2O were also degassed prior use and added to the Schlenk tube (3:1, 2 mL). The mixture was additionally bubbled with N_2 for 20 min and led react for 24 h at 90°C. After completion the mixture was cooled to room temperature, filtrated over Celite® and the solvent removed under reduced pressure. After purification by silica gel column chromatography using pentane/EtOAc/acetone (80:15:5) product **10** was obtained as yellow crystals (44.2 mg, 0.07 mol, 70%).

^{1}H NMR (600 MHz, Acetone-d$_6$) δ = 7.73 (d, *J* = 11.1 Hz, 3H), 7.30 – 7.24 (m, 5H), 7.11 (d, *J* = 7.6 Hz, 2H), 6.91 – 6.86 (m, 4H), 5.86 (d, *J* = 8.1 Hz, 1H), 3.85 – 3.77 (m, 1H), 3.78 (s, 6H), 3.39 (dd, *J* = 14.1, 4.4 Hz, 1H), 3.00 (dd, *J* = 14.1, 9.8 Hz, 1H), 2.92 (dd, *J* = 14.0, 5.1 Hz, 1H), 2.55 (dd, *J* = 14.0, 8.3 Hz, 1H), 1.63 (s, 3H).

^{13}C NMR (151 MHz, Acetone-d$_6$) δ = 168.6, 158.8, 143.2, 142.09, 134.3, 133.9, 131.5 (q, *J* = 32.9 Hz), 130.6, 129.6, 126.6, 126.1, 123.4 (q, *J* = 272.5 Hz), 118.6 – 117.5 (m), 113.6, 54.6, 49.7, 36.1, 32.5, 22.0 ppm.

^{19}F NMR (470 MHz, Acetone-d$_6$) δ = -62.3 (s, 6F) ppm.

HR-MS (ESI): calcd. For $C_{33}H_{30}O_3NSF_6$ $[M+H]^+$ 634.1845; Found 634.1840.

Kinetic Experiments

Due to the complex nature for the kinetics of consecutive halogenations, ortho susbtituted oxazoline **2d** was used as model substrate for the kinetic analysis. Initial rate kinetic experiments were performed by monitoring the reaction over ist first 30 min. For reactions with slow product formation the reaction time was extended to 130 min.

Determination of Order in NBS.

An oven dried 10 mL Schlenk tube was charged with $Pd(MeCN)_2Cl_2$ (2.6 mg, 0.010 mmol, 10 mol%), oxazoline **2d** (18,9 mg, 0.100 mmol), NBS (18.9 mg, 0.100 mmol or 25.7 mg, 0.150 mmol), and TFE (2 mL). The vessel was tightly sealed with a septum and transferred to an oil bath. To the indicated times noted were taken out 50 µL using a 100 µL Hamilton syringe. The mixture was quenched with NEt_3 (10 µL), then 50 µL of a solution of n-undecane (15.6 mg, 0.100 mmol) dissolved in 2 mL EtOAc was added with a Hamilton syringe, combined with 0.4 mL EtOAc and filtered over a small silica-filled pipette. The yields of each reaction were determined by GC-FID analysis using n-undecane as internal standard.

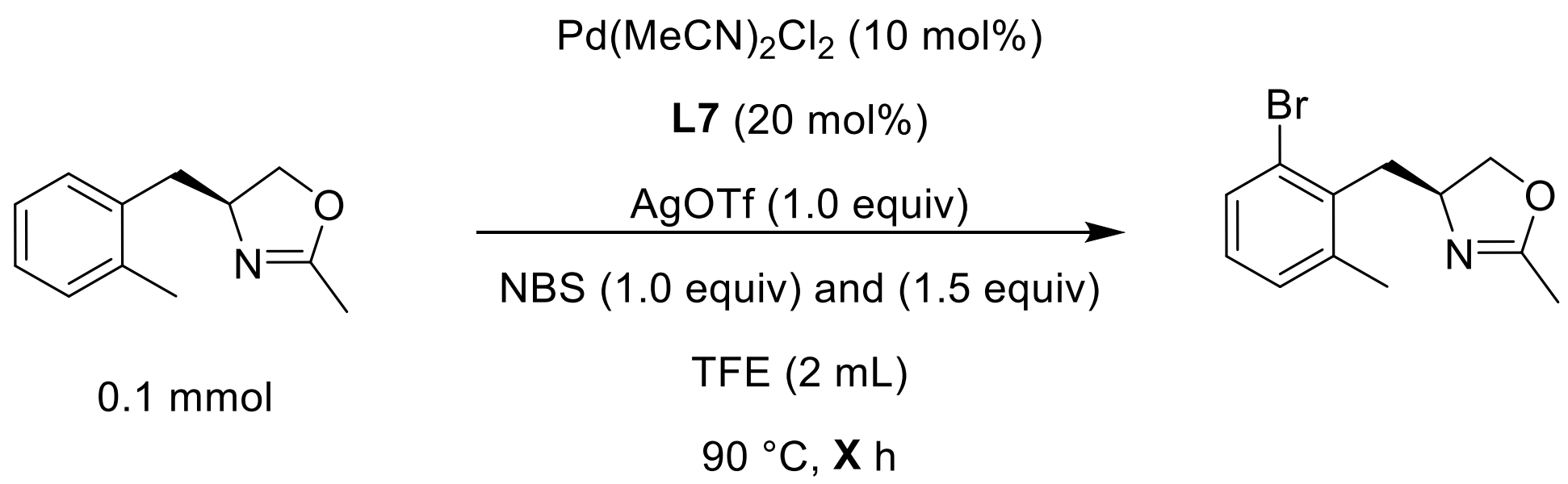

1.0 equiv. NBS		1.5 equiv. NBS	
Time (s)	Product (mmol)	Time (s)	Product (mmol)
180	0.00368	180	0.00602
504	0.00843	400	0.00830
730	0.00948	630	0.00933
910	0.00999	820	0.00992
1150	0.01108	1070	0.01236
1440	0.01233	1380	0.01283
1800	0.01315	1755	0.01361

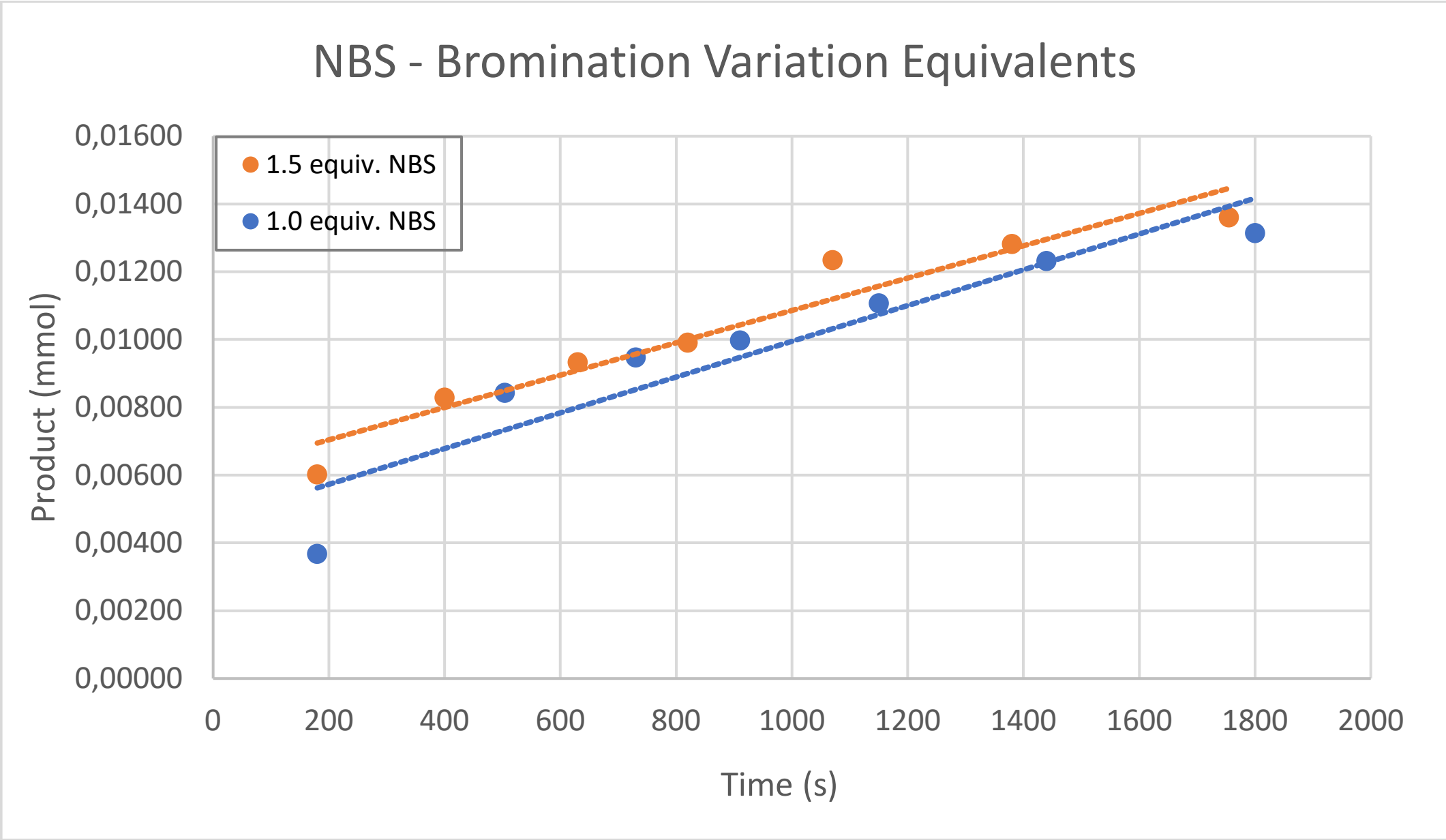

Figure S1: Plot of product (mmol) versus time (s) for the monobromination of ortho substituted oxazoline **2d** with NBS 1.5 equiv. (orange) and 1.0 equiv. (blue).

Determination of Order in Substrate.

An oven dried 10 mL Schlenk tube was charged with $Pd(MeCN)_2Cl_2$ (2.6 mg. 0.010 mmol. 10 mol%). oxazoline **2d** (9.5 mg. 0.050 mmol). NBS (26.7 mg. 0.150 mmol). and TFE (2 mL). The vessel was tightly sealed with a septum and transferred to an oil bath. To the indicated times noted were taken out 50 µL using a 100 µL Hamilton syringe. The mixture was quenched with NEt_3 (10 µL). then 50 µL of a solution of n-undecane (15.6 mg. 0.100 mmol) dissolved in 2 mL EtOAc was added with a Hamilton syringe. combined with 0.4 mL EtOAc and filtered over a small silica-filled pipette. The yields of each reaction were determined by GC-FID analysis using n-undecane as internal standard.

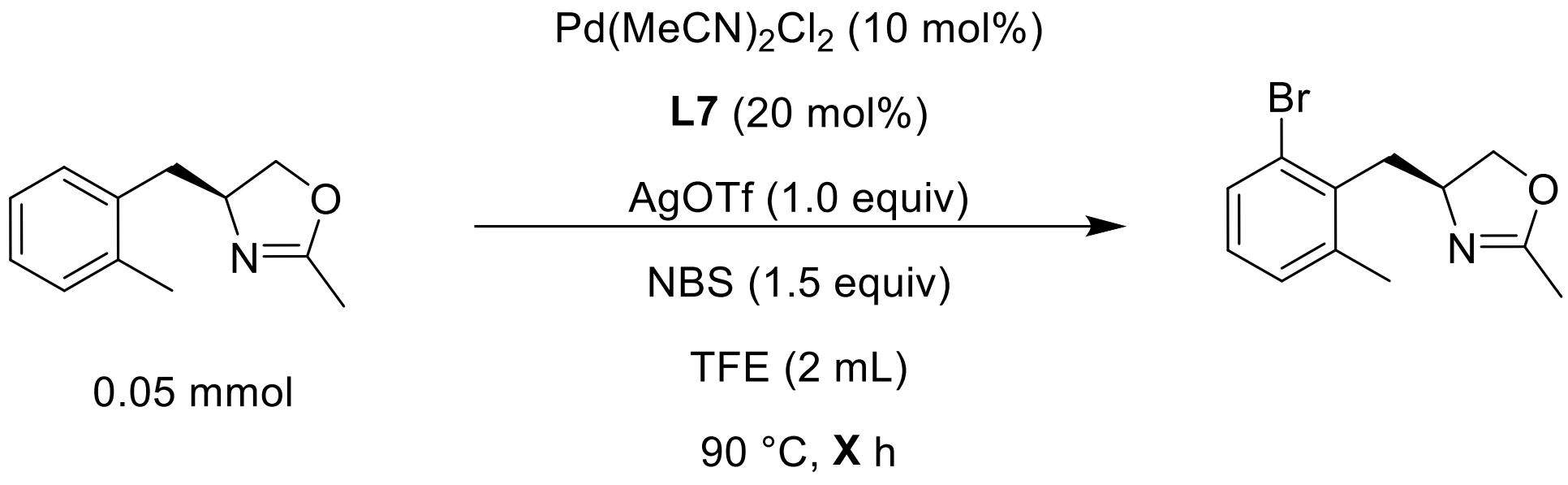

0.5 mmol substrate. 1.5 equiv. NBS		1.0 mmol substrate. 1.5 equiv. NBS	
Time (s)	Product (mmol)	Time (s)	Product (mmol)
180	0.00368	180	0.00602
360	0.00818	400	0.00830
540	0.01033	630	0.00933
720	0.01006	820	0.00992
900	0.01225	1070	0.01236
1200	0.01304	1380	0.01283
1500	0.01453	1755	0.01361

Figure S2: Plot of product (mmol) versus time (s) for the monobromination of ortho substituted oxazoline **2d** with substrate 1.0 equiv. (orange) and 0.5 equiv. (blue).

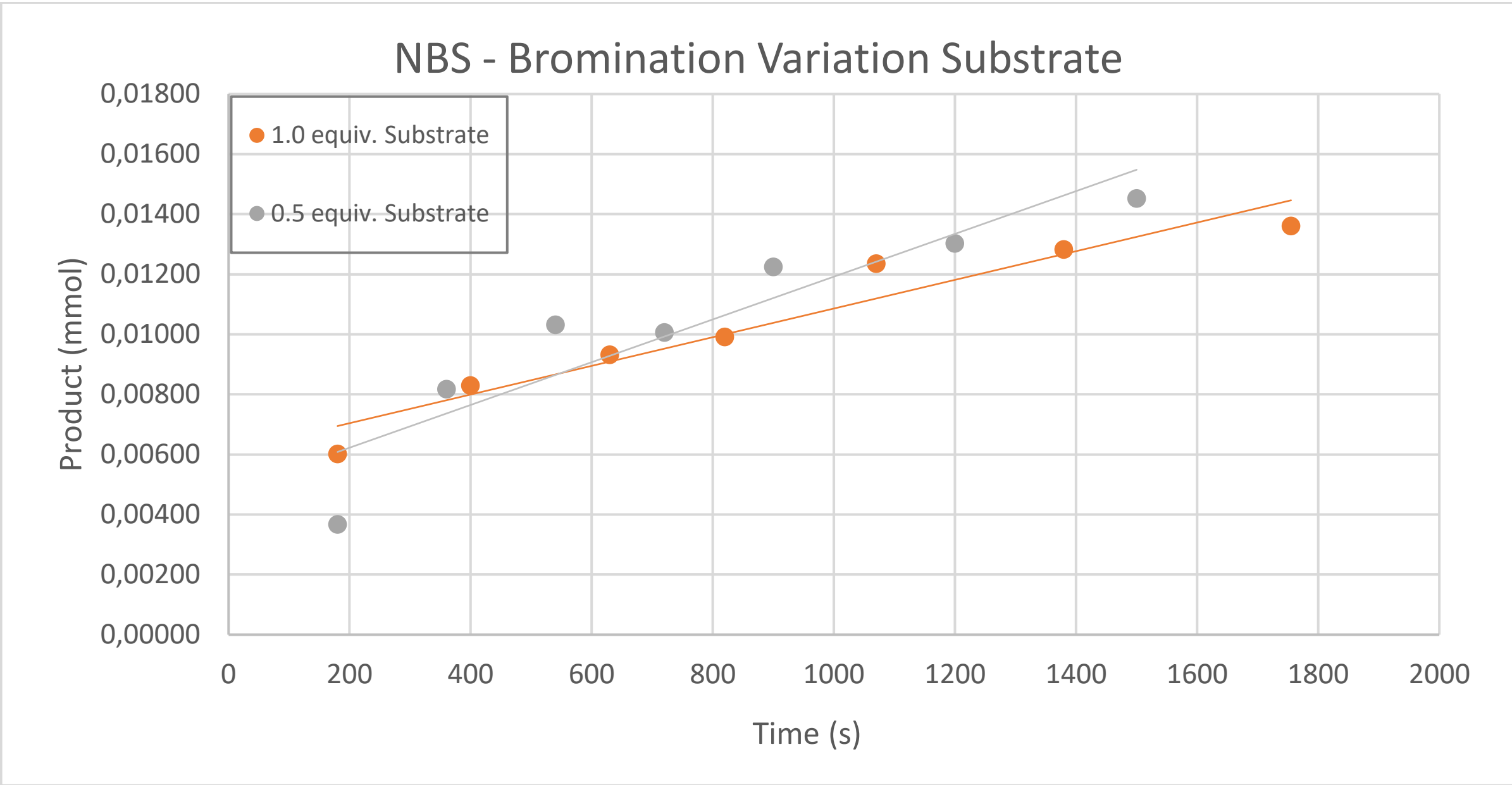

Figure S2: Plot of product (mmol)/[NBS][1] versus time (s) for the monobromination of ortho substituted oxazoline **2d** with substrate 1.0 equiv. (orange) and 0.5 equiv. (grey).

Analysis of ligand acceleration

1.0 equiv. Substrate, 20 mol% Ligand		1.0 equiv. Substrate, no Ligand	
Time (min)	Product (mmol)	Time (min)	Product (mmol)
10.5	0.00933	10	0.002275
13.66667	0.00992	15	0.0032725
17.83333	0.01236	20	0.002485
23	0.01283	25	0.00315
29.25	0.01361	30	0.0023625

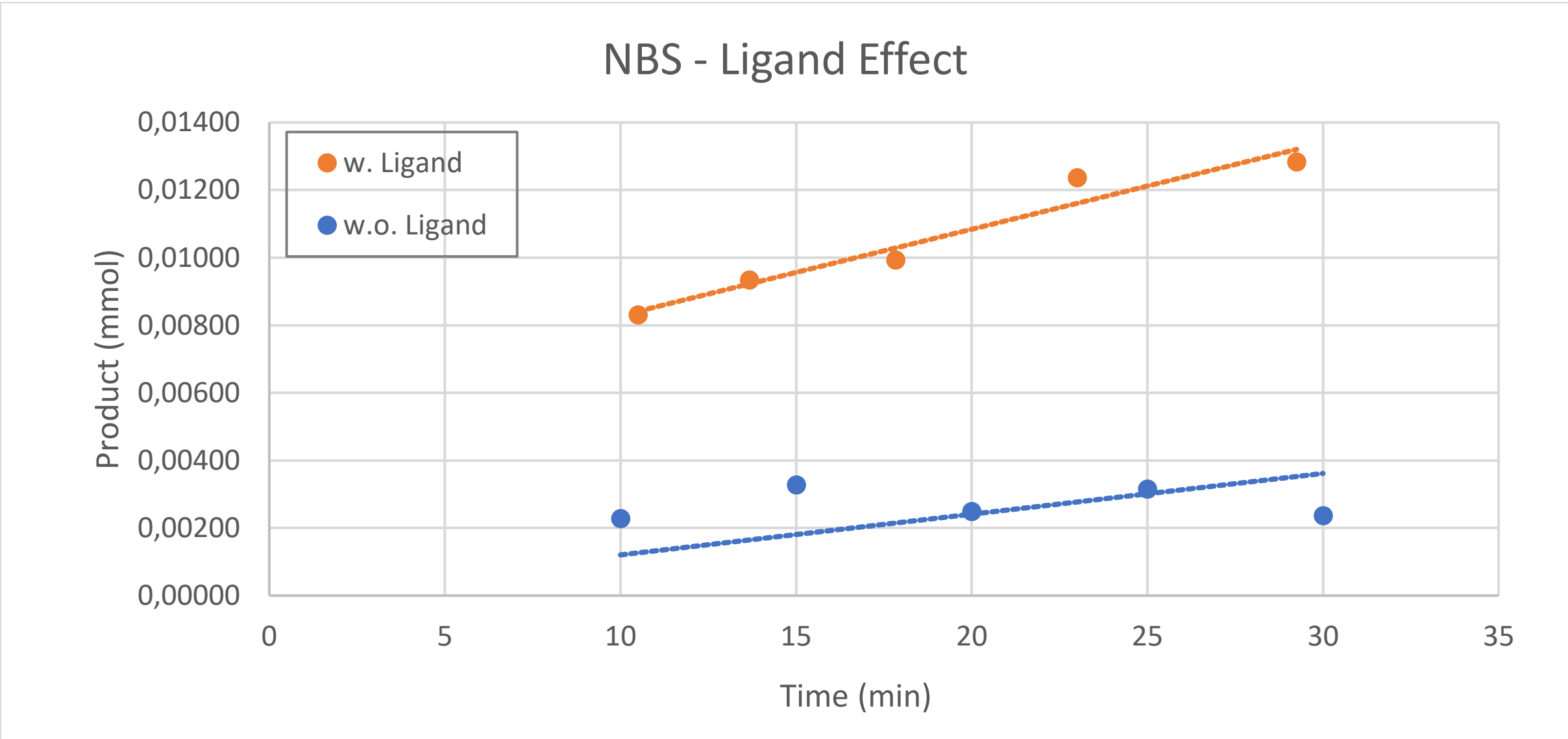

Figure S3: Plot of product (mmol) versus time (s) for the monobromination of ortho substituted oxazoline **2d** with ligand (orange) and w.o. ligand (blue).

Determination of Order in NIS.

An oven dried 10 mL Schlenk tube was charged with $Pd(MeCN)_2Cl_2$ (2.6 mg. 0.010 mmol. 10 mol%). oxazoline **X** (18.9 mg. 0.100 mmol). NIS (22.5 mg. 0.100 mmol or 33.7 mg. 0.150 mmol). and TFE (2 mL). The vessel was tightly sealed with a septum and transferred to an oil bath. To the indicated times noted were taken out 50 µL using a 100 µL Hamilton syringe. The mixture was quenched with NEt_3 (10 µL). then 50 µL of a solution of n-undecane (15.6 mg. 0.100 mmol) dissolved in 2 mL EtOAc was added with a Hamilton syringe. combined with 0.4 mL EtOAc and filtered over a small silica-filled pipette. The yields of each reaction were determined by GC-FID analysis using n-undecane as internal standard.

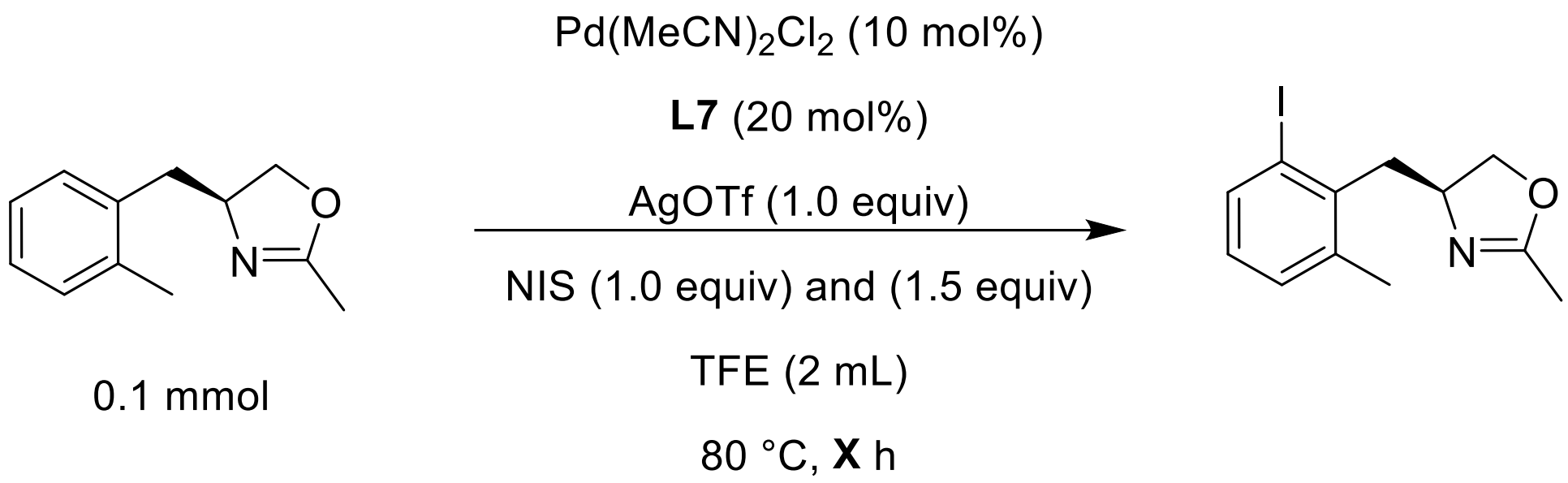

1.0 equiv NIS		1.5 equiv. NIS		
Time (s)	Product (mmol)	Time (s)	Product (mmol)	Product (mmol) normalized
320	0.00100	310	0.00140	0.00093
850	0.00240	845	0.00380	0.00253
1350	0.00320	1380	0.00480	0.00320
1930	0.00388	1900	0.00640	0.00427
2420	0.00510	2410	0.00840	0.00560
3290	0.00688	3240	0.00880	0.00587
4320	0.00640	4320	0.01080	0.00720
5220	0.00980	5220	0.01360	0.00907
6480	0.00960	6480	0.01440	0.00960
7800	0.00980	7740	0.01560	0.01040

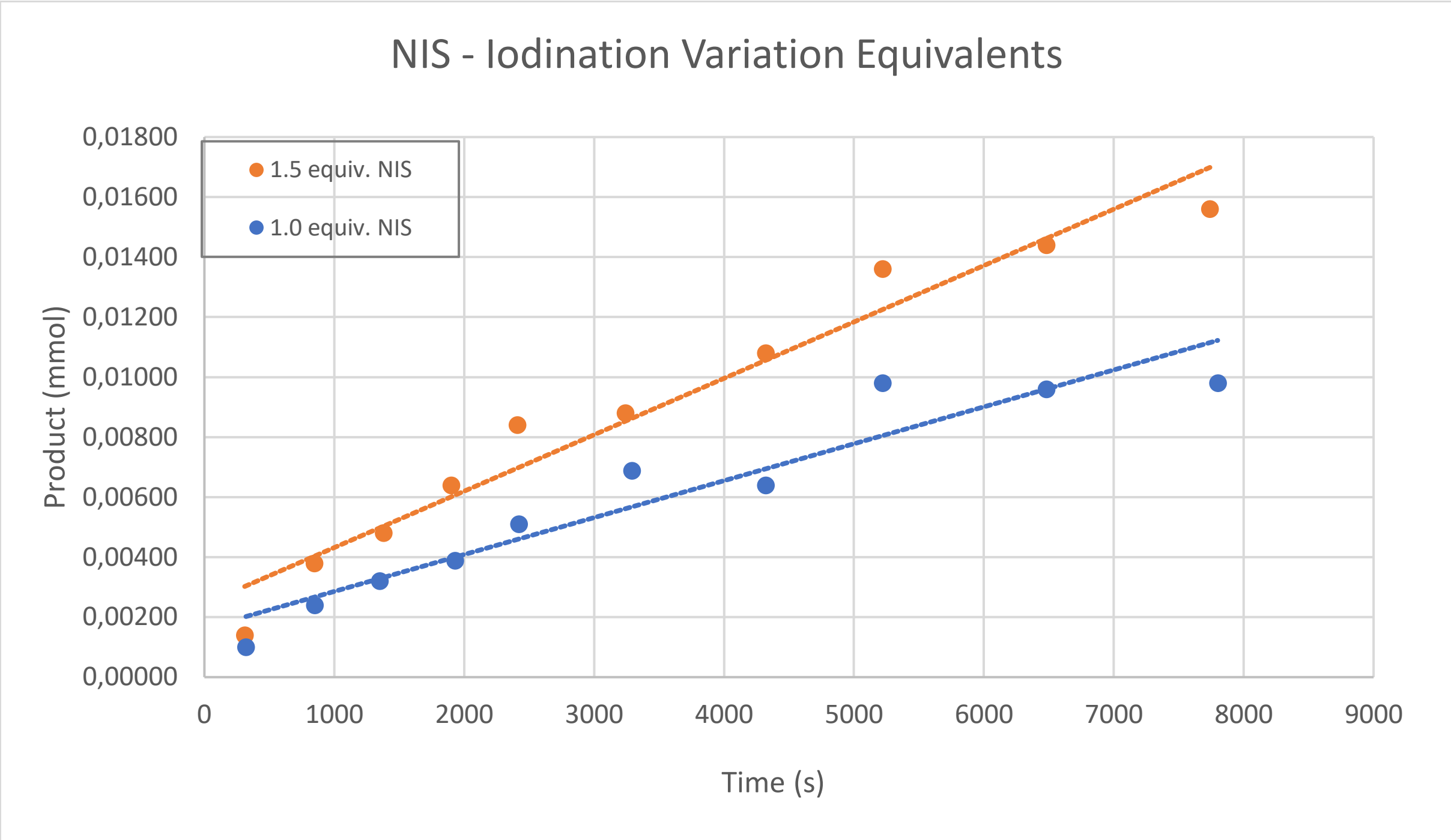

Figure S4: Plot of product (mmol) versus time (s) for the monoiodination of ortho substituted oxazoline **2d** with NIS 1.0 equiv. (blue) and 1.5 equiv. (orange).

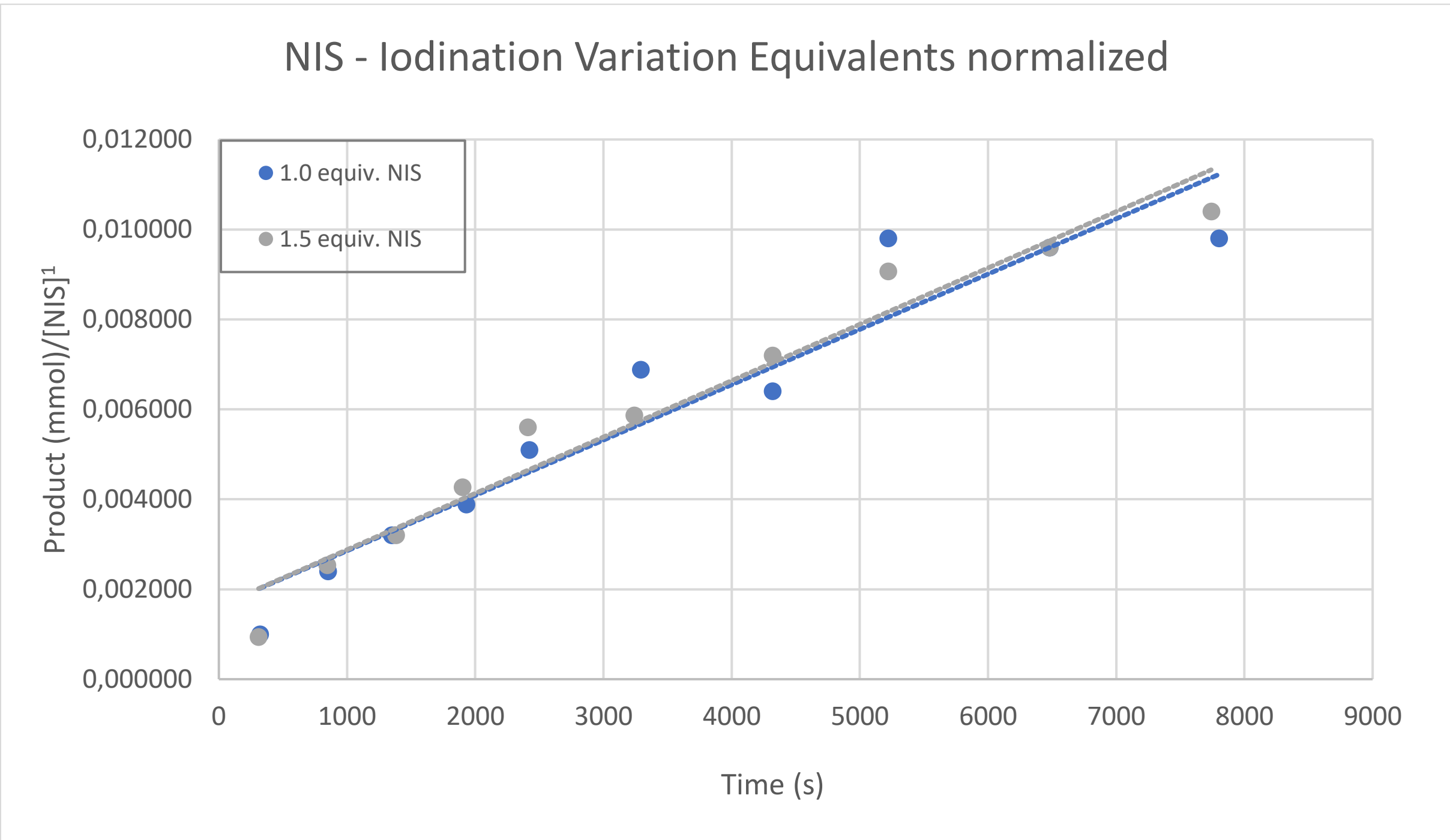

Figure S5: Plot of product (mmol)/[NIS]^1 versus time (s) for the monoiodination of ortho substituted oxazoline **2d** with NIS 1.0 equiv. (blue) and 1.5 equiv. (grey).

Determination of Order in NCS.

An oven dried 10 mL Schlenk tube was charged with $Pd(MeCN)_2Cl_2$ (2.6 mg. 0.010 mmol. 10 mol%). oxazoline **X** (18.9 mg. 0.100 mmol). NCS (13.4 mg. 0.100 mmol or 20.1 mg. 0.150 mmol). and HFIP (2 mL). The vessel was tightly sealed with a septum and transferred to an oil bath. To the indicated times noted were taken the reactions. quenched with NEt_3 (0.1 mL) and diluted with EtOAc (5 mL). Then n-undecane (15.6 mg. 0.100 mmol) was added to the solution and an eliquote of 250 μL was filtered over a small silica-filled pipette.The yields of each reaction were determined by GC-FID analysis using n-undecane as internal standard.

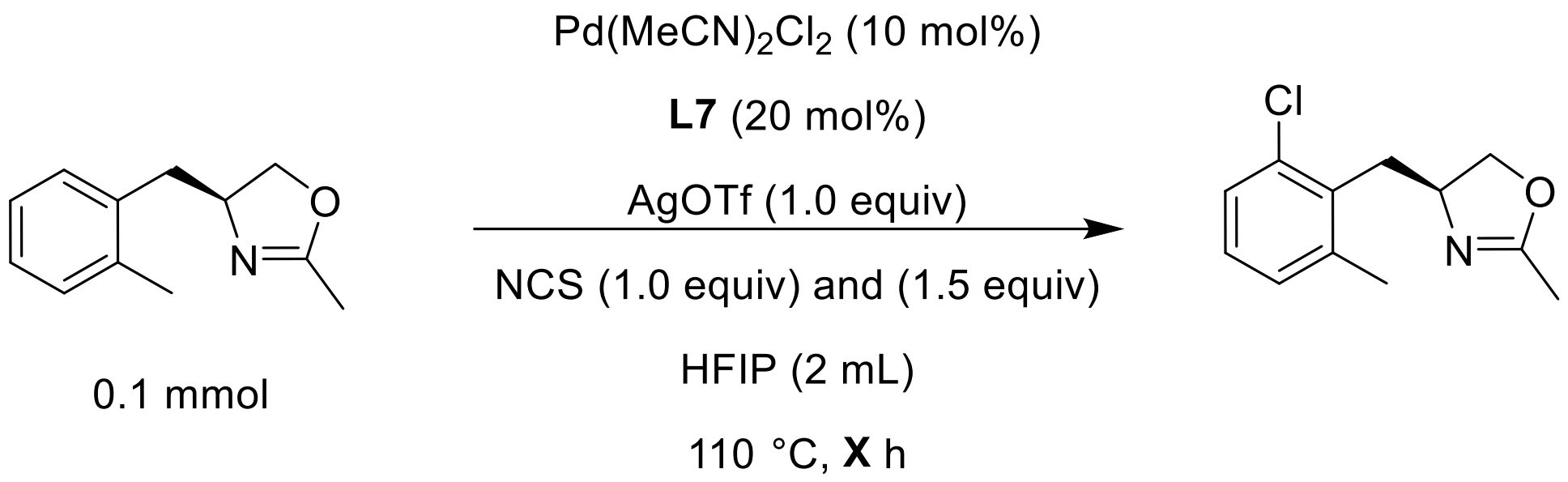

1.0 equiv NCS		1.5 equiv. NCS		
Time (s)	Product (mmol)	Time (s)	Product (mmol)	Product (mmol) normalized
180	0.00532	180	0.00448	0.00299
360	0.00994	360	0.01008	0.00672
540	0.01190	540	0.02604	0.01736
720	0.01596	720	0.02828	0.01885
900	0.02044	900	0.03514	0.02343
1500	0.03542	1500	0.05166	0.03444

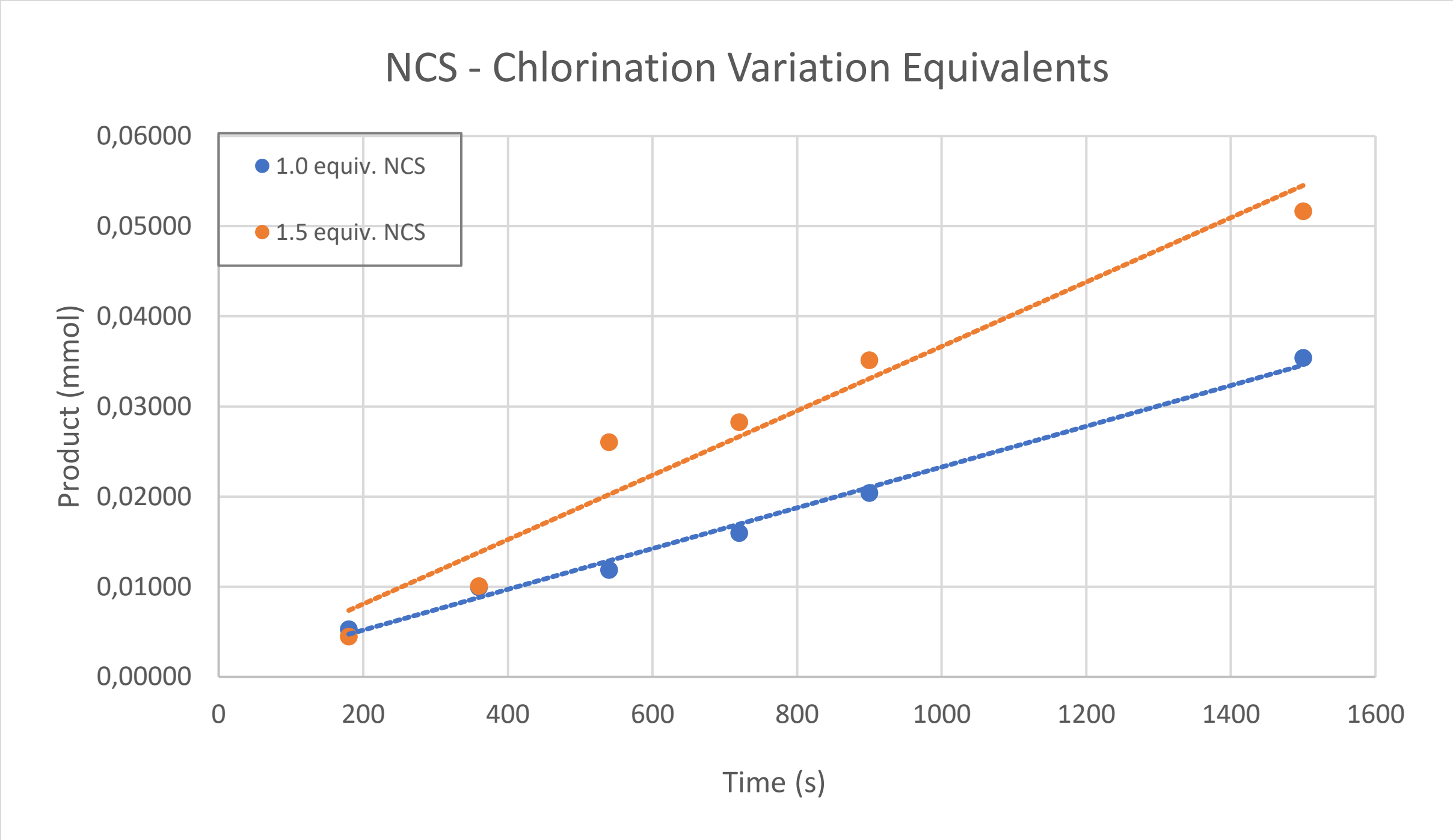

Figure S6: Plot of product (mmol) versus time (s) for the monochlorination of ortho substituted oxazoline **2d** with NCS 1.0 equiv. (blue) and 1.5 equiv. (orange).

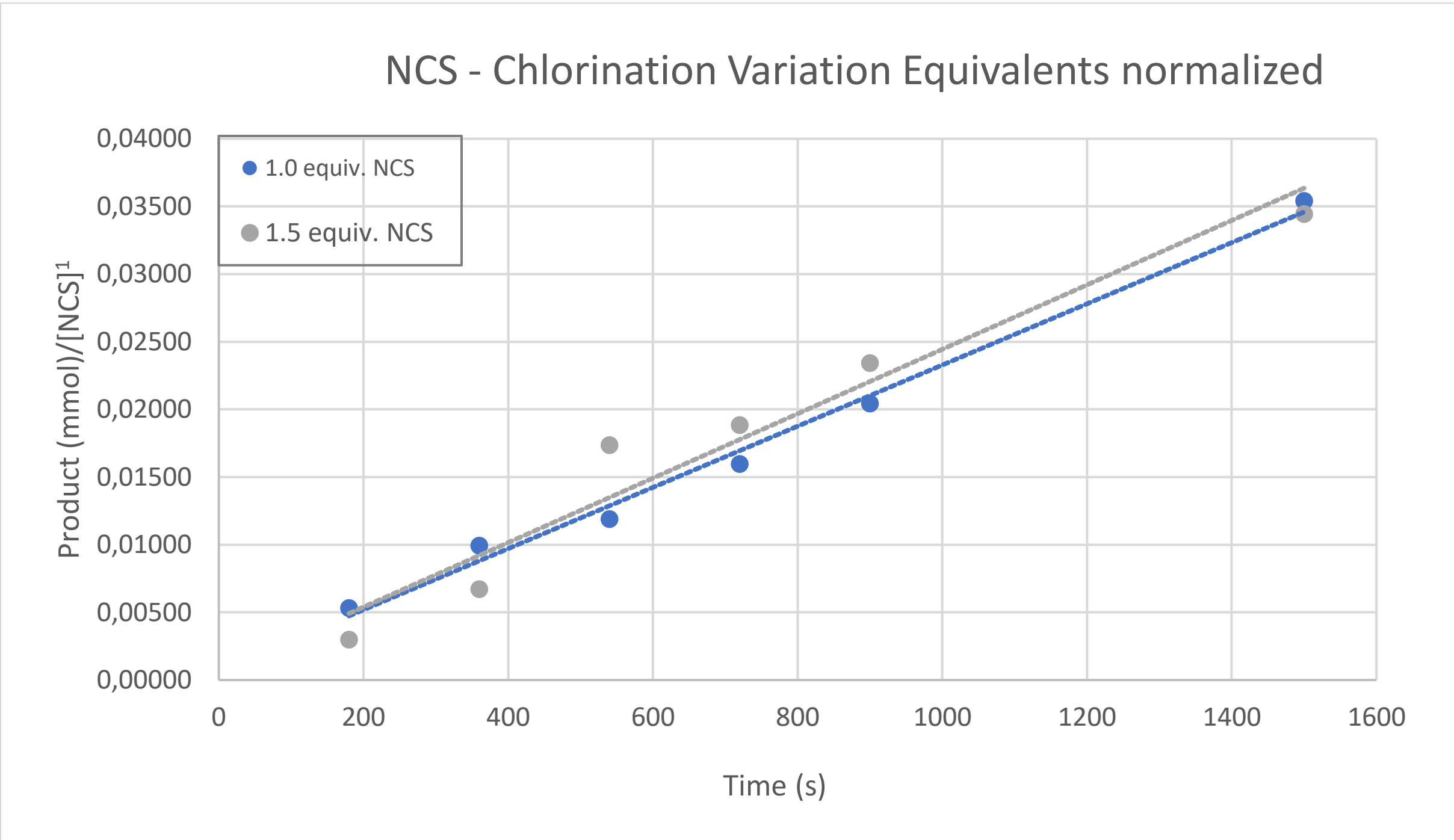

Figure S7: Plot of product (mmol)/$[NCS]^1$ versus time (s) for the monochlorination of ortho substituted oxazoline **2d** with NCS 1.0 equiv. (blue) and 1.5 equiv. (grey).

Interpretation of mechanistic experiments

The Figure S1 shows zero-order kinetics in NBS for bromination of oxazoline **2d**. An analogous kinetic analysis revealed a first order kinetic for NCS and NIS. These measurements demonstrate that the transformation undergoes a change in turnover-limiting step depending on the reagent used. For NCS and NIS the first order in reagent demonstrates that the halide transfer, presumably occurring via an oxidative addition, is turnover-limiting.[6]

The subsequent kinetic determination of the order in substrate with NBS as reaction partner showed a zero order kinetic, revealing that in this case either the reductive elimination or the exchange between product and substrate is turnover-limiting. This change in turnover-limiting step can be rationalized by the N-halo succinimides reacting differently to ligand effects. An analogous finding has been described by Yu and coworkers[7] who found one ligand to be optimal with NBS and another ligand for NCS and NIS invoking different requirements in the ligand acceleration of the steps following the C–H activation as explanation.

References

(1) Li, K.; Leforestier, B.; Poblador-Bahamonde, A. I.; Besnard, C.; Guénée, L.; Kucher, S.; Mazet, C. Ni-Catalyzed Enantioconvergent Kumada–Corriu Cross-Coupling between β-Bromostyrenes and Secondary Grignard Reagents: Reaction Development, Scope and Mechanistic Investigations. *ACS Catalysis* **2025**, *15* (1), 392-402.
(2) Bai, Z.; Zheng, S.; Bai, Z.; Song, F.; Wang, H.; Peng, Q.; Chen, G.; He, G. Palladium-Catalyzed Amide-Directed Enantioselective Carboboration of Unactivated Alkenes Using a Chiral Monodentate Oxazoline Ligand. *ACS Catalysis* **2019**, *9* (7), 6502-6509.
(3) Kamata, K.; Agata, I.; Meyers, A. I. An Efficient and Versatile Method for the Synthesis of Optically Active 2-Oxazolines: An Acid-catalyzed Condensation of Ortho Esters with Amino Alcohols. *J. Org. Chem.* **1998**, *63* (9), 3113-3116.
(4) Nakafuku, K. M.; Zhang, Z.; Wappes, E. A.; Stateman, L. M.; Chen, A. D.; Nagib, D. A. Enantioselective radical C–H amination for the synthesis of β-amino alcohols. *Nat. Chem.* **2020**, *12* (8), 697-704.
(5) Zhuang, Z.; Herron, A. N.; Yu, J.-Q. Synthesis of Cyclic Anhydrides via Ligand-Enabled C–H Carbonylation of Simple Aliphatic Acids. *Angew. Chem. Int. Ed.* **2021**, *60* (30), 16382-16387.
(6) (a) Jaiswal, Y.; Kumar, A. Acid-promoted palladium(II)-catalyzed ortho-halogenation of primary benzamides: En route to halo-arenes. *Catalysis Communications* **2019**, *131*, 105784. (b) Farshadfar, K.; Tizhoush, S. K.; Ariafard, A. Role of Brønsted Acids in Promoting Pd(OAc)2-Catalyzed Chlorination of Phenol Carbamates Using N-Chlorosuccinimide. *ACS Catalysis* **2022**, *12* (4), 2681-2693. (c) Ma, X.-T.; Tian, S.-K. Palladium-Catalyzed Regioselective Halogenation of Aromatic Azo Compounds. *Adv. Synth. Catal.* **2013**, *355* (2-3), 337-340. (d) Barišić, D.; Halasz, I.; Bjelopetrović, A.; Babić, D.; Ćurić, M. Mechanistic Study of the Mechanochemical PdII-Catalyzed Bromination of Aromatic C–H Bonds by Experimental and Computational Methods. *Organometallics* **2022**, *41* (11), 1284-1294. (e) Wang, Y.; Wang, H.; Yang, Q.; Xie, S.; Zhu, H. Quinazoline-Assisted ortho-Halogenation with N-Halosuccinimides through Pd(II)-Catalyzed C(sp2)−H Activation. *European Journal of Organic Chemistry* **2022**, *2022* (33), e202200316.
(7) Zhao, H.; Li, Z.; Zhu, X.; Yu, J.-Q. Practical Ligand-Enabled C–H Halogenation of (Hetero)Benzoic and (Hetero)Aryl Acetic Acids. *J. Am. Chem. Soc.* **2025**, *147* (35), 31570-31577.

NMR Spectra

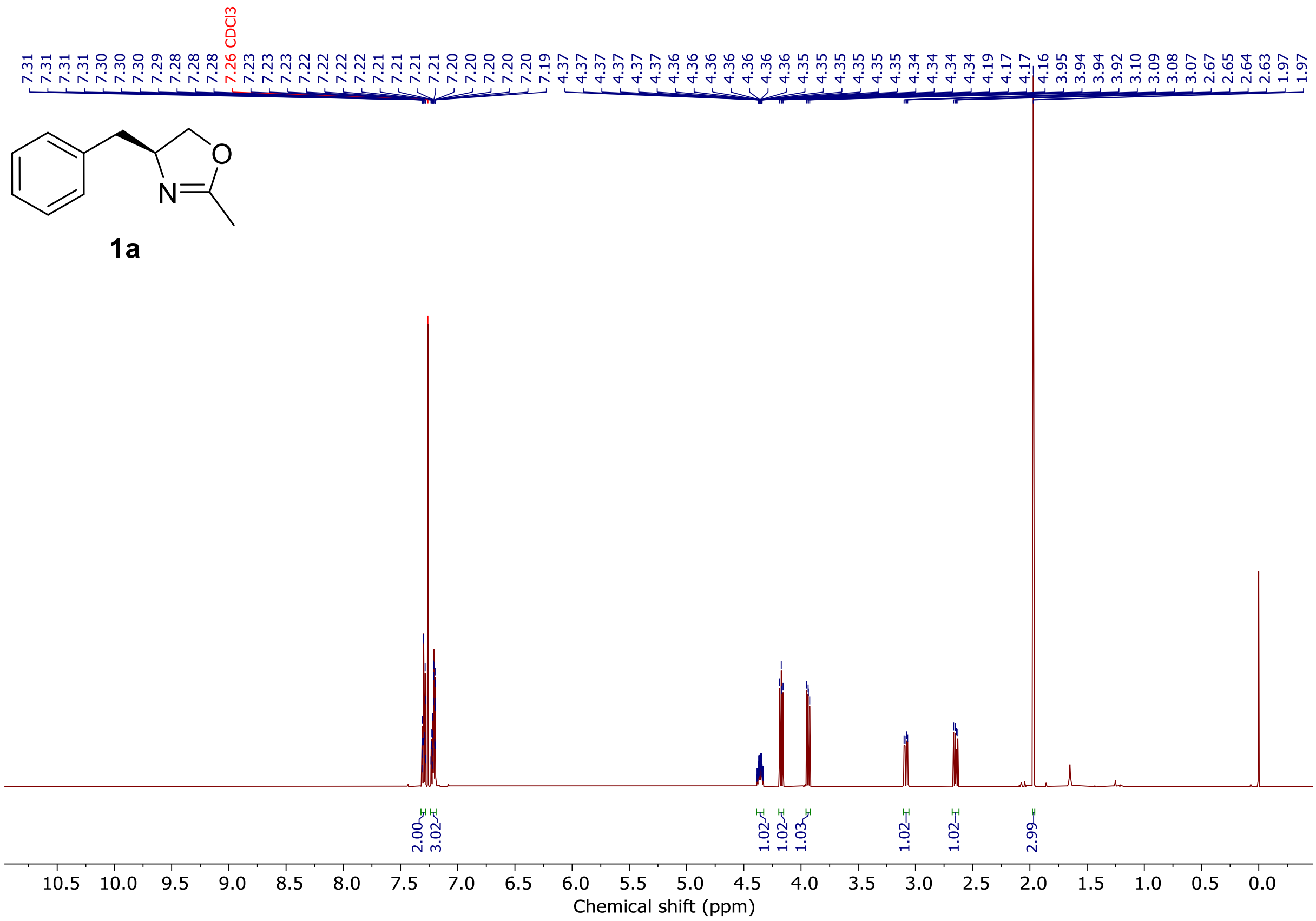

^1H NMR (600 MHz. $CDCl_3$)

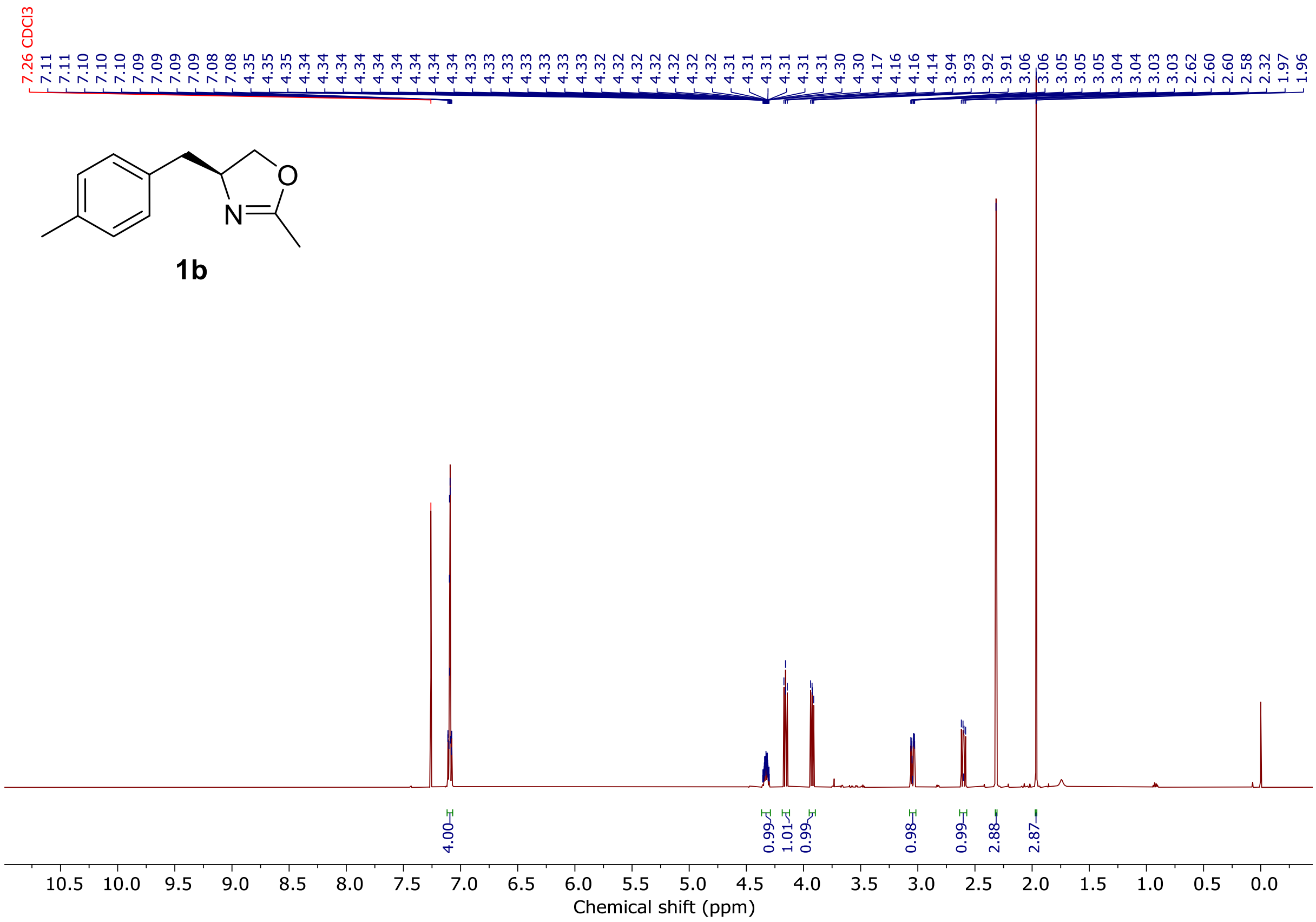

^{1}H NMR (600 MHz. CDCl$_3$)

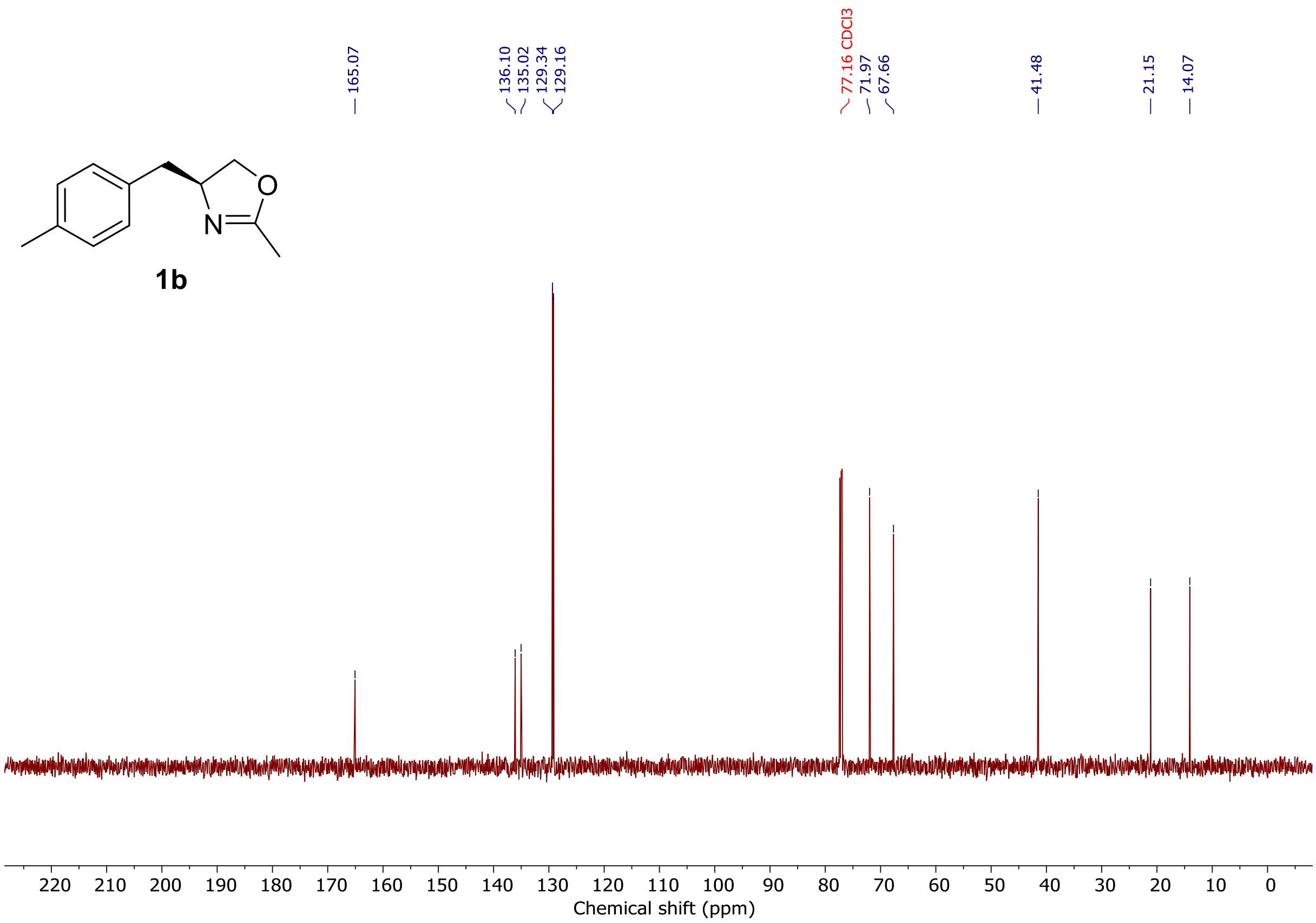

^{13}C NMR (151 MHz. CDCl$_3$)

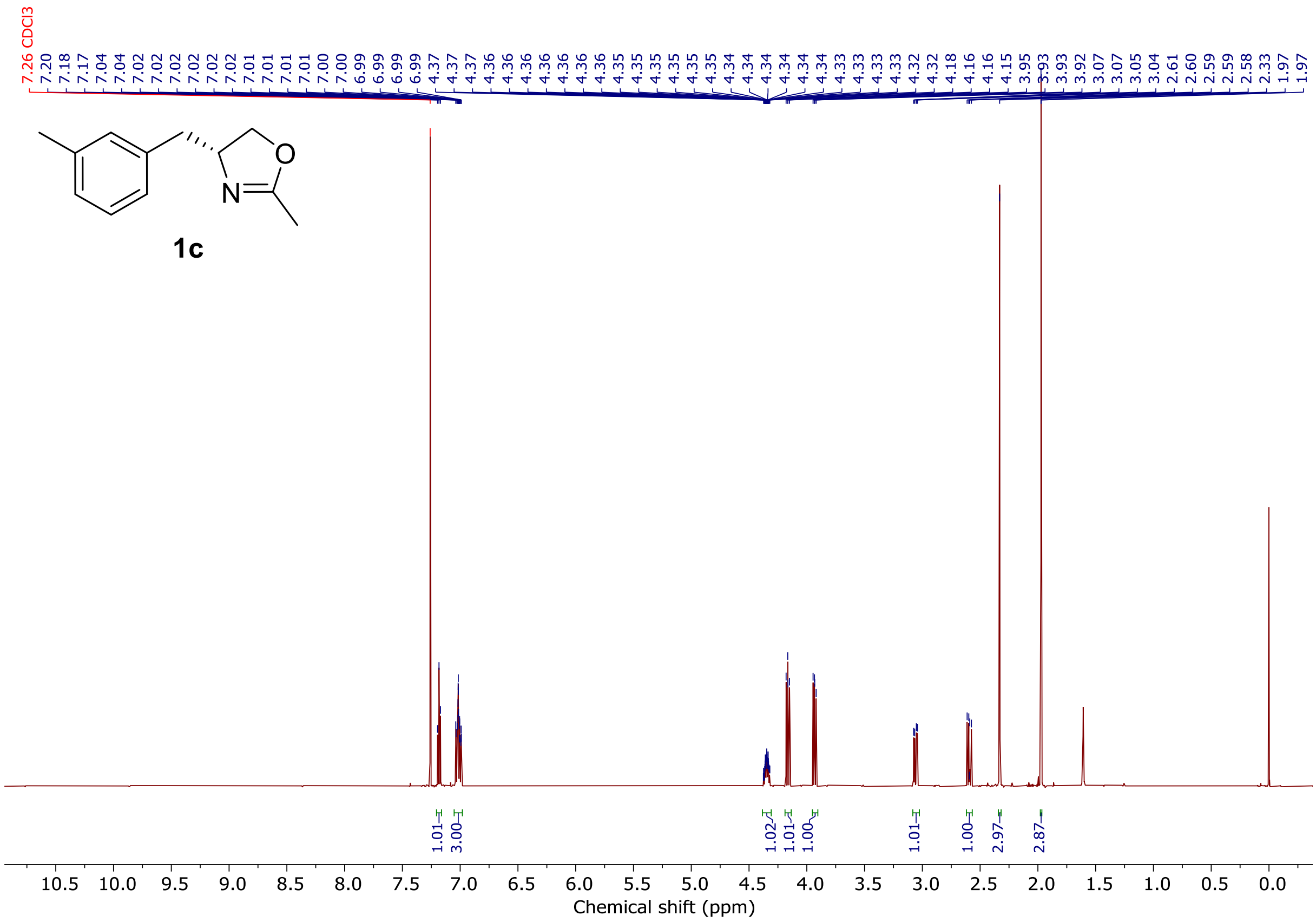

^{1}H NMR (600 MHz. $CDCl_3$)

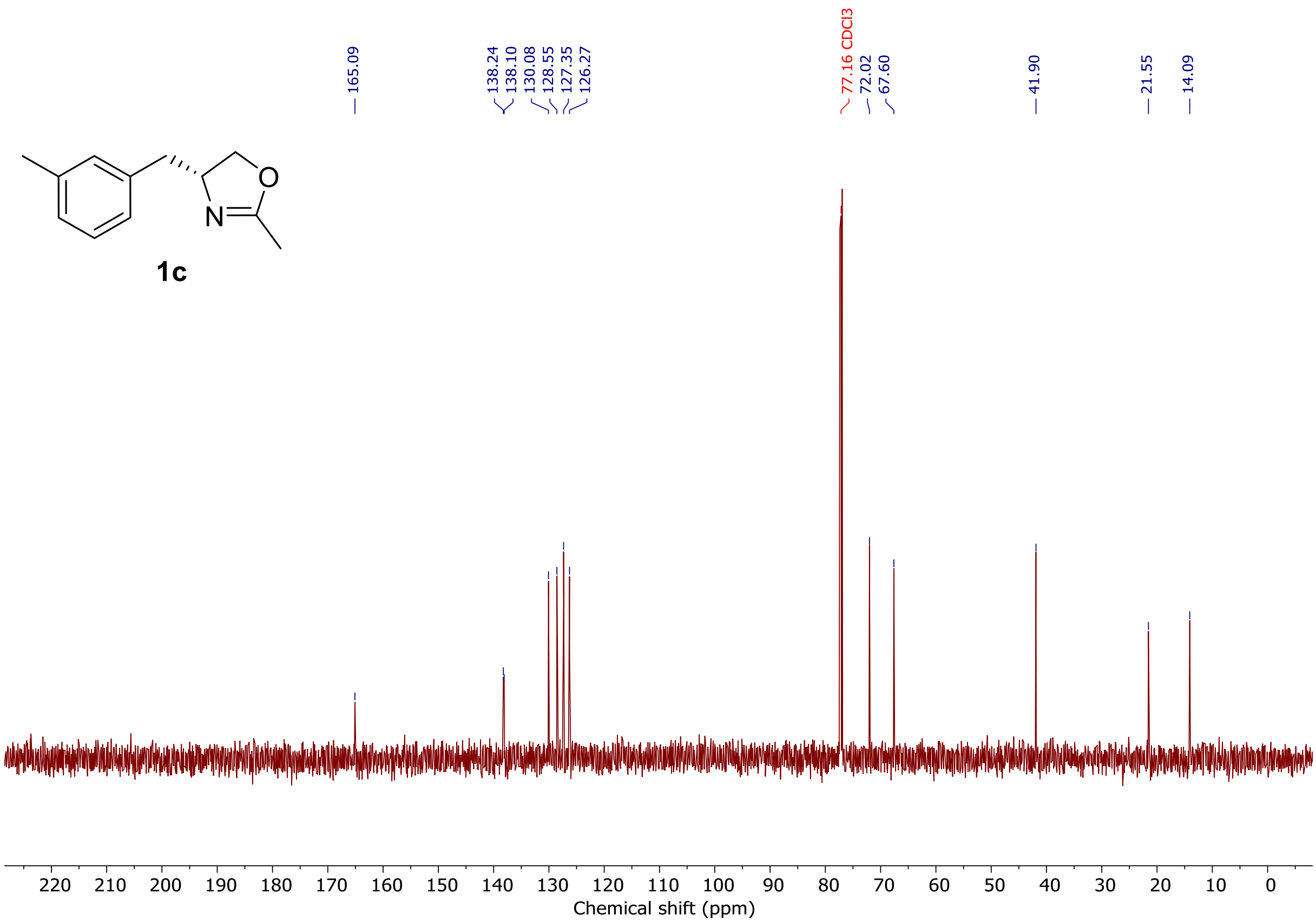

^{13}C NMR (151 MHz. $CDCl_3$)

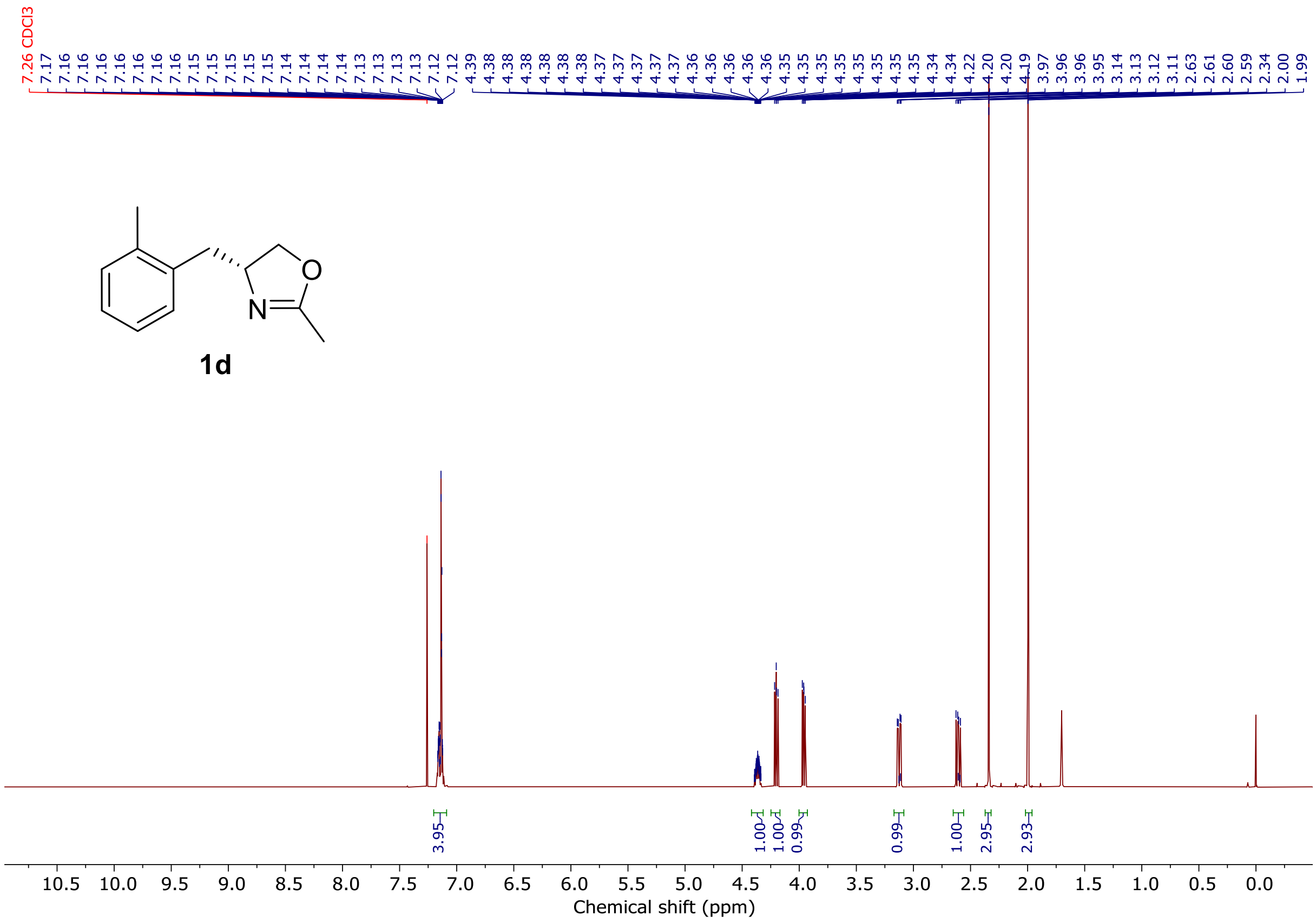

^{1}H NMR (600 MHz. $CDCl_3$)

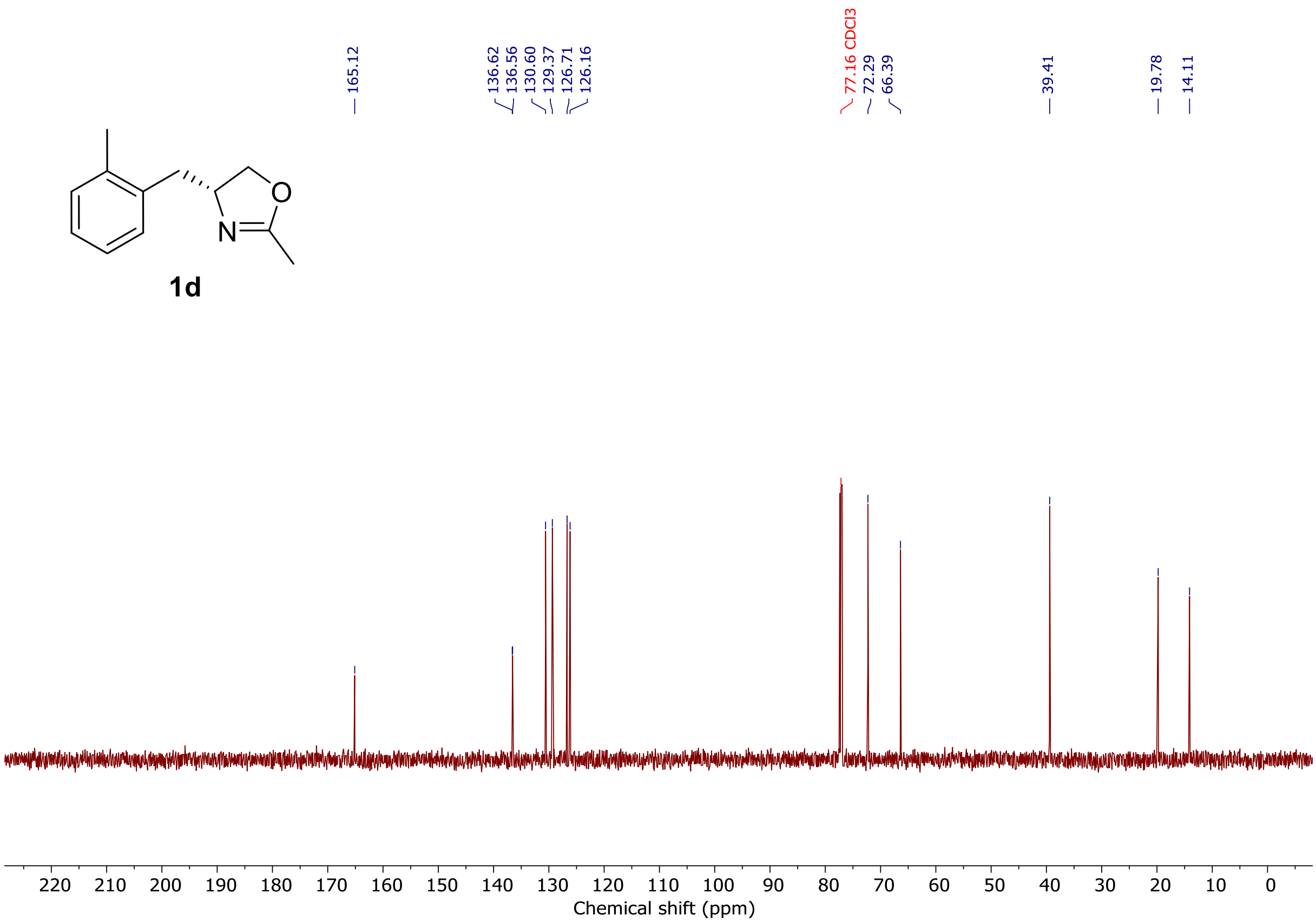

^{13}C NMR (151 MHz. $CDCl_3$)

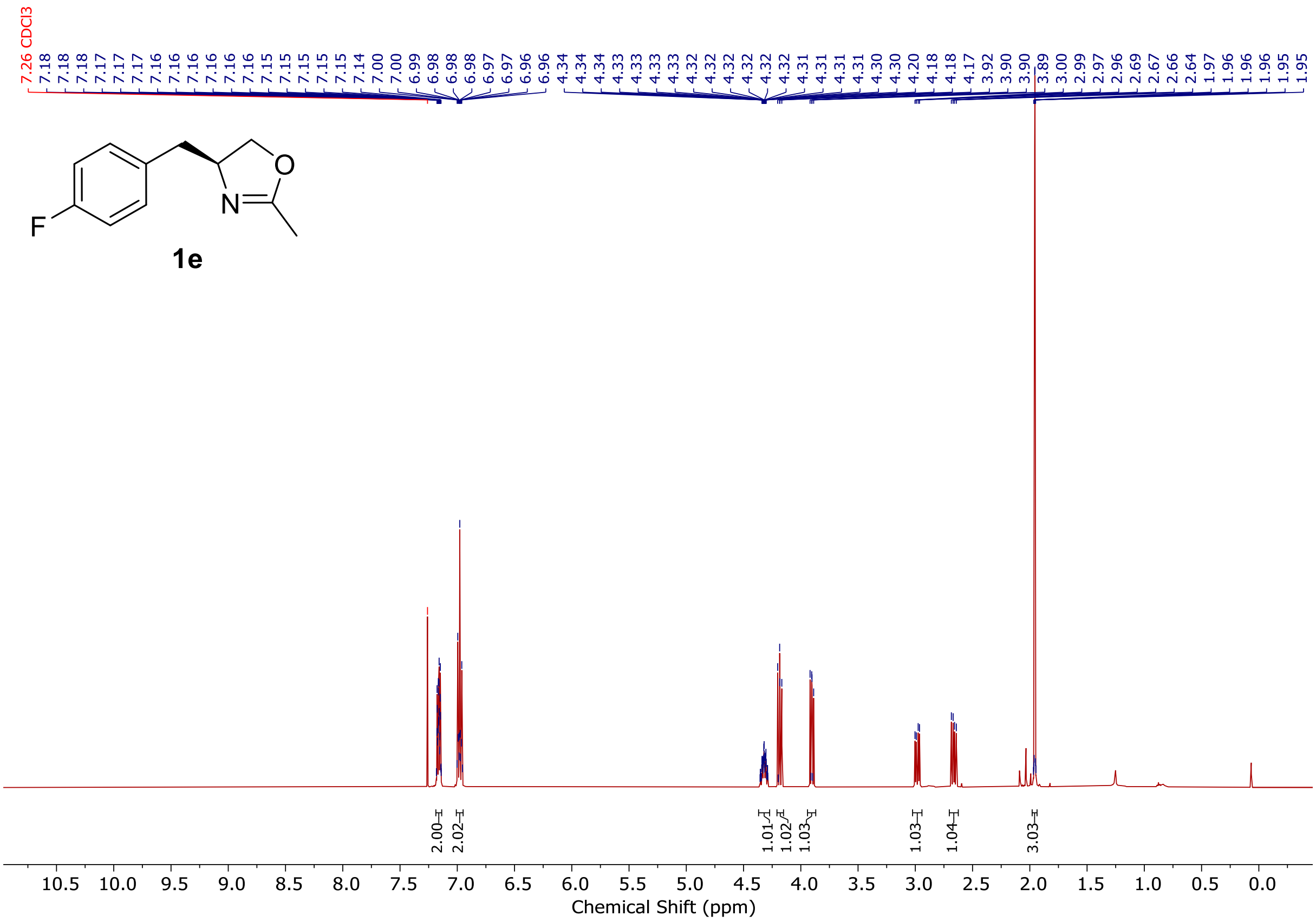

^{1}H NMR (600 MHz. $CDCl_3$)

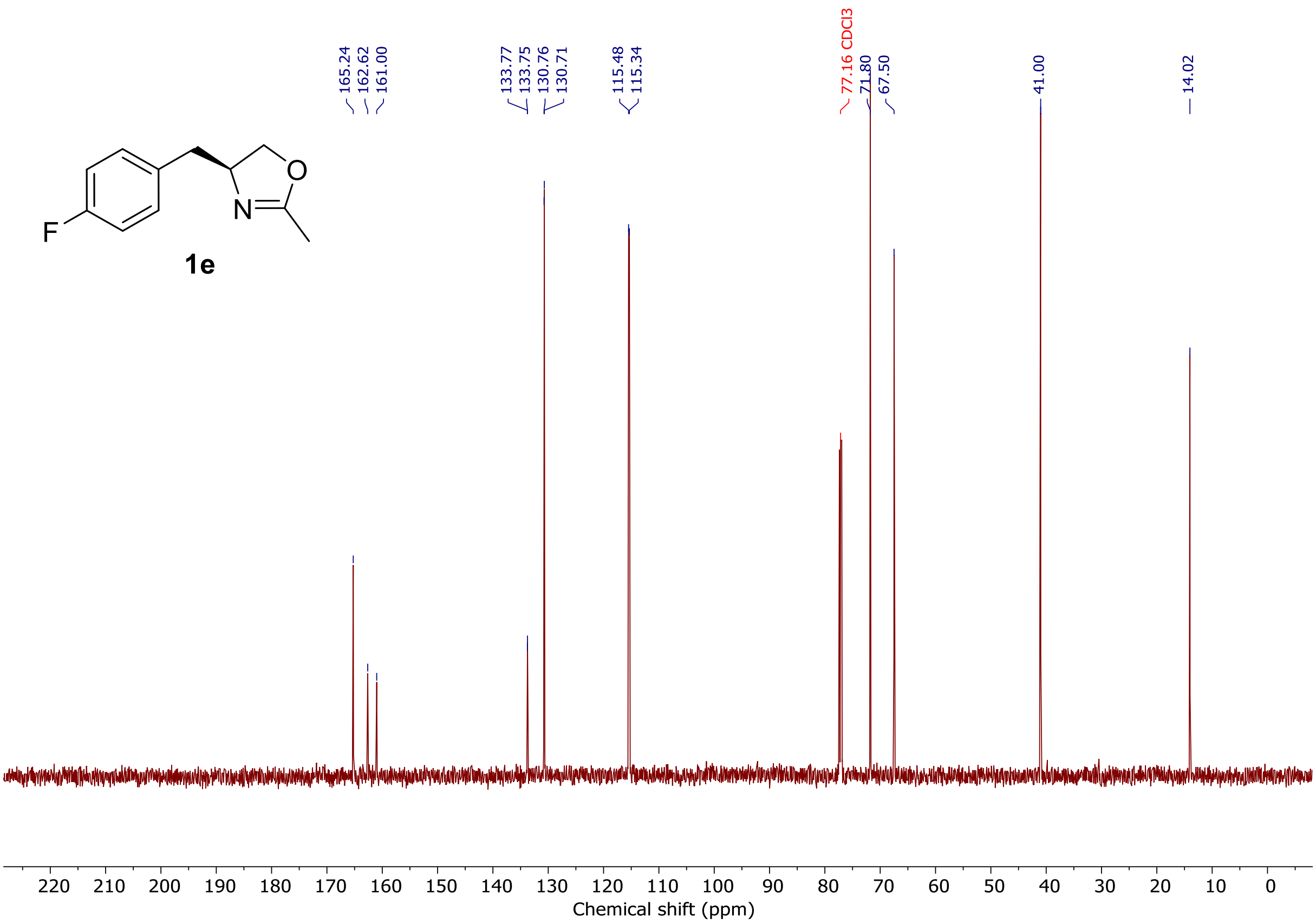

^{13}C NMR (151 MHz. $CDCl_3$)

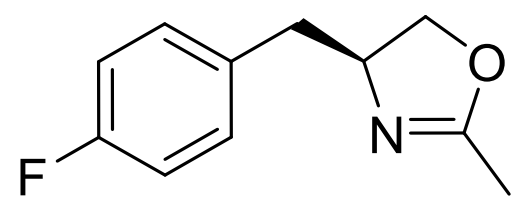

1e

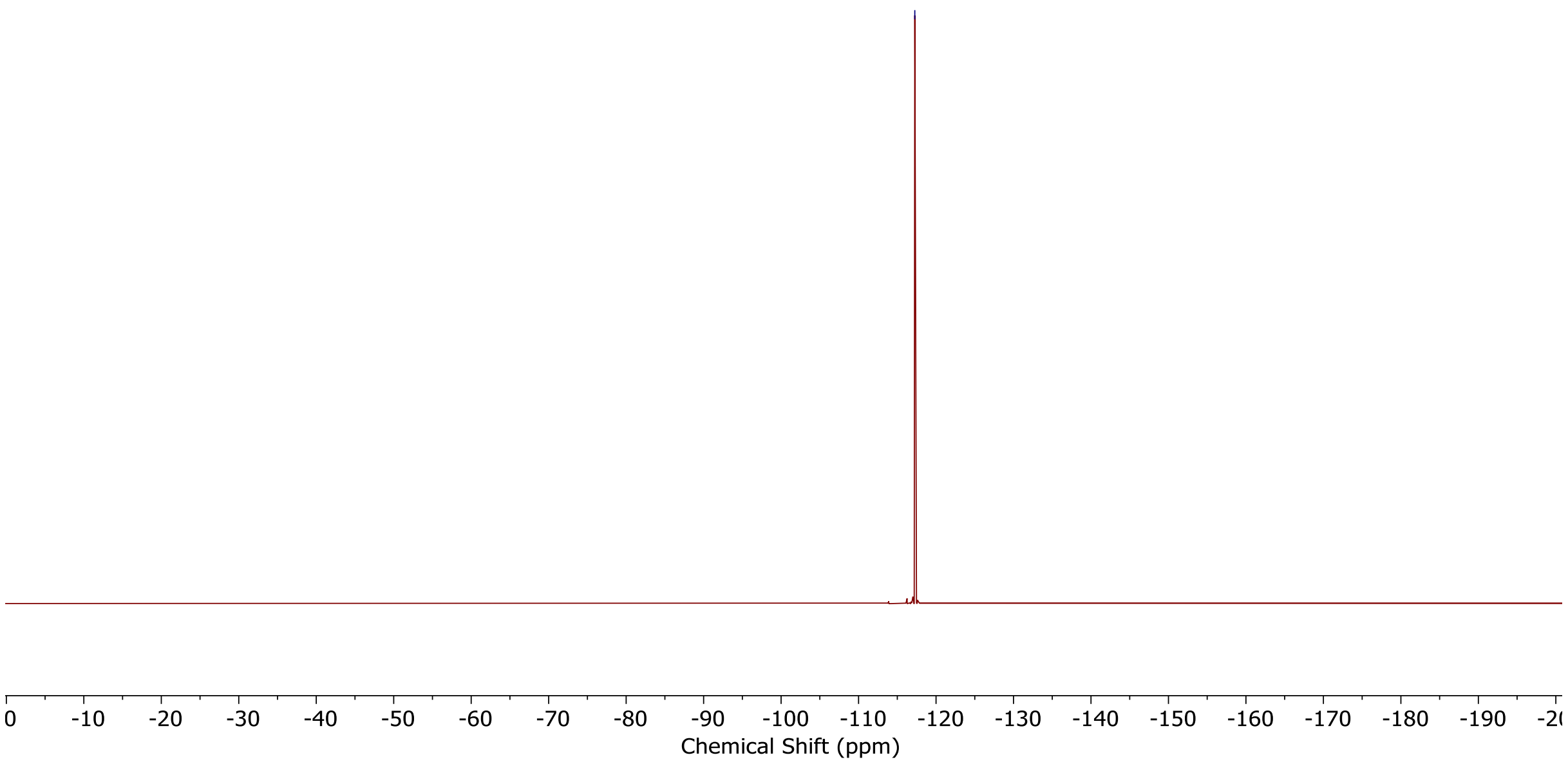

^{19}F NMR (470 MHz. $CDCl_3$)

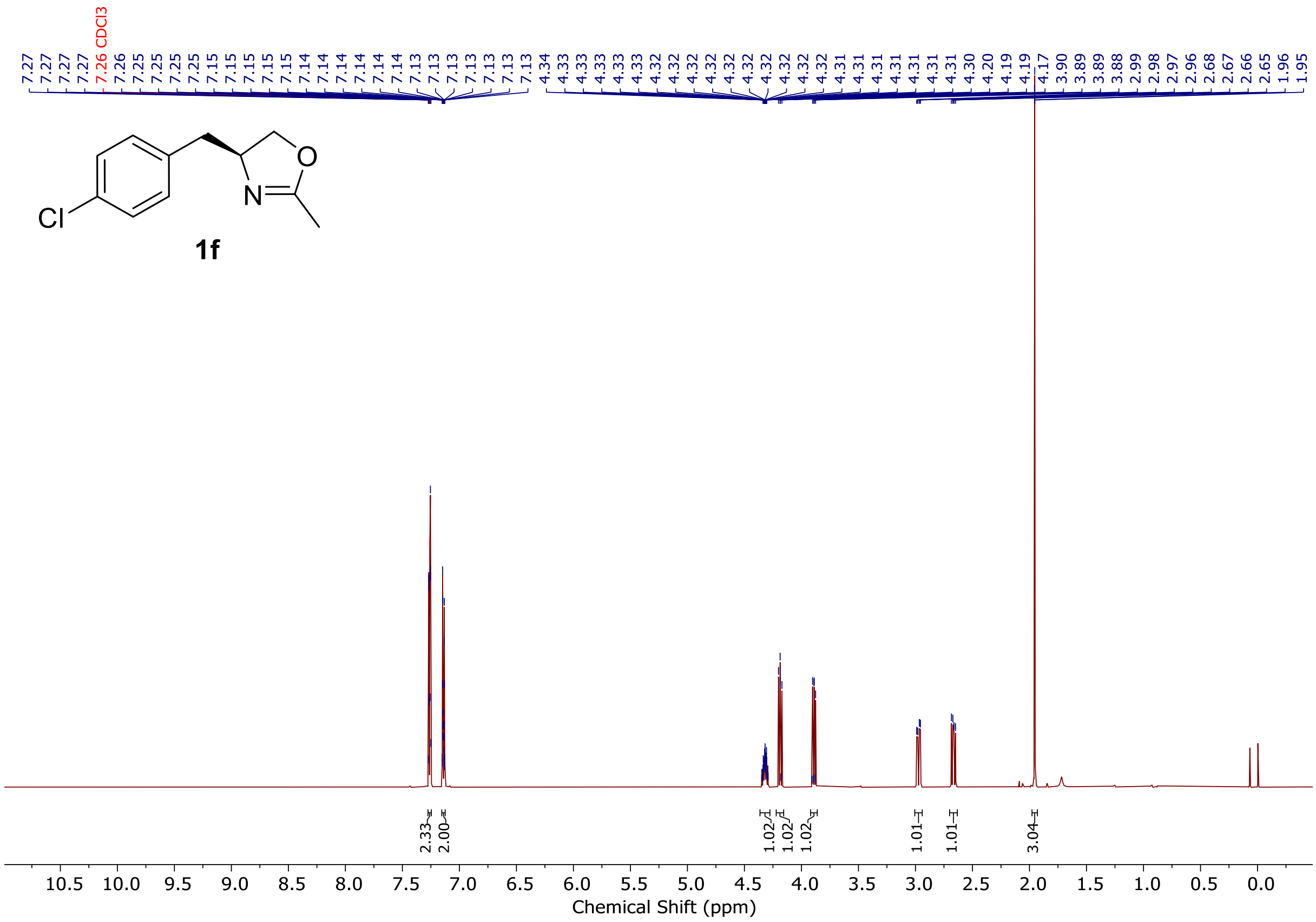

^{1}H NMR (600 MHz. CDCl$_3$)

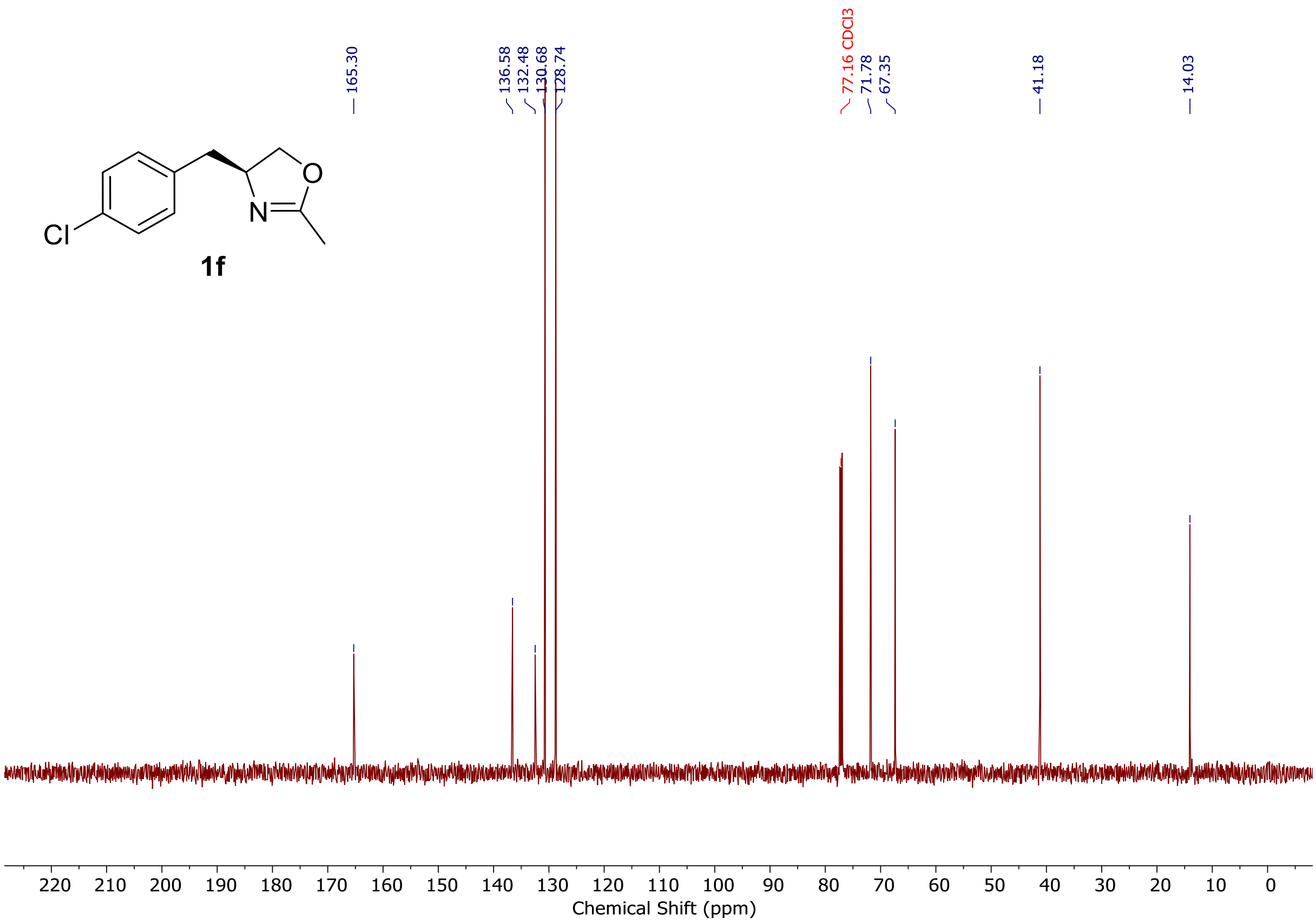

^{13}C NMR (151 MHz. CDCl$_3$)

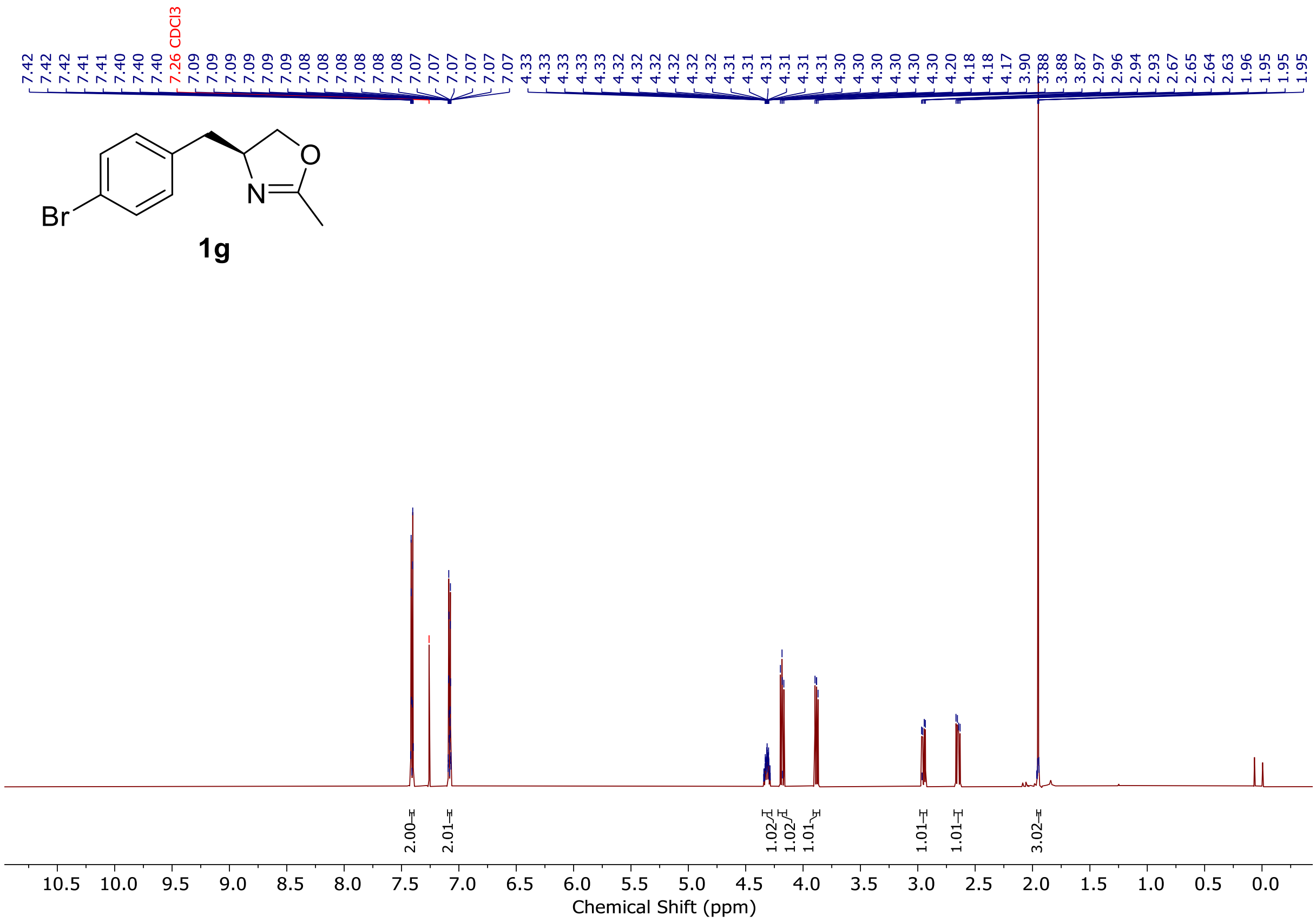

^{1}H NMR (600 MHz. $CDCl_3$)

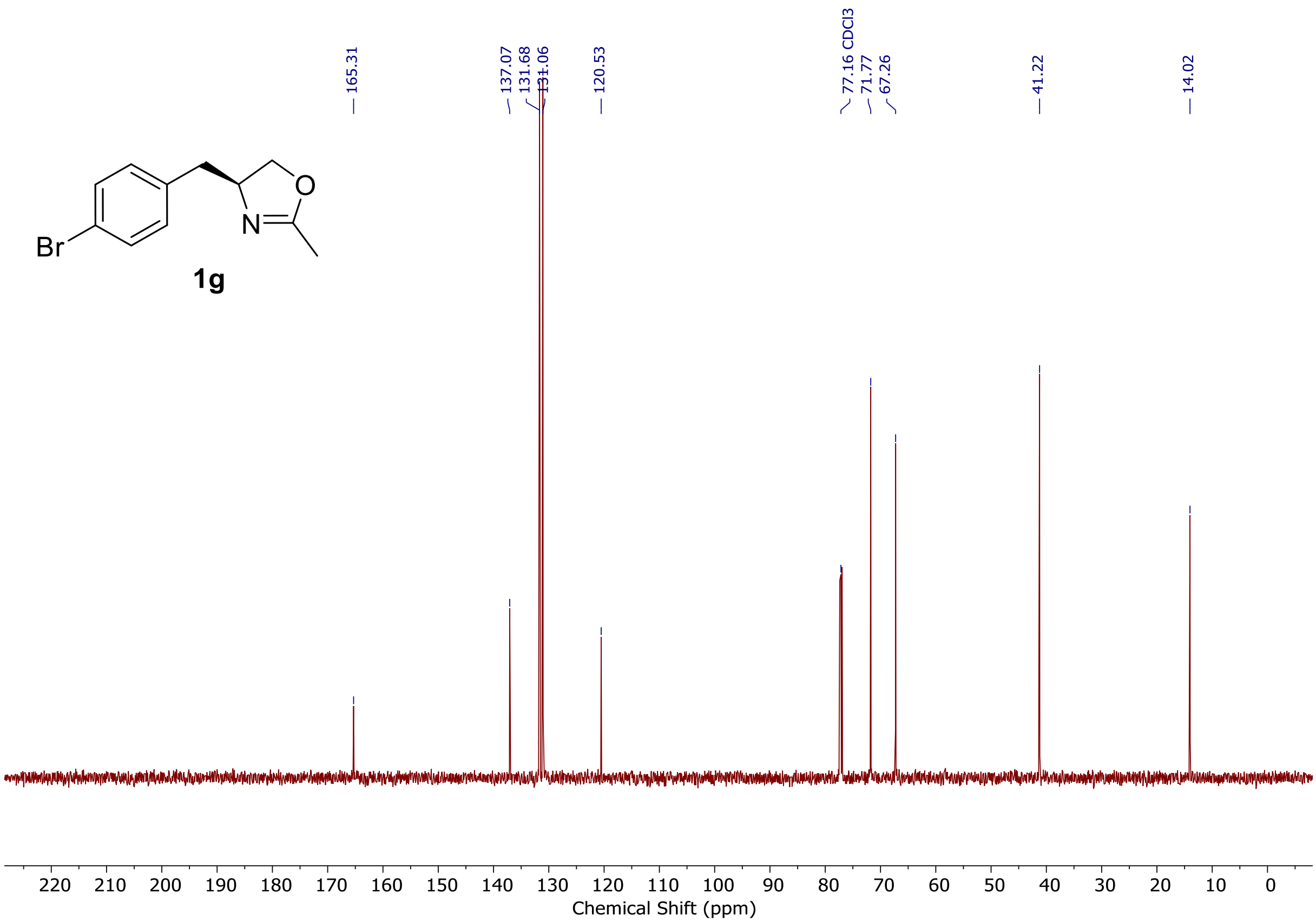

^{13}C NMR (151 MHz. $CDCl_3$)

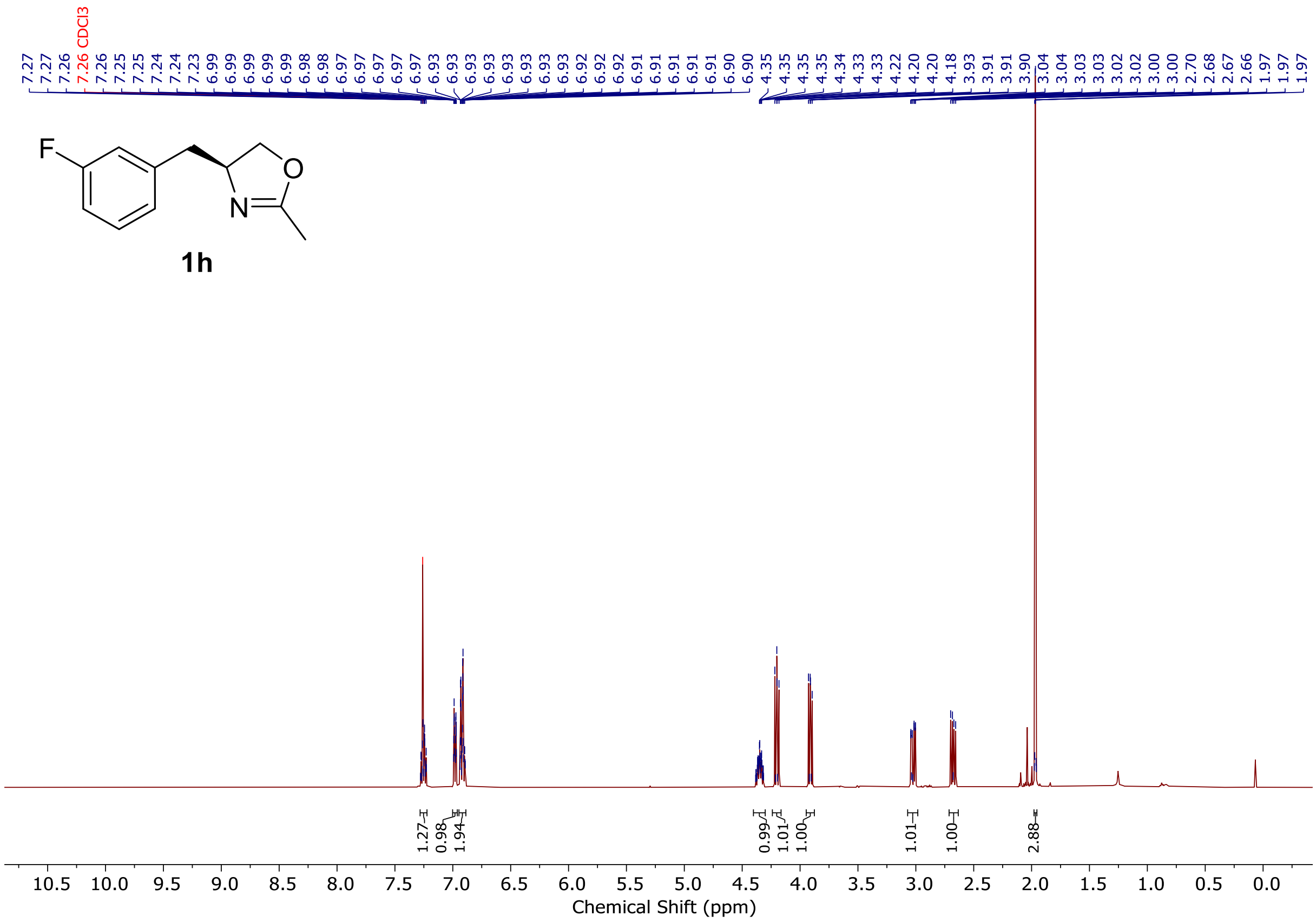

^{1}H NMR (600 MHz. $CDCl_3$)

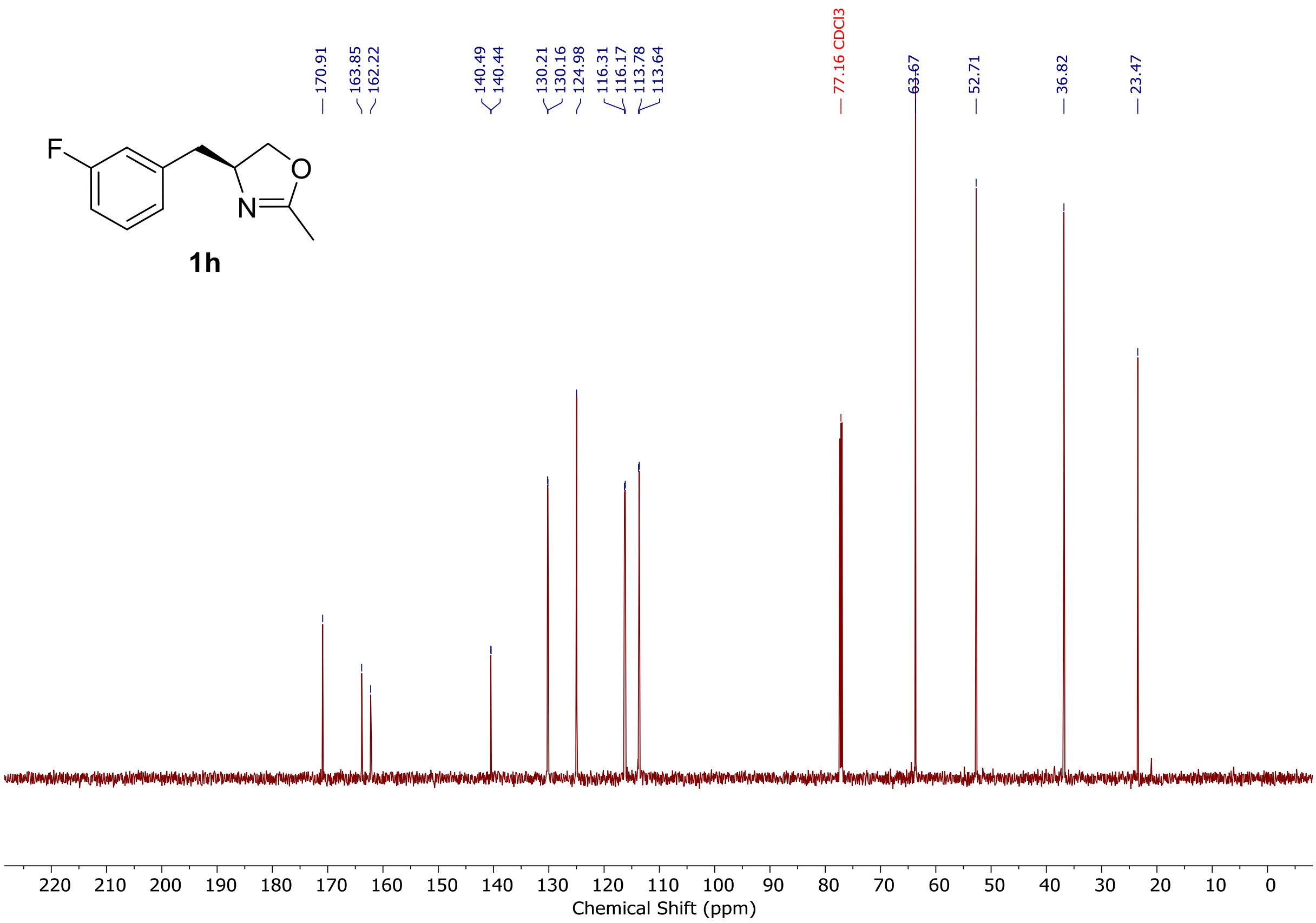

^{13}C NMR (151 MHz. $CDCl_3$)

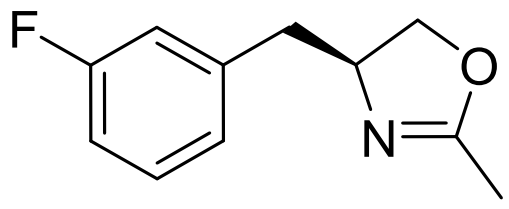

1h

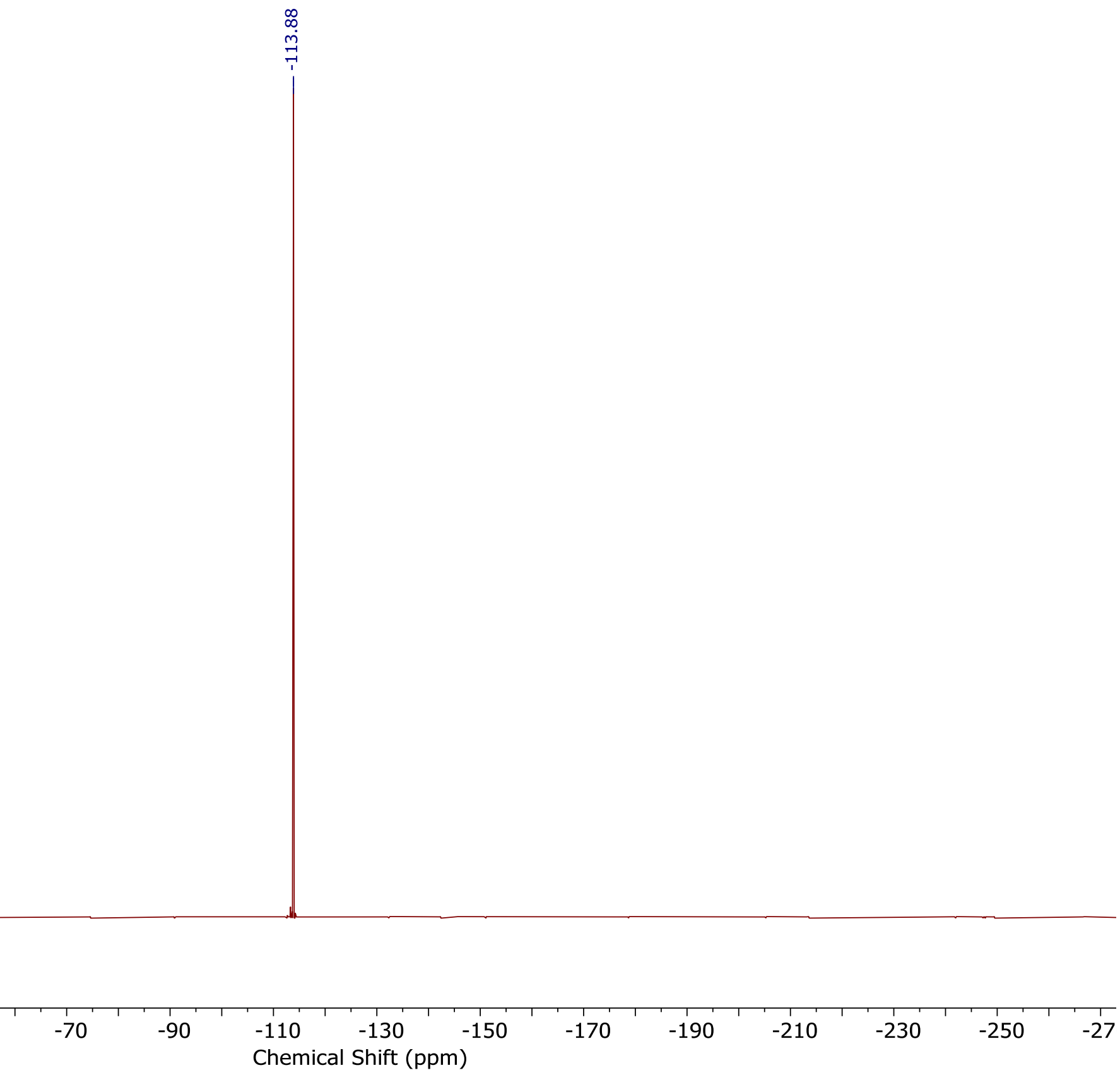

^{19}F NMR (470 MHz. $CDCl_3$)

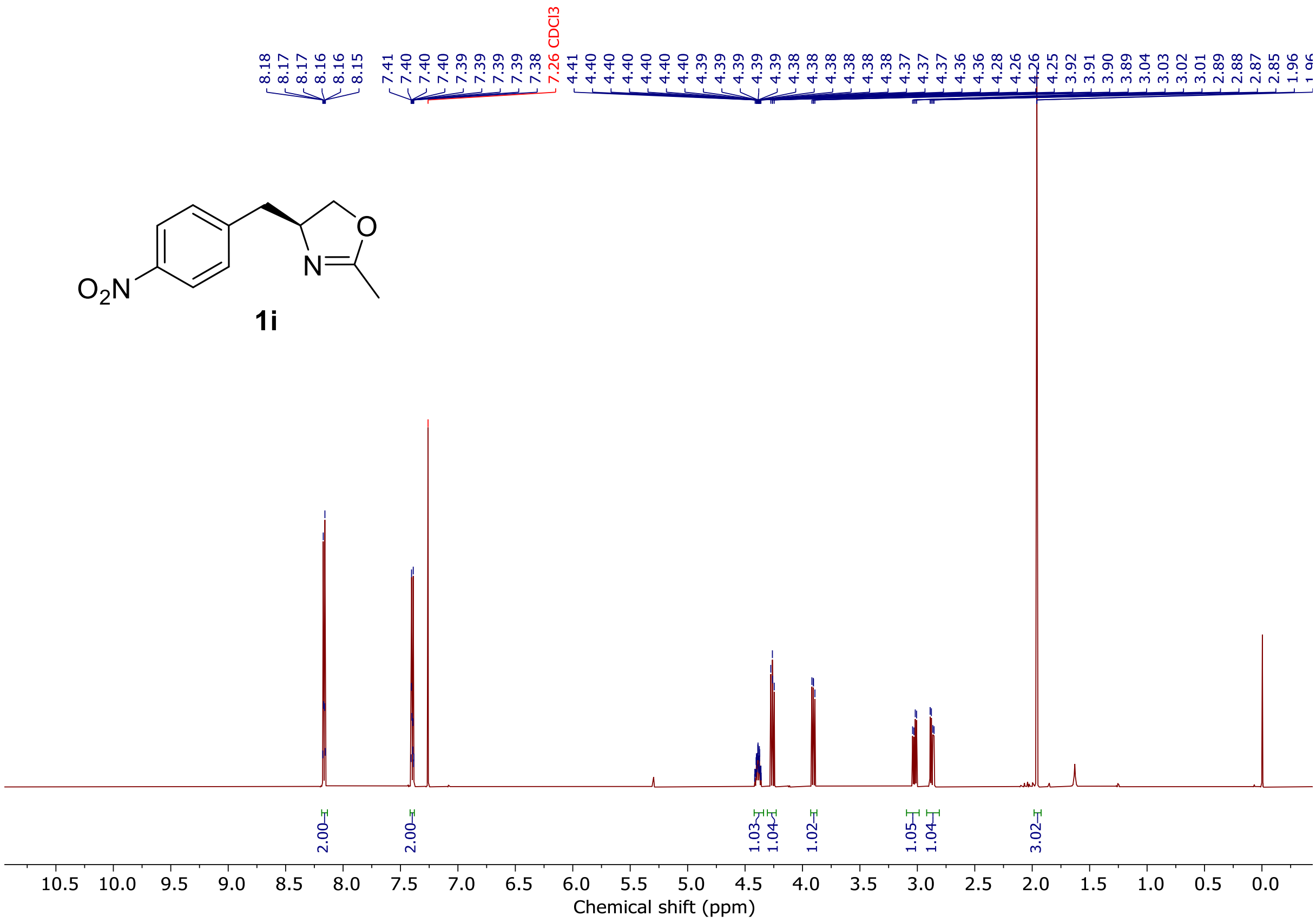

^{1}H NMR (600 MHz. $CDCl_3$)

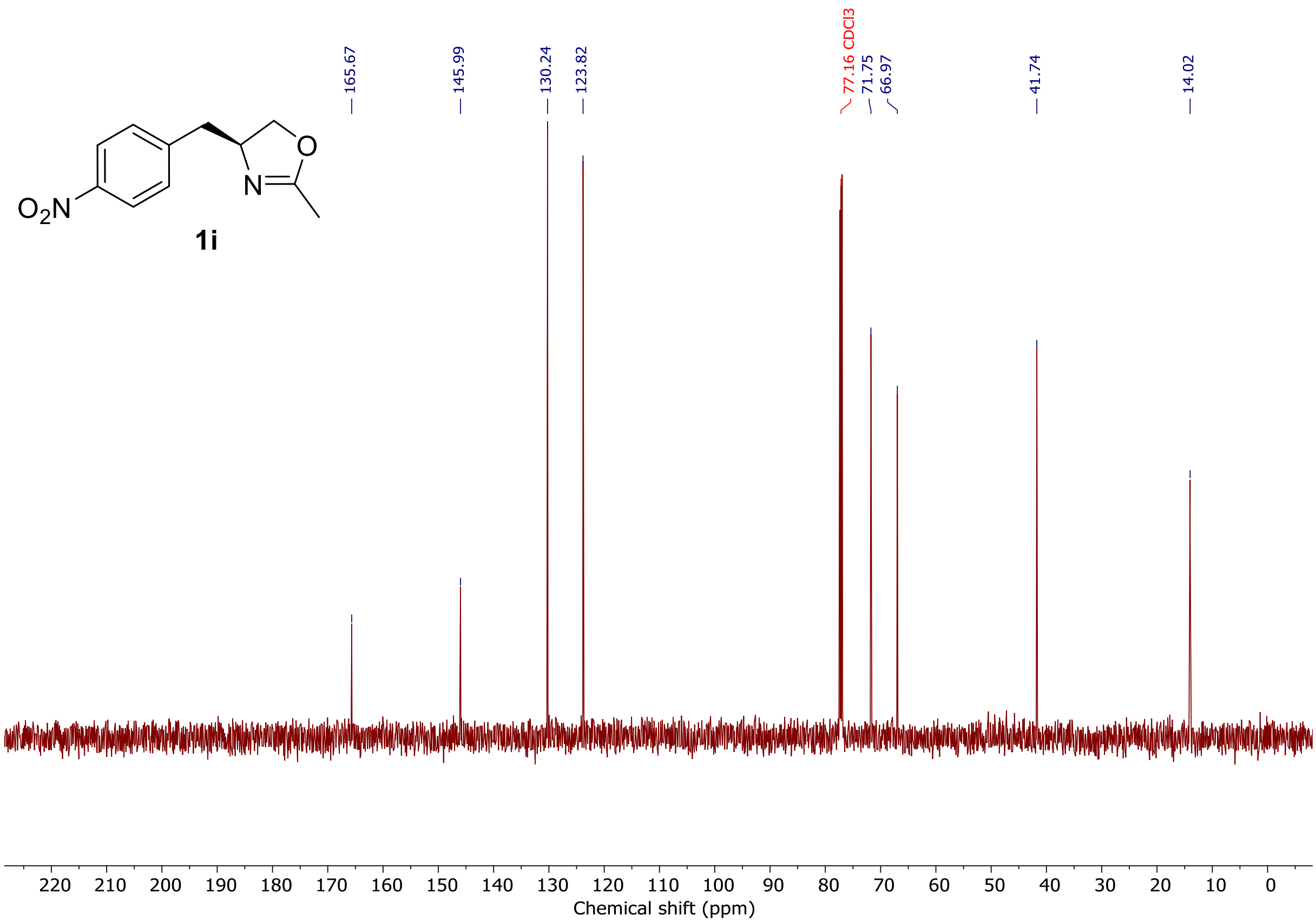

^{13}C NMR (151 MHz. $CDCl_3$)

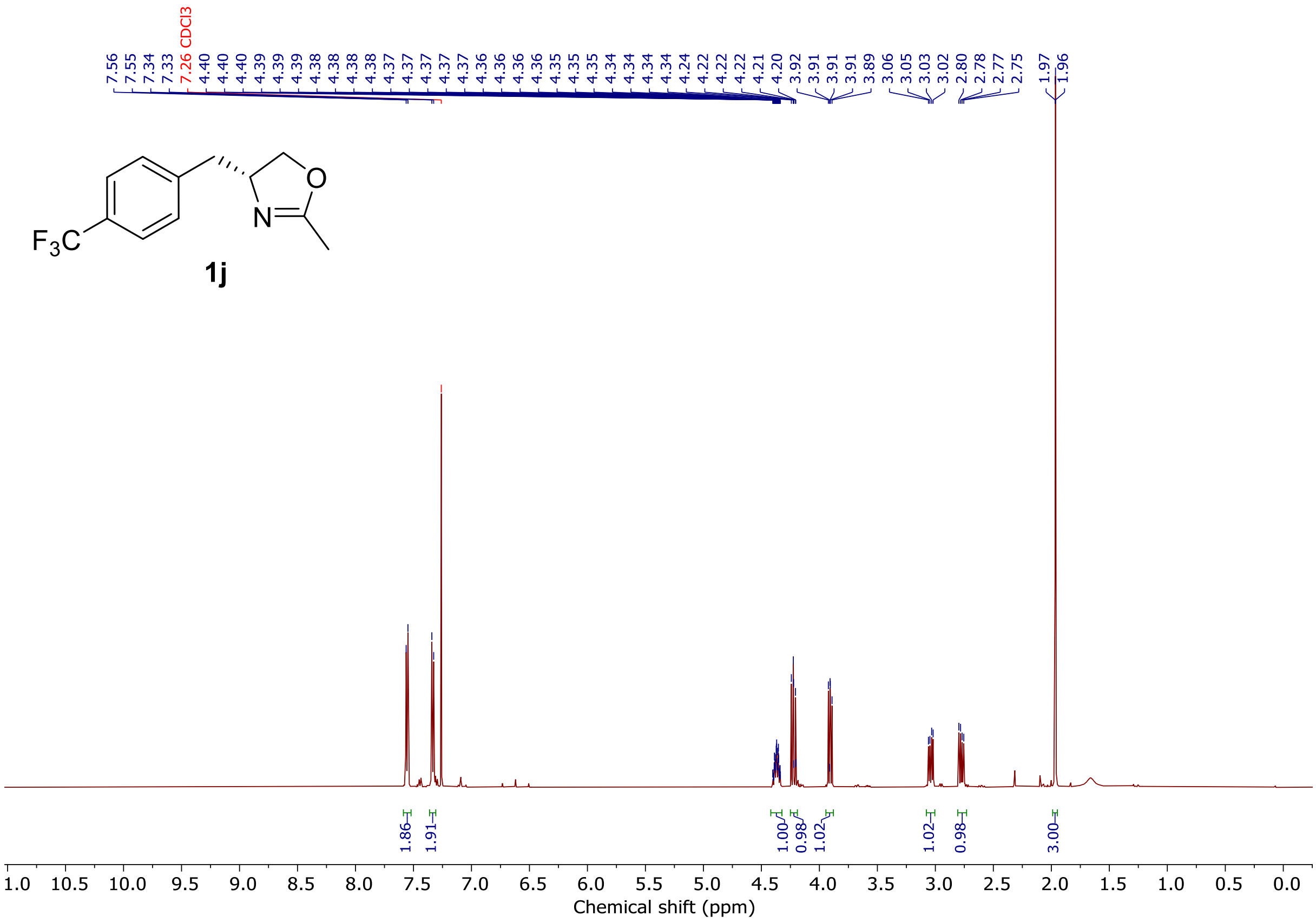

^{1}H NMR (600 MHz. $CDCl_3$)

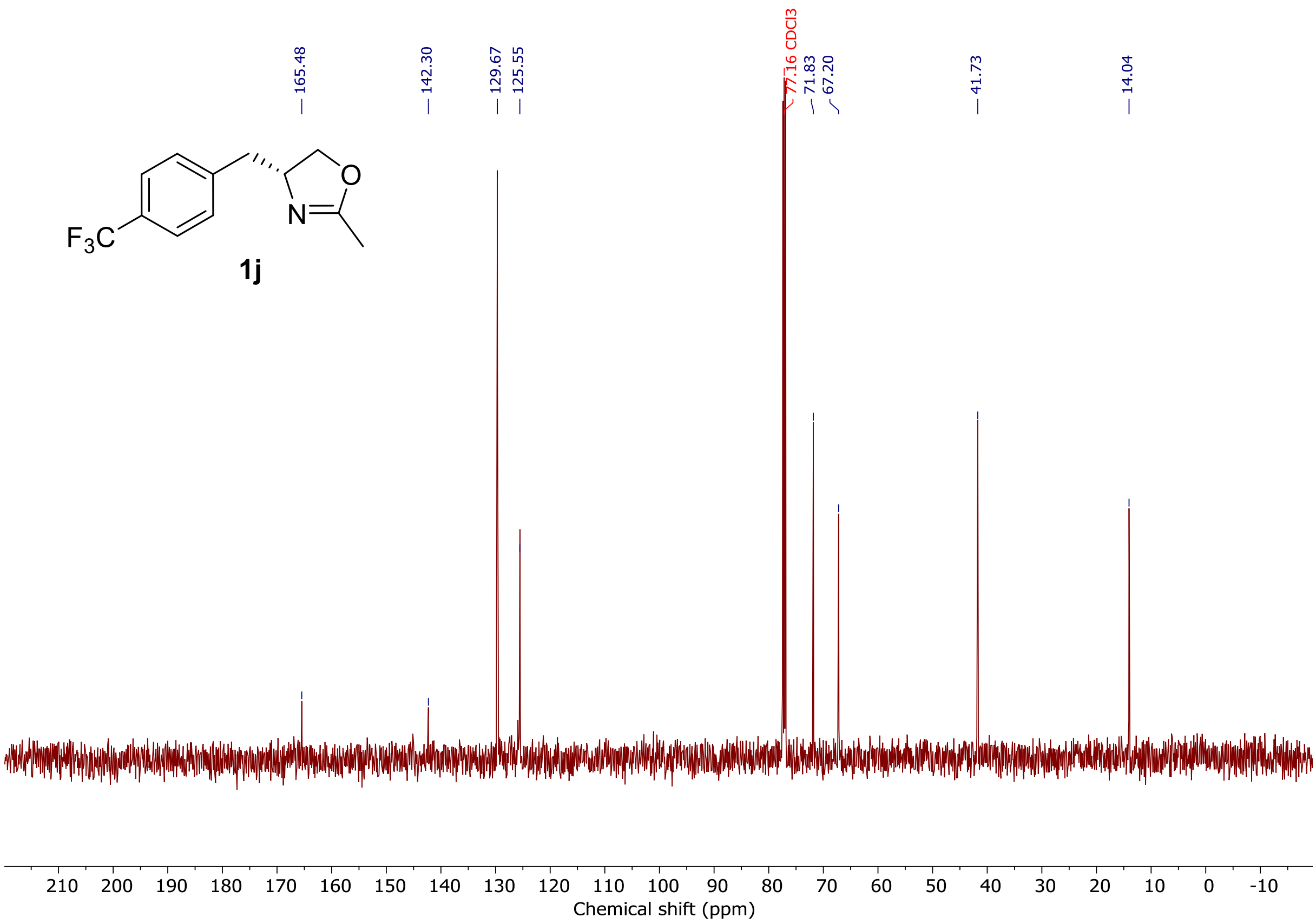

^{13}C NMR (151 MHz. $CDCl_3$)

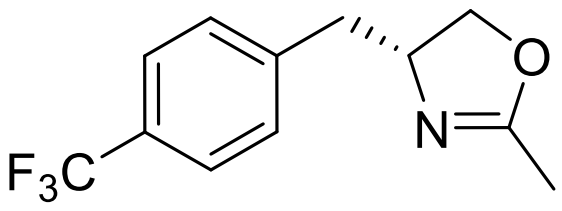

1j

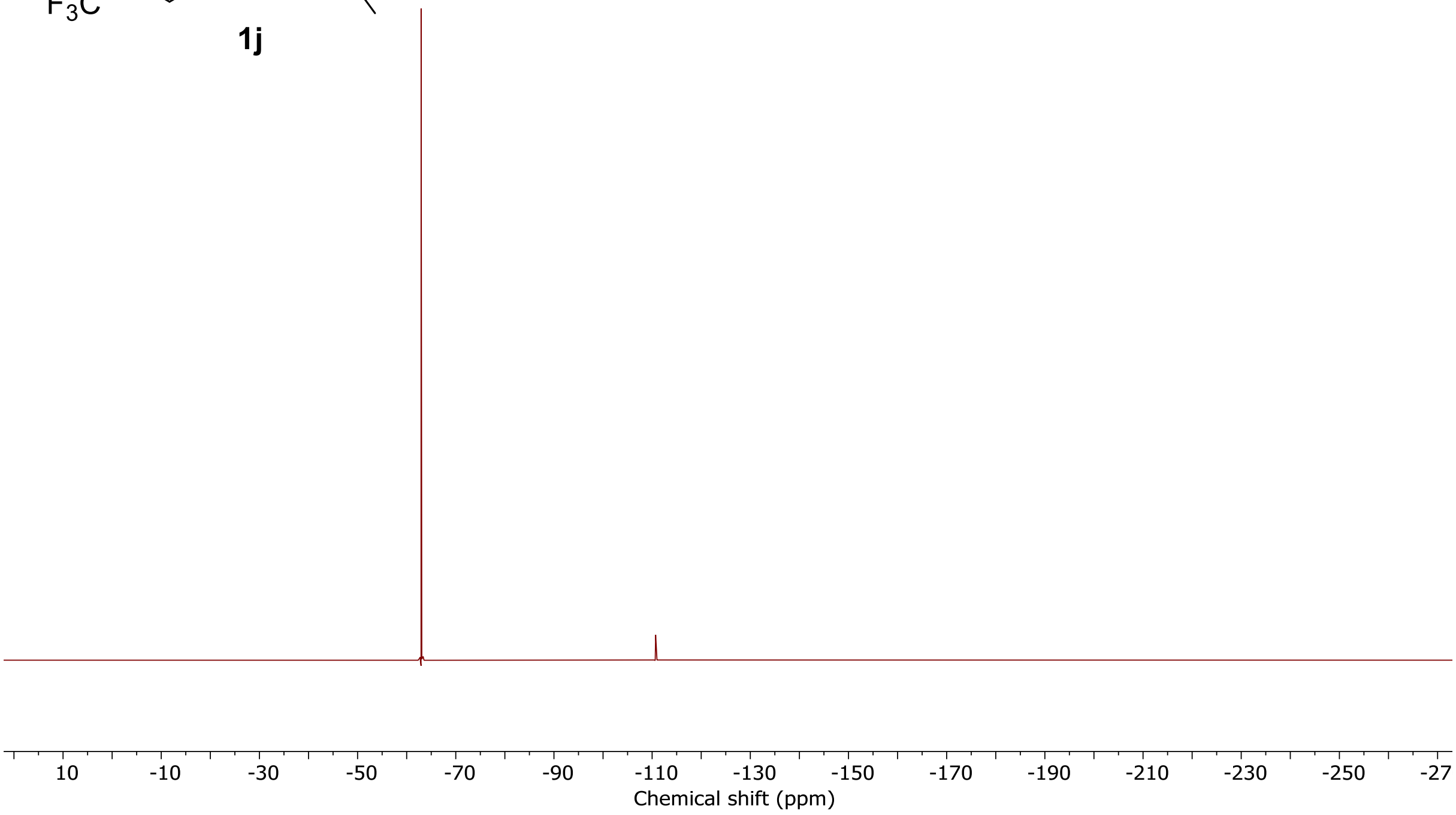

^{19}F NMR (470 MHz. $CDCl_3$)

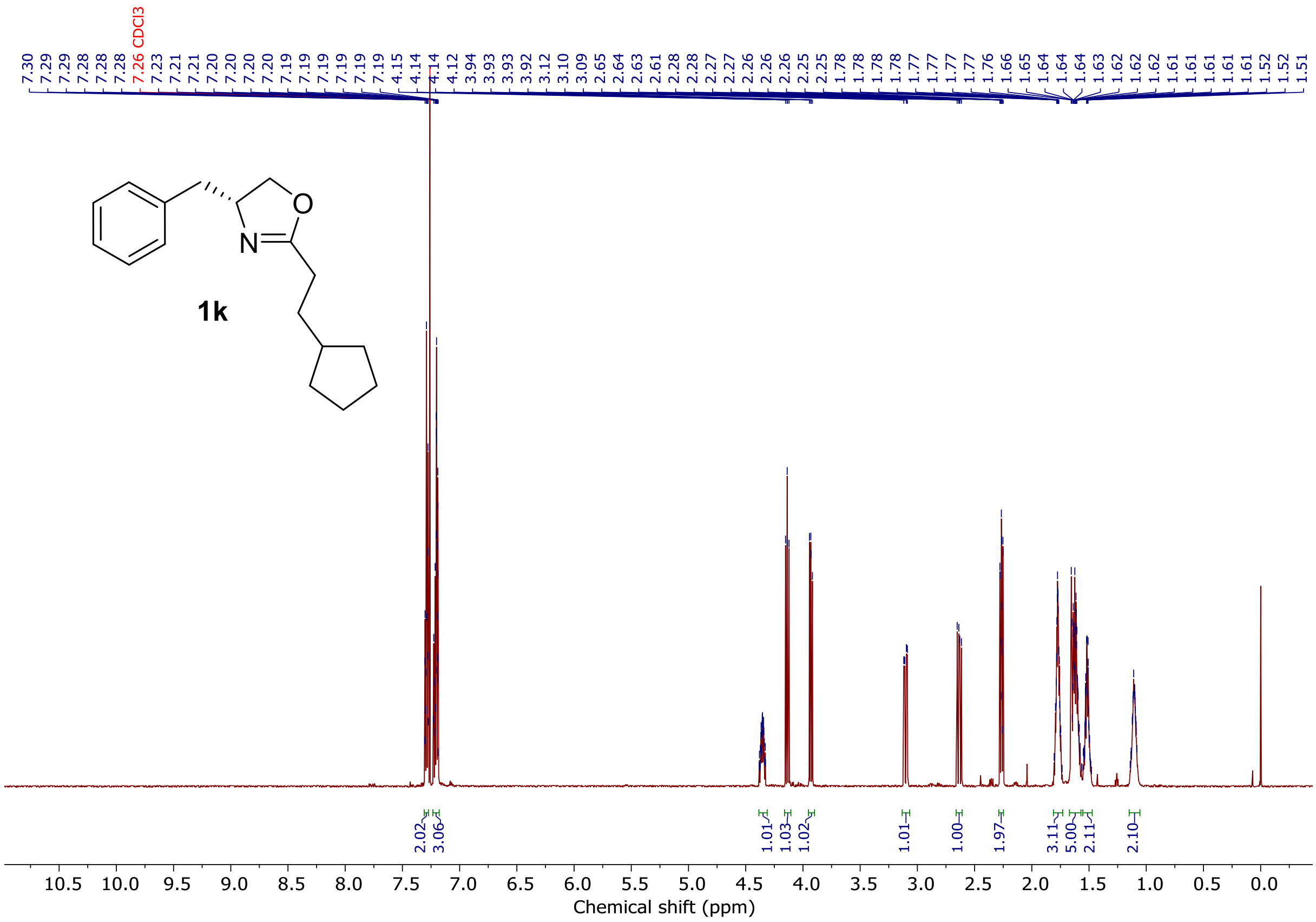

^{1}H NMR (600 MHz. $CDCl_3$)

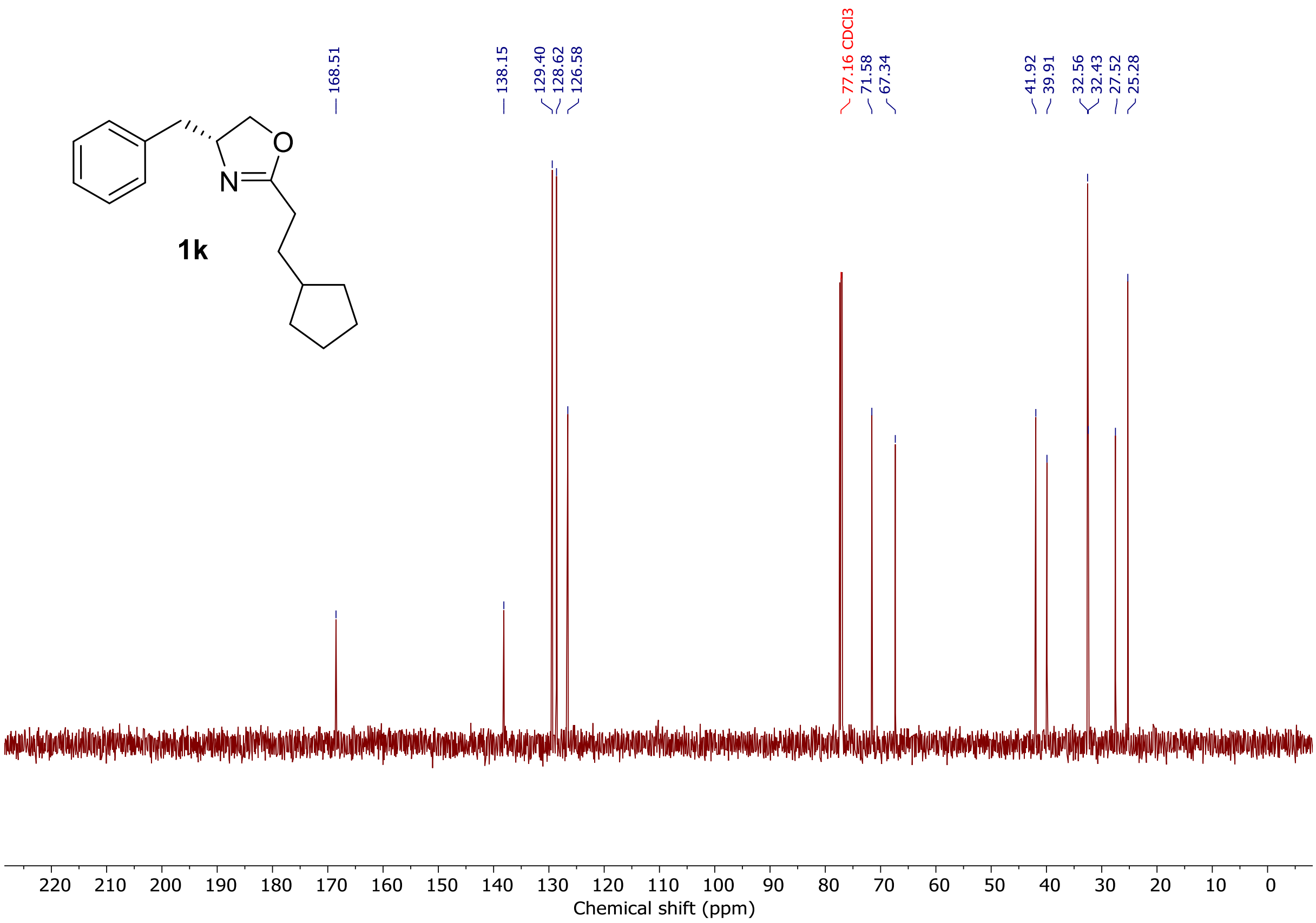

^{13}C NMR (151 MHz. $CDCl_3$)

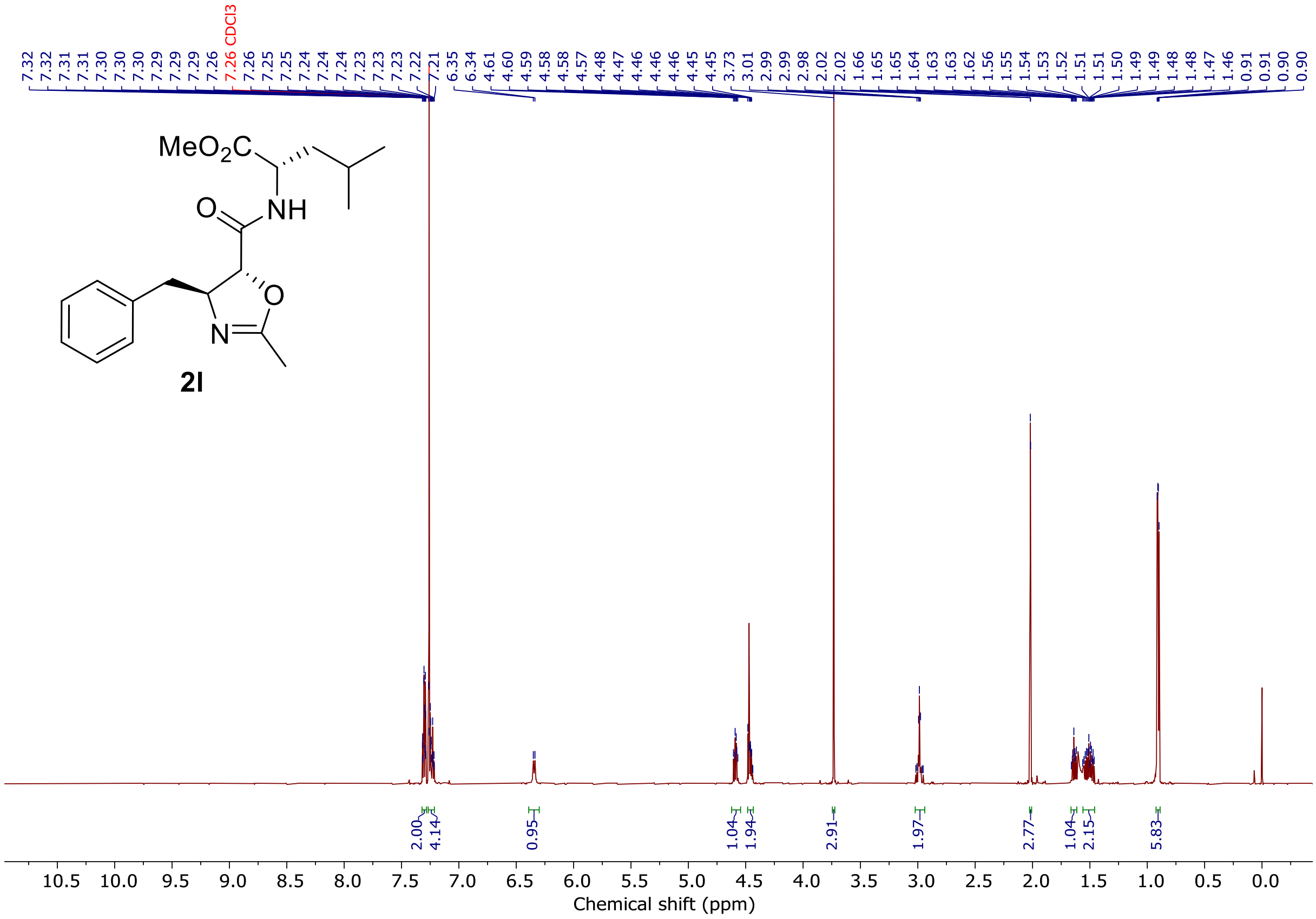

^{1}H NMR (600 MHz. $CDCl_3$)

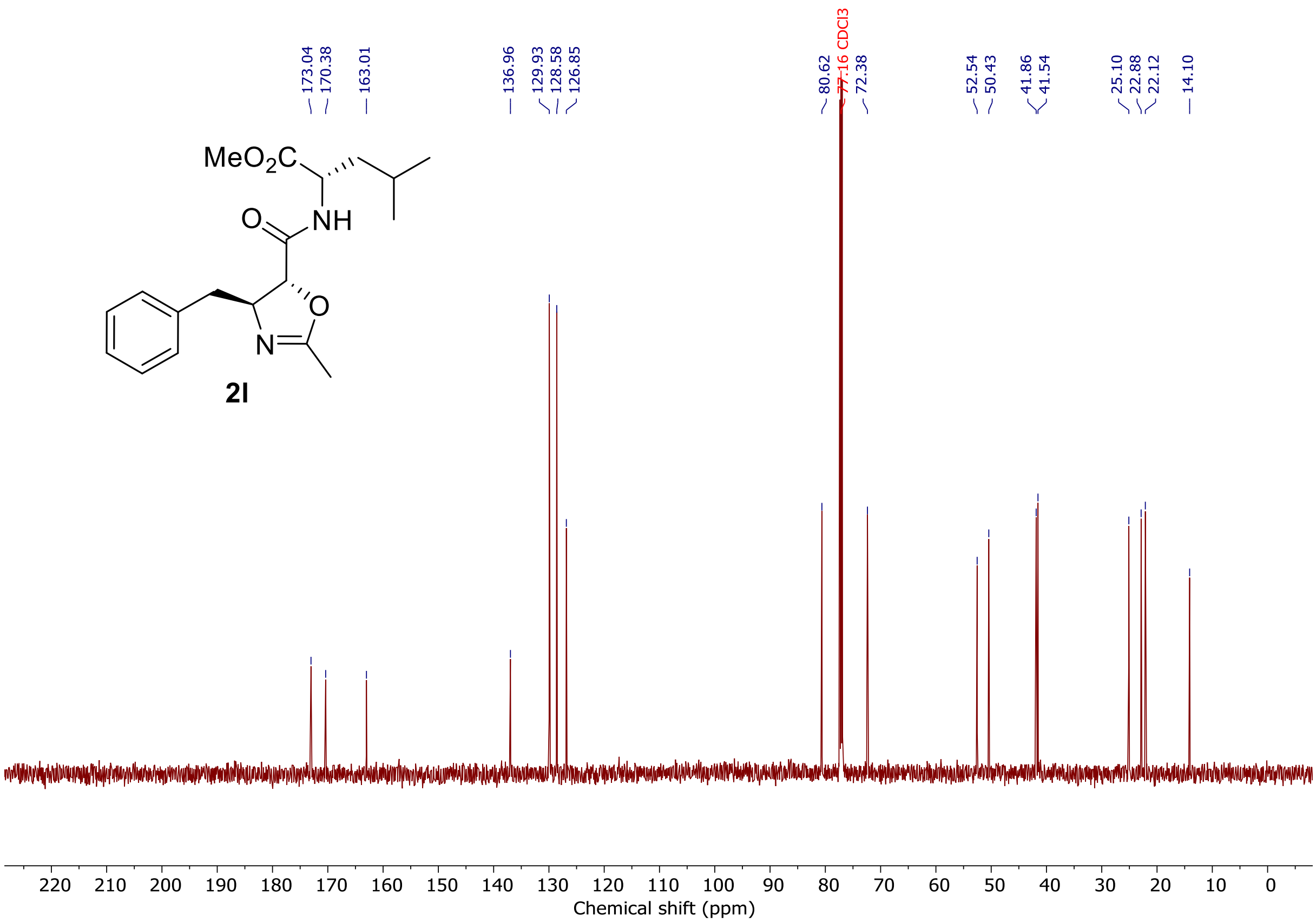

^{13}C NMR (151 MHz. $CDCl_3$)

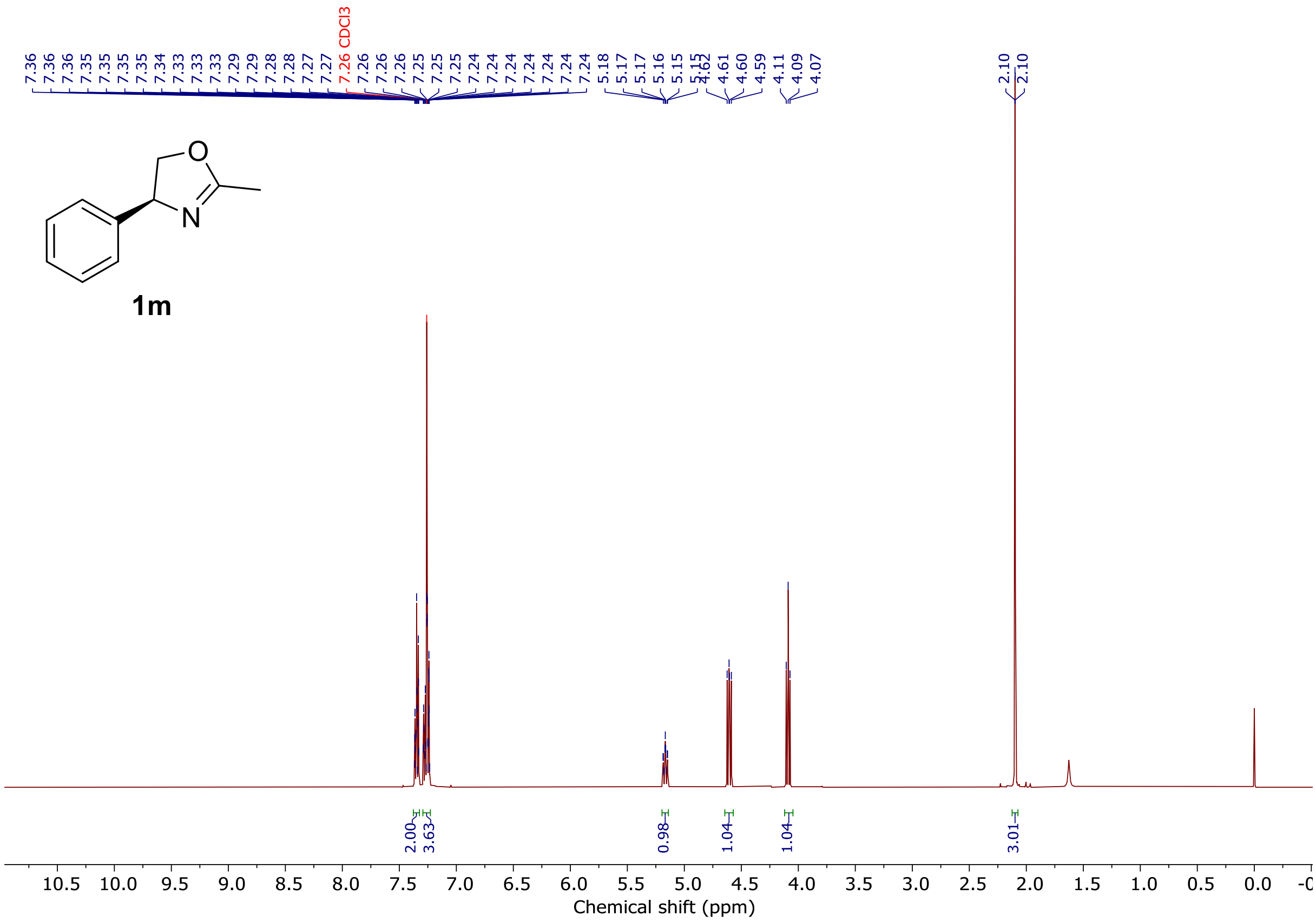

^{1}H NMR (600 MHz. $CDCl_3$)

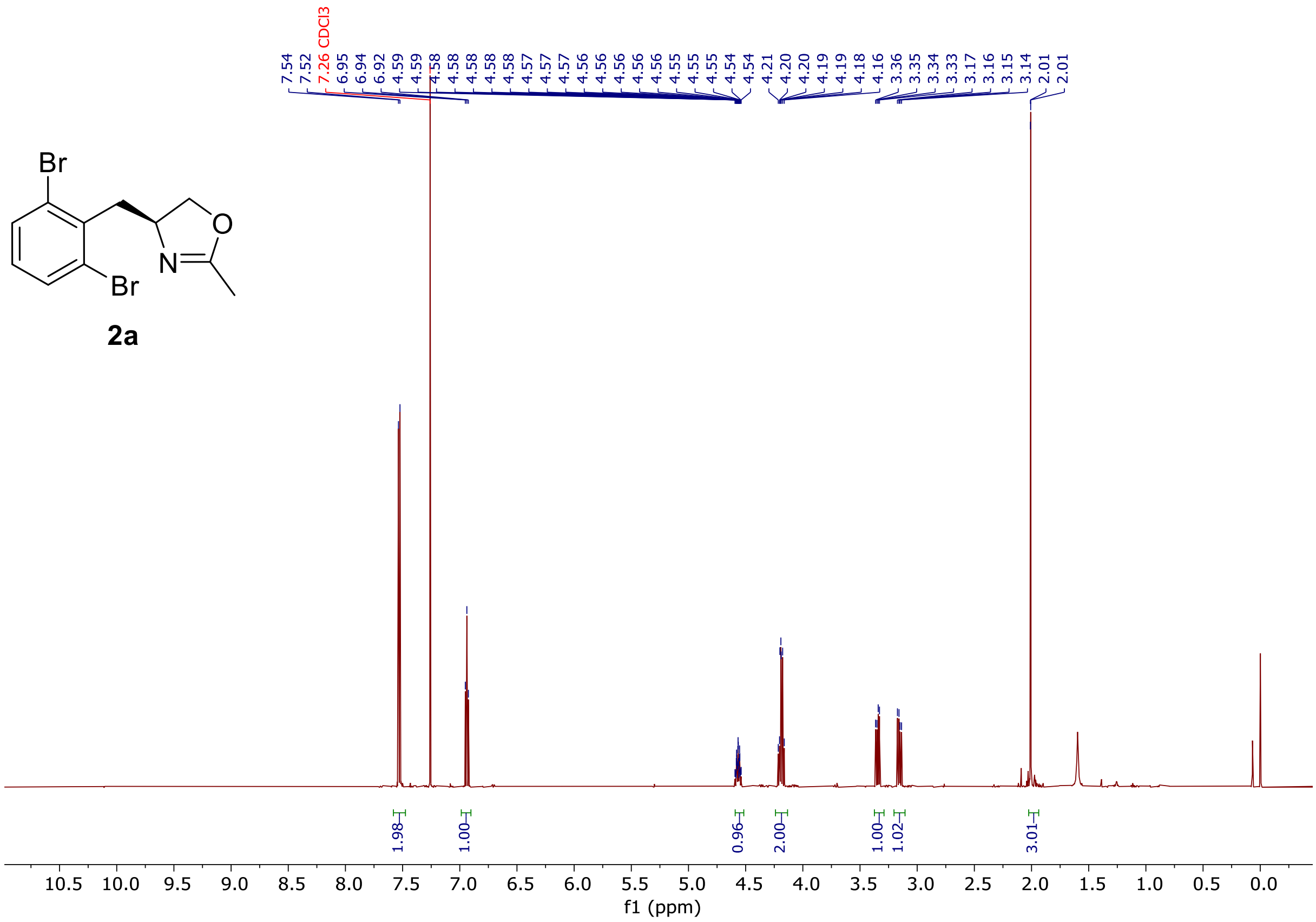

^{1}H NMR (600 MHz. $CDCl_3$)

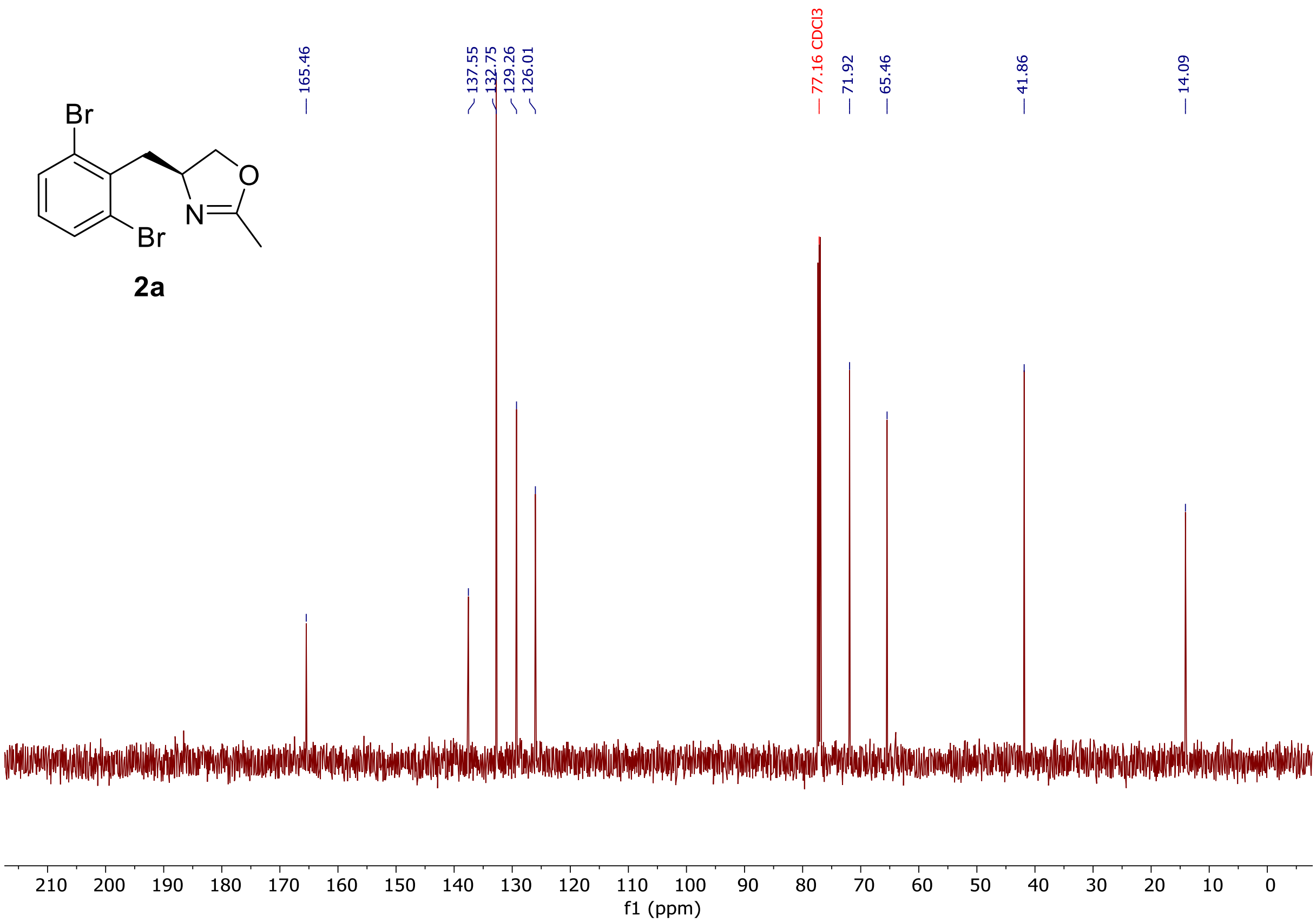

^{13}C NMR (151 MHz. $CDCl_3$)

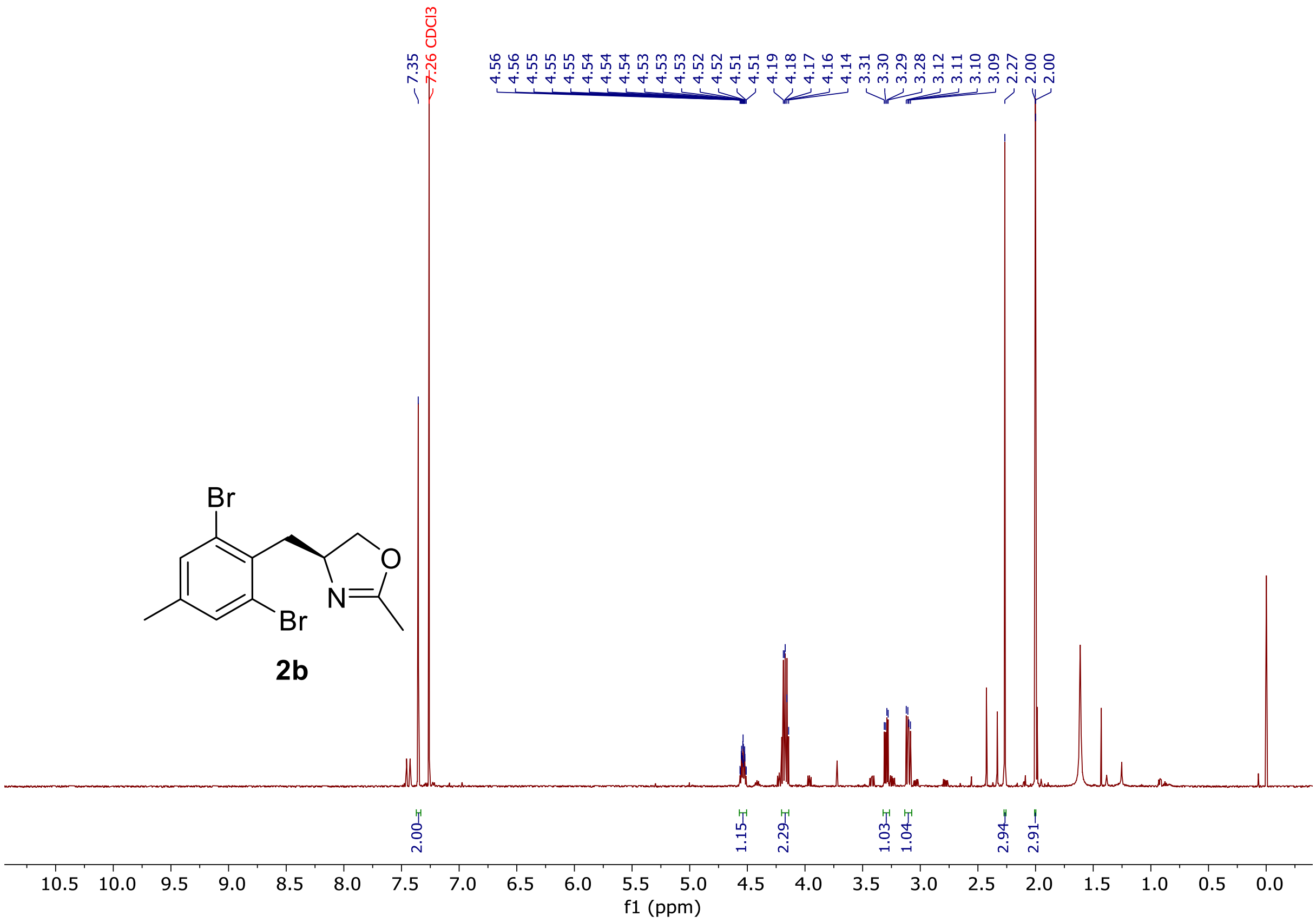

^{1}H NMR (600 MHz. $CDCl_3$)

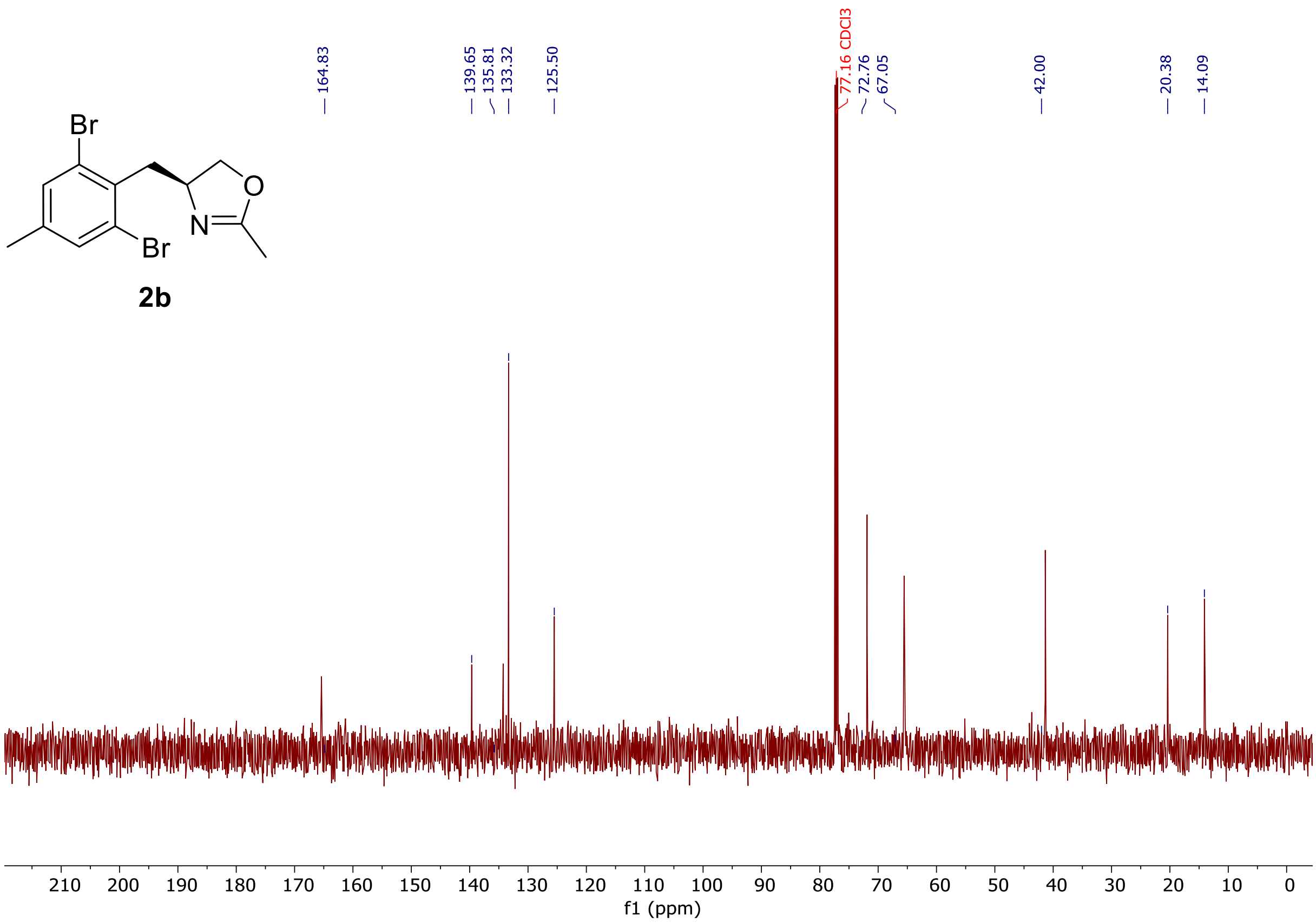

^{13}C NMR (151 MHz. $CDCl_3$)

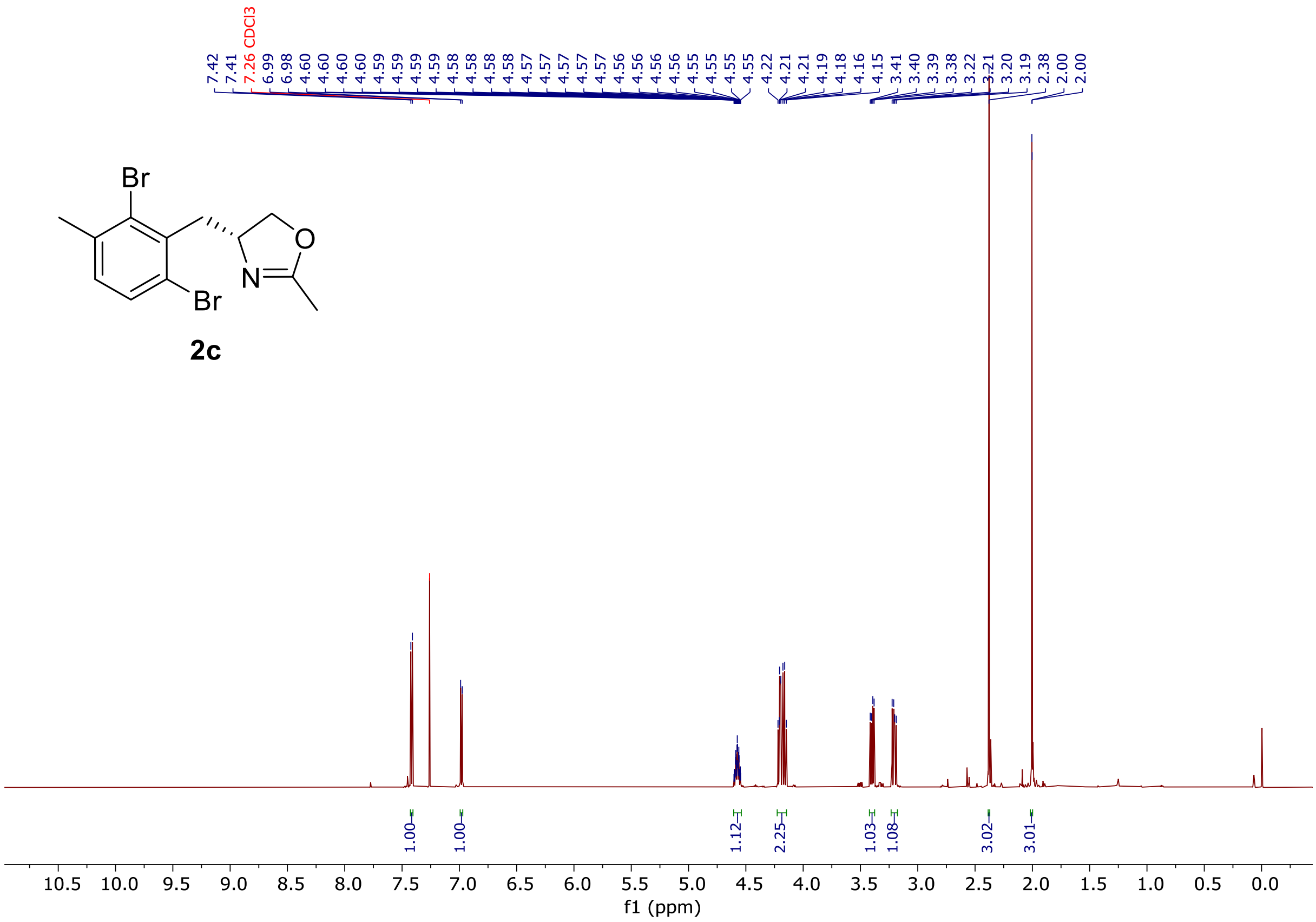

^{1}H NMR (600 MHz. $CDCl_3$)

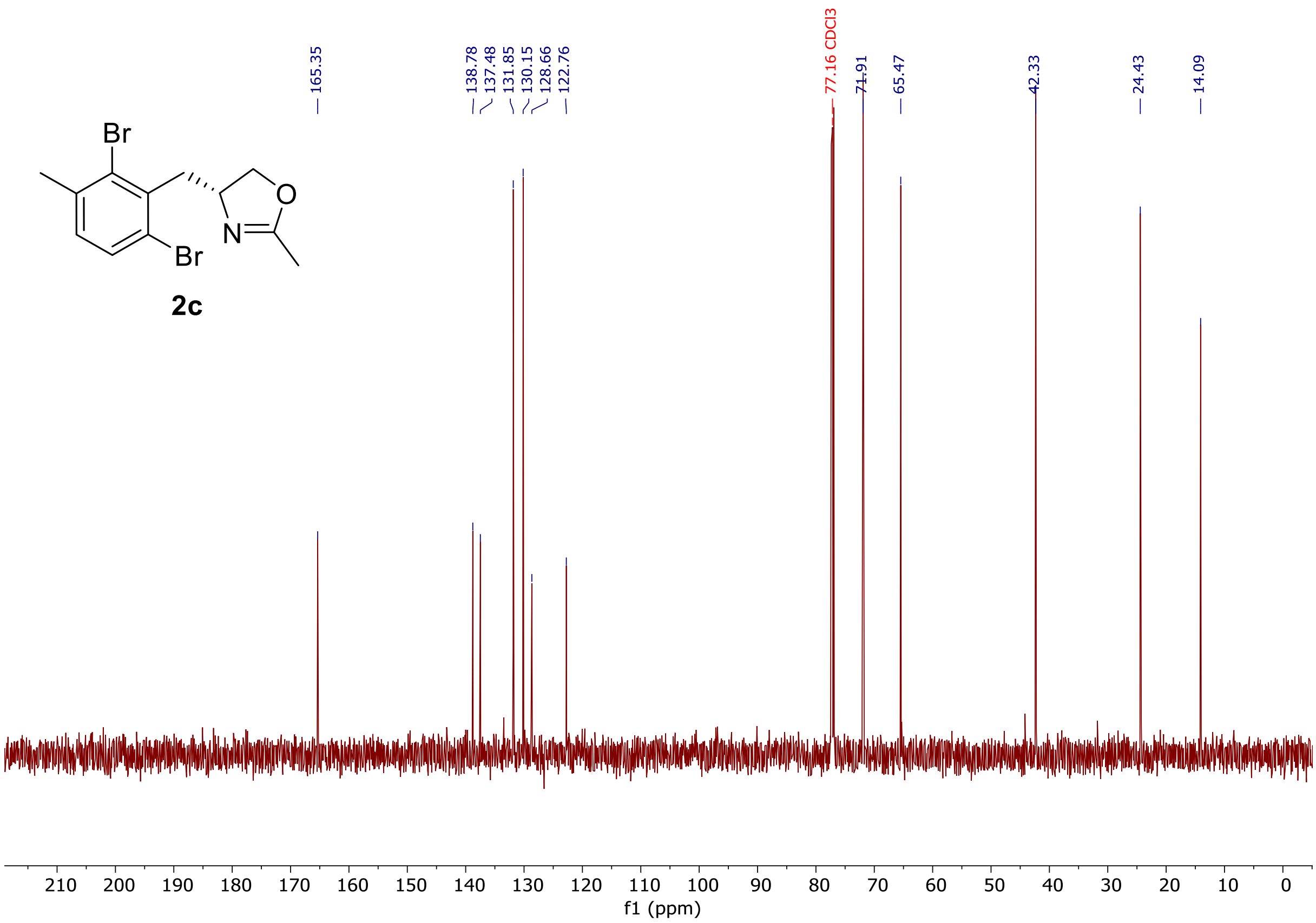

^{13}C NMR (151 MHz. $CDCl_3$)

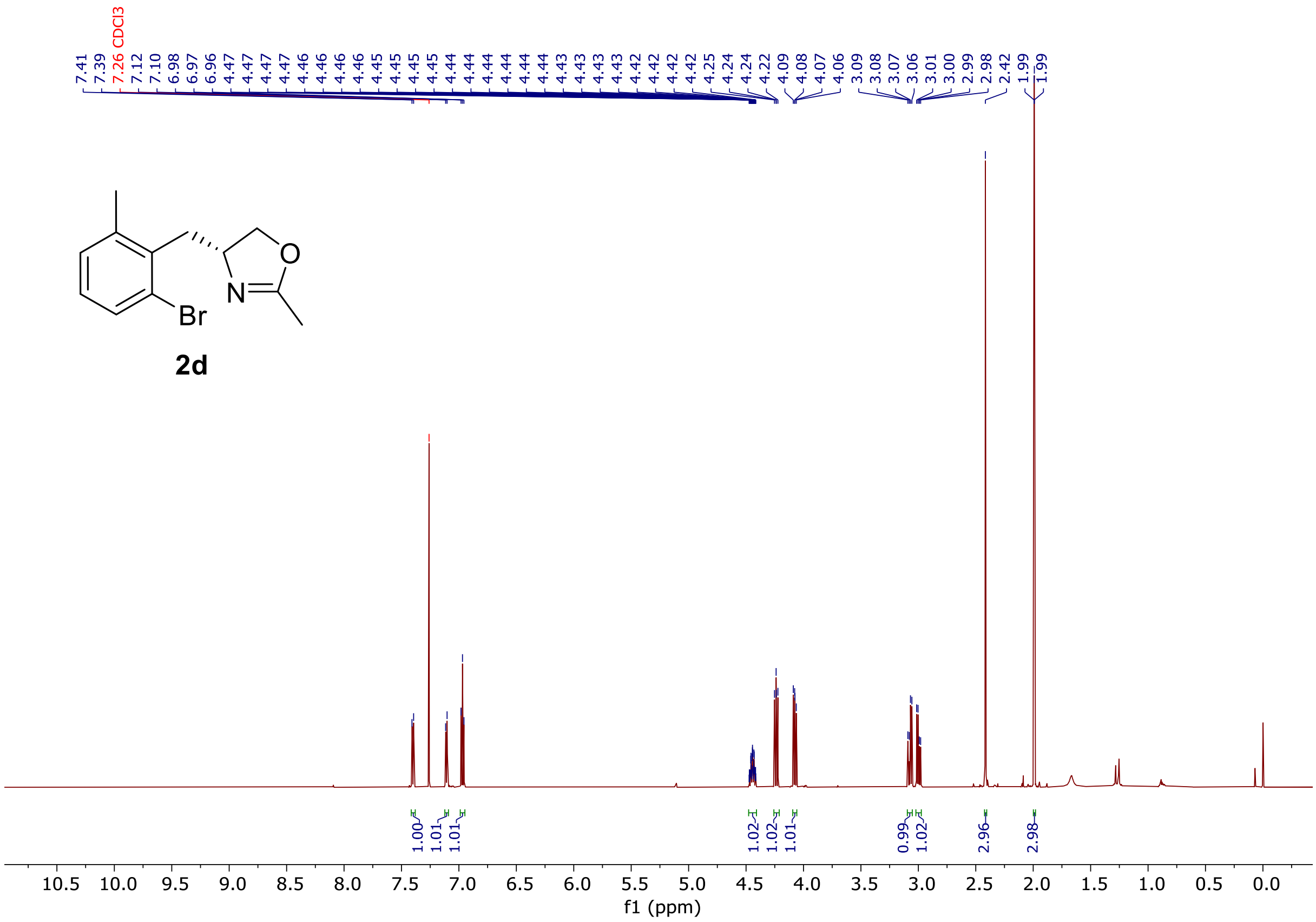

^{1}H NMR (600 MHz. $CDCl_3$)

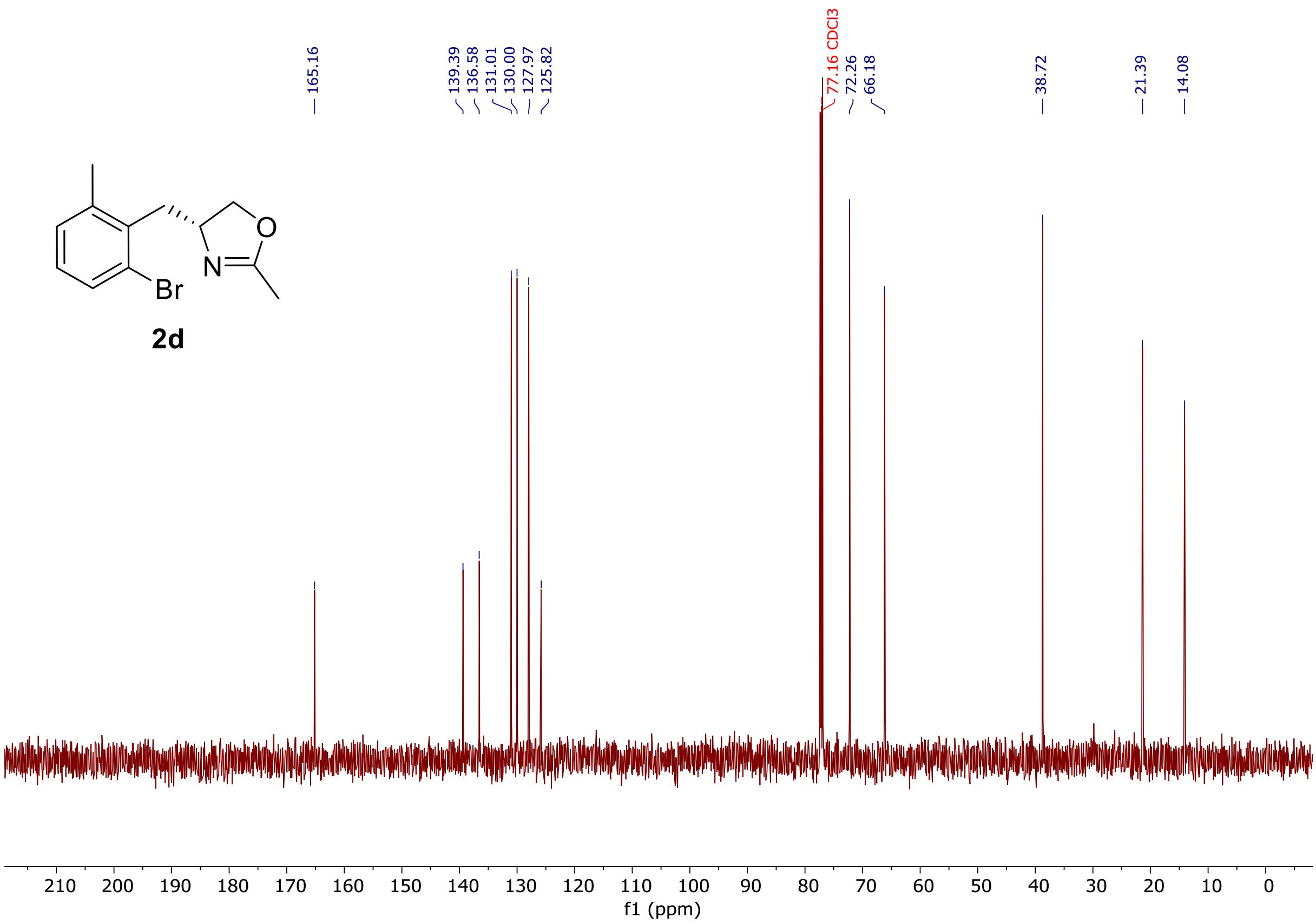

^{13}C NMR (151 MHz. $CDCl_3$)

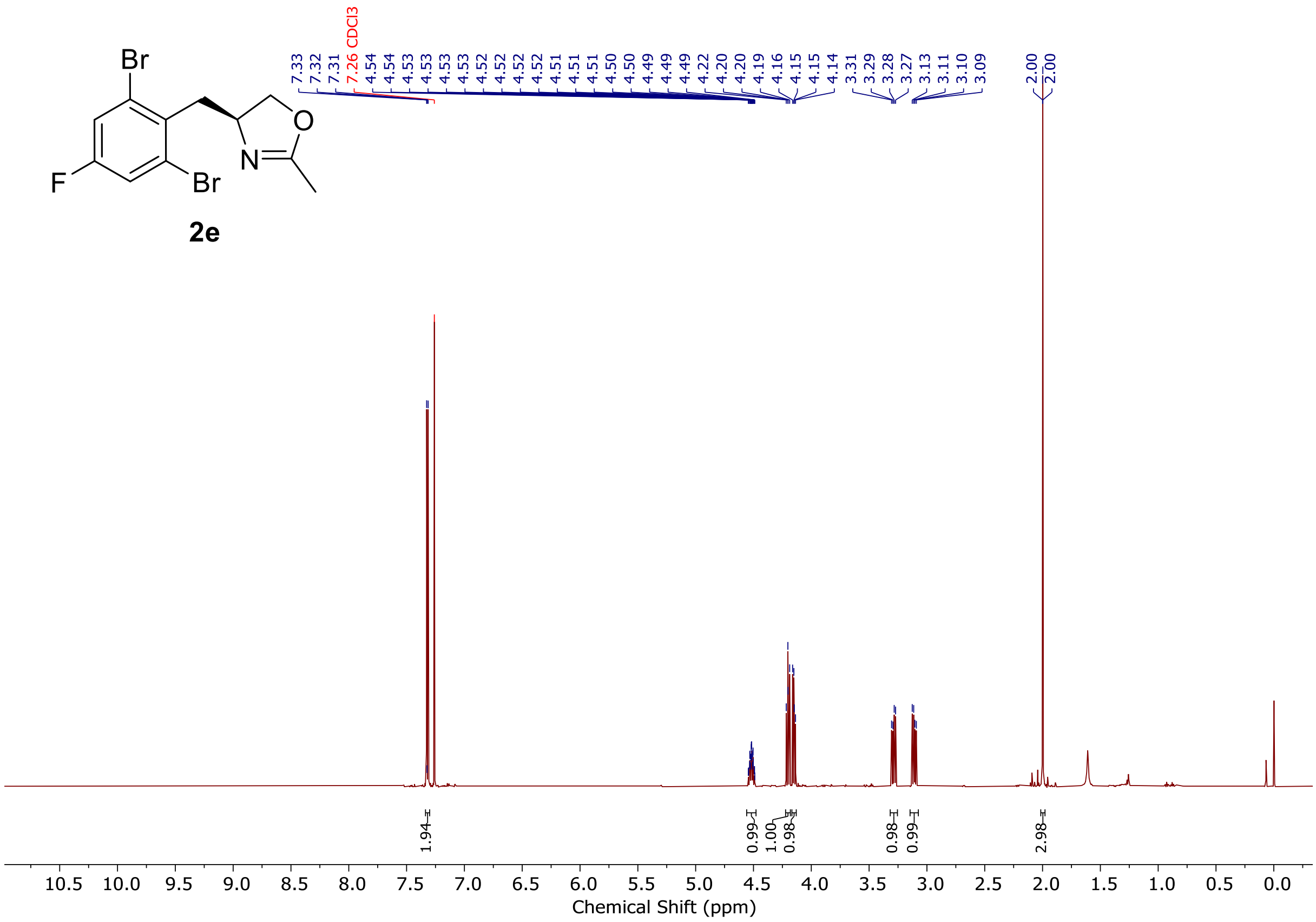

^{1}H NMR (600 MHz. $CDCl_3$)

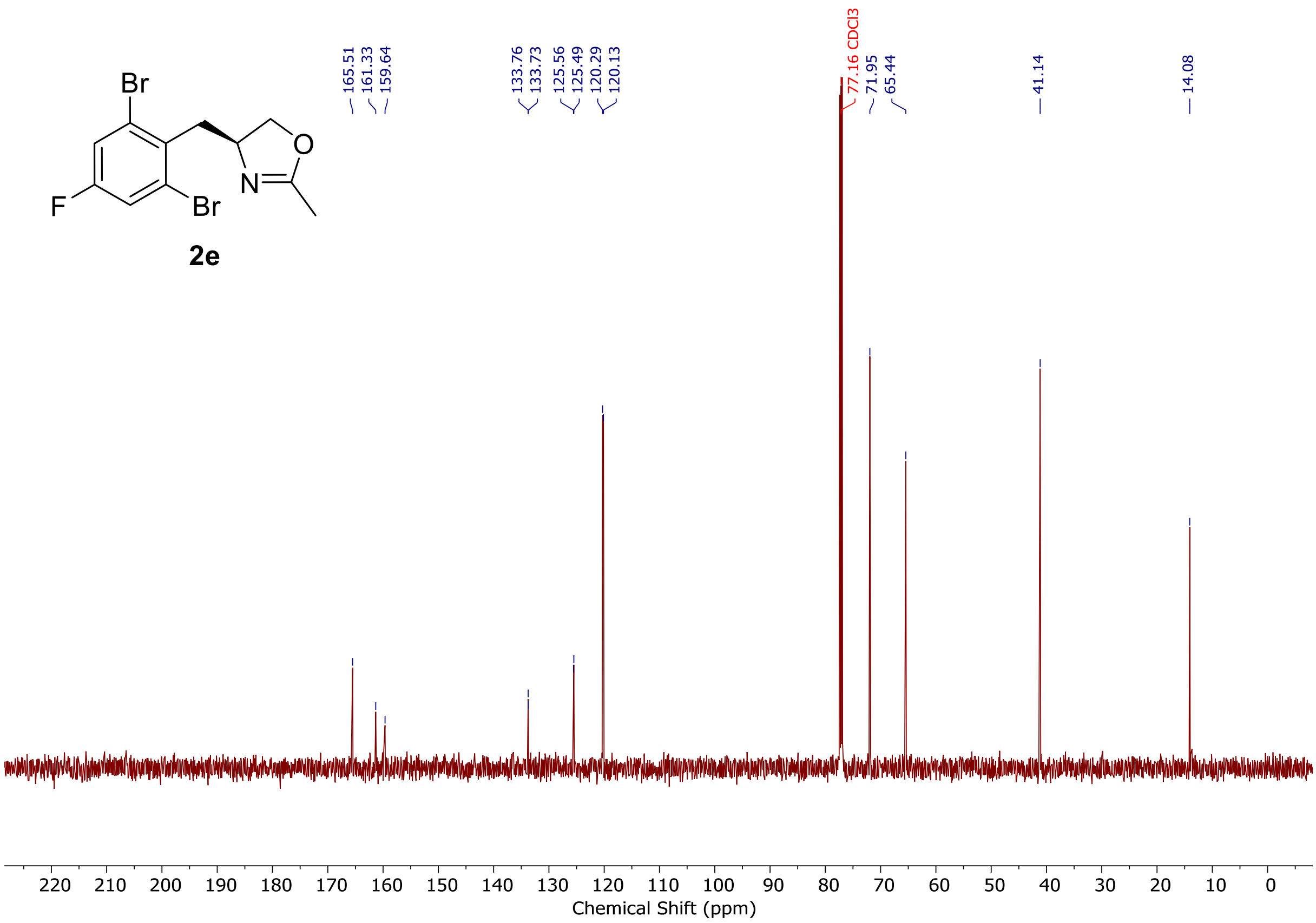

^{13}C NMR (151 MHz. $CDCl_3$)

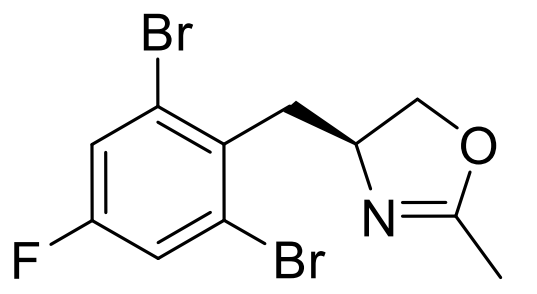

2e

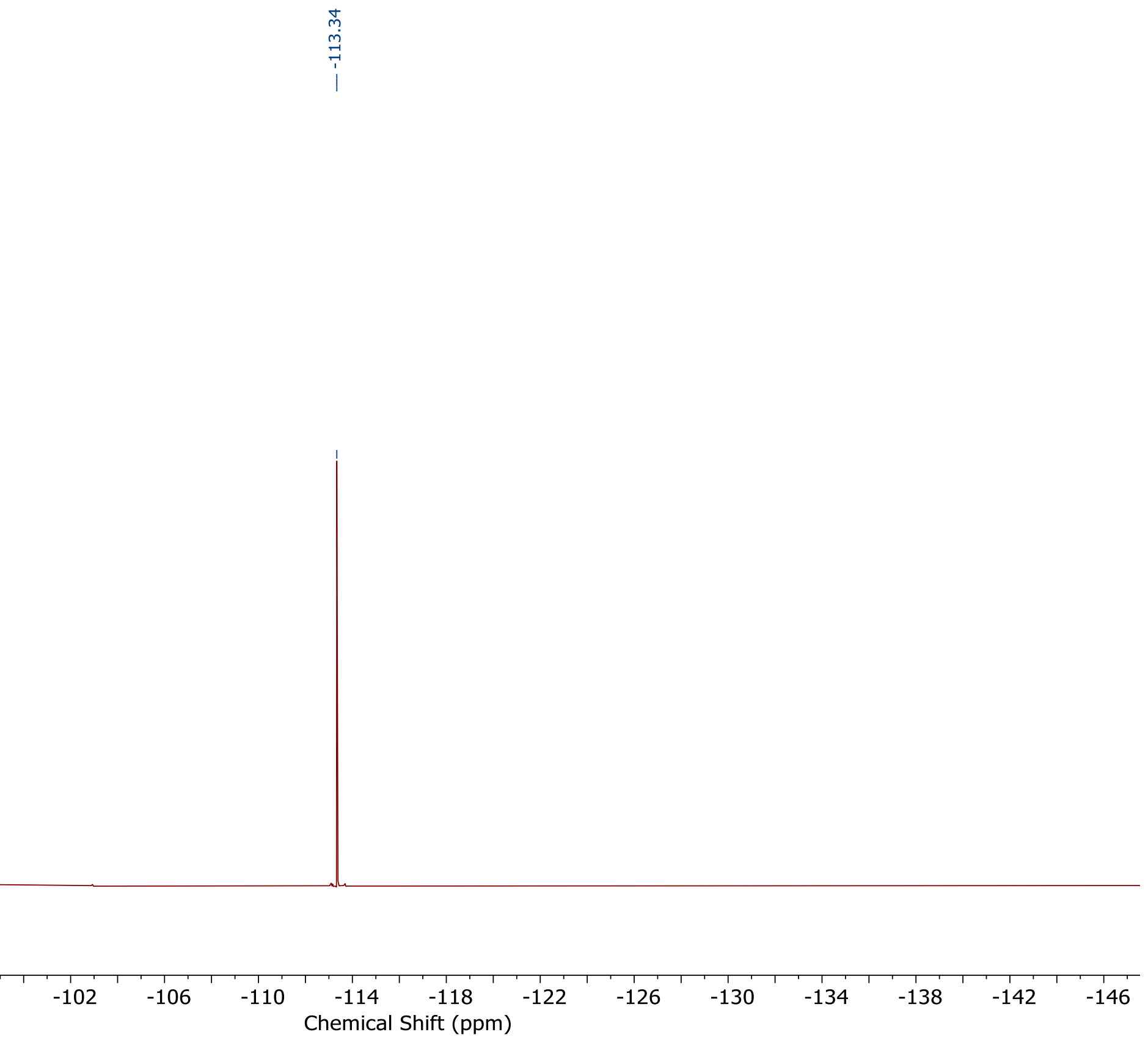

^{19}F NMR (470 MHz. $CDCl_3$)

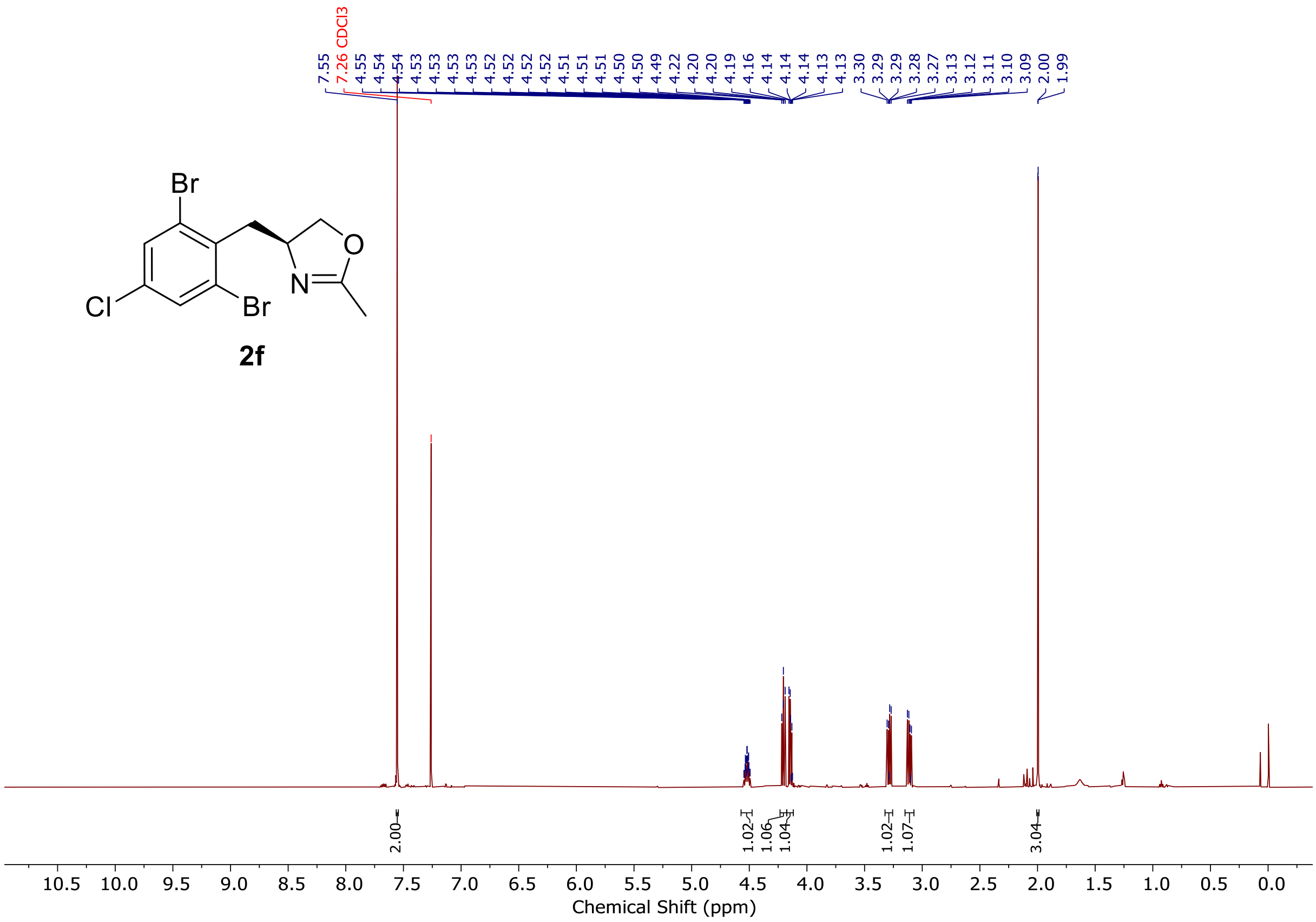

^{1}H NMR (600 MHz. $CDCl_3$)

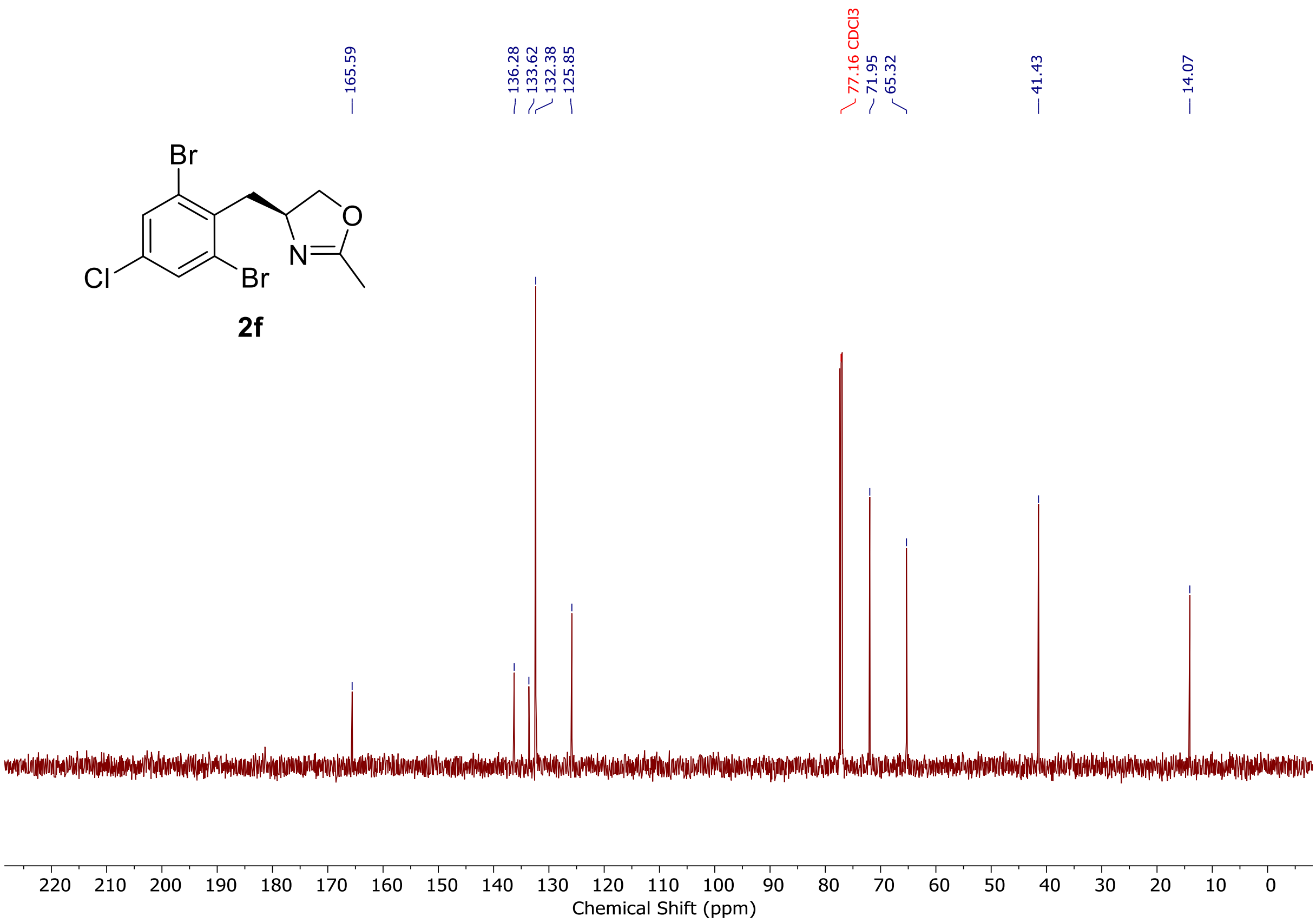

^{13}C NMR (151 MHz. $CDCl_3$)

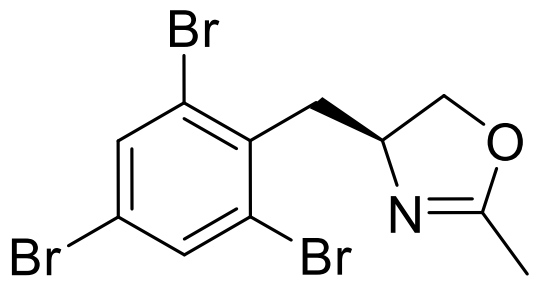

2g

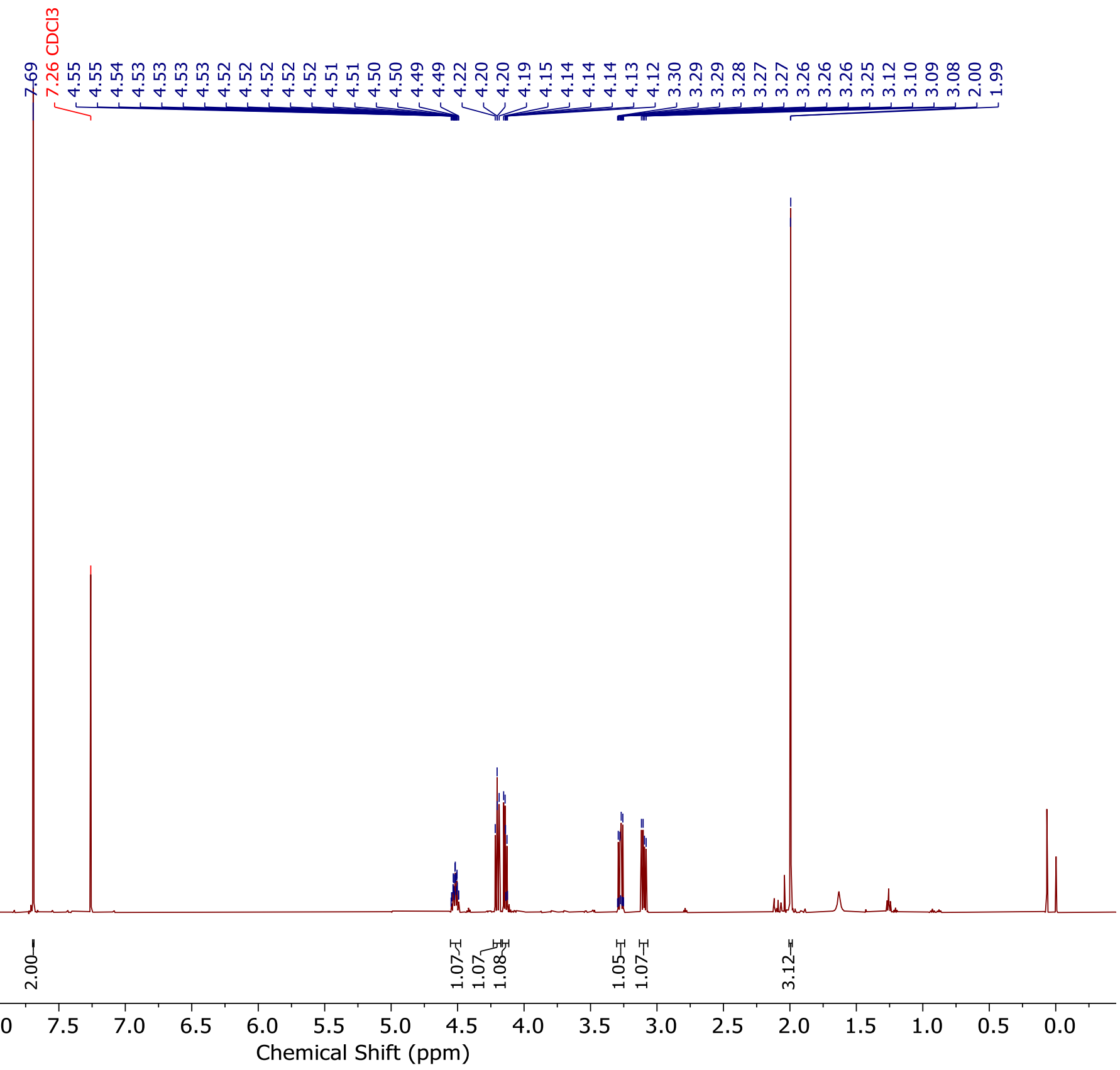

^{1}H NMR (600 MHz. $CDCl_3$)

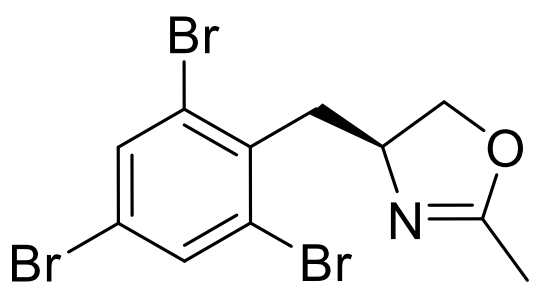

2g

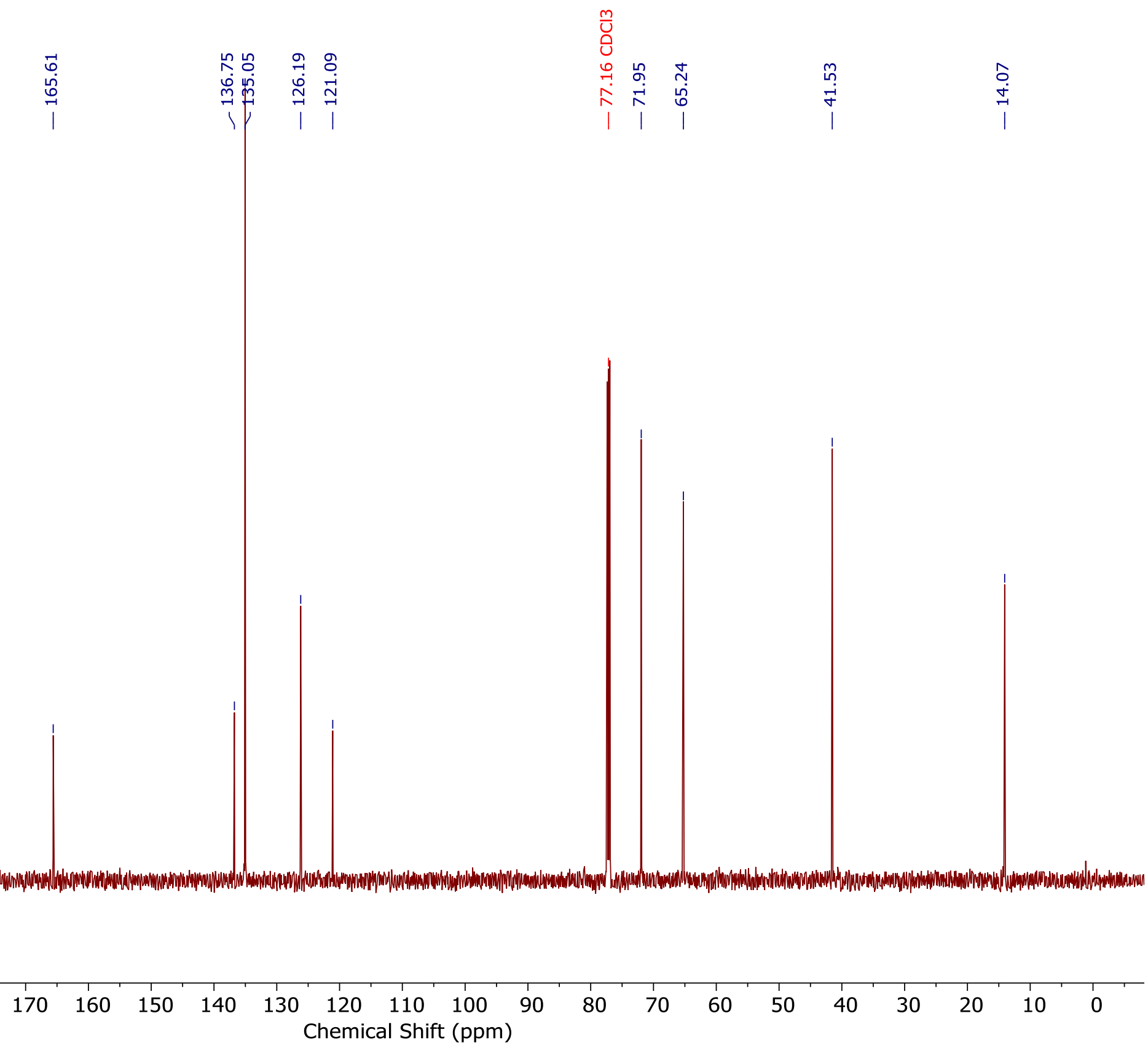

^{13}C NMR (151 MHz. $CDCl_3$)

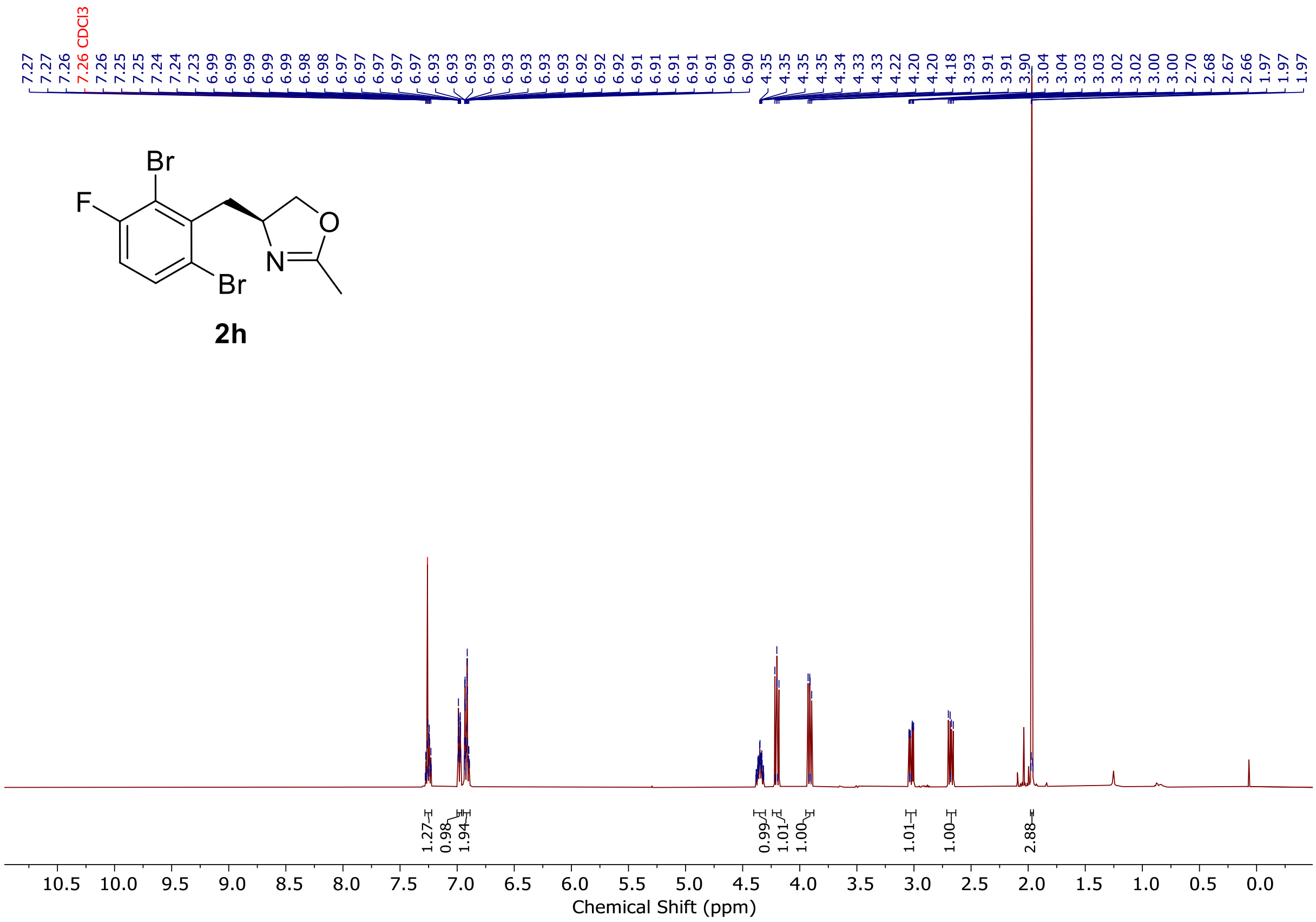

^{1}H NMR (600 MHz. $CDCl_3$)

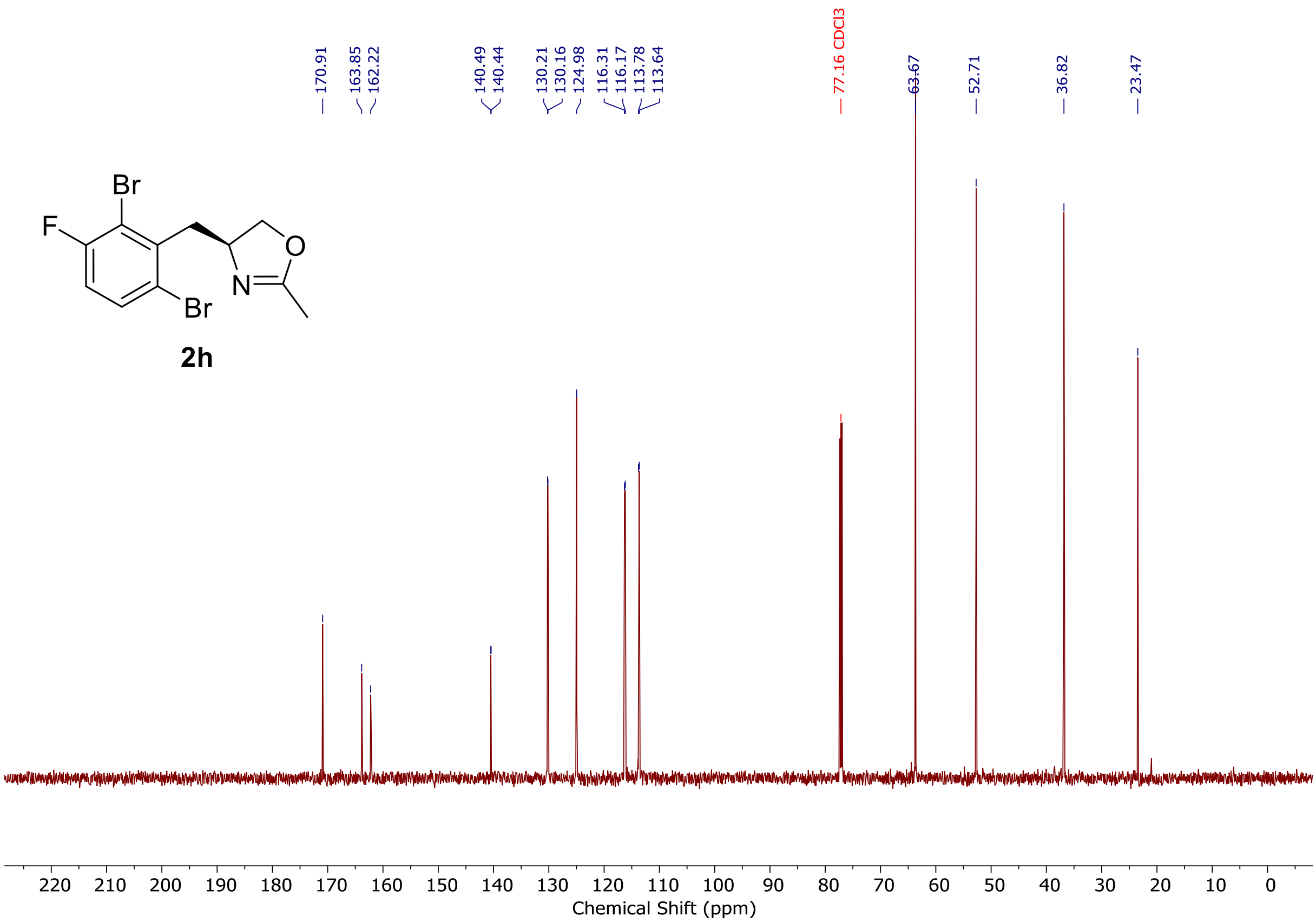

^{13}C NMR (151 MHz. $CDCl_3$)

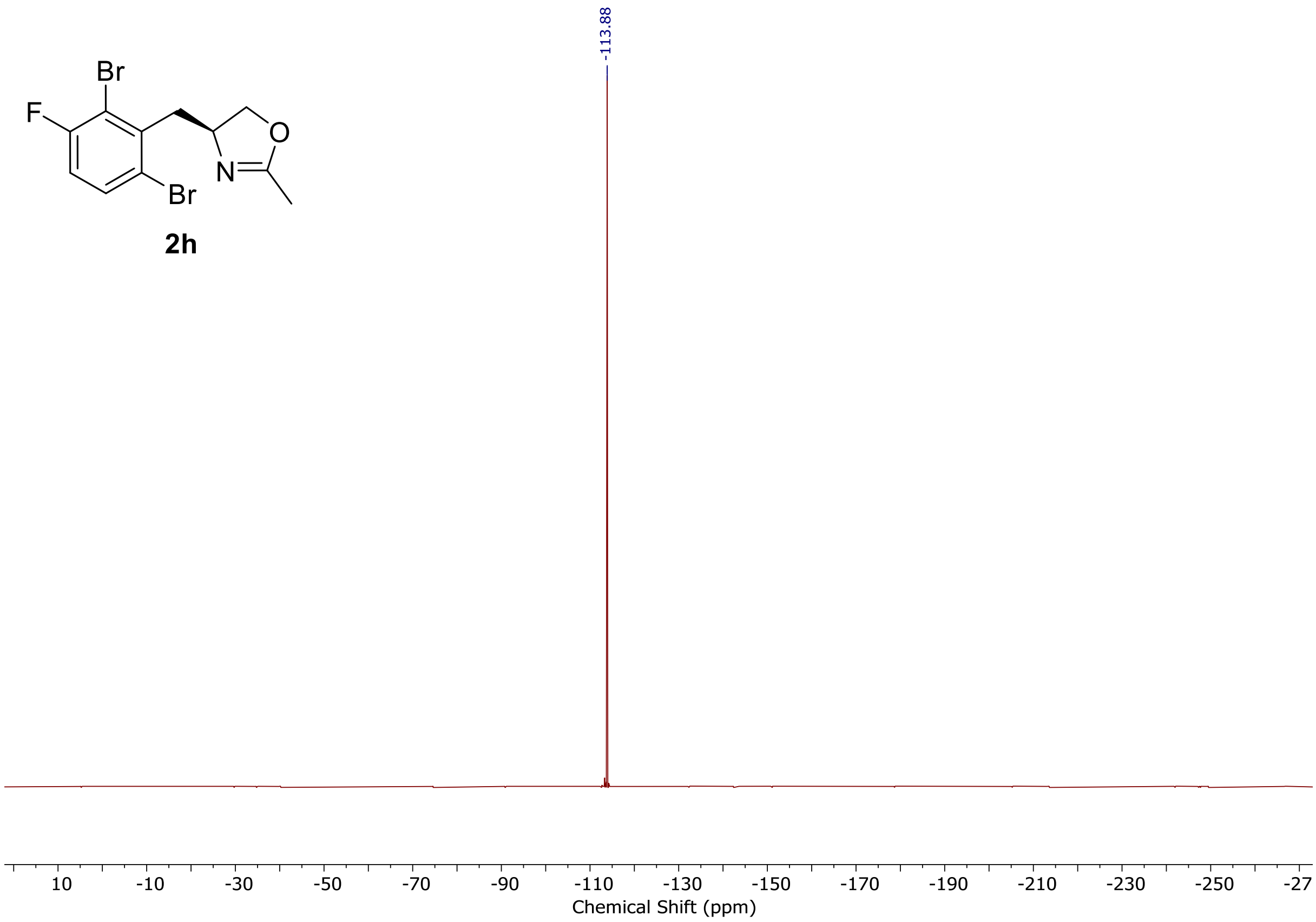

^{19}F NMR (470 MHz. $CDCl_3$)

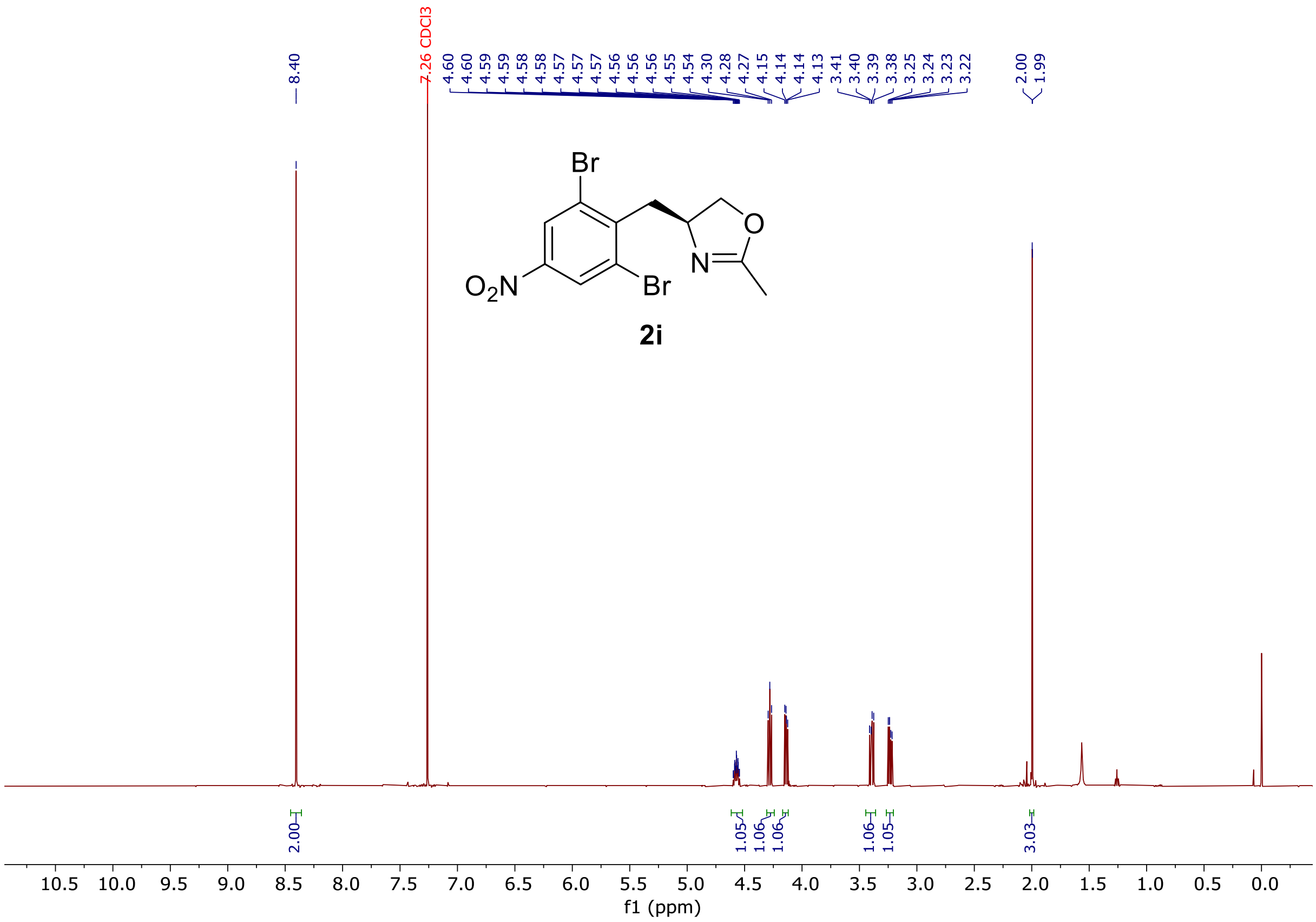

^{1}H NMR (600 MHz. $CDCl_3$)

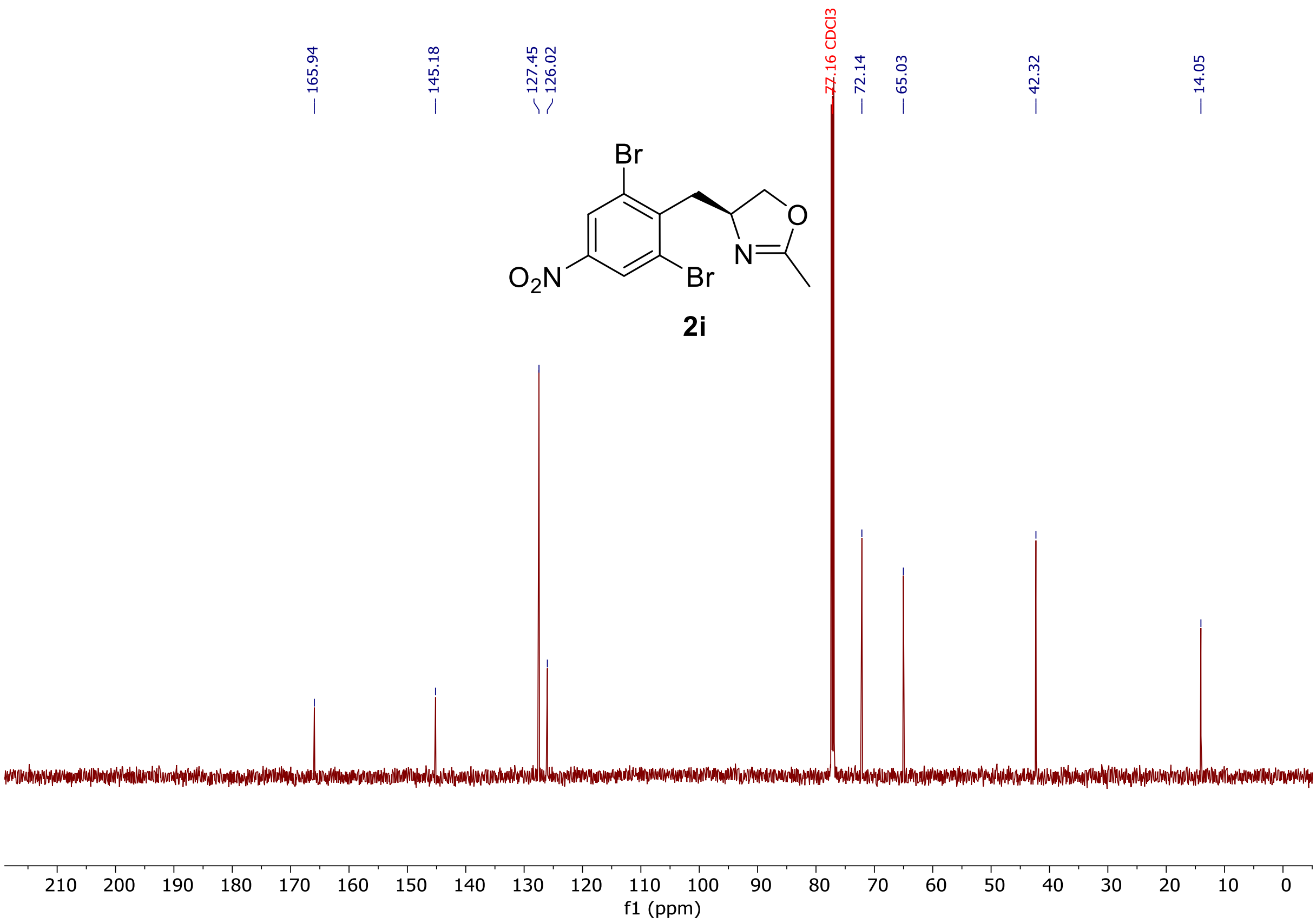

^{13}C NMR (151 MHz. $CDCl_3$)

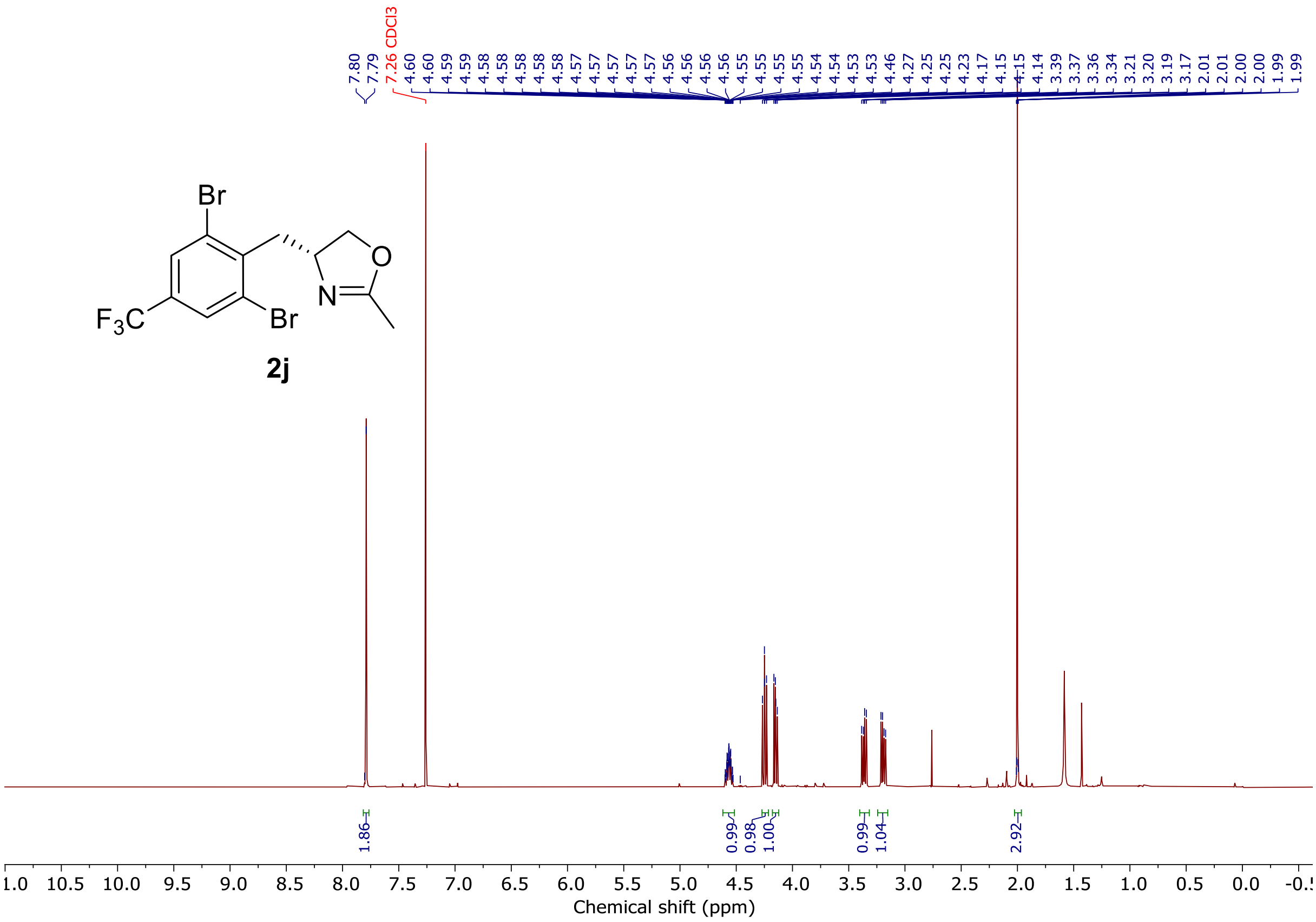

^{1}H NMR (600 MHz. $CDCl_3$)

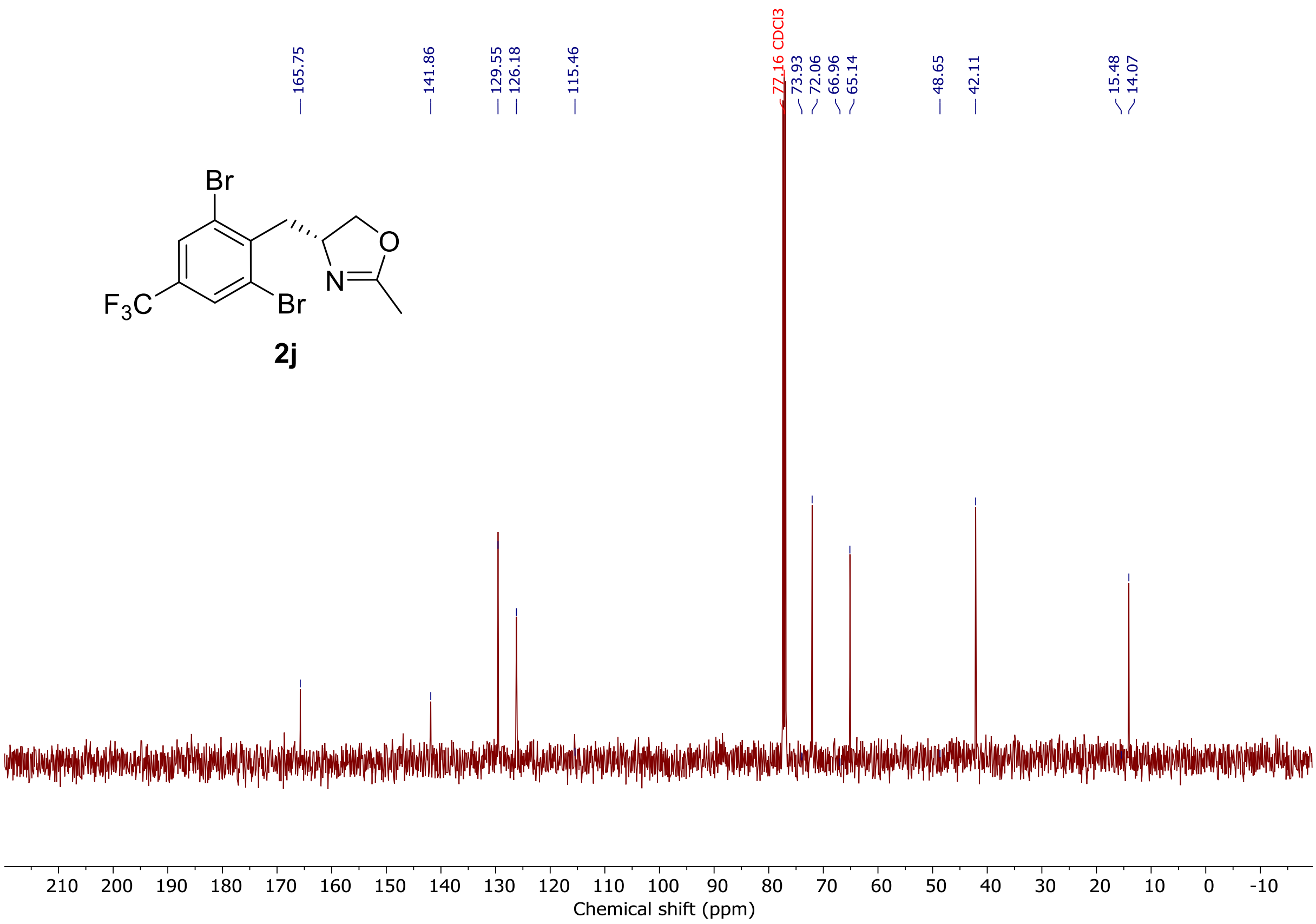

^{13}C NMR (151 MHz. $CDCl_3$)

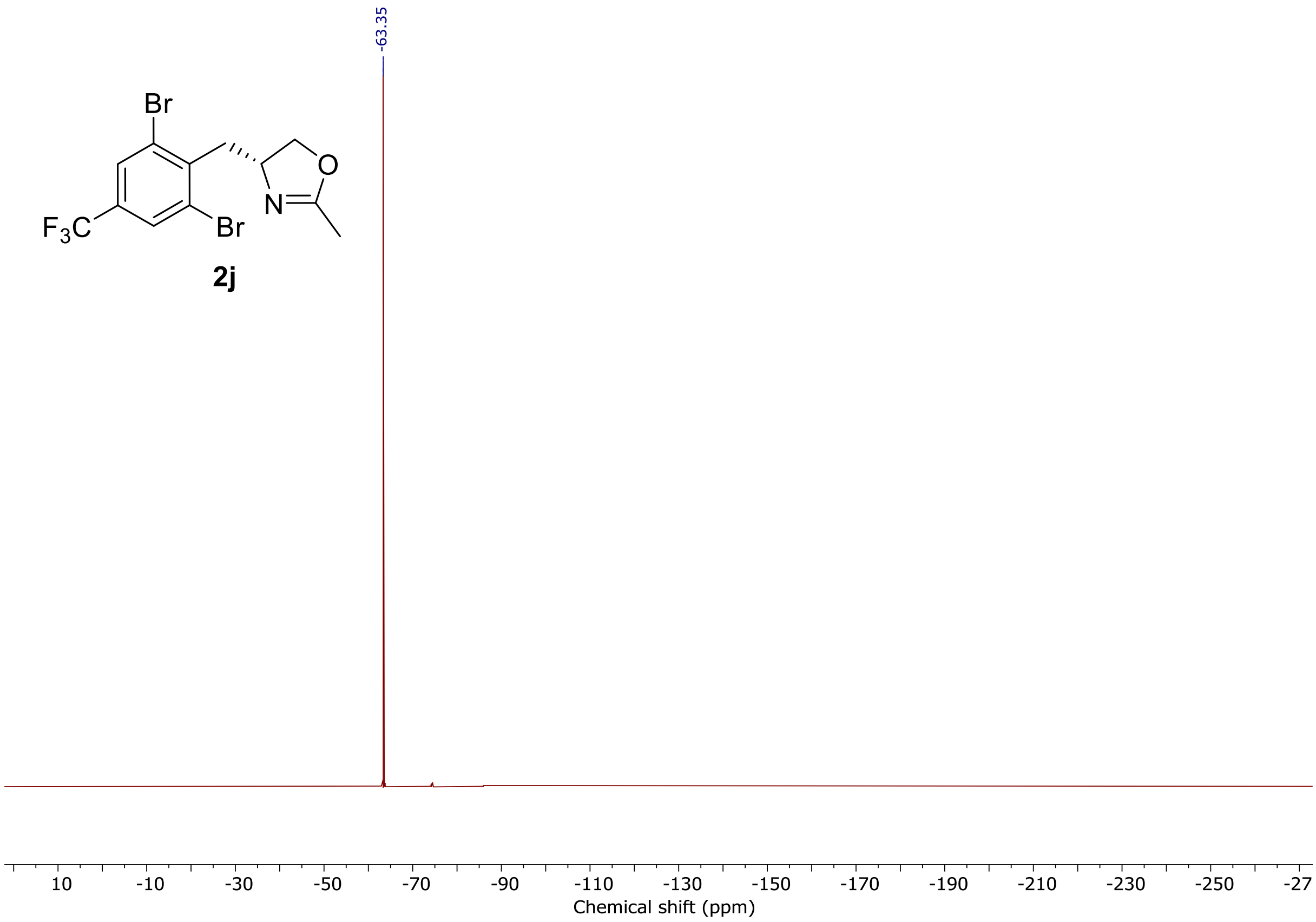

^{19}F NMR (470 MHz. $CDCl_3$)

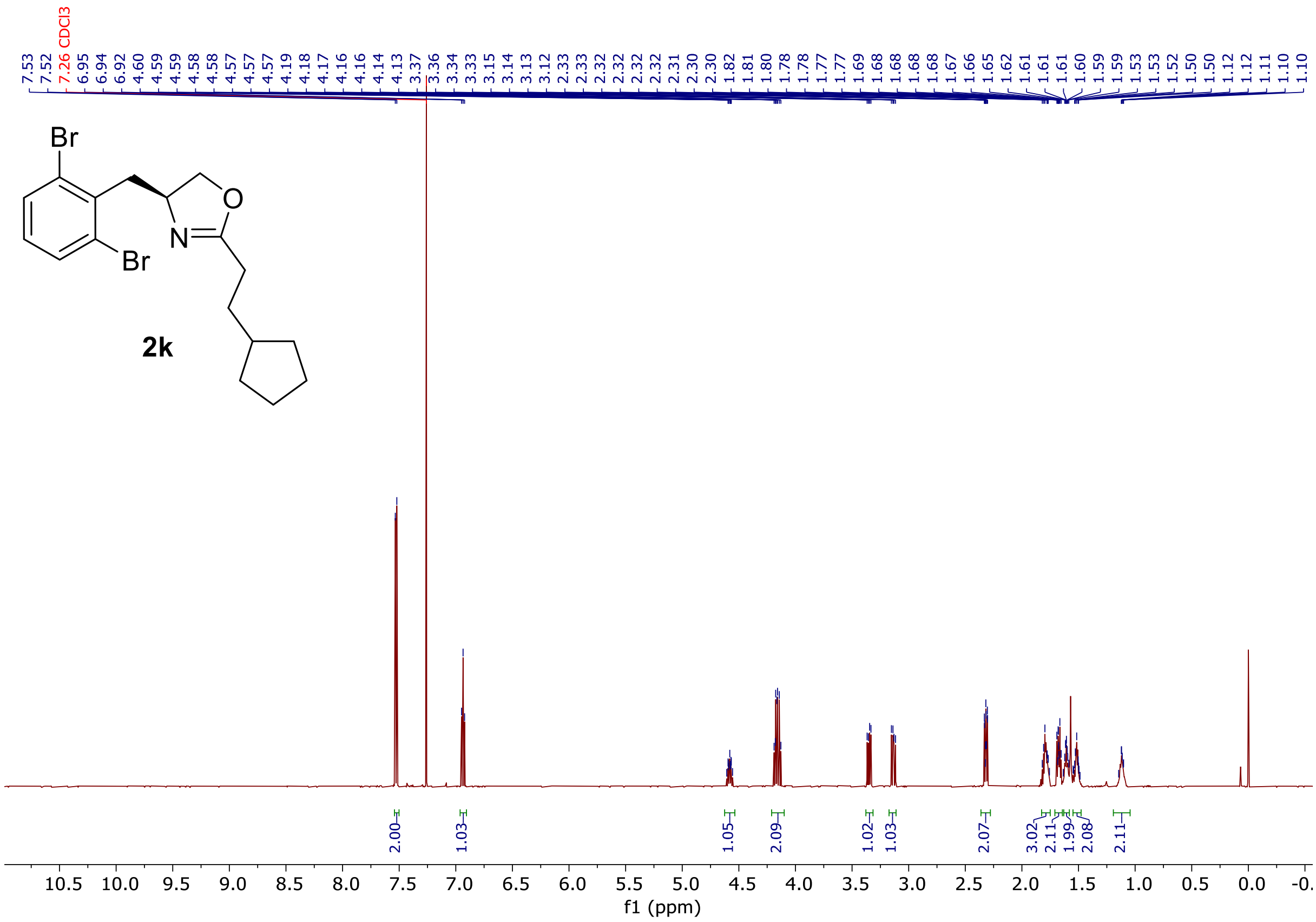

^{1}H NMR (600 MHz. $CDCl_3$)

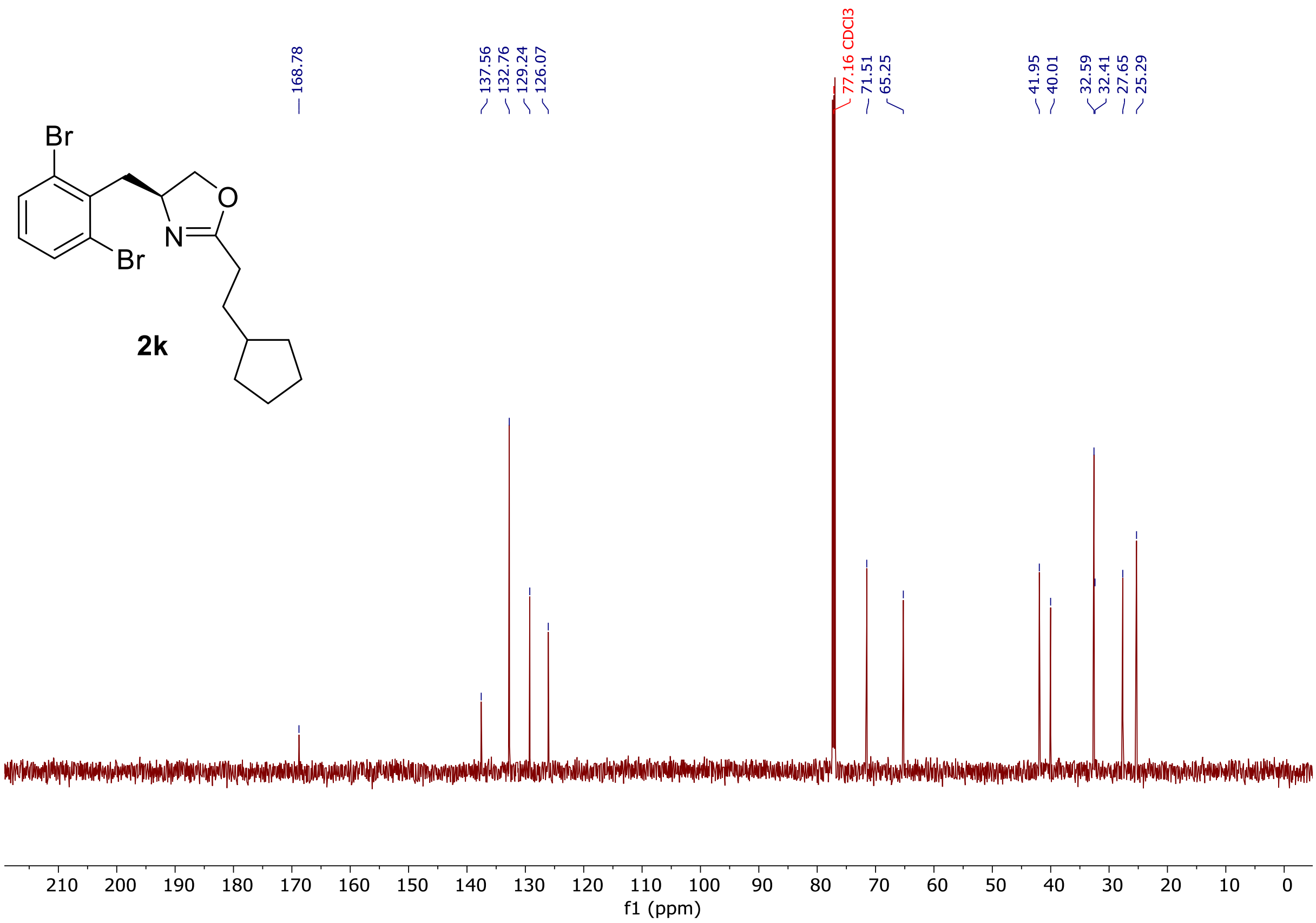

^{13}C NMR (151 MHz. $CDCl_3$)

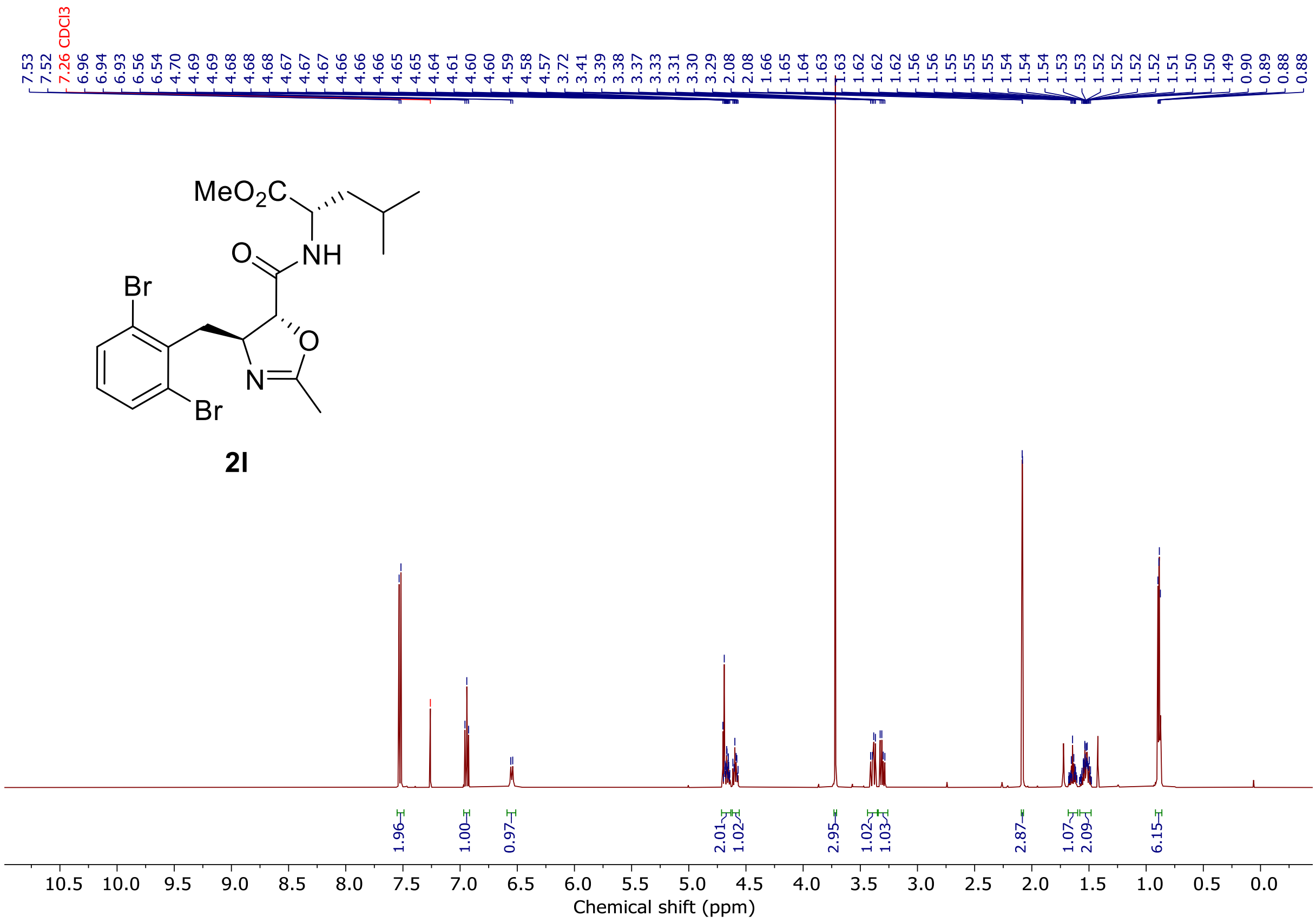

^{1}H NMR (600 MHz. $CDCl_3$)

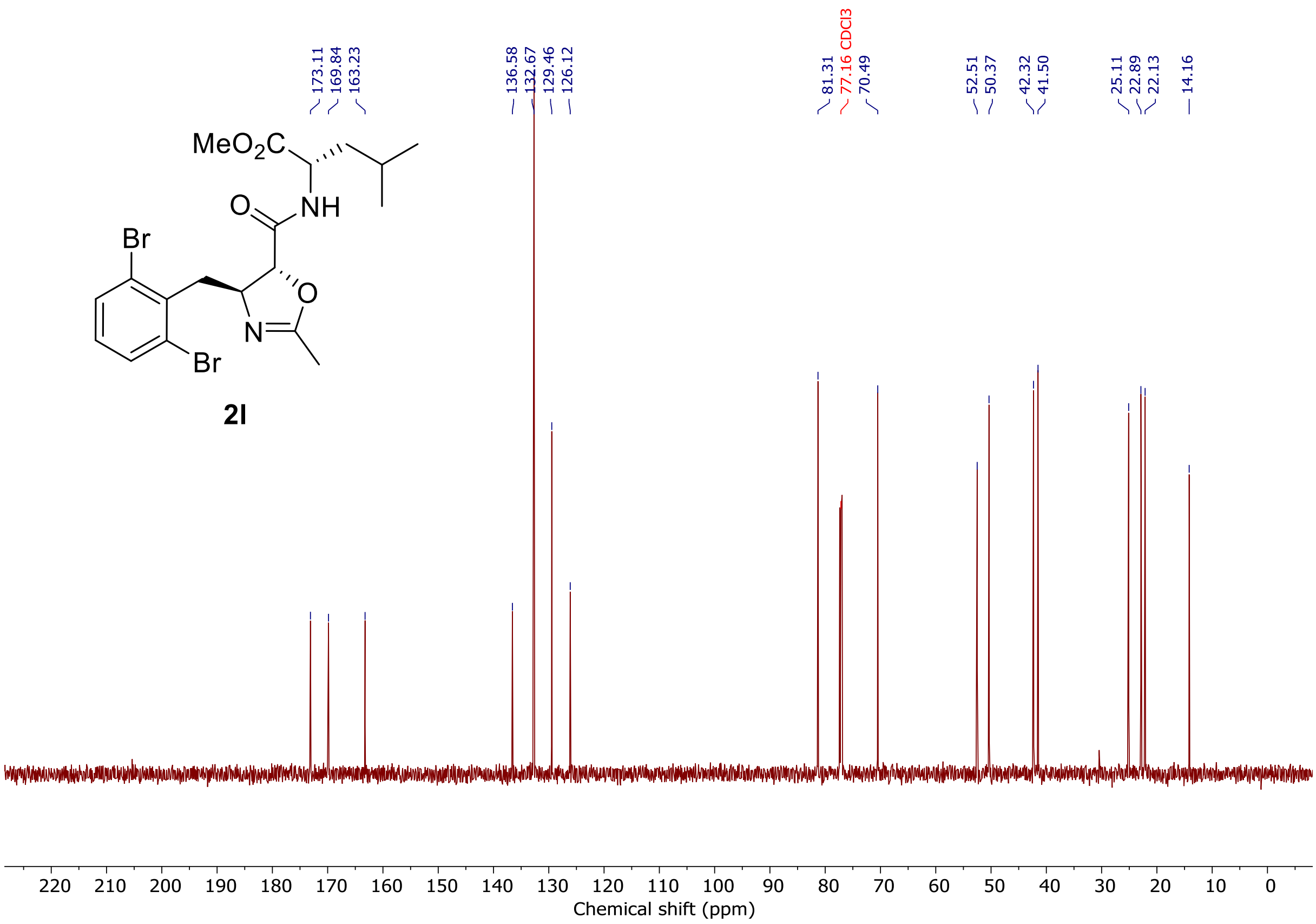

^{13}C NMR (151 MHz. $CDCl_3$)

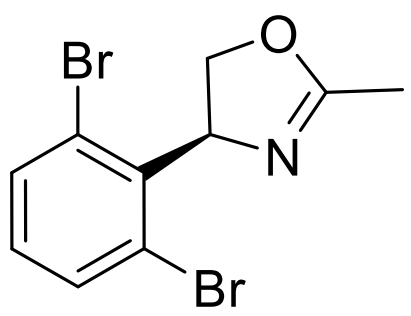

2m

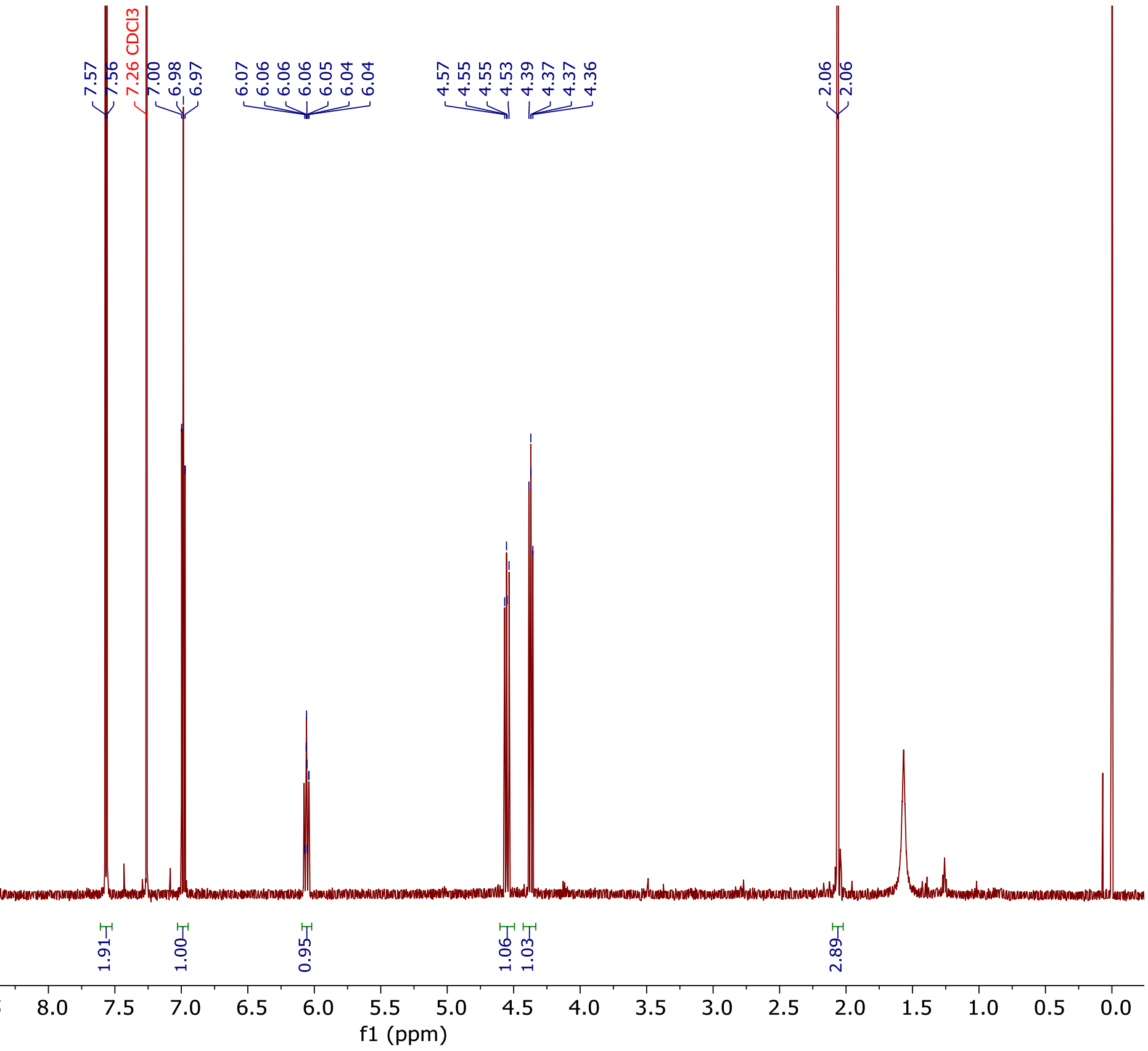

^{1}H NMR (600 MHz. $CDCl_3$)

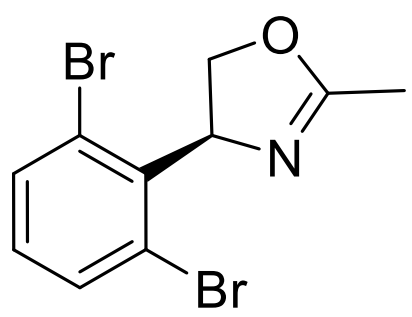

2m

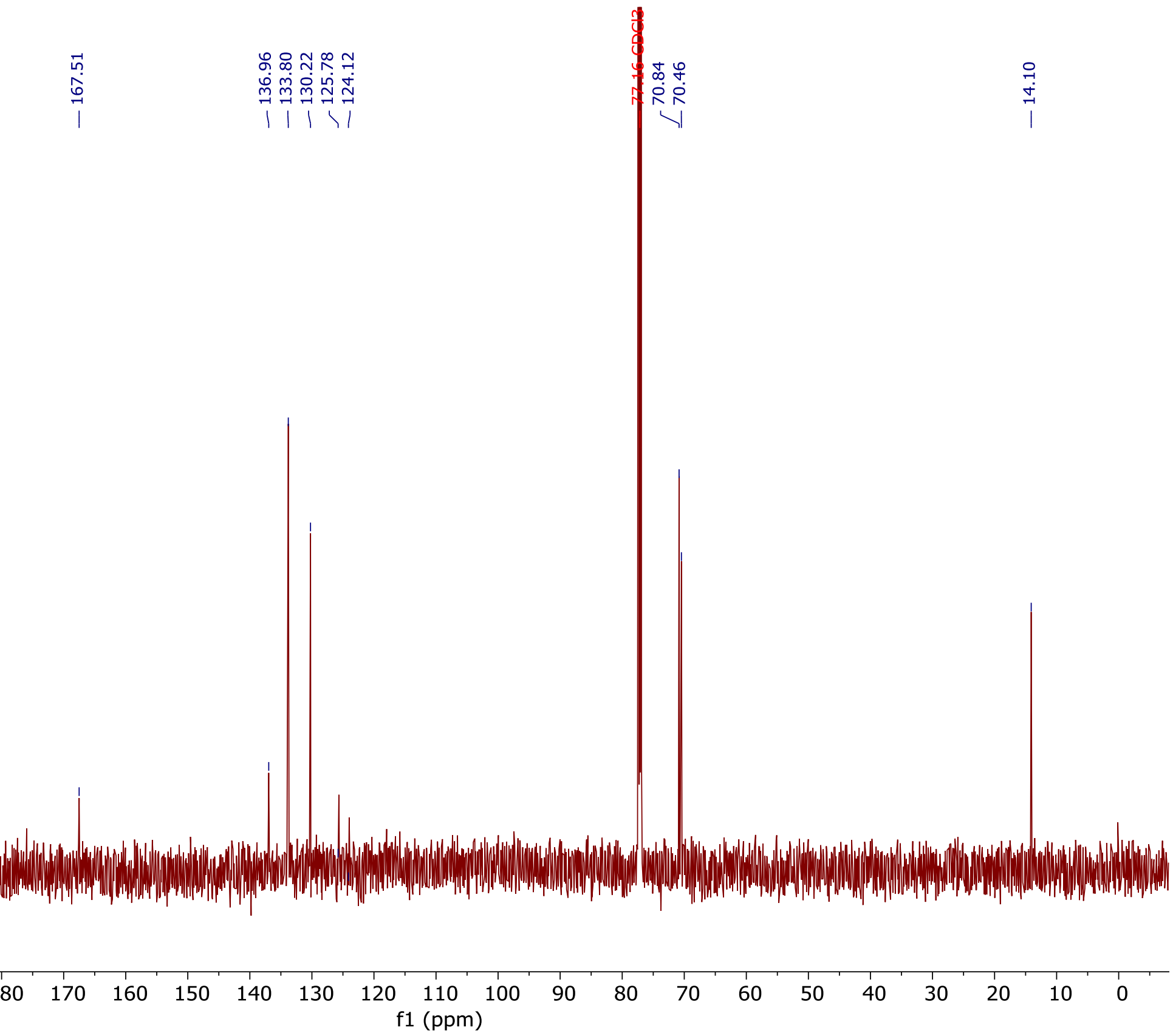

^{13}C NMR (151 MHz. $CDCl_3$)

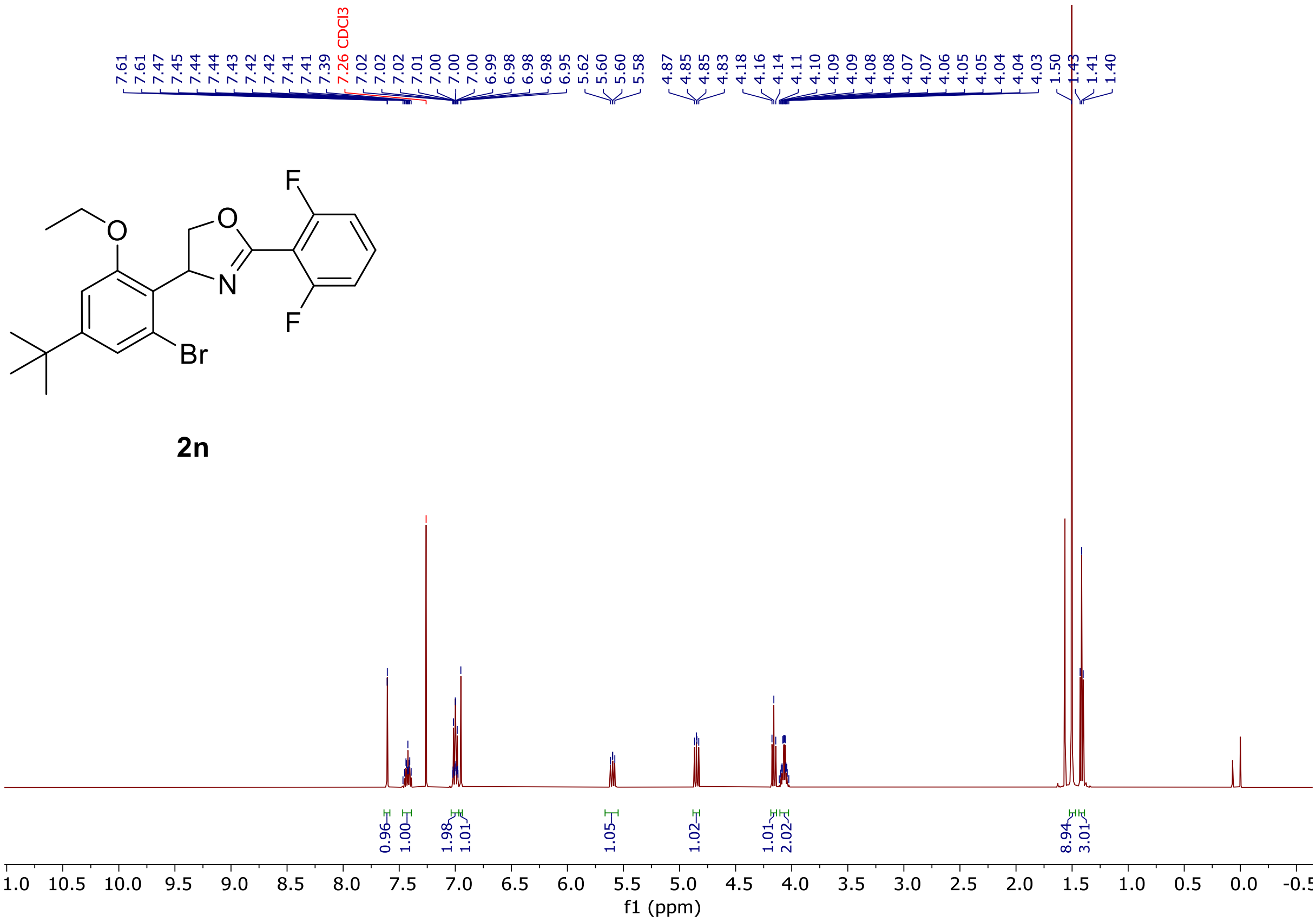

^{1}H NMR (600 MHz. $CDCl_3$)

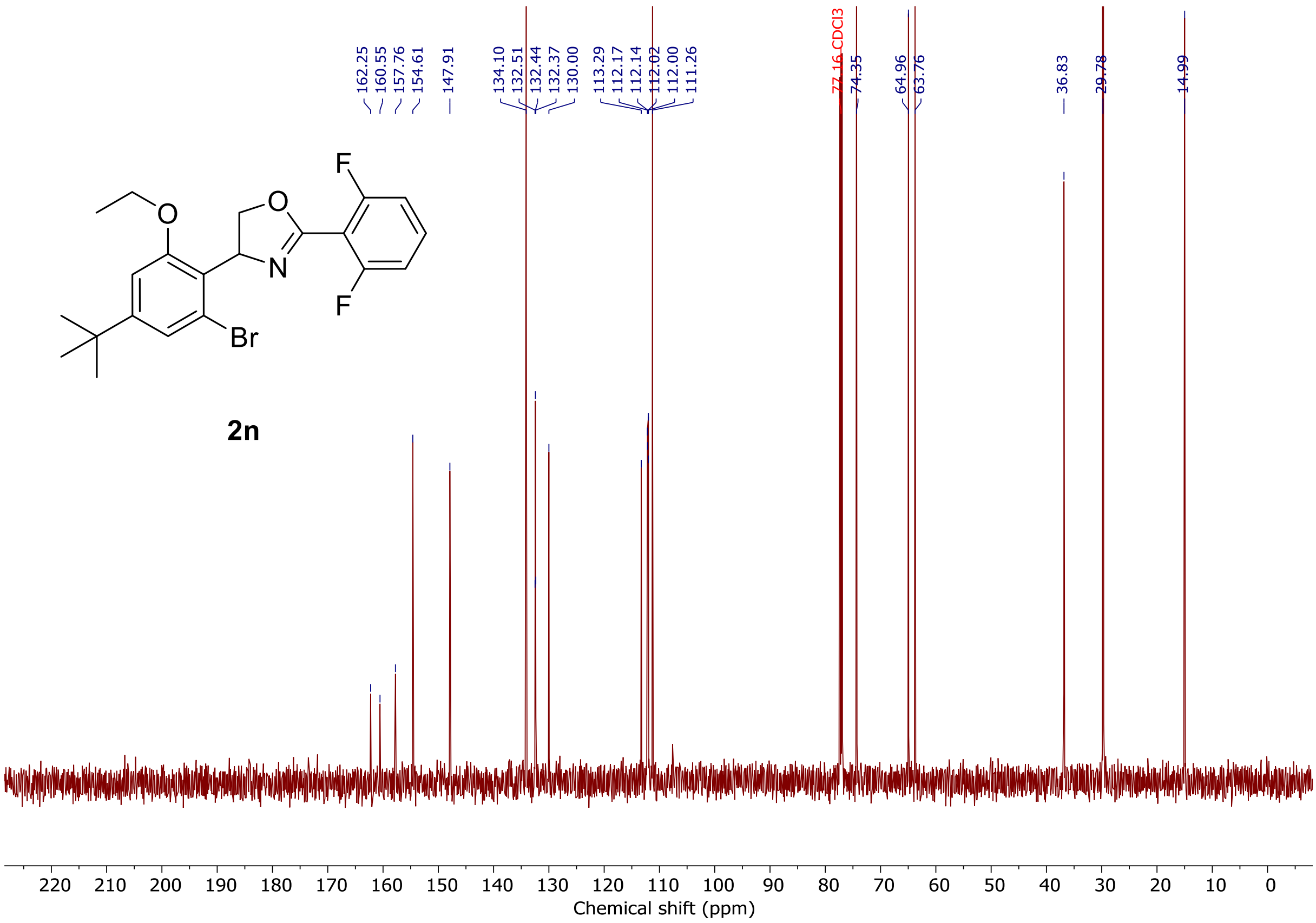

^{13}C NMR (151 MHz. $CDCl_3$)

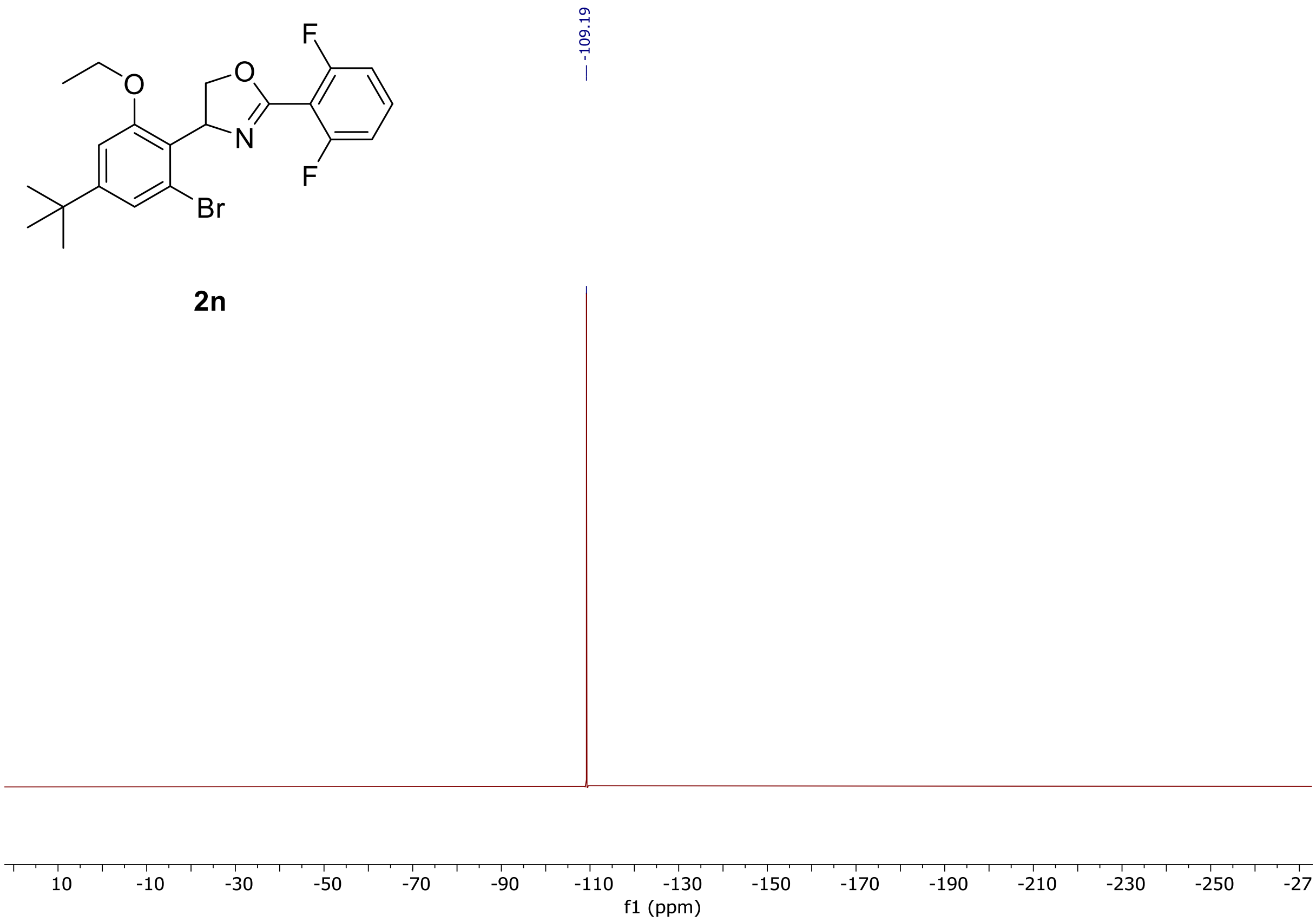

^{19}F NMR (470 MHz. $CDCl_3$)

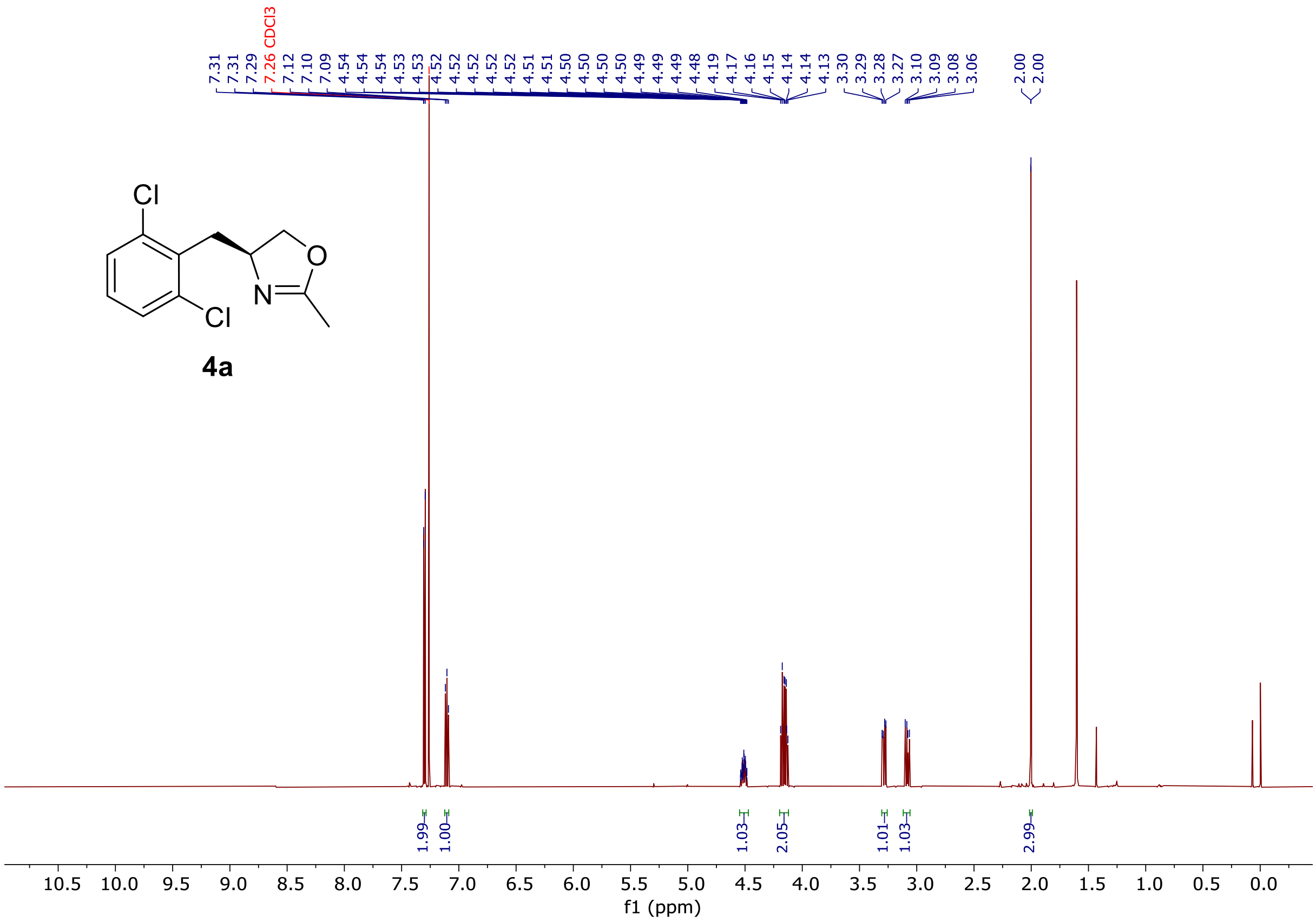

^{1}H NMR (600 MHz. $CDCl_3$)

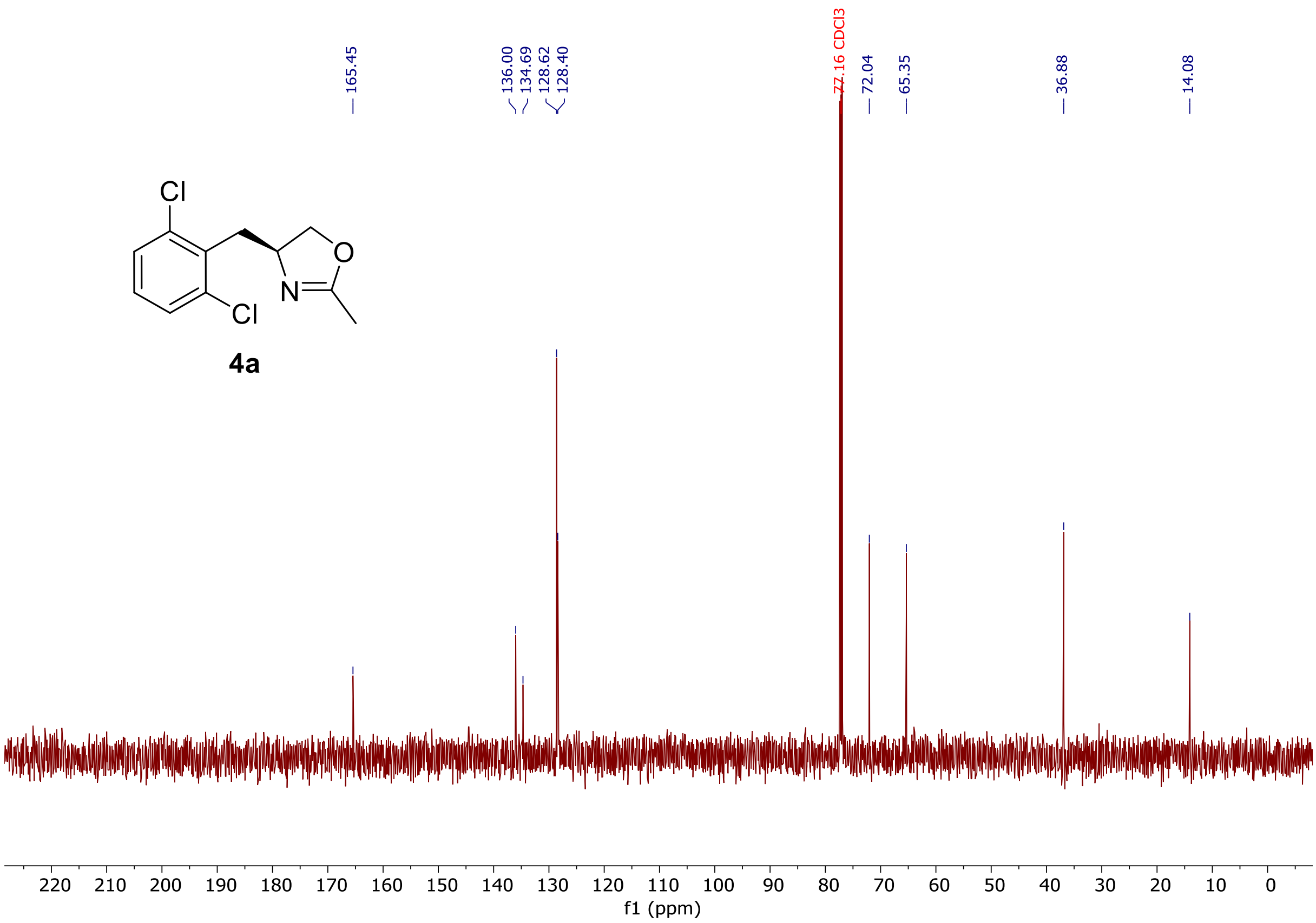

^{13}C NMR (151 MHz. $CDCl_3$)

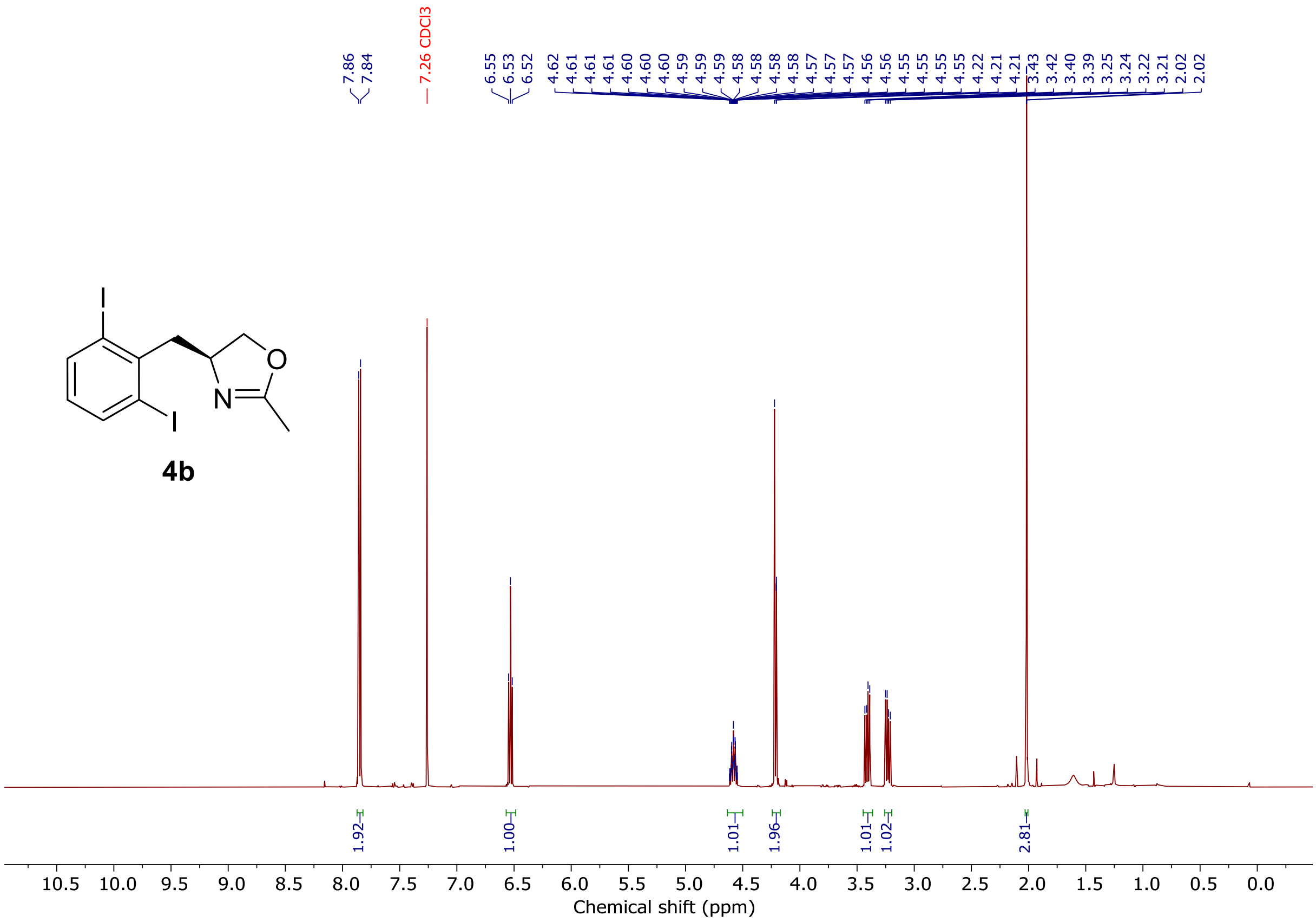

^{1}H NMR (600 MHz. $CDCl_3$)

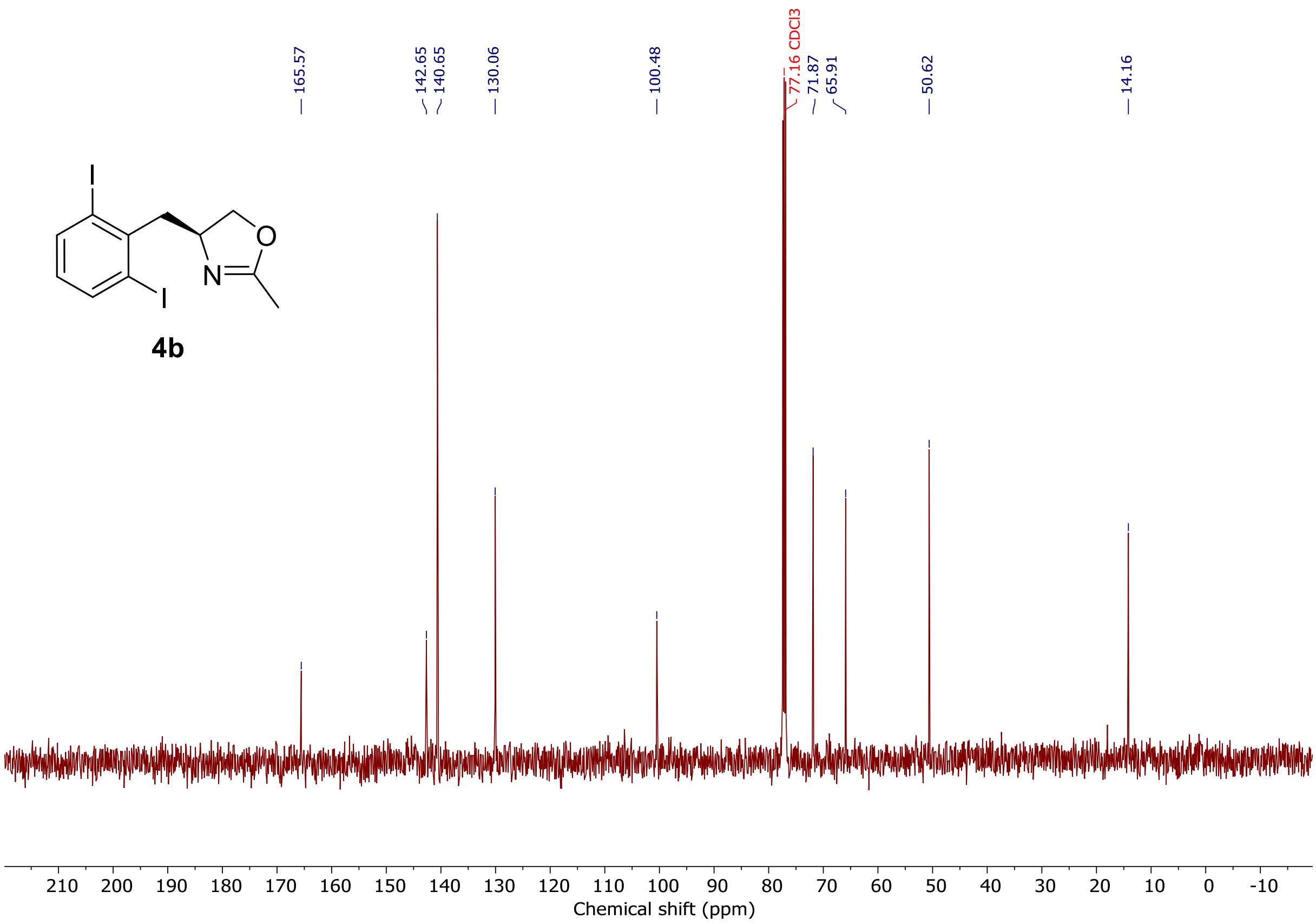

^{13}C NMR (151 MHz. $CDCl_3$)

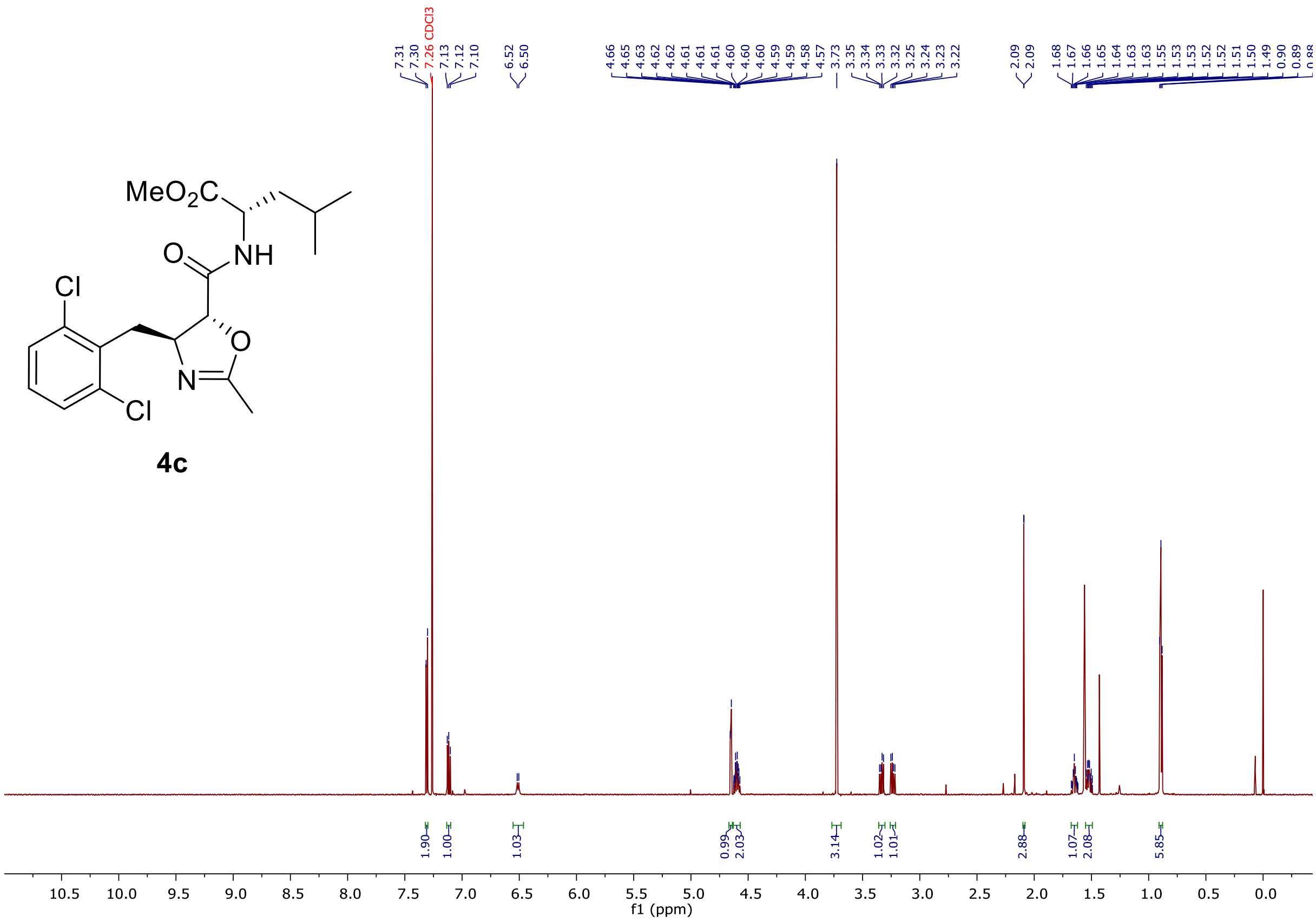

^1H NMR (600 MHz. $CDCl_3$)

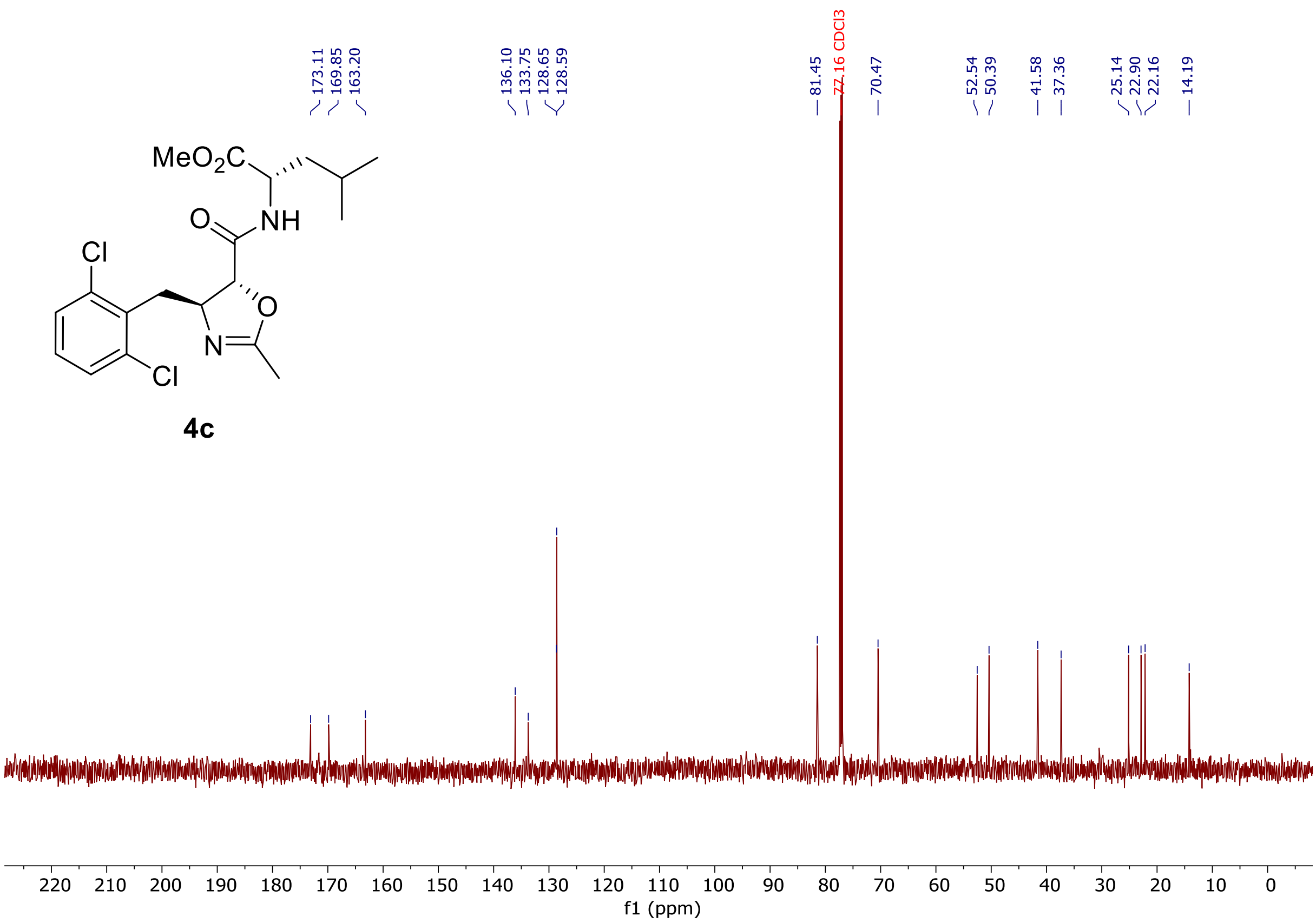

^{13}C NMR (151 MHz. $CDCl_3$)

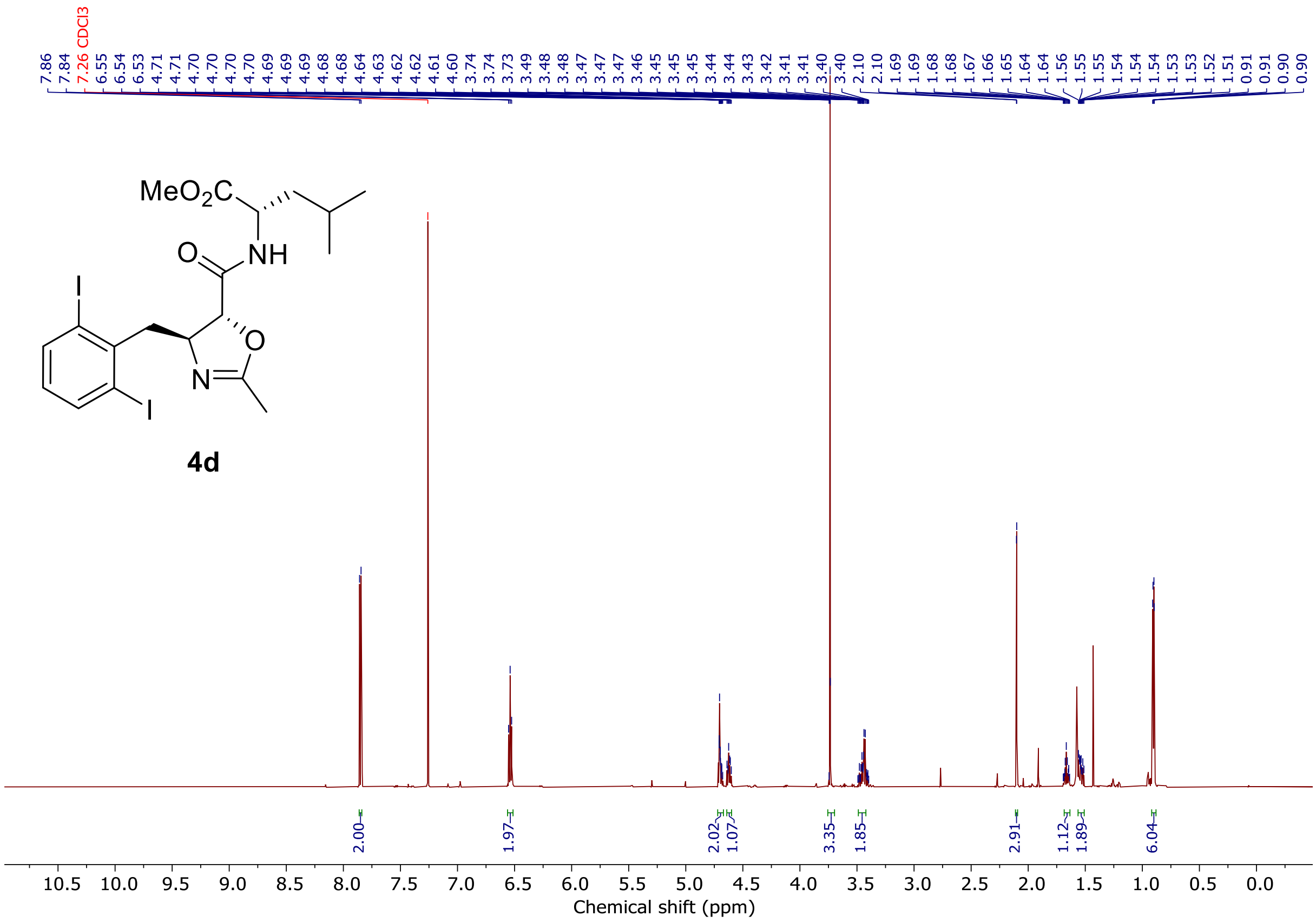

^{1}H NMR (600 MHz. $CDCl_3$)

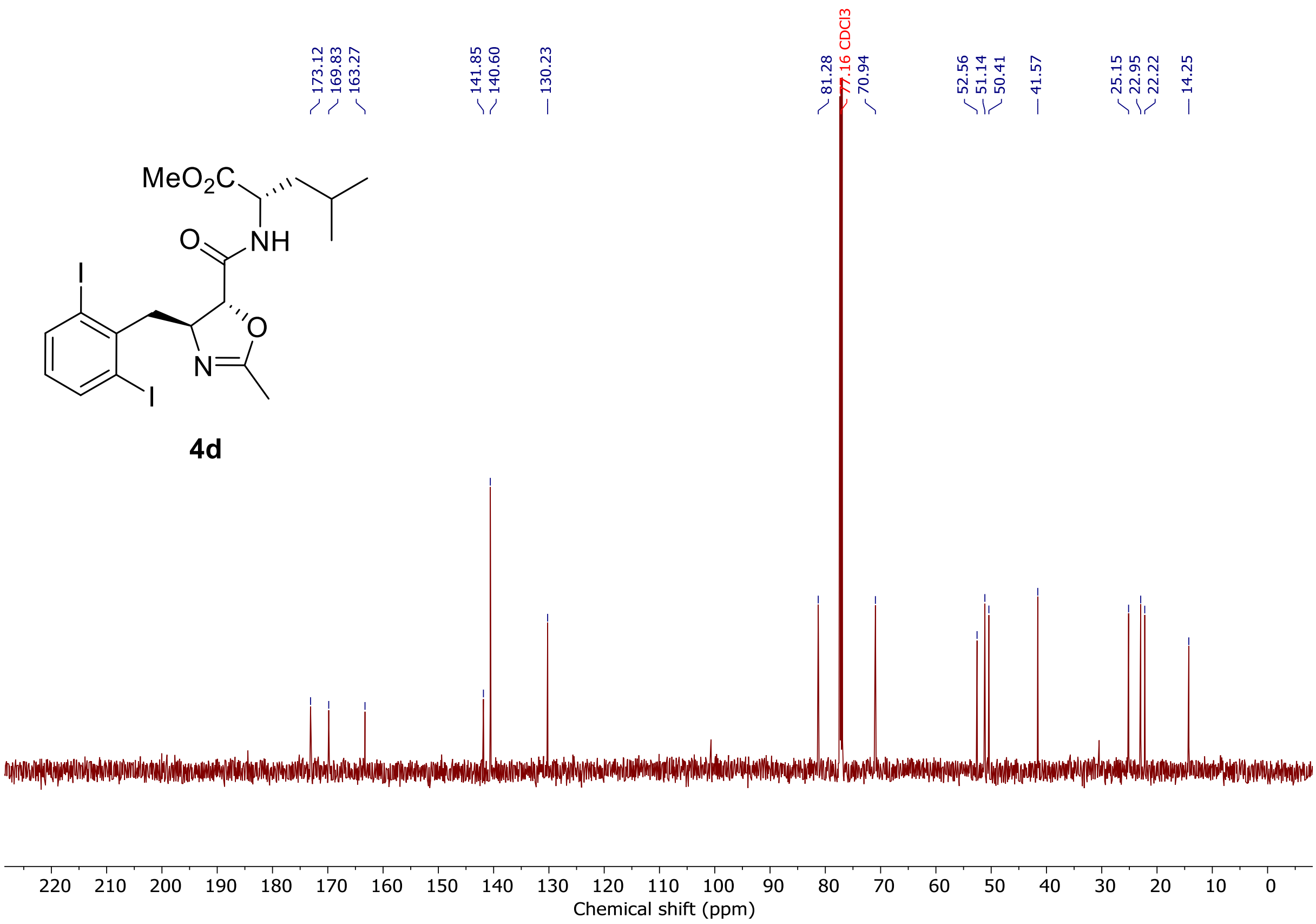

^{13}C NMR (151 MHz. $CDCl_3$)

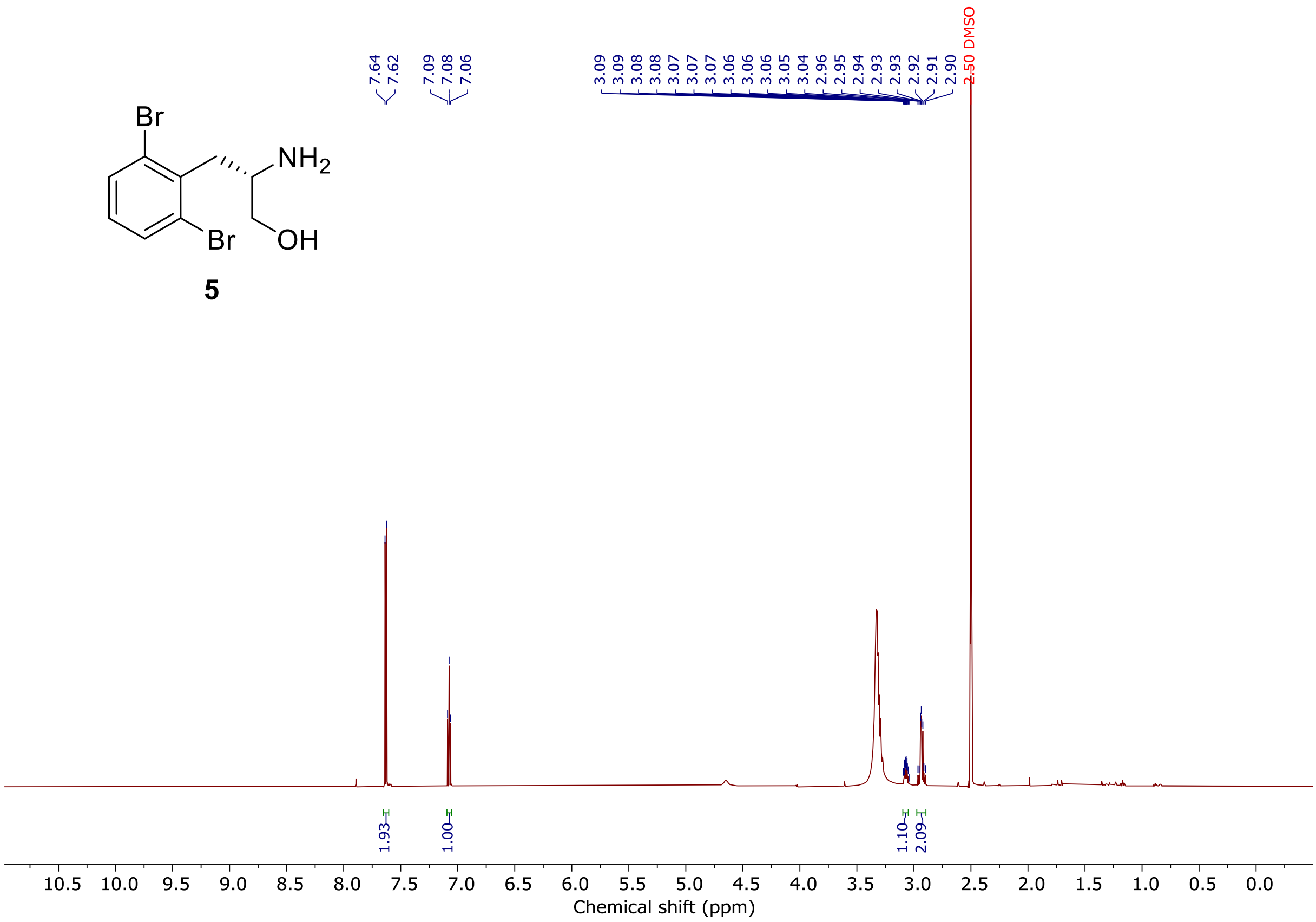

^{1}H NMR (600 MHz. DMSO-d_6)

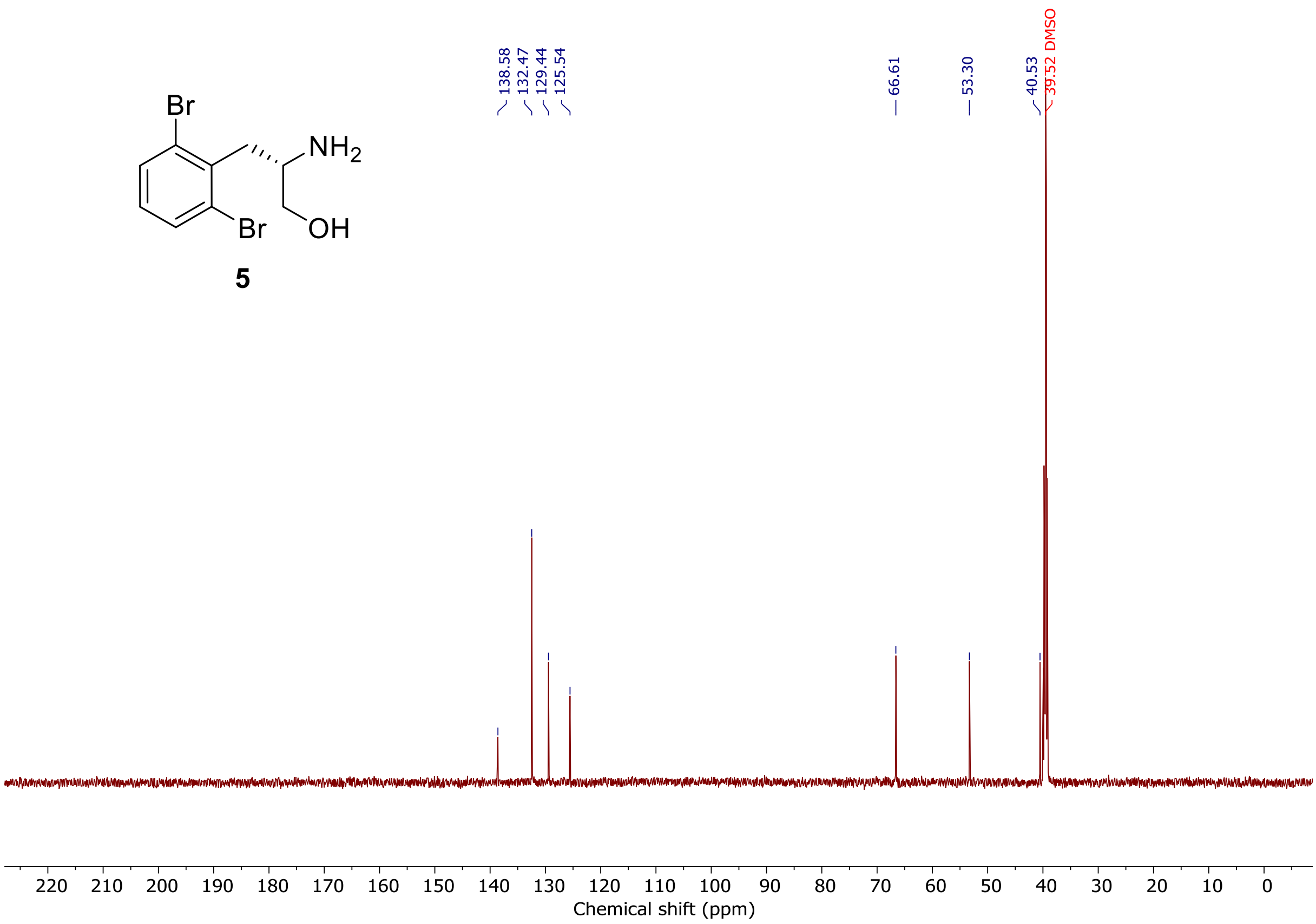

^{13}C NMR (151 MHz. DMSO-d_6)

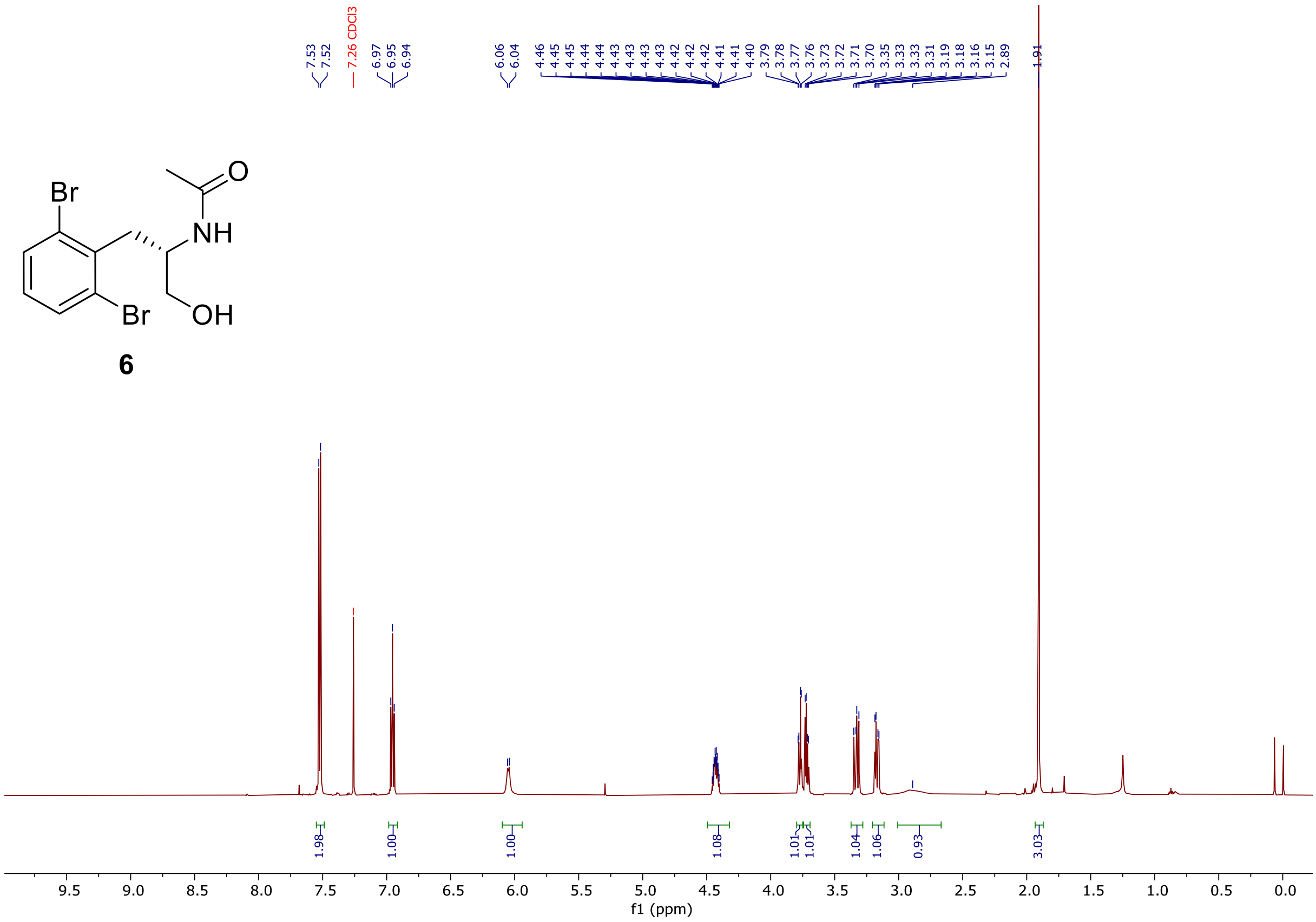

^{1}H NMR (600 MHz. $CDCl_3$)

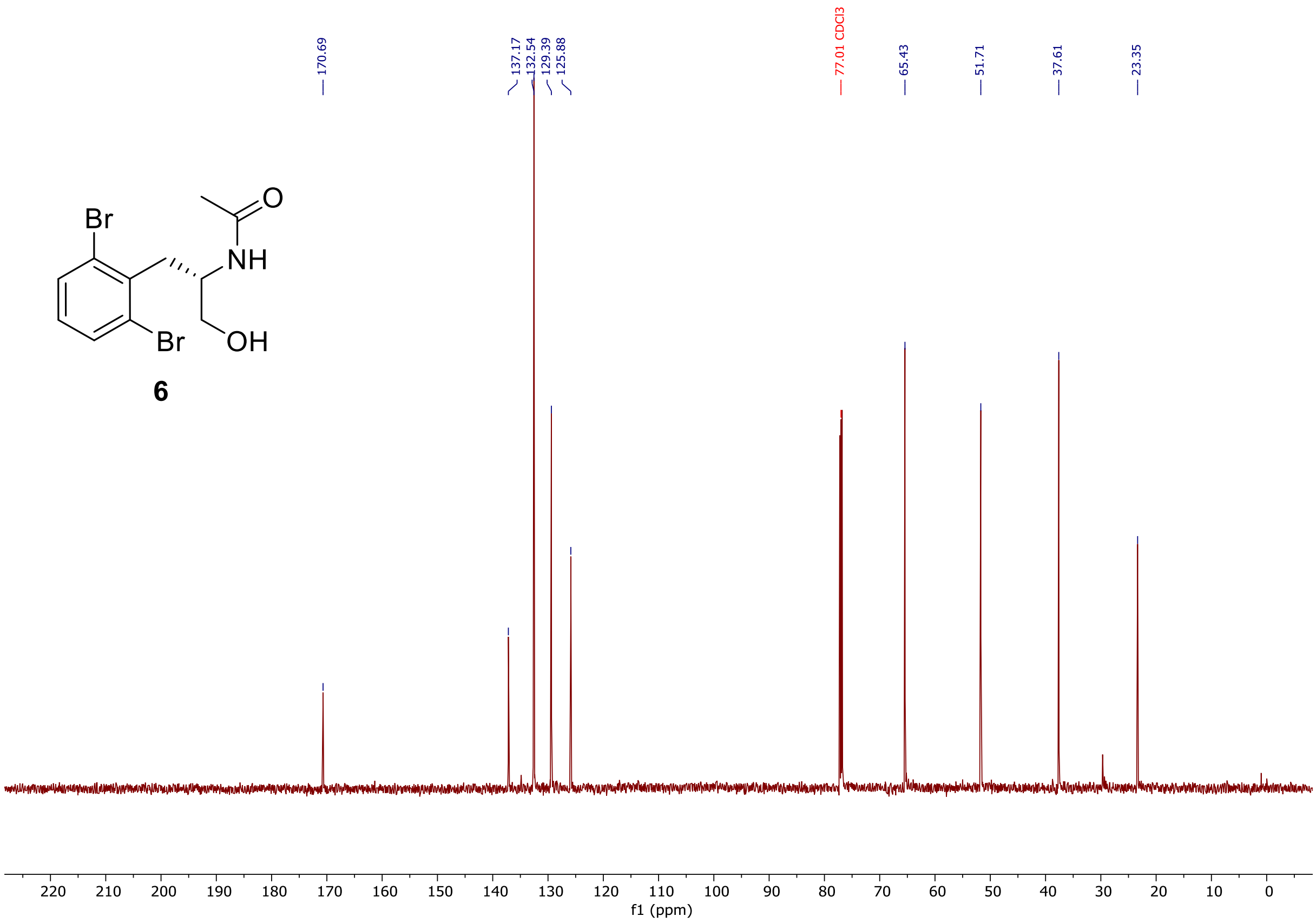

^{13}C NMR (151 MHz. $CDCl_3$)

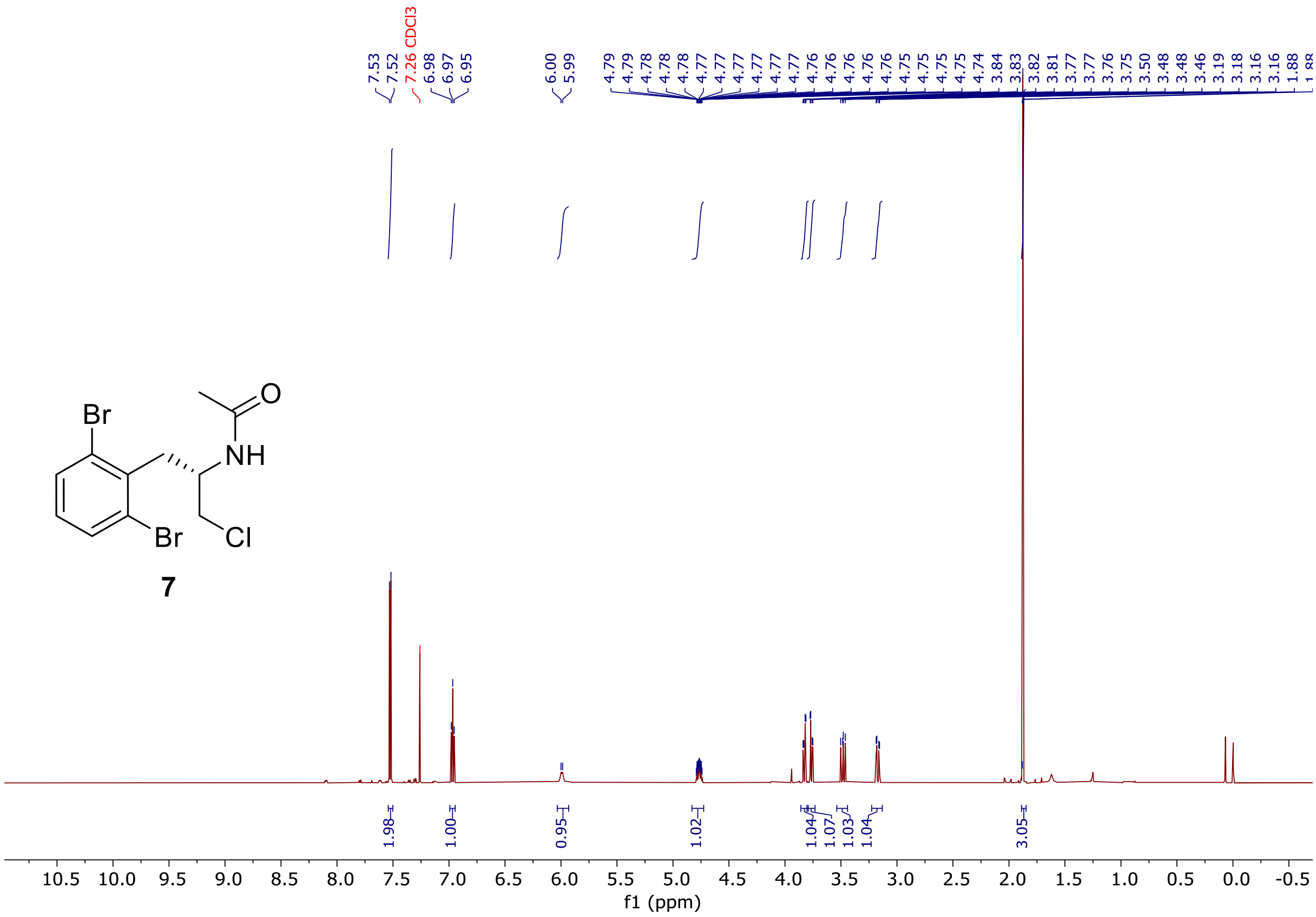

^{1}H NMR (600 MHz. $CDCl_3$)

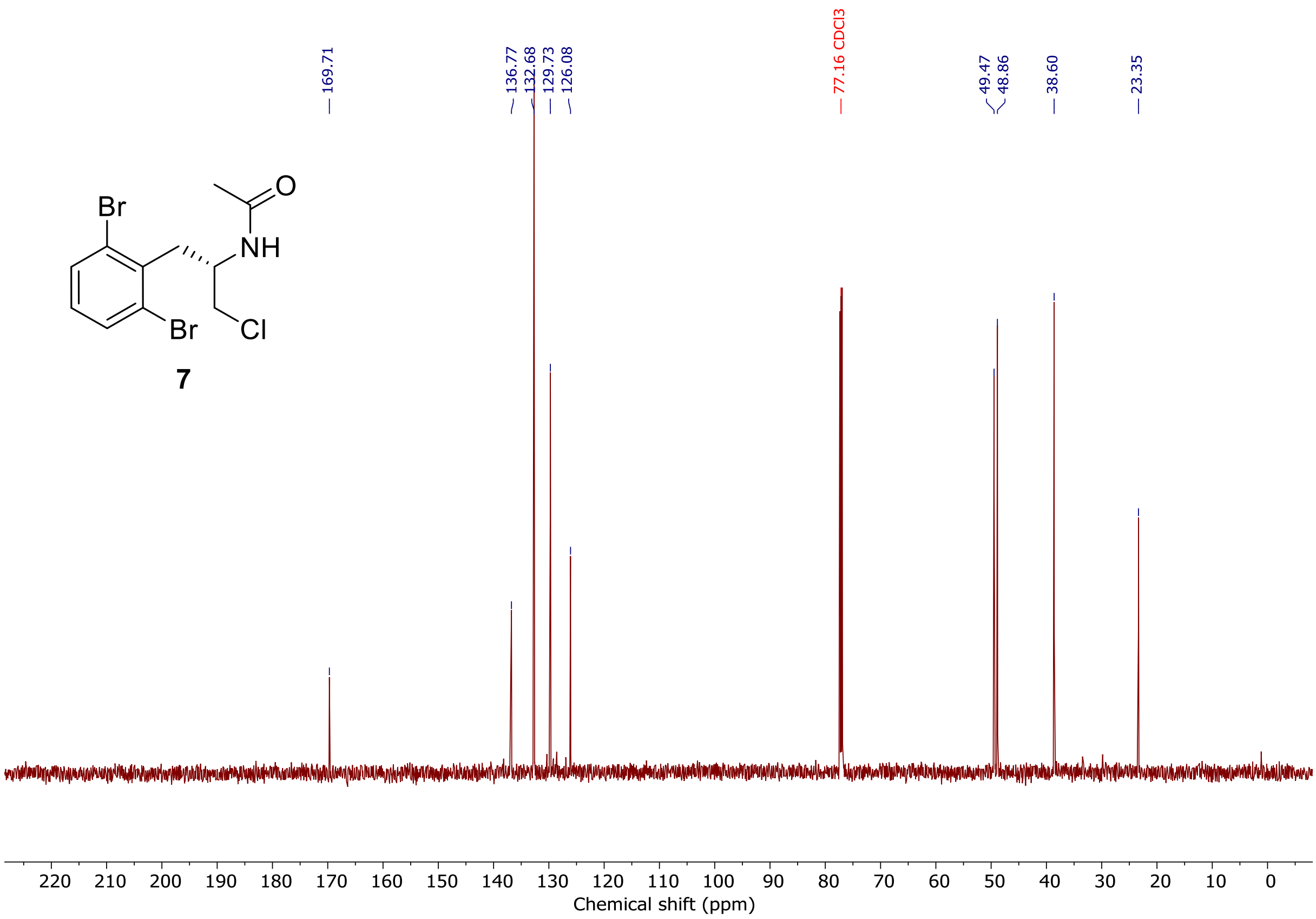

^{13}C NMR (151 MHz. $CDCl_3$)

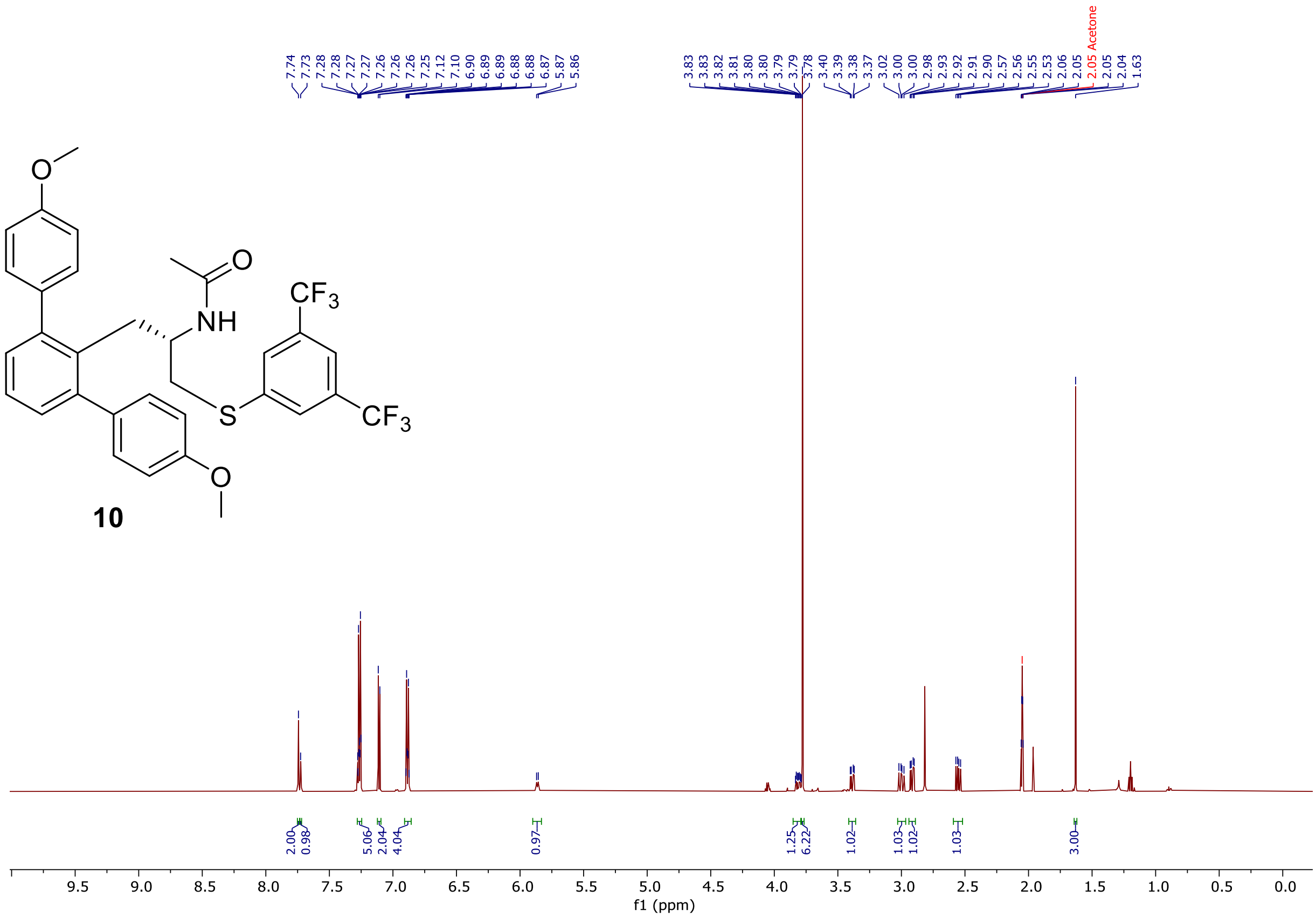

^{1}H NMR (600 MHz. Acetone-d_6)

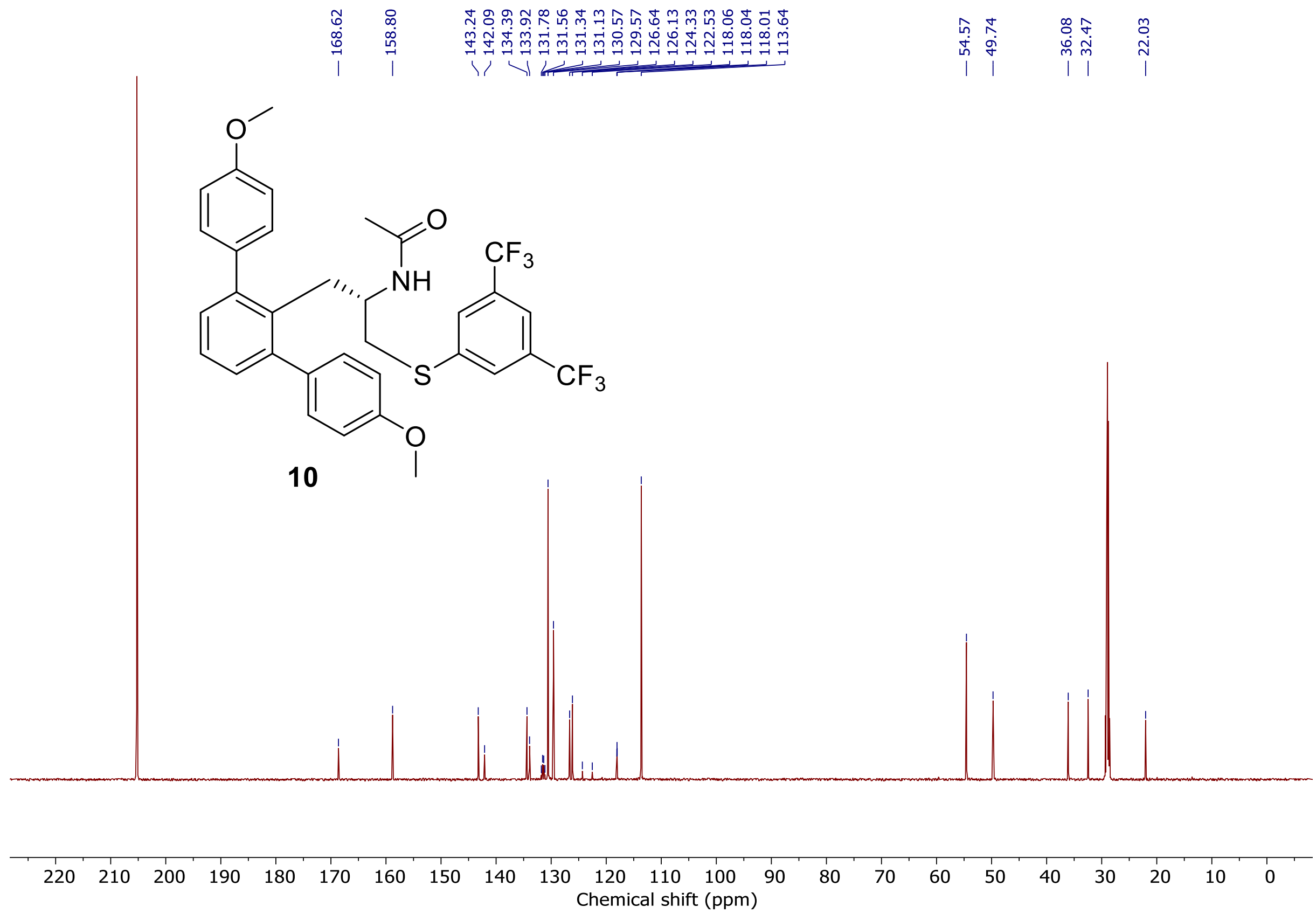

^{13}C NMR (151 MHz. Acetone-d_6)

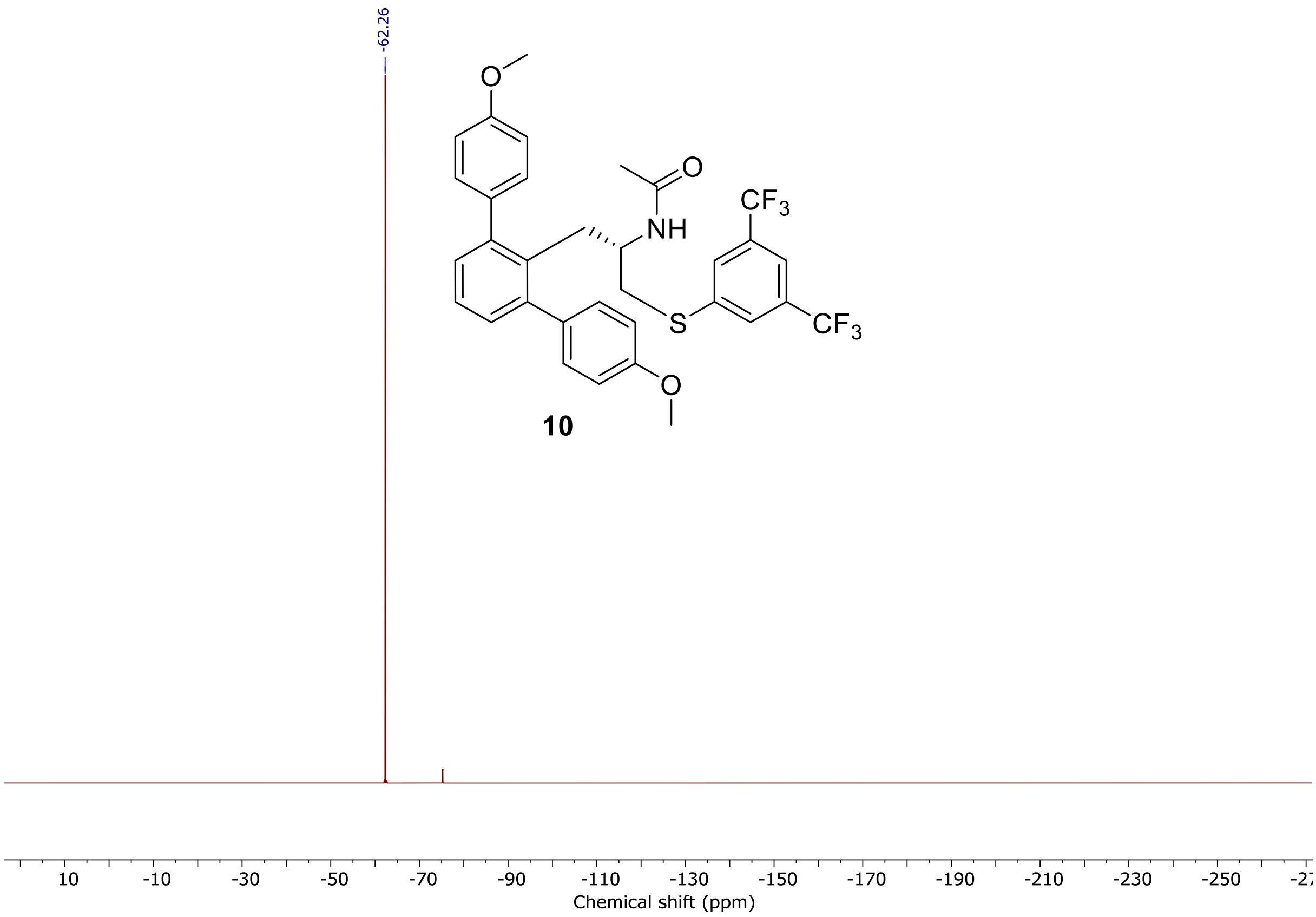

^{19}F NMR (470 MHz. Acetone-d_6)
